# Relativistic dynamics and extreme mass ratio inspirals

**DOI:** 10.1007/s41114-018-0013-8

**Published:** 2018-05-15

**Authors:** Pau Amaro-Seoane

**Affiliations:** 1 Institute of Space Sciences (ICE, CSIC), Institut d’Estudis Espacials de Catalunya (IEEC) at Campus UAB, Carrer de Can Magrans s/n, 08193 Barcelona, Spain; 2 Institute of Applied Mathematics, Academy of Mathematics and Systems Science, CAS, Beijing, 100190 China; 3 Kavli Institute for Astronomy and Astrophysics, Beijing, 100871 China; 4 Zentrum für Astronomie und Astrophysik, TU Berlin, Hardenbergstraße 36, 10623 Berlin, Germany

**Keywords:** Black holes, Gravitational waves, Stellar dynamics

## Abstract

It is now well-established that a dark, compact object, very likely a massive black hole (MBH) of around four million solar masses is lurking at the centre of the Milky Way. While a consensus is emerging about the origin and growth of supermassive black holes (with masses larger than a billion solar masses), MBHs with smaller masses, such as the one in our galactic centre, remain understudied and enigmatic. The key to understanding these holes—how some of them grow by orders of magnitude in mass—lies in understanding the dynamics of the stars in the galactic neighbourhood. Stars interact with the central MBH primarily through their gradual inspiral due to the emission of gravitational radiation. Also stars produce gases which will subsequently be accreted by the MBH through collisions and disruptions brought about by the strong central tidal field. Such processes can contribute significantly to the mass of the MBH and progress in understanding them requires theoretical work in preparation for future gravitational radiation millihertz missions and X-ray observatories. In particular, a unique probe of these regions is the gravitational radiation that is emitted by some compact stars very close to the black holes and which could be surveyed by a millihertz gravitational-wave interferometer scrutinizing the range of masses fundamental to understanding the origin and growth of supermassive black holes. By extracting the information carried by the gravitational radiation, we can determine the mass and spin of the central MBH with unprecedented precision and we can determine how the holes “eat” stars that happen to be near them.

## Foreword

The volume where capture orbits are produced is so small in comparison to other typical length scales of interest in astrodynamics that it has usually been seen as unimportant and irrelevant to the global dynamical evolution of the system. The only exception has been the tidal disruption of stars by massive black holes. Only when it transpired that the slow, adiabatic inspiral of compact objects onto massive black holes provides us with valuable information, did astrophysicists start to address the question in more detail. Since the problem of EMRIs (extreme mass ratio inspiral) started to draw our attention, there has been a notable progress in answering fundamental questions of stellar dynamics. The discoveries have been numerous and some of them remain puzzling. The field is developing very quickly and we are making important breakthroughs even before a millihertz mission flies.

When I was approached and asked to write this review, I was glad to accept it without realising the dimensions of the task. I was told that it should be * similar to a plenary talk for a wide audience*. I have a personal problem with instructions like this. I remember that when I was nine years old, our Spanish teacher asked us to summarise a story we had read together in class. I asked her to define “summarise”, because I could easily produce a summary of one, two or fifty pages, depending on what she was actually expecting from us. She was confused and I never got a clear answer. She replied that “A summary is a summary and that’s it”. On this occasion, I am afraid that I have run into the same snag and I have gone for the many-pages approach, to be sure that any newcomer will have a good overview of the subject, with relevant references, in a single document. If the document is too long, please address your complains to her, because she is solely responsible.

However, I would like to note that I have *not* focused on gathering as much information as possible from different sources. I think it is more interesting for the reader, though harder for the writer, to have a consistent document. This can be done by introducing the subject step by step, rather than working out a compendium of citations of the related literature. For instance, I present results that I have not previously published that will, I hope, enlighten the reader. Figures that I prepared myself and are not published elsewhere do not have a reference.

From the point of view of millihertz gravitational-wave (GW) missions, as the reader probably knows, the laser interferometer space antenna (LISA), see Amaro-Seoane et al. (2017), is now the official ESA L3 mission, already entering the phase A.

## Massive dark objects in galactic nuclei

Massive objects allowing no light to escape from them is a concept that goes back to the eighteenth century, when John Michell (1724–1793), an English natural philosopher and geologist, overtook Laplace by 12 years (see Montgomery et al. 2009) with the idea that a very massive object could be able to stop light escaping from it thanks to its overwhelming gravity. Such an object would be *black*, that is, invisible, precisely because of the lack of light (Michell 1784; Schaffer 1979). That is, a *dark star*. He wrote:

*If the semi-diameter of a sphere of the same density as the sun is in the proportion of five hundred to one, and by supposing light to be attracted by the same force in proportion to its mass with other bodies, all light emitted from such a body would be made to return towards it, by its own proper gravity.*

That dark star would hence not be directly observable, but if it is in a binary system, one could use the kinematics of a companion star. He even derived the corresponding radius, which corresponds to exactly the Schwarzschild radius.

A “black hole”^1^ means the observation of phenomena which are associated with matter accretion on to it, for we are not able to directly observe it electromagnetically. Emission of electromagnetic radiation, accretion discs and emerging jets are some, among many, kinds of evidence we have for the existence of such massive dark objects, lurking at the centre of galaxies.

On the other hand, spectroscopic and photometric studies of the stellar and gas dynamics in the inner regions of local spheroidal galaxies and prominent bulges suggest that nearly all galaxies harbour a central massive dark object, with a tight relationship between its mass and the mass or the velocity dispersion of the host galaxy spheroidal component (as we will see below). Nonetheless, even though we do not have any direct evidence that such massive dark objects are black holes, alternative explanations are sorely constrained (see, e.g., Kormendy 2004 and also Amaro-Seoane et al. 2010 for an exercise on constraining the properties of scalar fields with the observations in the galactic centre, although the authors conclude that one needs a mixed configuration with a black hole at the centre).

Super-massive black holes are ensconced at the centre of *active galaxies*. What we understand by *active* is a galaxy in which we can find an important amount of emitted energy which cannot be attributed to its “normal” components. These active galactic nuclei (AGNs) are powered by a compact region in their centres.

We will embark in the next sections of this review on a study of the dynamics of stellar systems harbouring a central massive object in order to extract the dominant physical processes and their parameter dependences, for instance, dynamical friction and mass segregation, as a precursor to the astrophysics of extreme mass ratio inspirals.

### Active galactic nuclei

In this section, and to motivate the introduction of the concept of massive black holes, I give a succinct introduction to active galactic nuclei, but I refer the reader to the book by Krolik (1999) on this topic.

The expression “active galactic nucleus” of a galaxy (AGN henceforth) is referring to the *energetic phenomena occurring at the central regions of galaxies which cannot be explained in terms of stars, dust or interstellar gas*. The released energy is emitted across most of the electromagnetic spectrum, UV, X-rays, as infrared, radio waves and gamma rays. Such objects have large luminosities ($10^4$ times that of a typical galaxy) coming from tiny volumes ($\ll 1\, \mathrm{pc}^3$); in the case of a typical Seyfert galaxy the luminosity is about $\sim 10^{11} ~L_{\odot }$ (where $L_{\odot } :=3.83 \cdot 10^{33}$ erg/s is the luminosity of the sun), whilst for a typical quasar it is brighter by a factor 100 or even more; actually they can emit as much as some thousand galaxies like our Milky-Way. They are, therefore, the most powerful objects in the universe. There is a connection between young galaxies and the creation of active nuclei, because the luminosity can strongly vary with the redshift.

In anticipation of something that I will elaborate on later, nowadays one explains the generation of energy as a product of matter accreting on to a super-massive black hole in the range of mass ${\mathscr {M}}_{\bullet }\sim 10^{\,6-10}\,M_{\odot }$ (where ${\mathscr {M}}_{\bullet }$ is the black hole mass). In this process, angular momentum flattens the structure of the in-falling material to a so-called *accretion disc*.

For some alternative and interesting schemes to that of MBHs, see Ginzburg and Ozernoy (1964) for spinars, Arons et al. (1975) for clusters of stellar mass BHs or neutron stars, and Terlevich (1989) for *warmers*: massive stars with strong mass-loss spend a significant amount of their He-burning phase to the left of the ZAMS on the HR diagram. The ionisation spectrum of a young cluster of massive stars will be strongly influenced by extremely hot and luminous stars.

It is frequent to observe jets, which may arise from the accretion disc, although we do not dispose of direct observations that corroborate this. Accretion is a very efficient channel for turning matter into energy. Whilst nuclear fusion reaches only a few percent, accretion can transfer almost 50% of the mass-energy of a star into energy.

Being a bit more punctilious, we should say that hallmark for AGNs is the frequency range of their electromagnetic emission, observed from $\lesssim 100$ MHz (as low frequency radio sources) to $\gtrsim 100$ MeV (which corresponds to $\sim 2 \cdot 10^{22}$ Hz gamma ray sources). Giant jets give the upper size of manifest activity $\lesssim 6~\mathrm{Mpc} \sim 2 \cdot 10^{25}$ cm,^2^ and the lower limit is given by the distance covered by light in the shortest X-ray variability times, which is $\sim 2\cdot 10^{12}$ cm.

With regard to the size, we can envisage this as a radial distance from the very centre of the AGN where, ostensibly, a supermassive black hole (SMBH) is harboured along with the different observed features of the nucleus. From the centre outwards, we have first a UV ionising source amidst the optical continuum region. This, in turn, is enclosed by the emission line clouds and the compact radio sources and these between another emitting region.

The radiated power at a certain frequency per *dex*^3^ frequency ranges from $\sim 10^{39}$ erg/s (radio power of the MW) to $\sim 10^{48}$ erg/s, the emitted UV power of the most powerful, high-redshifted quasars. Such broad frequency and radius ranges for emission causes us to duly note that they are far out of thermal equilibrium. This manifests in two ways: first, smaller regions are hotter; second, components of utterly different temperature can exist together, even though components differ by one or two orders of magnitude in size.

### Massive black holes and their possible progenitors

The quest for the source of the luminosities of $L \approx 10^{12}\, \mathrm{L}_{\odot }$ produced on such small scales, jets and other properties of quasars and other types of active galactic nuclei led in the 1960s and 1970s to thorough research that pointed to the inkling of “super-massive central objects” or “dark compact objects” (DCO) harboured at their centres.

These objects were suggested to be the main source of such characteristics Lynden-Bell (1967), Lynden-Bell and Rees (1971), Hills (1975). Lynden-Bell (1969) showed that the release of gravitational binding energy by stellar accretion on to a MBH could be the primary powerhouse of an AGN Lynden-Bell (1969). Following the same argument, 13 years later Sołtan related the quasars luminosity to the accretion rate of mass on to MBHs, so that if we use the number of observed quasars at different redshifts, we can obtain an integrated energy density Sołtan (1982). This argument strengthened the thought that MBHs are found at the centre of galaxies and acted in the past as the engines that powered ultraluminous quasars.

In the last decade, observational evidence has been accumulating that strongly suggests that MBHs are indeed present at the centre of most galaxies with a significant spheroidal component. Mostly thanks to the Hubble Space Telescope (HST), the kinematics of gas or stars in the present-day universe has been measured in the central parts of tens of nearby galaxies. In almost all cases,^4^ proper modelling of the measured motions requires the presence of a central compact dark object with a mass of a few $10^{6}$ to $10^{9}\,M_{\odot }$, see Ferrarese et al. (2001), Gebhardt et al. (2002), Pinkney et al. (2003), Kormendy (2004), Genzel et al. (2010) and references therein. Note, however, that the conclusion that such an object is indeed a MBH rather than a cluster of smaller dark objects (like neutron stars, brown dwarfs etc) has only been reached for a two galaxies. The first one is the Milky Way itself at the centre of which the case for a 3–$4\times 10^{6}\,M_{\odot }$ MBH has been clinched, mostly through ground-based IR observations of the fast orbital motions of a few stars (Ghez et al. 2005; Schödel et al. 2003 and see Genzel et al. 2010 for a review). The second case is NGC4258, which possesses a central Keplerian gaseous disc with $\mathrm {H_2O}$ MASER strong sources allowing high resolution VLBI observations down to 0.16 pc of the centre Miyoshi et al. (1995), Herrnstein et al. (1999), Moran et al. (1999).

It is, hence, largely accepted that the central dark object required to explain kinematics data in local active and non-active galaxies should be a MBH. The large number of galaxies surveyed has allowed us to study the demographics of the MBHs and, in particular, to look for correlations with properties of the host galaxy. Indeed, a deep link exists between the central MBH and its host galaxy Kormendy and Ho (2013), illuminated by the discovery of correlations between the mass of the MBH, $M_{\bullet }$, and global properties of the surrounding stellar system, e.g., the velocity dispersion $\sigma$ of the spheroid of the galaxy, known as the $M-\sigma$ relation. In spite of some progress in recent decades, many fundamental questions remain open. There is still no clear evidence of MBH feedback in galaxies, and the low mass end of the $M-\sigma$ relation is very uncertain. These facts certainly strike a close link between the formation of the galaxy and the massive object harboured at its centre.

It is also important to note that claims of detection of “intermediate-mass” black holes (IMBHs) at the centre of globular clusters raise the possibility that these correlations could extend to much smaller systems, see e.g., Gebhardt et al. (2002), Gerssen et al. (2002). The origin of these (I)MBH is still shrouded in mystery, and many aspects of their interplay with the surrounding stellar cluster remain to be elucidated.

### Tidal disruptions

The centre-most part of a galaxy, its *nucleus* consists of a cluster of a few $10^7$ to a few $10^8$ stars surrounding the DCO, assumed from now onward to be a MBH, with a size of a few pc. The nucleus is naturally expected to play a major role in the interaction between the DCO and the host galaxy, as we mentioned before. In the nucleus, stellar densities in excess of $10^6\,\mathrm{pc}^{-3}$ and relative velocities of order a few 100 to a few $1000\,\mathrm{km\,s}^{-1}$ are reached. In these exceptional conditions, unlike anywhere else in the bulk of the galaxy, collisional effects come into play. These include 2-body relaxation, i.e., mutual gravitational deflections, and genuine contact collisions between stars.

This means that, if a star happens to pass very close to the MBH, some part of it or all of it may be torn apart because of the tidal gravity of the central object. The difference in gravitational forces on points diametrically separated on the star alter its shape, from its initial approximately spherical architecture to an ellipsoidal one and, in the end, the star is disrupted. This radius can be easily calculated as follows. The star gets disrupted whenever the work exerted over it by the tidal force exceeds its own binding energy, (all energies are per unit mass). We can hence derive the radius where this happens easily. The binding energy of the star is

1 $$\begin{aligned} E_{\mathrm{bind}}=\alpha \,\frac{Gm_{\star }}{r_{\star }},~\alpha =\frac{3}{5-n}, \end{aligned}$$

**Fig. 1:**
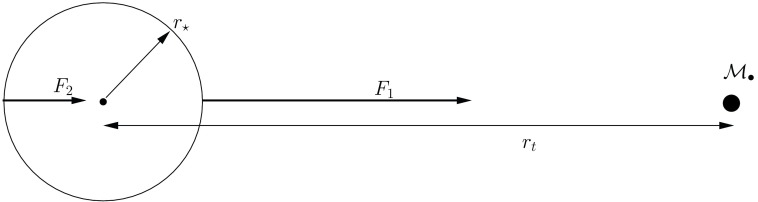
Decomposition of the tidal forces over a star. The tidal radius is $r_\mathrm{t}$, ${\mathscr {M}}_{\bullet }$ the mass of the MBH and $F_{1}$, $F_{2}$ the forces exerted on two points of the star which are diametrically separated

In the equation $r_{\star }$ and $m_{\star }$ are the radius and mass of the star, respectively, *G* the gravitational constant and *n* the polytropic index Chandrasekhar (1942) (Fig. 1).

We now equate the binding energy of the star to the work exerted over it on two points diametrically separated,

2 $$\begin{aligned} (F_{1}-F_{2})\,2r_{\star }=\alpha \frac{Gm_{\star }}{r_{\star }}, \end{aligned}$$

with

3 $$\begin{aligned} F_{1}&=\frac{G{\mathscr {M}}_{\bullet }}{(r_\mathrm{t}-r_{\star })^2},\nonumber \\ F_{2}&=\frac{G{\mathscr {M}}_{\bullet }}{(r_\mathrm{t}+r_{\star })^2}. \end{aligned}$$

Considering $r_{\star }\ll r_\mathrm{t}$, we can approximate the expressions:

4 $$\begin{aligned} \frac{1}{(r_\mathrm{t}-r_{\star })^2}&\approx \frac{1}{r_\mathrm{t}^2}+ \frac{2r_{\star }}{r_\mathrm{t}^3} \nonumber \\ \frac{1}{(r_\mathrm{t}+r_{\star })^2}&\approx \frac{1}{r_\mathrm{t}^2}- \frac{2r_{\star }}{r_\mathrm{t}^3}; \end{aligned}$$

then,

5 $$\begin{aligned} r_\mathrm{t}=\Bigg [\frac{2}{3} (5-n) \frac{{\mathscr {M}}_{\bullet }}{m_{\star }}\Bigg ]^{1/3} r_{\star }. \end{aligned}$$

For solar-type stars it is (considering a $n=3$ polytrope)

6 $$\begin{aligned} r_\mathrm{t} \simeq 1.4\times 10^{11} \left( \frac{{\mathscr {M}}_{\bullet }}{M _{\odot }} \right) ^{1/3}~\mathrm{cm}. \end{aligned}$$

**Fig. 2:**
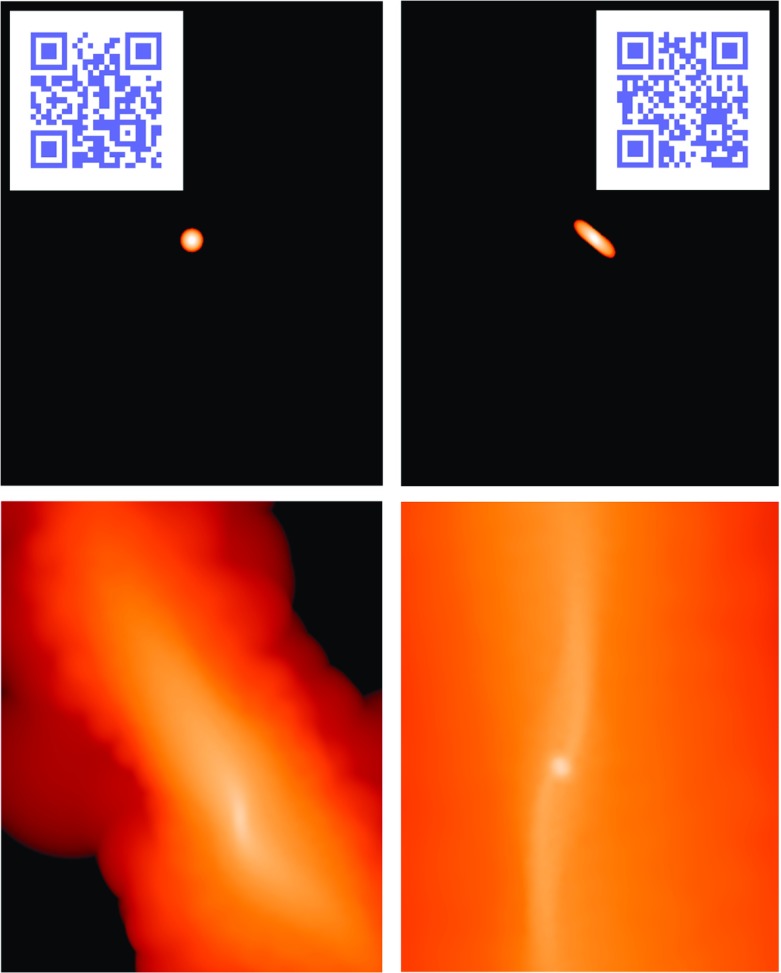
Four snapshots in the evolution of a tidal disruption of a star. In this simulation, which I have done with GADGET-2 (Springel 2005), the star is modelled as a polytrope using $5\cdot 10^4$ particles. The penetration factor, which is defined to be the ratio between the tidal radius and the distance of periapsis, has been set to 9. The mass of the MBH is $10^6\,M_{\odot }$ and of the star $1\,M_{\odot }$. The snapshots correspond to the initial time, and three later moments in the evolution. The left and right quick response codes link to two movies in the frame of the star and the general one, which point to the URLs https://youtu.be/Ryc44v4Eb7I and https://youtu.be/uZqXBD8R9Dw, respectively

In Fig. 2, I show the simulation of the tidal disruption of a star. The initial spherical architecture of the star is altered after the passage through periapsis, as we can see in the second snapshot. The third and fourth panels show the star at much later times. We can see the core of the star in the last one, idenfitied as a bright, spherical condensate of SPH particles.

**Fig. 3:**
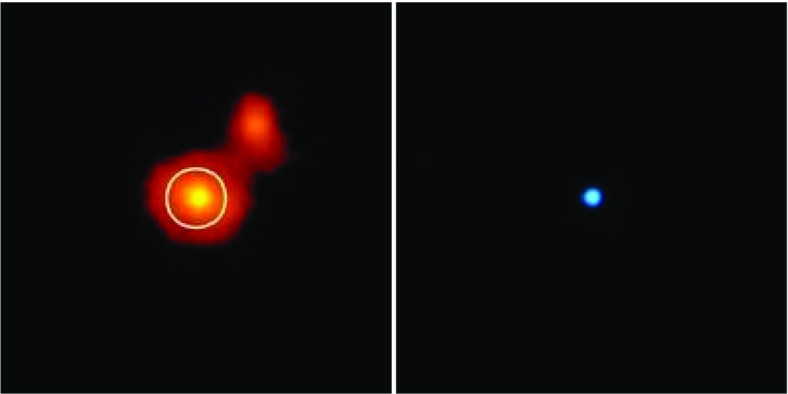
Optical and X-ray images of RX J1242-11. Credits: (left) ESO/MPE/S.Komossa and (right) NASA/CXC/MPE (Komossa et al. 2004)

Figure 3 (left) shows a Chandra X-ray image of J1242-11 with a scale of 40 arcsec on a side. This figure pinpoints one of the most extreme variability events ever detected in a galaxy. One plausible explanation for the extreme brightness of the ROSAT source could be accretion of stars on to a super-massive black hole. On the right, we have its optical companion piece, obtained with the 1.5 m Danish telescope at ESO/La Silla. The right circle indicates the position of the Chandra source in the centre of the brighter galaxy.

These processes may contribute significantly to the mass of the MBH, see e.g., Murphy et al. (1991), Freitag and Benz (2002). Tidal disruptions trigger phases of bright accretion that may reveal the presence of a MBH in an otherwise quiescent, possibly very distant, galaxy (Hills 1975; Gezari et al. 2003).

### Extreme mass ratio inspirals

On the other hand, stars can be swallowed whole if they are kicked directly through the horizon of the MBH (the so-called *direct plunges*) or gradually inspiral due to the emission of GWs The latter process, known as an “*extreme mass ratio inspiral*” (EMRI) is one of the main objects of interest for LISA, see Amaro-Seoane et al. (2017), eLISA Consortium et al. (2013), Amaro-Seoane et al. (2012a, 2013a). A compact object, such as a star so dense that it will not be disrupted by the tidal forces of the MBH, (say, a neutron star, a white dwarf or a small stellar-mass black hole), is able to approach very close to the central MBH. When the compact object comes very close to the MBH, a large amount of orbital energy is radiated away, causing the semi-major axis shrink. This phenomenon will be repeated thousand of times as the object inspirals until is swallowed by the central MBH.

The “doomed” object spends many orbits around the MBH before it is swallowed. When doing so, it radiates energy which can be conceptualised as a snapshot containing detailed information about spacetime and *all the physical parameters* that characterise the binary, the MBH and the stellar-mass black hole: their masses, spins, inclination and their sky position. The emitted GWs encode a map of the spacetime. If we can record and decode it, then we will be able to test the theory that massive dark objects are indeed Kerr black holes as the theory of general relativity predicts, and not exotic objects such as boson stars. This would be the ultimate test of general relativity.

The detection of such an EMRI will allow us to do very exciting science: EMRIs will give us measurements of the masses and spins of BHs to an accuracy which is beyond that of any other astrophysical technique. Such information will tell us about cosmic evolution, about the history and growth of MBHs in the nearby universe, with unprecedented accuracy.

The theoretical study of the structure and evolution of a stellar cluster (galactic nucleus or globular cluster) harbouring a central MBH started a few decades years ago. However, due to the complex nature of the problem which includes many physical processes and span a huge range of time and length scales, our understanding of such systems is still incomplete and, probably, subjected to revision. As in many fields of astrophysics, analytical computations can only been applied to highly idealised situations and only a very limited variety of numerical methods have been developed so far that can tackle this problem. In the next sections I will address the most relevant astrophysical phenomena for EMRIs and in the last section I give a description of a few different approaches to study these scenarios with numerical schemes.

## GWs as a probe to stellar dynamics and the cosmic growth of SMBHs

### GWs and stellar dynamics

The challenge of detection and characterisation of gravitational waves is strongly coupled with the dynamics of dense stellar systems. This is especially true in the case of the capture of a compact object by a MBH.

In order to estimate how many events one can expect and what we can assess about the distribution of parameters of the system, we need to have a very detailed comprehension of the physics. In this regard, the potential detection of GWs is an incentive to dive into a singular realm otherwise irrelevant for the global dynamics of the system.

As mentioned, a harbinger in this respect has been the tidal disruption of stars as a way to feed the central MBH. About 50% of the star is bound to the MBH and accreted on to it, producing an electromagnetic flare which tops out in the UV/X-rays, emitting a luminosity close to Eddington. Nonetheless, the complications of accretion are particularly intricate, tight on many different timescales to the microphysics of gaseous processes. Even on local, galactic accreting objects the complications of accretion are convoluted. It is thus extremely difficult to understand how to extract very detailed information about extragalactic MBHs from the flare. The question of feeding a MBH is a statistical one. We do not care about individual events to understand the growth in mass of the hole, but about the statistics of the rates on cosmological timescales. Obviously, if we tried to understand the individual processes, we would fail.

As for the fate a compact object which approaches the central MBH, this was never addressed before we had the incentive of direct detection of gravitational radiation. Astrophysical objects such as a black hole binary, generate perturbations in space and time that spread like ripples on a pond. Such ripples, known as “gravitational waves” or “gravitational radiation”, travel at the speed of light, outward from their source. These gravitational waves are predicted by general relativity, first proposed by Einstein. Measurement of these gravitational waves give astrophysicists a totally new and different way of studying the Universe: instead of analysing the propagation and transformation of particles such as photons, we have direct information from the fabric of spacetime itself. The information carried by the gravitational radiation will tell us in exquisite detail about the history, behaviour and structure of the universe: from the Big Bang to black holes.

When we started to look into this problem, we realised that there were many questions of stellar dynamics that either did not have an answer or that had not even been addressed at all. In this review I will discuss the relaxation processes that we know to play a major role in the dynamics of this particular regime. This involves two-body as well as many-body-coherent or non-coherent relaxation, and relativity. The list of processes is most likely incomplete, for there can still be additional, even more complicated processes unknown to us. We now have more questions than answers.

### The mystery of the growth of MBHs

**Fig. 4:**
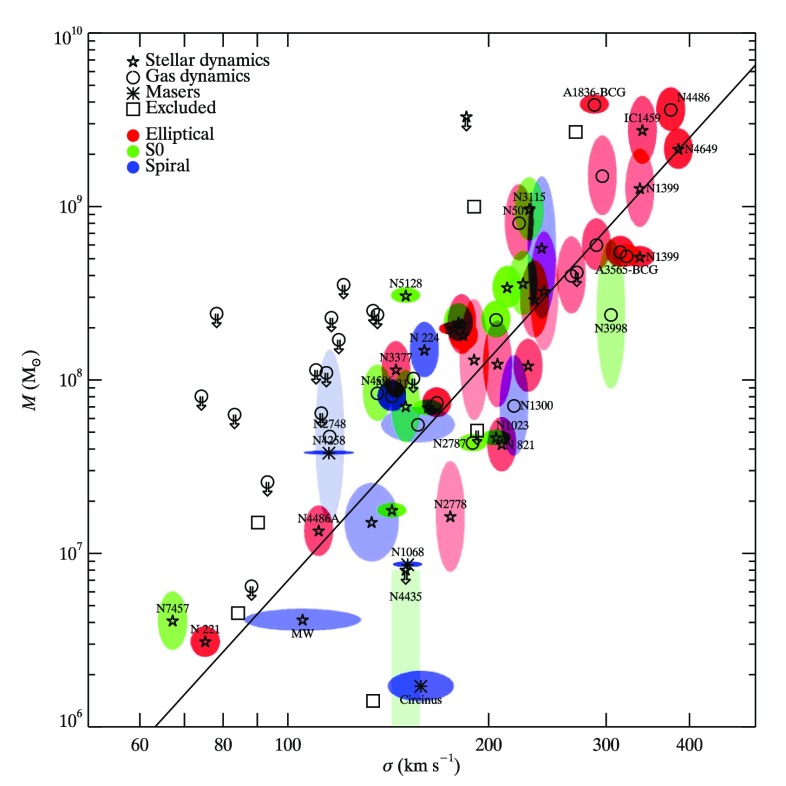
Correlation between the mass of supermassive black holes and the velocity dispersion of their host galaxies. Image reproduced with permission from Gültekin et al. (2009), copyright by AAS

One of the most exciting results of modern astronomy is the discovery, mostly through high-resolution observations of the kinematics of stars and gas, that most, if not all, nearby bright galaxies harbour a dark, massive, compact object at their centre, see Ferrarese and Ford (2005), Kormendy (2004), Gültekin et al. (2009), from which we reproduce their figure in Fig. 4, and Kormendy and Ho (2013). The most spectacular case is our own galaxy, the Milky Way, see Genzel et al. (2010) for a review. By tracking and interpreting the stellar dynamics at the centre of our galaxy, we have the best evidence for the existence of a massive dark object, very probably a MBH.

The close examination of the Keplerian orbits of the so-called “S-stars” (also called S0-stars, where the letter S stands simply for source) has revealed the nature of the central dark object located at the Galactic Centre. By following one of them, S2 (S02), the mass enclosed by the orbit, a volume with radius no larger than 6.25 light-hours, was estimated to be about $3.7\times 10^6\,M_{\odot }$ Schödel et al. (2003), Ghez et al. (2003). More recent data based on many years of observations set the mass of the central MBHs to $\sim 4 \times 10^{6} \, M_{\odot }$.

Observations of other galaxies indicate that the masses of SMBH can reach a few billion solar masses ($M_{\odot }$), they correlate tightly with the stellar properties of the host galaxies (e.g., the velocity dispersion $\sigma$ of galaxy bulge). The existence of such a SMBH population in the present-day universe is strongly supported by Sołtan’s argument that the average mass density of these SMBHs agrees with expectations from integrated luminosity of quasars Sołtan (1982), Yu and Tremaine (2002). Claims of detection of “intermediate-mass” black holes (IMBHs, with masses ranging between $100-10^4\,M_{\odot }$) at the centre of globular clusters Gebhardt et al. (2002), Gerssen et al. (2002) raise the possibility that these correlations extend to much smaller systems, but so far the strongest, although not conclusive, observational support for the existence of IMBHs are ultra-luminous X-ray sources Miller and Colbert (2004), Kong et al. (2010).

Although there is an emerging consensus regarding the growth of large-mass MBHs thanks to Sołtan’s argument, MBHs with masses up to $10^7\,M_{\odot }$, such as our own MBH in the Galactic Centre (with a mass of $\sim 4\times 10^6\,M_{\odot }$), are enigmatic. There are many different explains of their masses: accretion of multiple stars from arbitrary directions, see Phinney (1989), Magorrian and Tremaine (1999), Syer and Ulmer (1999), Hills (1975), Rees (1988), mergers of compact objects such as stellar-mass black holes and neutron stars, see Quinlan and Shapiro (1990), or IMBHs falling on to the MBH, Portegies Zwart et al. (2006). Other more peculiar means are accretion of dark matter Ostriker (2000) or collapse of supermassive stars Hara (1978), Shapiro and Teukolsky (1979), Rees (1984), Begelman (2010). The origin of these low-mass MBHs and, therefore, the early growth of *all* MBHs, remains a conundrum.

The centre-most part of a galaxy, its *nucleus*, consists of a nuclear star cluster of a few millions of stars surrounding the MBH, see Schödel et al. (2014). The nucleus is naturally expected to play a major role in the interaction between the MBH and the host galaxy. In the nucleus, stellar densities in excess of a million stars per cubic parsec and relative velocities of the order $\sim$ 100–1000 $\mathrm{km\,s}^{-1}$can be reached. In these conditions, as mentioned before, collisional effects are important come into play. This is true except in globular clusters, but one important difference is that the SMBH gives the central part of the cluster almost a Keplerian potential, and thus very tricky resonance characteristics. This is one reason it has been difficult to analyse the stars here.

### A magnifying glass

The laser interferometer space antenna (LISA), see in particular the document prepared in response to the call for missions for the L3 slot in the Cosmic Vision Programme, Amaro-Seoane et al. (2017), but also Danzmann (2000), Amaro-Seoane et al. (2012a, 2013a), will be our reference point throughout my review. LISA consists of three spacecraft arranged in an equilateral triangle with sides of length 2.5 million kilometre. LISA will scan the entire sky and covers a band from below $10^{-4}\,$Hz to above $10^{-1}\,$Hz. In this frequency band, the Universe is populated by strong sources of GWs such as binaries of supermassive black holes merging in the centre of galaxies, massive black holes “swallowing” entirely small compact objects like stellar-mass black holes, neutron stars and white dwarfs. The information is encoded in the gravitational waves: the history of galaxies and black holes, the physics of dense matter and stellar remnants like stellar-mass black holes, as well as general relativity and the behaviour of space and time itself. Chinese mission study options, such as Taiji, Bender et al. (2005a), Gong et al. (2011, 2015), Huang et al. (2017) will also be able to catch these systems with good signal-to-noise ratios.

**Fig. 5:**
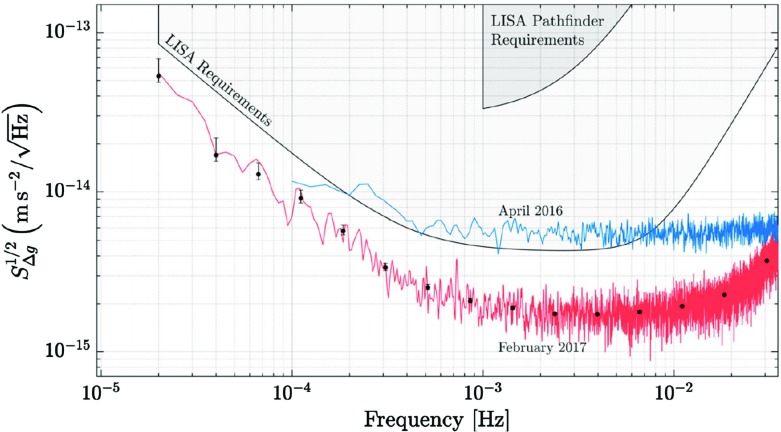
Amplitude spectral density of LISA Pathfinder as compared to the previous publication of the LPF group (Armano et al. 2016), which is the curve in blue. The data are compare with LISA requirements, as presented in Amaro-Seoane et al. (2017). We can see that LISA Pathfinder exceeds the requirements for key technologies for LISA over a factor of two over the entire observation band. Image reproduced with permission from Armano et al. (2018), copyright by the authors

In any case, a key property of GW astrophysics is the fact that GWs interact only very weakly with matter, except for high-z. The observations we will make with LISA will not suffer any of the usual problems in astrophysics—absorption, scattering, or obscuration. This is what makes LISA-like missions such as LISA or Taiji unique. It is not “merely” a test of general relativity; these missions would be able to corroborate the underlying theory of the nature of the central dark objects which we now observe in most galaxies. We will get direct information from the heart of the densest stellar systems in the Universe: galactic nuclei, nuclear stellar clusters and globular clusters. The LISA mission technology has been successfully tested with the LISA Pathfinder^5^ mission, an ESA-led mission with a contribution from NASA, launched in 2015 from Kourou, French Guiana. Figure 5 is reproduced from Armano et al. (2018). This publication has remarkably improved the previous results of Armano et al. (2016), which showed that LISA Pathfinder has satisfied the mission requirements by factors ranging from 10 to 1000 depending on the frequency range, achieving a sub-Femto-*g* in free fall (Armano et al. 2016). Indeed, the results published in 2018 show that, actually, LISA Pathfinder has exceeded the requirements for LISA by more than a factor of two over the whole observation band (down to 20 $\upmu$Hz).

For the full success of a mission such as LISA, it is important that we *understand* the systems that we expect to observe. A deep theoretical comprehension of the sources which will populate LISA’s field of view is important to achieve its main goals.

Whilst main-sequence stars are tidally disrupted when approaching the central MBH, compact objects (stellar-mass black holes, neutron stars, and white dwarfs) slowly spiral into the MBH and are swallowed whole after some $\sim 10^5$ orbits in the LISA band. At the closest approach to the MBH, the system emits a burst of GWs which contains information about spacetime and the masses and spins of the system. We can envisage each such burst as a snapshot of the system. This is what makes EMRIs so appealing: a set of $\sim 10^5$ bursts of GWs radiated by *one* system will tell us with the utmost accuracy about the system itself, it will test general relativity, it will tell us about the distribution of dark objects in galactic nuclei and globular clusters and, thus, we will have a new understanding of the physics of the process. New phenomena, unknown and unanticipated, are likely to be discovered.

If the central MBH has a mass larger than $10^7\,M_{\odot }$, then the signal of an inspiraling stellar-mass black hole, even in its last stable orbit (LSO) will have a frequency too low for detection. On the other hand, if it is less massive than $10^4\,M_{\odot }$, the signal will also be quite weak unless the source is very close. This is why one usually assumes that the mass range of MBHs of interest in the search of EMRIs for LISA is between $[10^7,\,10^4]\,M_{\odot }$. Nonetheless, if the MBH is rotating rapidly, then even if it has a mass larger than $10^7\,M_{\odot }$, the LSO will be closer to the MBH and thus, even at a higher frequency, the system should be detectable. This would push the total mass to a few $\sim 10^7\,M_{\odot }$.

For a binary of a MBH and a stellar-mass black hole to be in the LISA band, it has to have a frequency of between roughly $10^{-5}$ and 1 Hz. The emission of GWs is more efficient as they approach the LSO, so that LISA will detect the sources when they are close to the LSO. The total mass required to observe systems with frequencies between 0.1 Hz and $10^{-4}$ is of $10^4$–$10^7\,M_{\odot }$. For masses larger than $10^7\,M_{\odot }$, the frequencies close to the LSO will be too low, so that their detection will be very difficult. On the other hand, for a total mass of less than $10^3\,M_{\odot }$ we could in principal detect them at an early stage, but then the amplitude of the GW would be rather low.

On top of this, the measurement of the emitted GWs will give us very detailed information about the spin of the central MBH. With current techniques, we can only hope to measure MBH spin through X-ray observations of Fe K$\alpha$ profiles, but the numerous uncertainties of this technique may disguise the real value. Moreover, such observations can only rarely be made.

This means that LISA will scrutinise exactly the range of masses fundamental to the understanding of the origin and growth of supermassive black holes. By extracting the information encoded in the GWs of this scenario, we can determine the redshifted mass and spin of the central MBH with an astonishing relative precision. Additionally, the mass of the compact object which falls into the MBH and the eccentricity of the orbit will be recovered from the gravitational radiation with a tiny fractional accuracy. All this means that LISA will not be “just” the ultimate test of general relativity, but an exquisite probe of the spins and range of masses of interest for theoretical and observational astrophysics and cosmology.

#### A problem of $\sim$ 10 orders of magnitude

For the particular problem of how does a compact object end up being an extreme mass ratio inspiral, we have to study very different astrophysical regimes, spanning over many orders of magnitude.

*Galactic or cosmological dynamics*

**Fig. 6:**
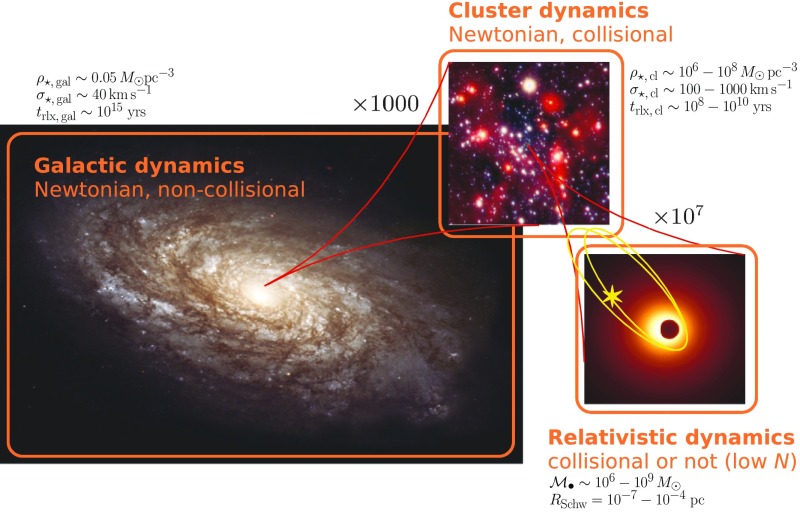
On the left, and with the largest scale, the galaxy has an average density of stars of about $0.05\,M_{\odot }\mathrm{pc}^{-3}$. The velocity dispersion is $\sim 40\,\mathrm{km\,s}^{-1}$. From these quantities one can infer that the relaxation time in the vicinity of our Sun is $t_\mathrm{rlx} \sim 10^{15}$ yrs. The upper panel shows the galactic nucleus that such a galaxy has. A typical size for it is $\sim \,1$ pc, the stellar density ranges between $10^6 -10^8\,M_{\odot }\,\mathrm{pc}^{-3}$ and the velocity dispersion is of $\sigma \sim 100 - 1000\,\mathrm{km\,s}^{-1}$. In this region, $t_\mathrm{rlx} \sim 10^{8} - 10^{10}$ yrs. In the last panel, we have that the dynamics of the system is dominated by General Relativity. As a reference point, the Schwarzschild radius of a $10^6\,M_{\odot }$ ($10^9\,M_{\odot }$) is $10^{-7}$ pc ($10^{-4}$ pc)

Figure 6 depicts the three different realms of stellar dynamics of relevance for the problem of EMRIs. At the largest scale exists the galaxy, with a size of a few kiloparsecs. Just as a point of reference, the gravitational radius of a MBH of $10^6\,M_{\odot } \sim 5\cdot 10^{-8}$ pc. The relaxation time, $t_\mathrm{rlx}$ which I will introduce with more detail ahead, is a timescale which can be envisaged as the required time for the stars to exchange energy and angular momentum between them: it is the time that the stars need to “see” each other individually and not only the average, background stellar potential of the whole stellar system. For the galaxy, $t_\mathrm{rlx}$ is larger than the Hubble time, which means that, on average, it has no influence on the galaxy at all. A test star will only feel the mean potential of the rest of the stars and it will never exchange either energy or angular momentum with any other star. The system is “collisionless”, meaning that two-body interactions can be neglected. This defines the realm of stellar galactic dynamics, the one investigated in Cosmological simulations using, e.g., *N*-body integrators. Since we do not have to take into account the strong interactions between stars, one can easily simulate ten billion particles with these integrators.

*Cluster dynamics*

If we zoom in by typically a factor of $10^3$, we enter the (mostly Newtonian) stellar dynamics of galactic nuclei. There, $t_\mathrm{rlx} \sim 10^8{-}10^{10}$ yrs. In this realm stars do feel the graininess of the stellar potential. The closer we get to the central MBH, the higher $\sigma$ will be, if the system is in centrifugal equilibrium; the stars have to orbit around the MBH faster. In particular, S2 (or S02), one of the S-stars (S0-stars) for which we have enough data to reconstruct the orbit to a very high level of confidence—as we saw in the previous section—has been observed to move with a velocity of $15\cdot 10^{3}\,\mathrm{km\,s}^{-1}$. Typically, $t_\mathrm{rlx}$ is (on occasion *much*) shorter than the age of the system, of a few $\sim 10^{8} - 10^{10}$ yrs. For these kind of systems one has to take into account relaxation, exchange of energy and angular momentum between stars. The system is “collisional”. When we have to take into account this in the numerical simulations, the result is that we cannot simulate with *N*-body integrators more than some thousands of stars on a regular computer. To get to more realistic particle numbers one has to resort to many computers operating in parallel, special-purpose hardware or the graphic processor units. I will discuss this later.

*Relativistic stellar dynamics*

Last, in the right panel of Fig. 6, we have the relativistic regime of stellar dynamics when we enlarge the previous by a factor of ten million. There the role of relativistic effects is of paramount importance for the evolution of the system. In this zone, generally, *there are no stars*. Even at the densities which characterise a galactic nucleus, the probability of having a star in such a tiny volume is extremely small. Moreover, even if we had a significantly larger volume, or a much higher density for the galactic nucleus, so that we had a few stars close to the MBH, these would quickly merge with the MBH due to the emission of GWs, which is what defines an EMRI. But they do it *too* fast. These systems can be collisional or collisionless, depending on how many stars we have at a given time. If they are there, they will exchange energy and angular momentum between them. Nevertheless, relaxation is not well-defined in this regime.

The key point here is how to replenish that area, so that there are other stars replacing those which merge quickly with the central MBH. On average, there are *zero* stars. As a matter of fact, and in general, for the general study of the stellar dynamics of galactic nuclei, the role of this last realm is negligible. One does not have to bother with the effects of GR; most, if not all, stars are on a Newtonian regime. The impact on the dynamics of galactic nuclei is zero. It is impressive that this last region dominated by the effects of GR has an effect worth studying at all. But, as we will see ahead, the encoded information that one can recover from the detection of an EMRI about its surrounding dynamical system is dramatic. If we want to address this problem, we need to cope with a range of scales that spans over seven orders of magnitude when understanding the role of the dynamics of galactic nuclei in relativistic dynamics, and of ten orders of magnitude in the big picture.

### How stars distribute around MBHs in galactic nuclei

**Fig. 7:**
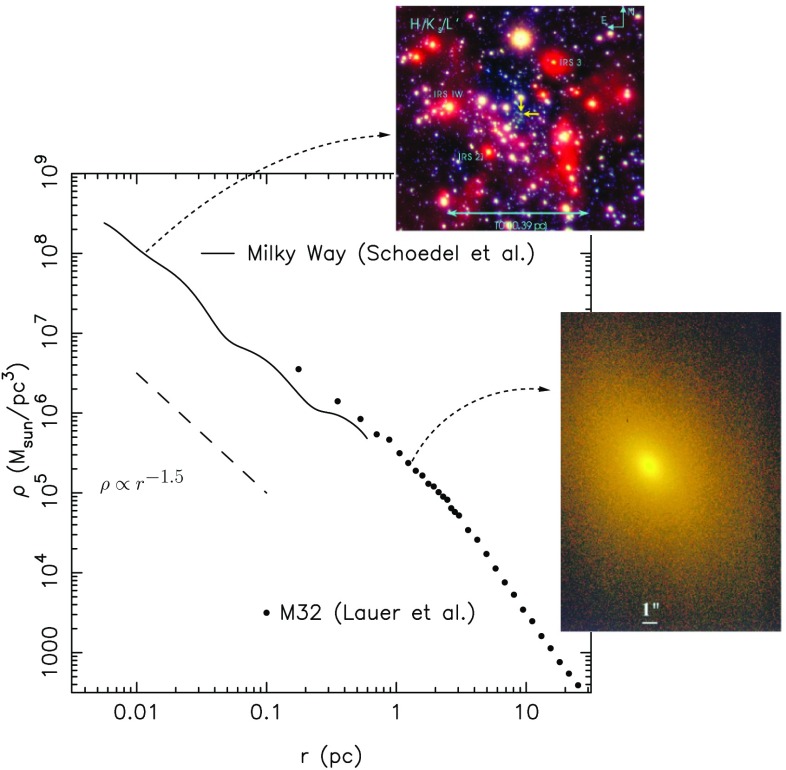
Density profile for the GC of the MW and for M32, both with MBHs of masses $3\times 10^6\,M_{\odot }$ and influence radii $\sim 3$ pc. The dashed curve on the very left corresponds to a slope of $\rho \propto r^{-3/2}$. Image adapted from Merritt (2006), Schödel et al. (2002), Lauer et al. (1998)

In Fig. 7 I show data constrained by electromagnetic measurements. One of the very first questions one has to address when trying to understand the stellar dynamics around a MBH is *how many stars are there and how do they distribute around it*? Unfortunately there are very few observations for this because we are interested in nuclei that harbour lower-mass MBHs, i.e., with masses ranging $10^4$ and $10^6\,M_{\odot }$, so that they therefore have a small radius of influence $r_\mathrm{inf}$ and, thus, they are observationally very difficult to resolve. Currently there are only a very few galaxies that are both in the range of GW frequencies interesting to us and that have a resolved $r_\mathrm{inf}$. For these we have information on how bound stars that can become EMRIs are distributed around the central MBH. Obviously, the Milky Way (MW) is one of these galaxies. In Fig. 7 the stellar density profile of the MW is displayed. We see that it goes up to at least $10^8\,M_{\odot }/\mathrm{pc}^3$ in the inner regions. This number has been calculated by assuming a population of stars; one has to deproject the observation, because we are only seeing a few of the total amount of stars, the brightest ones. One assumes that the observed stars are tracing an underlying population invisible to us. This requires a considerable amount of modelling to obtain the final results. These are uncertain by, at most, a factor of ten. In the same figure we have another nucleus, M32, which should be harbouring a MBH with a mass similar to the one located in the GC. The density profile happens to be similar to the one corresponding to the GC. Whether this is a coincidence or something deeper is not clear. In any case, and to *first order of approximation*, we can state that once we know the mass of the MBH, we know the way stars distribute around it. Later the relevance of this point will be obvious to the reader.

**Fig. 8:**
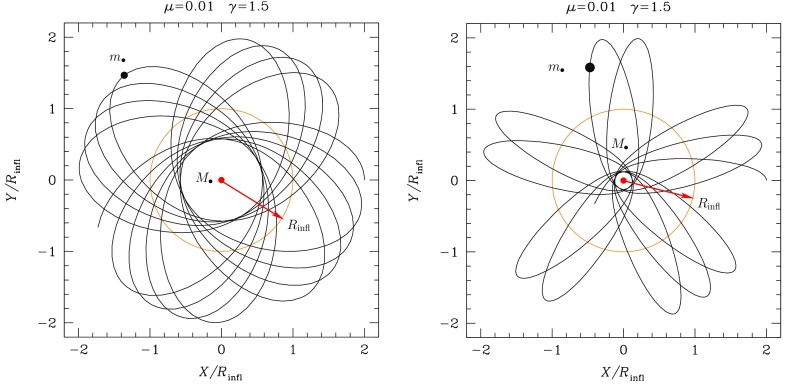
Projection in the X–Y plane of the evolution of two test star orbits in a stellar system without relaxation. The central, orange point represents the position of the MBH, the black dots on the orbits the position of the test stars and the red arrow delimits the influence radius $R_\mathrm{infl}$ of the MBH. The right panel represents a case with a larger eccentricity. The orbits extend further than the $R_\mathrm{infl}$

## A taxonomy of orbits in galactic nuclei

Before we address the physics and event rate estimates of EMRIs, it is crucial that we have a good understanding of the kind of orbits that we might expect in the environments natural to COs in dense systems around a MBH. An important factor in understanding how a star can become an EMRI is the shape and evolution of its orbit. In this section, I will address these two aspects. First, we will *not* take into account the role of relaxation. The stellar potential in which our test star $m_{\bullet }$ is moving is completely smooth. For any purpose, the test star will not feel any individual star, but a background potential.

### Spherical potentials

Consider now Fig. 8; there we have two orbits which differ in their eccentricity. The rosettes are characterised by their energy and angular momentum. Since the test stars do not suffer any individual gravitational tug from the stellar system (at least not on a noticeable timescale), the orbital elements are kept constant. The periapsis^6^ is fixed because the angular momentum is conserved, so that the test star will never come arbitrarily close to the central MBH. In order to achieve anything interesting, one needs to perturbate the system.

**Fig. 9:**
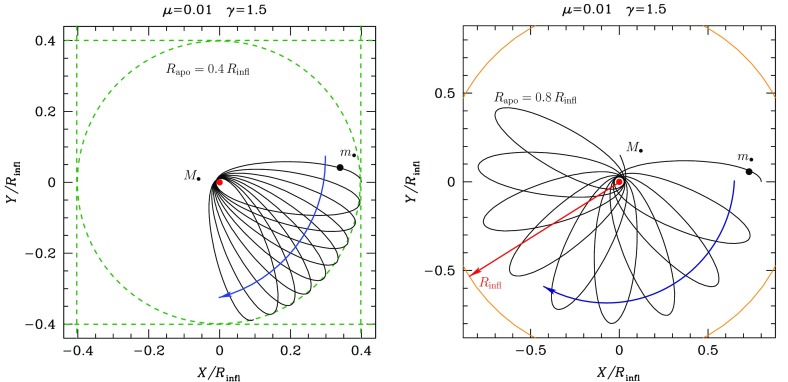
Same as Fig. 8 for an apoapsis $R_\mathrm{apo}=0.4,\,0.8\,R_\mathrm{infl}$ and a velocity of the CO of $0.2\,V_\mathrm{circ}$, with $V_\mathrm{circ}$ the circular velocity

A different situation, however, is when the orbit of the test star is *within* the $R_\mathrm{infl}$ of the MBH. In this case, the orbits look more and more like Keplerian ellipses, unless one gets *very* close to the central MBH, so that we get relativistic precession. In Fig. 9 we have an ellipse which precesses with time. This is neither the relativistic precession nor an advance, but a purely Newtonian perihelion (periapsis) retard, counterclockwise. The timescale for it is

7 $$\begin{aligned} T_\mathrm{New,\,PS} \approx \frac{{\mathscr {M}}_{\bullet }}{M_{\star }(a)}\,P_\mathrm{orb} \approx \frac{R_\mathrm{infl}}{a}P_\mathrm{orb} \end{aligned}$$

In this last equation, $M_{\star }(a)$ is the amount of stellar mass encompassed within the orbit. The Newtonian periapsis retard is the result of the fact that we do not have a perfect Keplerian orbit because we do not have a point mass, but an *extended* mass distribution. As an exercise, we can compare the last equation to the relativistic periapsis *advance* (in order of magnitude),

8 $$\begin{aligned} T_\mathrm{Rel,\,PS} \approx \frac{R_\mathrm{peri}}{R_\mathrm{Schw}}\,P_\mathrm{orb} \end{aligned}$$

This equation is only relevant for orbits whose periapsis is very small, whilst the later one is only important for relatively extended orbits (because $M_{\star }(a)$ is larger)

### Non-spherical potentials

**Fig. 10:**
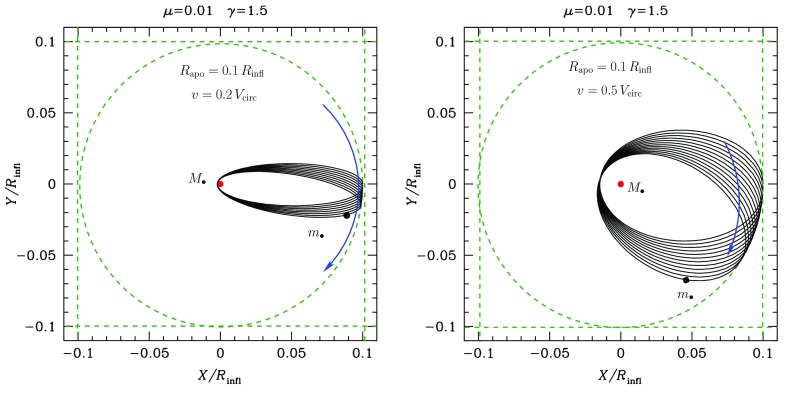
Same as Figs. 8 and 9 for different values of the apoapsis radius and velocity of the CO

**Fig. 11:**
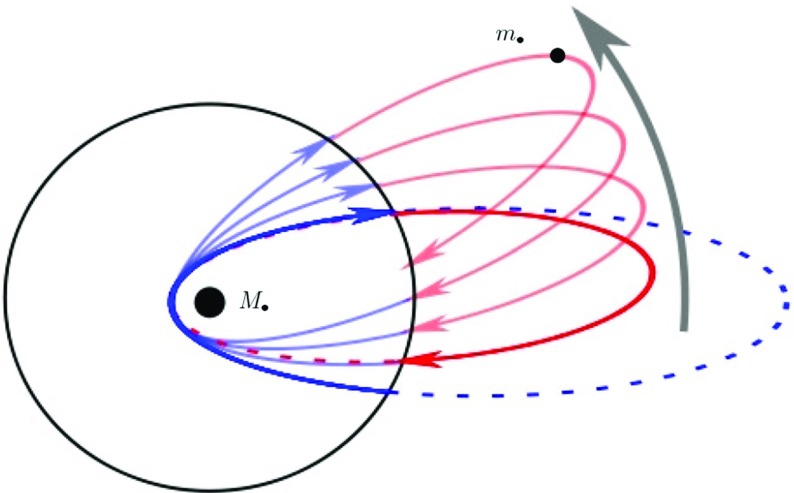
In the Newtonian case we have an extended mass distribution, so that the star feels more mass far away than closer to the centre. When the star traverses the “sphere”, the trajectory abruptly changes and becomes a smaller ellipse Thus, the object goes back to the centre faster; the orbit precesses in the opposite direction to the orbital one In the relativistic case the kinetic energy of the star increases its gravitational mass when it’s close to the centre: The effective attraction is more efficient and the trajectory is more curved towards the centre

The most general case is the triaxial potential, in which we still have symmetry but neither spherical nor axial-symmetry, it is a general ellipsoidal configuration. The angular momentum has no component conserved. This, obviously, allows orbits to get “as close as they want” to the centre. Not *all* orbits will, but there are specific families of orbits which, if one waits long enough, will get arbitrarily close to the centre. This is evidently very relevant for our study. These orbits are refered to as *centrophilic* orbits for clear reasons. Studies of models of triaxial galaxies have found that there is a significant fraction of such orbits even very close to the central MBH. At distances as short as $r<R_\mathrm{infl}$, within the sphere of influence, some models have as many as 20% of stars that are on centrophilic orbits. One should nevertheless bear in mind that these are *models*, not corroborated by direct observations of galaxies. They depend on a number of set-up parameters which will result in strong fluctuations of the final result: the true number could be between 0 and 20% according to these models. Therefore, unfortunately we do not know what the real implications are for observed nuclei, since it is not well-constrained. Of course, one can resort to (non-collisional) *N*-body simulations to study the merger of two galaxies to see in the resulting product how many of these orbits one can get (Figs. 10, 11).

**Fig. 12:**
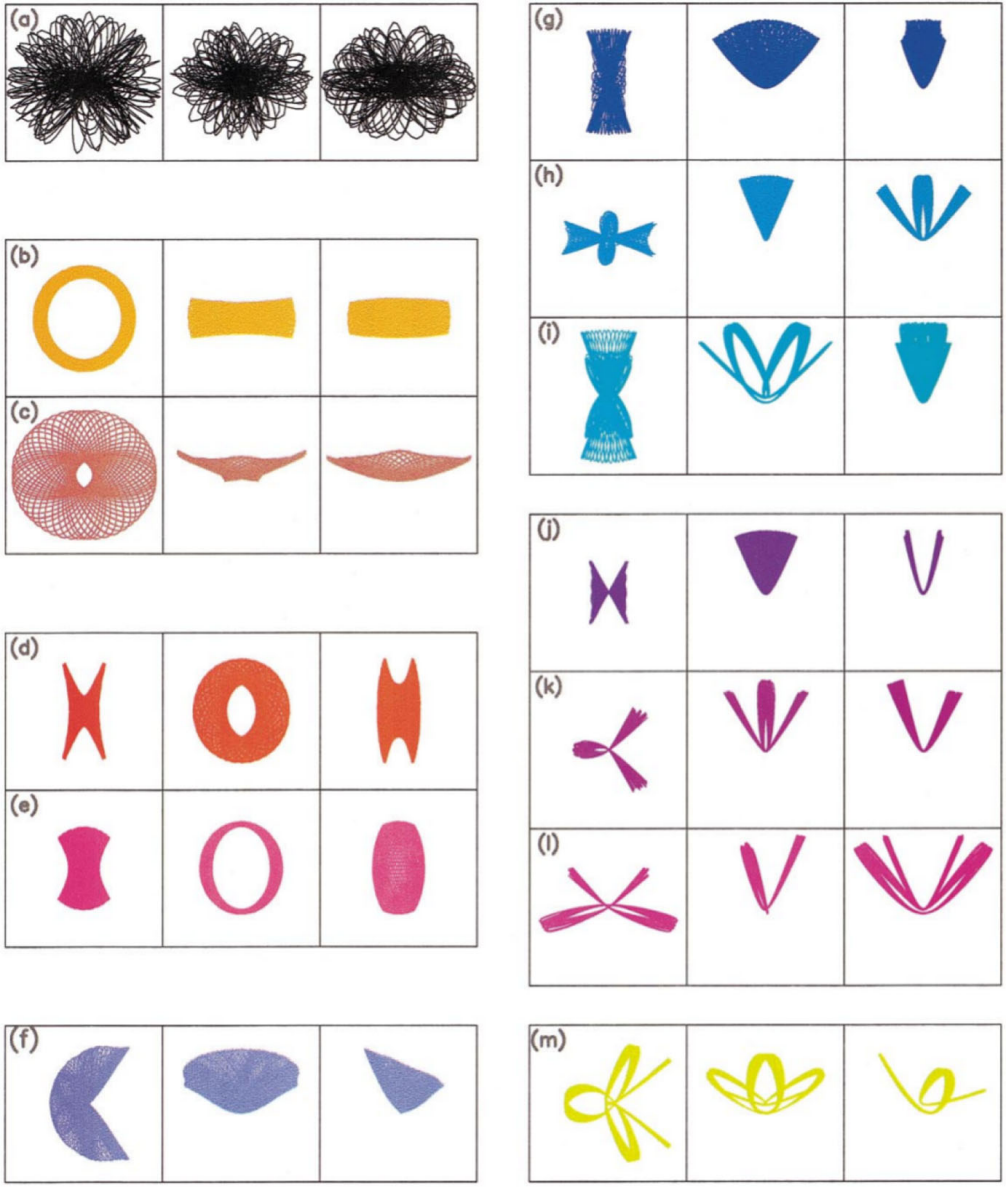
From the left to the right and from the top to the bottom, we have stochastic orbits, short-axis tube orbit, saucer orbit, a resonant short-axis tube, inner long-axis tube, long-axis tube, resonant, pyramid, resonant pyramid, resonant pyramid orbit, banana orbit, 2 : 3 : 4 resonant banana, 3 : 4 : 6 resonant banana, and a 6 : 7 : 8 resonant orbit. We note that the projections of “tube”, centrophilic stellar orbits around a MBH in triaxial nuclei will look aligned in one or another plane depending on the potential. Image reproduced with permission from Poon and Merritt (2001), copyright by AAS

As for the implications of the detection rates of EMRIs, this could have a huge impact, but the problem should probably be revisited due to the enormous difficulties that force us to make broad simplifications. For instance, we should explore the behaviour of the potential *very* close to the MBH because, by definition, at some point the potential is completely dominated by the MBH and, thus, spherically symmetric. The only realistic hope here are those stars that typically are on orbits with semi-major axis much larger than the radii of interest to us, so that even if they spend most of the time very far away from the MBH, they will be set on a centrophilic orbit due to the triaxiality of the system, but it is unclear whether these can contribute significantly to the local density around the MBH. As an example of the kind of orbits one can get in a triaxial galactic nucleus, in Fig. 12 I show some representative examples of *centrophobic* orbits from Poon and Merritt (2001) (cases b, c, d, e). This means that the stars never reach the centre. The lack of conservation of the angular momentum can set stars on either centrophilic orbits or, alternatively, on centrophobic orbits. These can be envisaged as a generalisation of rosette orbits. Nevertheless, since we are interested in EMRIs, we will focus on centrophilic orbits and leave the further description of centrophobic orbits aside. I refer the interested reader to the work by Holley-Bockelmann et al. (2001, 2002), and also to the more recent one by Merritt and Vasiliev (2011).

We have two different kinds of centrophilic orbits: (i) pyramid or box orbits. These are still regular but a star on such an orbit can reach arbitrarily small distances in its periapsis; (ii) stochastic orbits, which also come arbitrarily close to the centre. The probability for an orbit to get within a distance *d* from the central MBH, the very centre of the potential, is proportional to *d*.

This is non-intuitive. If you have a target with a mass and you shoot a projectile from random directions, the probability of coming within a distance *d* of the target $R_\mathrm{p}<d$ is proportional to *d* itself and not $d^2$ (which would have been the case for a totally random experiment, without focusing). In the case of a star on an orbit towards the MBH, the number of times you have to “throw” it to get to a periapsis distance closer than *d* is, $N_\mathrm{pass}\,(R_\mathrm{p}<d) \propto d$. The reason for this is that our target is a particular one and influences the projectile through a process called gravitational focusing. The projectile, the star, is attracted by the target, the MBH.

Something to also bear in mind is that all of these simulations are limited by a particular resolution, which is still far from being close to reality, so that we are not in the position of extrapolating these results to the distances where the star will be captured by the MBH and become and EMRI.

## Two-body relaxation in galactic nuclei

### Introduction

We are now back to a spherical system world, in which orbits such as those in the previous section do not exist. Therefore, one needs an additional factor to bring stars close to the MBH. As I have already discussed before, a possibility, is to have a source of exchange of energy and angular momentum. We use and abuse the term collisional to refer to any effect not present in a smooth, static potential, including secular effects. Among these, standard two-body relaxation excells not due to its relevance of contributing to EMRI sources, but due to the fact that this is the best-studied effect; namely the exchange of energy and angular momentum between stars due to gravitational interactions.

Another possibility is physical collisions.^7^ The stars come so close to each other that they collide, they have a hydrodynamic interaction; the outcome depends on a number of factors, but the stars involved in the collision could either merge with each other or destroy each other completely or partially. Contrary to what one could expect, the impact of these processes for the global evolution of the dynamics of galactic nuclei is negligible Freitag and Benz (2002). In most of the cases, when these extended stars, such as main-sequence stars (MS) collide, they do not merge due to the very high velocity dispersion, and they will also not be totally destroyed, because for that they would need a nearly head-on collision, so that they have a partial mass-loss and are for our purposes uninteresting. For the kind of objects of interest to us in this review, stellar-mass black holes, the probability that they physically collide is negligible.

A third way of altering the angular momentum of stars are secular effects. They do nevertheless *not* modify the energy. If we assume that the orbits around the MBH are nearly Keplerian, the shape, an ellipse, does not change, and the orientation will not change much. If we have another orbit with a different orientation, both orbits will exert a torque $\mathcal T$ on each other. This will change angular momentum but not energy. A Keplerian orbit can be described in terms of its semi-major axis and eccentricity. The semi-major axis is only connected to energy and, for a given semi-major axis the eccentricity is connected to the angular momentum. If one changes the angular momentum but not the energy, the eccentricity will vary but not the semi-major axis. By decreasing the angular momentum, one increases the eccentricity.

In this section, however, I introduce the fundamentals of relaxation theory, focusing on the aspects that will be more relevant for the main interest of this review. Further ahead, in Sect. 7, I will address resonant relaxation and other “exotic” (in the sense that they are not part of the traditional two-body relaxation theory) processes. For a comprehensive discussion on two-body relaxation, I recommend the textbooks by Spitzer Jr (1969) and Binney and Tremaine (2008) or, for a shorter but very nice introduction, the article by Freitag and Benz (2001).

I will first introduce handy timescales in Sect. 4.2 that will allow us to pinpoint the relevant physical phenomena that reign the process of bringing stars (extended or compact) close to the central MBH. I will then address a particular case of relaxation, in Sect. 4.3, dynamical friction. Later, in Sect. 4.4, I will define more concisely the region of space-phase in which we expect stars to interact with the central MBH. Once we have all these concepts, we can cope with the problem of how mass segregates in galactic nuclei, in Sect. 5. We will first see in detail the “classical” although academic solution, and later a more recent and physical result, the so-called strong mass segregation, in Sect. 7.3

### Two-body relaxation

I introduce now some useful time-scales to which I will refer often throughout this review; namely the relaxation time, the crossing time and the dynamical time. These three time-scales allow us to delimit our physical system.

*The relaxation time*

In Chandrasekhar (1942) a time-scale was defined which stems from the 2-body small-angle encounters and gives us a typical time for the evolution of a stellar system.

This relaxation time could be regarded as an analogy of the shock time of the gas dynamics theory, by telling us when a particle (a star) has forgotten its initial conditions or, expressed in a different way, when the local thermodynamical equilibrium has been reached. Then, we can roughly say that the most general idea is that this is the time over which the star “forgets” its initial orbit due to the series of gravitational tugs caused by the passing-by stars. After a relaxation time the system has lost all information about the initial orbits of all the stars. This means that the encounters alter the star orbit from the one it would have followed if the distribution of matter was smooth. Hence, we can regard *the relaxation time* as the time interval required for the velocity distribution to reach the Maxwell–Boltzmann form.

**Fig. 13:**
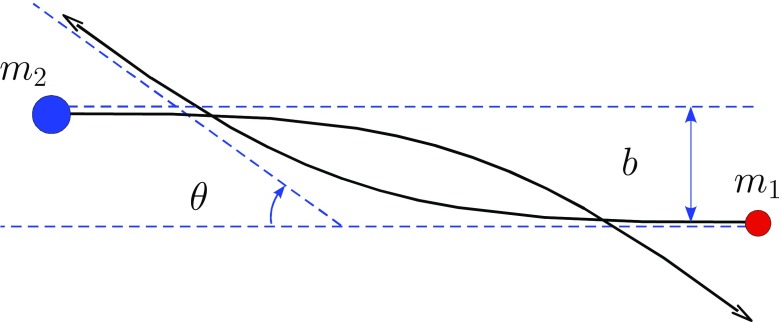
Deflection angle $\theta$ of a “test” star of mass $m_1$ with a field star of mass $m_2$

Consider two stars of masses $m_1$ and $m_2$ deflecting each other as in Fig. 13. The deflection angle $\theta$ is given by the relation

9 $$\begin{aligned} \tan \frac{\theta }{2}=\frac{b_0}{b},~\mathrm{with}~b_0=\frac{G(m_1+m_2)}{v_\mathrm{rel}^2} \end{aligned}$$

**Fig. 14:**
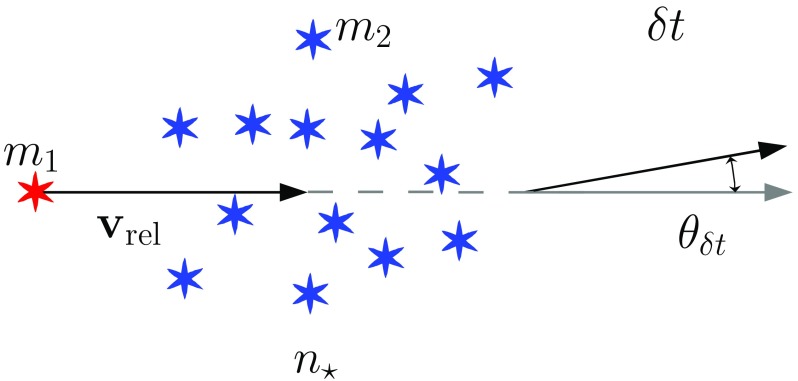
The test stars suffers a change in direction by $\theta _{\delta t}$ due to the accumulation of encounters with field stars

If the relative velocity $v_\mathrm{rel}$ is high, $\theta$ is small and the larger the mass, the stronger the deflection. This simple relation expresses the kernel of relaxation. One has to integrate it over all possible parameters to get the relaxation rate. When we do the integration over the impact parameter *b* whilst keeping $v_\mathrm{rel}$ and the masses fixed, we have the picture of Fig. 14. The test star encounters a lot of field stars, all of them with the same mass $m_2$ and relative velocity $\mathbf{v}_\mathrm{rel}$. After a time $\delta t$, the velocity vector of the test star has slightly changed direction by an angle $\theta _{\delta t}$. On average, $\langle \theta _{\delta t}\rangle =0$ but

10 $$\begin{aligned} \langle \theta _{\delta t}^2\rangle =\left( \frac{\pi }{2}\right) ^2\,\frac{\delta t}{\hat{t}_\mathrm{rlx}} \end{aligned}$$

Therefore, it is a *diffusion* process; $\langle \theta _{\delta t}^2\rangle \propto \delta t$, see e.g., Spitzer and Hart (1971), Hénon (1975). I have introduced the special relaxation time for this situation as

11 $$\begin{aligned} \hat{t}_\mathrm{rlx} = \frac{\pi }{32}\frac{v_\mathrm{rel}^3}{\ln \varLambda \,G^2\,n_{\star }(m_1+m_2)^2} \end{aligned}$$

In this last equation, $\ln \varLambda$, the Coulomb logarithm, has appeared as a result of integrating for all impact parameters. The information encoded in it is how many orders of magnitude of *b* contribute to the relaxation,

12 $$\begin{aligned} \ln \varLambda =\ln \,\frac{b_{\max }}{b_0}\simeq \ln \,\frac{P_\mathrm{orb}}{b_0/v_\mathrm{rel}} \end{aligned}$$

In this last equation $b_0$, which I introduced before, is the effective minimum impact parameter for relaxation. Our main focus is not a detailed review of stellar dynamics. For a detailed description of the Coulomb logarithm, I refer the reader to Binney and Tremaine (2008), Spitzer Jr (1987). Therefore, I will simply comment that, for our purposes, $\ln \varLambda \approx 10{-}15$ always. This is very useful because the exact calculation can be rather arduous and almost an incubus which to our knowledge nobody has attempted to implement in any calculation. Therefore we mention only two special cases for the argument of the logarithm,

13 $$\begin{aligned} \varLambda \approx \left\{ \begin{array}{ll} 0.01\,N_{\star } &{} \text {(a) for a self-gravitating}\\ &{} \text {stellar cluster}\\ {\mathscr {M}}_{\bullet }/m &{} \text {(b) close to the MBH} \end{array} \right. \end{aligned}$$

In case (a), we have a self-gravitating cluster of stars in equilibrium with itself but lacking a central MBH. The argument is proportional to the number of stars in the system. In the situation in which a star is orbiting the MBH, the previous value is formally no longer valid and one should use the value (b). Nevertheless, in effect this is neglected because the value turns out to be $\sim 10$. To define a local average value of the relaxation time we integrate over the distribution of relative velocities.

It must, nevertheless, be noted that the way in which I have introduced the concept of the relaxation time is a particular one. In Eq. (11), I have introduced the “encounter relaxation time” to stress that it depends on the characteristics of a peculiar class of encounter: a star of mass $m_1$ with “field stars” of mass $m_2$ with a local density $n_{\star }$ and a relative velocity $v_\mathrm{rel}$. It can be envisaged as the required time to deflect gradually the motion of star $m_1$ due to encounters with field stars by a root mean square (RMS) angle $\pi /2$. This definition is useful to understand the fundamentals of relaxation, but it must be noted that it is subject to this very peculiar type of encounter.

However, in a general case, we define relaxation by simplifying the problem: (i) We restrict to the radius of influence for a system in which the distribution of stars is spherically symmetric, (ii) stars are treated as single objects, with a two-body relaxation as the only mechanism that can change the angular momentum, and (iii) we neglect mass segregation.

The influence radius within which the central MBH dominates the gravitational field is

14 $$\begin{aligned} r_\mathrm{infl}=\frac{G{\mathscr {M}}_{\bullet }}{\sigma _0^2}\approx 1~\mathrm{pc}\left( \frac{{\mathscr {M}}_{\bullet }}{10^6\,M_\odot }\right) \left( \frac{60~\mathrm{km/s}}{\sigma _0}\right) ^2. \end{aligned}$$

Hence, in our approximation, the relaxation time is

15 $$\begin{aligned} t_\mathrm{rlx}(r)= & {} \frac{0.339}{\ln \varLambda }\frac{\sigma ^3(r)}{G^2\langle m\rangle m_\mathrm{CO}n(r)}\nonumber \\&\simeq 1.8\times 10^8\,\mathrm{yr}\left( \frac{\sigma }{100\,\mathrm{km\,s}^{-1}}\right) ^3 \left( \frac{10\,M_{\odot }}{m_\mathrm{CO}}\right) \left( \frac{10^6\,M_{\odot }\mathrm{pc}^{-3}}{\langle m\rangle n}\right) . \end{aligned}$$

Here, $\sigma (r)$ is the local velocity dispersion. It is approximately equal to the Keplerian orbital speed $\sqrt{G{\mathscr {M}}_{\bullet }r^{-1}}$ for $r<r_\mathrm{infl}$ and has a value $\approx \sigma _0$ outside of it. In the expression *n*(*r*) is the local number density of stars, $\langle m\rangle$ is the average stellar mass, $m_\mathrm{CO}$ is the mass of the compact object (we take a standard $m_\mathrm{CO}=10\,M_\odot$ for stellar-mass black holes).

For typical density profiles, $t_\mathrm{rlx}$ decreases slowly with decreasing *r* inside $r_\mathrm{infl}$. It should be noted that the exchange of energy between stars of different masses—sometimes referred to as dynamical friction, as we will see ahead, in Sect. 4.3 in the case of one or a few massive bodies in a field of much lighter objects—occurs on a timescale shorter than $t_\mathrm{rlx}$ by a factor of roughly $M/\langle m\rangle$, where *M* is the mass of a heavy body.

As we will see later, relaxation redistributes orbital energy amongst stellar-mass objects until the most massive of them (presumably stellar-mass black holes) form a power-law density cusp around the MBH, $n(r)\propto r^{-\gamma }$ with $\gamma$ ranging between $\simeq$ 1.75–2.1, which depends on the solution to mass segregation considered, while less massive species arrange themselves into a shallower profile, with $\alpha \simeq 1.4{-}1.5$ (Bahcall and Wolf 1976; Lightman and Shapiro 1977; Duncan and Shapiro 1983; Freitag and Benz 2002; Amaro-Seoane et al. 2004; Baumgardt et al. 2004a; Preto et al. 2004; Freitag et al. 2006b; Hopman and Alexander 2006a; Alexander and Hopman 2009; Merritt 2010; Preto and Amaro-Seoane 2010; Amaro-Seoane and Preto 2011; see also Sect. 8.7). Nuclei likely to host MBHs in the LISA mass range (${\mathscr {M}}_{\bullet }\lesssim \text{ few }\times 10^6\,M_{\odot }$) probably have relaxation times comparable to or less than a Hubble time, so that it is expected that their heavier stars form a steep cusp.

*Collision time*

**Fig. 15:**
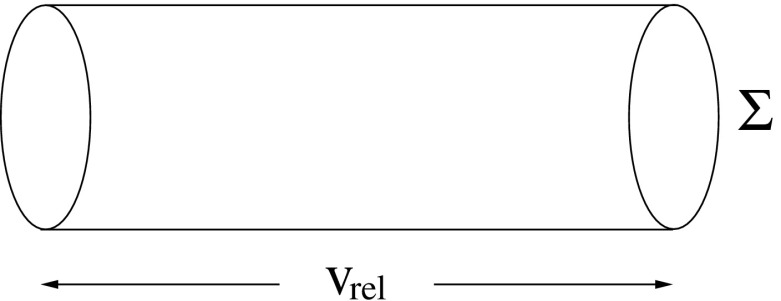
Definition of the collision time

${t_\mathrm{coll}}$ is defined as the required mean time for the number of stars within a volume $V=\varSigma v_\mathrm{rel} \triangle t$ to be one (see Fig. 15), where $v_\mathrm{rel}$ is the relative velocity at infinity of two colliding stars.

Computed for an average distance of closest approach $\bar{r}_{\min }= \frac{2}{3}r_{\star }$, this time is given by

16 $$\begin{aligned} n_{\star }V(t_\mathrm{coll})=1= n_{\star }\,\varSigma \, v_\mathrm{rel}\, t_\mathrm{coll}. \end{aligned}$$

And so,

17 $$\begin{aligned} t_\mathrm{coll}=\frac{m_{\star }}{\rho _{\star }\varSigma \sigma _\mathrm{rel}}, \end{aligned}$$

where

18 $$\begin{aligned} \varSigma =\pi \bar{r}_{\min }^2 \left( 1+ \frac{2Gm_{\star }}{\bar{r}_{\min }\sigma _\mathrm{rel}^2}\right) , \end{aligned}$$

$\sigma _\mathrm{rel}^2=2\sigma _{\star }^2$ is the stellar velocity dispersion and $\varSigma$ a collisional cross-section with gravitational focusing.

*The crossing time*

As the name suggests, this is the required time for a star to pass through the system, i.e., to *cross* it. Obviously, this value is given by the ratio between space and velocity,

19 $$\begin{aligned} t_\mathrm{cross}= \frac{R}{v}, \end{aligned}$$

where *R* is the size of the physical system and *v* the velocity of the star crossing it.

For instance, in a star cluster it would be:

20 $$\begin{aligned} t_\mathrm{cross}= \frac{r_\mathrm{h}}{\sigma _\mathrm{h}}; \end{aligned}$$

where $r_\mathrm{h}$ is the radius containing 50% of the total mass and $\sigma _\mathrm{h}$ is a typical velocity taken at $r_\mathrm{h}$. One denominates it *velocity dispersion* and is introduced by the statistical concept of RMS dispersion; the *variance* $\sigma ^2$ gives us a measure of the dispersion, or scatter, of the measurements within the statistical population, which in our case is the star sample:

$$\begin{aligned} \sigma ^2= \frac{1}{N} \sum _{i=1}^{N} (x_{i}- \mu _{a})^2. \end{aligned}$$

In the last expression, $x_{i}$ are the individual stellar velocities and $\mu _{a}$ is the arithmetic mean,

$$\begin{aligned} \mu _{a} \equiv \frac{1}{N} \sum _{i=1}^{N}x_{i}. \end{aligned}$$

If virial equilibrium prevails, we have $\sigma _\mathrm{h} \approx \sqrt{GM_\mathrm{h}/r_\mathrm{h}}$, then we get the dynamical time-scale

21 $$\begin{aligned} t_\mathrm{dyn} \approx \sqrt{\frac{r_\mathrm{h}^3}{GM_\mathrm{h}}}\approx \frac{1}{\sqrt{G\rho _{\star }}}, \end{aligned}$$

where $\rho _{\star }$ is the mean stellar density.

Contrary to gas dynamics, the thermodynamical equilibrium time-scale $t_\mathrm{rlx}$ in a stellar system is large compared with the crossing time $t_\mathrm{cross}$. In a homogeneous, infinite stellar system, we expect some kind of stationary state to be established in the limit $t\rightarrow \infty$. The decisive feature for such a virial equilibrium is how quickly a perturbation of the system will be smoothed down.

The dynamical time in virial equilibrium is (cf., e.g., Spitzer Jr 1987):

22 $$\begin{aligned} t_\mathrm{dyn}\propto \frac{\log (\gamma N)}{N} t_\mathrm{rlx} \ll t_\mathrm{rlx}. \end{aligned}$$

If we have perturbations in the system because of the heat conduction, star accretion on to the MBH, etc. a new virial equilibrium will be established within a time $t_\mathrm{dyn}$, which is short. This means that we get again a virial-type equilibrium in a short time. This situation can be considered not far from a virial-type equilibrium. We say that the system *changes in a quasi-stationary way*.

### Dynamical friction

Consider now a star more massive than the average. In this case, relaxation boils down to dynamical friction (DF). The massive intruder will suffer from dynamical friction, which is an effect of all encounters with lighter stars. For this special kind of star, the timescale over which its orbital parameters change is not the relaxation time. This star will lose kinetic energy in the following timescale:

23 $$\begin{aligned} t_\mathrm{DF} \sim \frac{\langle m \rangle }{m}\,t_\mathrm{rlx}. \end{aligned}$$

As we can see, if the object is 10–20 times more massive than the average, as in the case of a stellar-mass black hole, this timescale is correspondigly 10–20 times shorter than the $t_\mathrm{rlx}$.

**Fig. 16:**
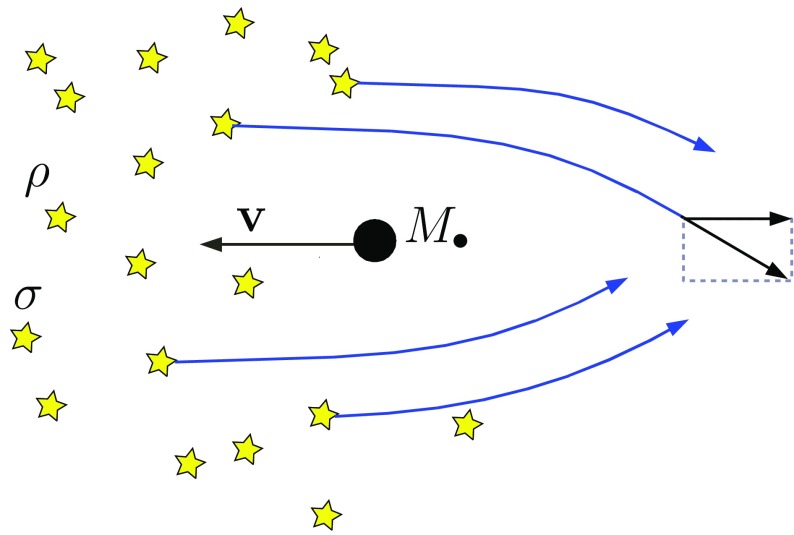
In the reference frame of the encounter I depict a massive interloper, a stellar-mass black hole, traversing a sea of lighter stars which are deflected by it. The velocity vector of the stellar-mass black hole is bearely modified (at least in direction) by the deflections, because they cancel out on average

**Fig. 17:**
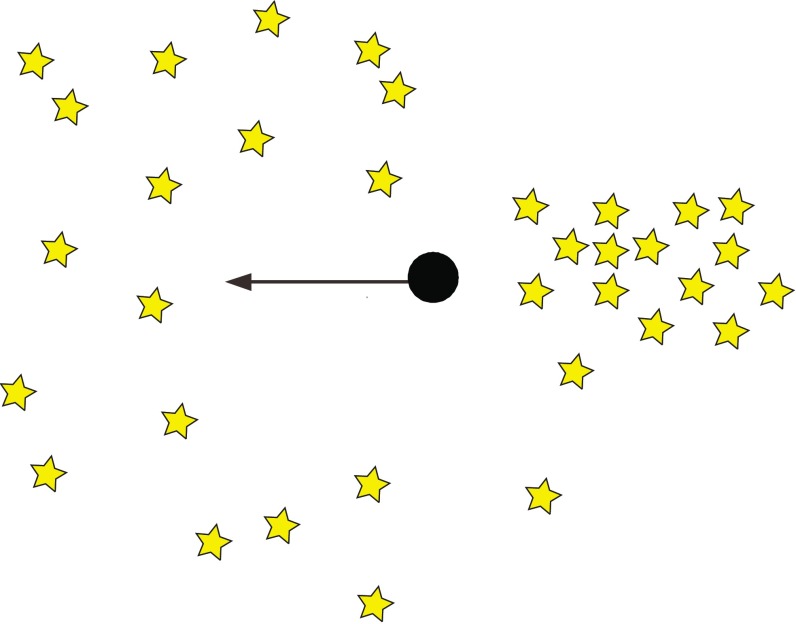
The accumulation of stars right behind the massive perturber creates a region of stellar overdensity that acts on the perturber, slowing it down, braking it

In Fig. 16 we have an illustration for what DF is. A massive intruder, a stellar-mass black hole, is travelling in a homogeneous sea of stars of density $\rho$ and velocity dispersion $\sigma$. The velocity vectors of the stars is rotated after the deflection and the projected component in the direction of the deflection is shorter. Therefore, the massive object is accumulating just after it a high-density stellar region. The perturber will feel a drag from that region from the conservation of angular momentum in the direction of its velocity vector, just as depicted in Fig. 17. The direction does not change to first-order, but the amplitude decreases. The intruder will feel a force (acceleration) given by the Chandrasekhar formula,

24 $$\begin{aligned} \mathbf {a}_\mathrm{DF} = -\frac{\mathbf {v}}{t_\mathrm{DF}} -\frac{4\pi \ln \varLambda \,G^2\rho \,M}{v^3}\,\xi (X)\mathbf {v}. \end{aligned}$$

In this last equation,

25 $$\begin{aligned} \xi (X)&= \mathrm{erf}(X)-2\pi ^{-1/2}Xe^{-X^2},\nonumber \\ X&= \frac{v}{\sqrt{2}\sigma } \end{aligned}$$

The most interesting point is that if we plug into Eq. 24 the velocity of the perturber which is $v \approx \sigma$, we have that

26 $$\begin{aligned} t_\mathrm{DF} \sim \frac{m}{M}t_\mathrm{rlx} \ll t_\mathrm{rlx} \end{aligned}$$

**Fig. 18:**
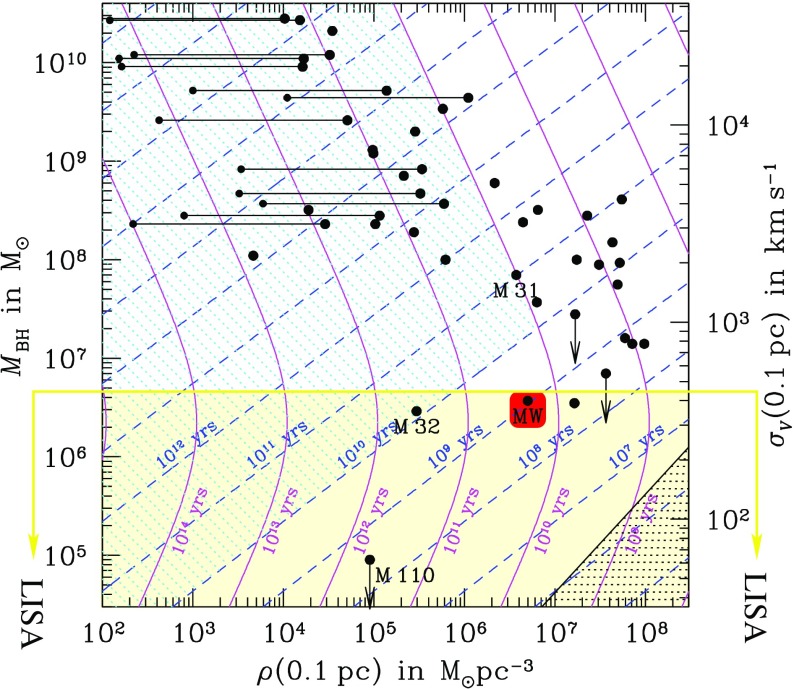
Plane of the stellar density at 0.1 pc and the mass of the central MBH. Relaxation (and collision times) at 0.1 pc from an MBH in the centre of a galactic nucleus. Image adapted from Freitag and Benz (2005)

As I have already mentioned before, galactic nuclei in the range of what a mission like LISA could observe have relaxation times that are shorter than a Hubble time. In Fig. 18, which is a modified version of the one to be found in Freitag and Benz (2005), we have a schematic representation of what relaxation times in other observed galaxies could be. Each dot shows the mass of the central MBH or the upper limit to it (the arrows). From this mass we can derive what the velocity dispersion would be at 0.1 pc, and from observations of the brightness surface profiles we can estimate what the stellar density at that distance would be. In many cases this distance is usually not resolvable, so that one has to extrapolate in order to obtain the density at 0.1 pc, which is what has been done in Fig. 18. The blue, dashed lines correspond to $t_\mathrm{rlx}(r=0.1\,\mathrm{pc})$, the relaxation time at that distance. Any system below $10^{10}$ yrs should be relaxed and is, hence, interesting. For the range of frequencies we are interested in, MBHs with masses typically less than a few $10^7\,M_{\odot }$ (the region below the red line) we can see only three (since M110 is only an upper limit and M33 possibly lacks an MBH). This low number does not mean that nuclei in the range of frequencies of interest are rare, it simply means that it is hard to observe MBHs in that range of masses. In this regard, a GW mission that could observe MBHs in that region would provide us with very valuable information, since in the electromagnetic domain we are still far from resolving those nuclei.

### The difussion and loss-cone angles

As we have seen, the relaxation time is the required time to induce a change in the perpendicular velocity component of the same order as the perpendicular velocity component itself, i.e., $\triangle v_{\perp }^2 /v_{\perp }^2 \simeq 1$. Therefore,

27 $$\begin{aligned} \triangle v_{\perp }^2 = n_\mathrm{rlx} \cdot \delta v_{\perp }^2. \end{aligned}$$

Hence,

28 $$\begin{aligned} \triangle v_{\perp }^2 / v_{\perp }^2 = 1 = \frac{n_\mathrm{rlx} \cdot \delta v_{\perp }^2}{ v_{\perp }^2}. \end{aligned}$$

And then,

29 $$\begin{aligned} t_\mathrm{rlx}=n_\mathrm{rlx} \cdot t_\mathrm{dyn} = \left( \frac{v_{\perp }^2}{\delta v_{\perp }^2} \right) \cdot t_\mathrm{dyn}, \end{aligned}$$

where $n_\mathrm{rlx}$ is the numbers of crossings for $\triangle v_{\perp }^2 / v_{\perp }^2 \simeq 1$. This conforms to the definition of the relaxation time, $\triangle v_{\perp }^2 / v_{\perp }^2 = t/t_\mathrm{rlx}$, see Binney and Tremaine (1987).

A useful quantity to derive is the diffusion angle, $\theta _\mathrm{D}$, which is defined to be the mean deviation of a star orbit in a dynamical time, i.e., $t_\mathrm{rlx} \simeq t_\mathrm{dyn}/{\theta _\mathrm{D}^2}$. I assume that this angle must be a very small one, so that

30 $$\begin{aligned} \sin {\theta _\mathrm{D}} \simeq \frac{ \delta v_{\perp }}{v} \simeq \theta _\mathrm{D}. \end{aligned}$$

Therefore,

31 $$\begin{aligned} \theta _\mathrm{D} \simeq \sqrt{\frac{t_\mathrm{dyn}}{t_\mathrm{rlx}}}. \end{aligned}$$

I now introduce the loss-cone angle $\theta _\mathrm{lc}$ as an illustrative example. Suppose that the central object with mass ${\mathscr {M}}_{\bullet }$ has an influence radius $r_\mathrm{h}$. To define this radius we say that a star will interact with the central object only when $r \le r_\mathrm{h}$. Then, we look for a condition at a place $r>r_\mathrm{h}$ for a star to touch or to cross the influence radius of the central object within a crossing time $t_\mathrm{cross}= r/ \sigma _\mathrm{r}$.

**Fig. 19:**
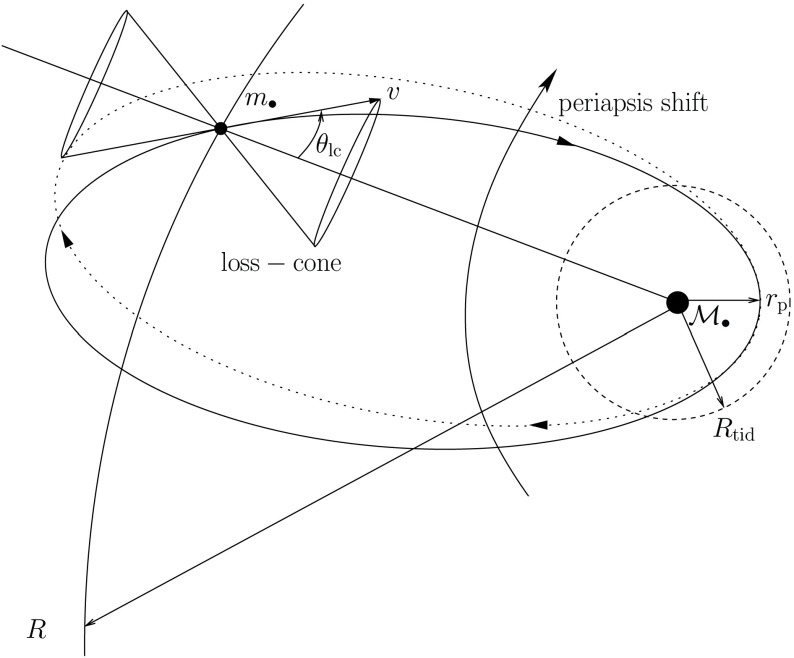
Definition of the loss-cone angle $\theta _\mathrm{lc}$. The star has a mass $m_{\bullet }$, the MBH a mass $\mathscr {M}_{\bullet }$, $r_\mathrm{p}$ is the periapsis distance, $R_\mathrm{tid}$ the tidal radius and *R* the distance to the MBH

I depict this in Fig. 19: A star on a certain orbit will get into the tidal disruption radius of the MBH if its velocity vector is such that the distance of periapsis is within that radius. The velocity and radial distance vectors define the angle of the cone in phase-space for this to happen. Extended stars are torn apart and lost for the system, which is why we refer to that angle as the loss-cone angle. If the star is a stellar-mass black hole, it can withstand the tidal forces. Although I have also illustrated the effect of periapsis shift in the figure, I do not take it into account for the derivation of the loss-cone. It is meant to illustrate the complexity of the problem we are interested in, the gravitational capture of compact objects. As we have seen before, the condition that defines this angle is the following:

32 $$\begin{aligned} r_\mathrm{p}(E,L)&\le r_\mathrm{t},\nonumber \\ \theta&\le \theta _\mathrm{lc}, \end{aligned}$$

33 $$\begin{aligned} \sin {\theta }&= \frac{v_\mathrm{t}}{v},~\mathrm{with}~\theta \ll 1 \nonumber \\ \theta&\simeq \frac{v_\mathrm{t}}{v}= \frac{L/r}{v}. \end{aligned}$$

In the last expression I have introduced $L:=r\,v_\mathrm{t}$ as the specific angular momentum. Now, I derive an expression for this angle in terms of the influence radius. Within the region $r \le r_\mathrm{h}$, the star moves under the MBH potential influence, then

34 $$\begin{aligned} \sigma (r)&\approx \sqrt{ \frac{G{\mathscr {M}}_{\bullet }}{r}} = \sqrt{ \frac{G{\mathscr {M}}_{\bullet }}{R_\mathrm{h}}} \sqrt{ \frac{R_\mathrm{h}}{r}} \nonumber \\&= \sigma (R_\mathrm{h}) \sqrt{R_\mathrm{h}/r} = \sigma _{c} \sqrt{R_\mathrm{h}/r}, \end{aligned}$$

since $\sigma _{c}^2 \equiv G{\mathscr {M}}_{\bullet } / R_\mathrm{h}.$ The typical velocity of the orbit is $\langle v^2 \rangle \simeq 3 \sigma ^3$, where the factor three accounts for the three directions in the space. Since $\sigma$ means the one-dimensional dispersion, we have to take into account the dispersion of the velocity in each direction. Then,

35 $$\begin{aligned} \langle v \rangle \simeq \sqrt{3} \sigma _{c} \sqrt{r_\mathrm{h}/r}. \end{aligned}$$

Finally, we obtain the loss-cone angle,

36 $$\begin{aligned} \theta _\mathrm{lc} = \sqrt{ \frac{2}{3} \frac{r_\mathrm{t}}{r}}. \end{aligned}$$

In the region in which $r \ge r_\mathrm{h}$ we can consider that the velocity dispersion is more or less constant from this $r_\mathrm{h}$ onwards, $v \approx \sqrt{3} \sigma _{c}$,

37 $$\begin{aligned} \theta _\mathrm{lc}&= \frac{ \sqrt{2G{\mathscr {M}}_{\bullet }r_\mathrm{t}}}{ \sqrt{3} r \sigma _{c}};\nonumber \\ \sigma _{c}&= \sqrt{G{\mathscr {M}}_{\bullet }/r_\mathrm{h}}. \end{aligned}$$

The angle is

38 $$\begin{aligned} \theta _\mathrm{lc} \approx \frac{1}{r}\,\sqrt{\frac{2\,r_\mathrm{t}\, r_\mathrm{h}}{3}} \end{aligned}$$

**Fig. 20:**
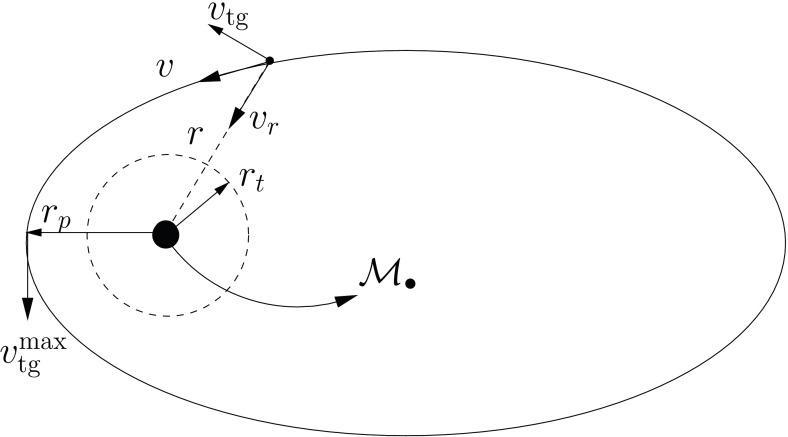
Definition of the tidal radius “$r_\mathrm{t}$”, and depiction of the distance of closest approximation of the star in its orbit to the MBH. In this point the radial component of the velocity of the star cancels and the tangential component is maximum. In the figure “$r_\mathrm{p}$” stands for the periapsis radius

I have derived the loss-cone velocity $v_\mathrm{lc}(r)$ using angular momentum and energy conservation arguments. We just have to evaluate it at a general radius *r* and at the tidal radius $r_\mathrm{t}$, where the tangential velocity is maximal and the radial velocity cancels (see Fig. 20).

For a general radius we have that

39 $$\begin{aligned} E(r)=&\phi (r) - \frac{v_\mathrm{tg}(r)^2}{2} - \frac{v_\mathrm{r}(r)^2}{2} \nonumber \\ L(r)=&r v_{tg}(r) \end{aligned}$$

For the tidal radius:

40 $$\begin{aligned} E(r_\mathrm{t})=&\phi (r_\mathrm{t}) - \frac{v_{tg}(r_\mathrm{t})^2}{2} \nonumber \\ L(r_\mathrm{t})=&r_\mathrm{t} v_{tg}(r_\mathrm{t}), \end{aligned}$$

Hence, from momentum conservation and the fact that $v_\mathrm{r}(r_\mathrm{t})=0$, we get

41 $$\begin{aligned} v_\mathrm{tg}(r_\mathrm{t})= \frac{r}{r_\mathrm{t}} v_\mathrm{tg}(r). \end{aligned}$$

Using energy conservation and the last result,

42 $$\begin{aligned}&\phi (r) - \frac{v_{tg}(r)^2}{2} - \frac{v_\mathrm{r}(r)^2}{2} \nonumber \\&\quad =\phi (r_\mathrm{t}) - \frac{r^2}{2r_\mathrm{t}^2} v_{tg}(r)^2. \end{aligned}$$

Then we get the tangential velocity of the stars in terms of r; namely, the loss-cone velocity:

43 $$\begin{aligned} v_\mathrm{lc}(r) =&\frac{r_\mathrm{t}}{\sqrt{r^2-r_\mathrm{t}^2}} \nonumber \\&\times \sqrt{2[ \phi (r_\mathrm{t}) - \phi (r)] + v_\mathrm{r}(r)^2}. \end{aligned}$$

The angular momentum is

44 $$\begin{aligned} L(r_\mathrm{t})=&r_\mathrm{t} v_\mathrm{tg}(r)|_{\max }=r_\mathrm{t} \frac{r}{r_\mathrm{t}}v_\mathrm{tg}(r)= \nonumber \\ r v_\mathrm{tg}(r)&=r \frac{r_\mathrm{t}}{\sqrt{r^2-r_\mathrm{t}^2}} \sqrt{2\triangle \phi + v_\mathrm{r}(r)^2}, \end{aligned}$$

where

45 $$\begin{aligned} \triangle \phi&\equiv \phi (r_\mathrm{t}) - \phi (r) \nonumber \\&= \frac{G{\mathscr {M}}_{\bullet }}{r_\mathrm{t}} + \phi _{\star }(r_\mathrm{t})- \frac{G{\mathscr {M}}_{\bullet }}{r}- \phi _{\star }(r) \end{aligned}$$

If we use the fact that $r \gg r_\mathrm{t}$, then

46 $$\begin{aligned} \frac{G{\mathscr {M}}_{\bullet }}{r_\mathrm{t}} \gg \left( \frac{G{\mathscr {M}}_{\bullet }}{r} + \phi _{\star }(r) \right) = \phi (r) \end{aligned}$$

Also, since $${\mathscr {M}}_{\bullet }\gg {\mathscr {M}}_{\star }(r_\mathrm{t})$$,

47 $$\begin{aligned} \frac{G{\mathscr {M}}_{\bullet }}{r_\mathrm{t}} \gg \phi _{\star }(r_\mathrm{t}). \end{aligned}$$

Thus,

48 $$\begin{aligned} v_\mathrm{lc}(r) \approx \frac{r_\mathrm{t}}{r} \sqrt{ \frac{2G{\mathscr {M}}_{\bullet }}{r_\mathrm{t}}}. \end{aligned}$$

If we use now the fact that

49 $$\begin{aligned} \sigma _\mathrm{r}(r)&=\sigma _\mathrm{r}(r_\mathrm{t}) \left( \frac{r}{r_\mathrm{t}} \right) ^{-1/2}\nonumber \\&= \sqrt{ \frac{G{\mathscr {M}}_{\bullet }}{r_\mathrm{t}}} \left( \frac{r}{r_\mathrm{t}} \right) ^{-1/2}, \end{aligned}$$

we have that

50 $$\begin{aligned} \sqrt{ \frac{G{\mathscr {M}}_{\bullet }}{r_\mathrm{t}}} = \sigma _\mathrm{r}(r) \left( \frac{r}{r_\mathrm{t}} \right) ^{-1/2} \end{aligned}$$

And so,

51 $$\begin{aligned} v_\mathrm{lc}(r) \approx \frac{r_\mathrm{t}}{r} \sqrt{ \frac{2G{\mathscr {M}}_{\bullet }}{r_\mathrm{t}}} \approx \sigma _\mathrm{r}(r) \left( \frac{r_\mathrm{t}}{r} \right) ^{1/2}. \end{aligned}$$

## “Standard” mass segregation

### Introduction

In order to address the question of how many objects a year get close enough to the central MBH to be tidally destroyed, in the case of an extended star, or captured, if a compact object, the zero-th order problem we must solve is how stars distribute around MBHs.

In a system with a spectrum of masses initially distributed uniformely, the more massive ones have a higher kinetic energy than the lighter ones, simply due to the fact that they have the same velocity dispersion but a higher mass. The heavy stars exchange energy with each other and with the light stars through relaxation. The exchange of energy goes in the direction of equipartition, because the system searches the equilibrium. The heavy stars will lose energy to the light ones. When they do so, since they feel their own potential or the potential well of the MBH, their semi-major axis shrinks and they segregate to the centre of the system. When doing so, their kinetic energy will become higher. The system tries to re-equilibrate itself; the velocity dispersion is larger as it was when the massive stars were at larger distances from the centre. As they approach the MBH, their kinetic energy will be higher as compared to the light stars, which are pushed to the outskirts of the system.

**Fig. 21:**
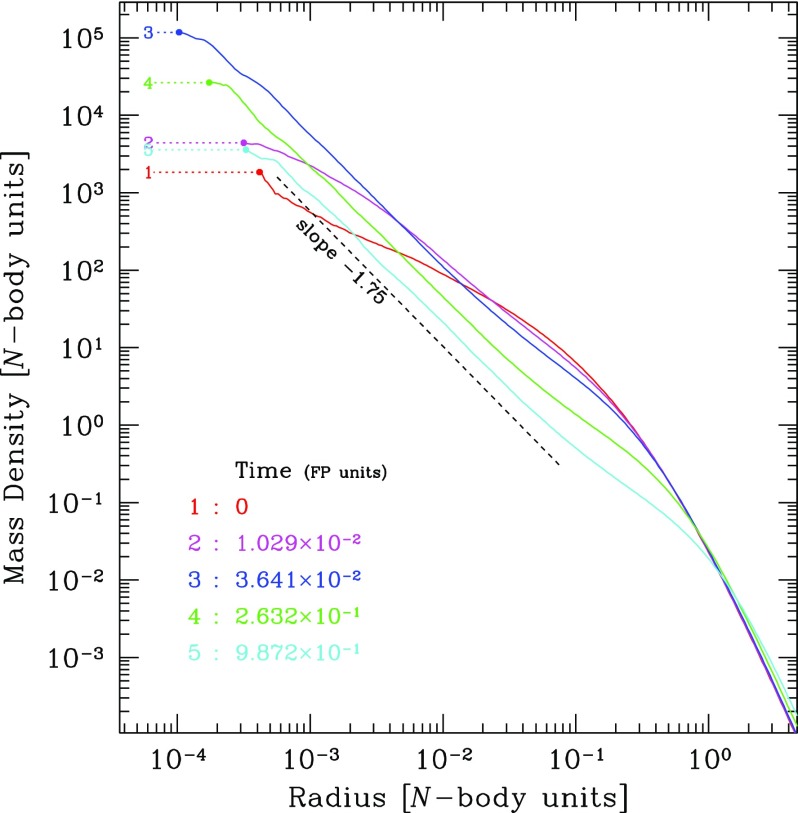
Density profile for a galactic nucleus with a single stellar population in different moments of the evolution of the system, in Fokker–Plank units (FP), as defined in the footnote. Image reproduced with permission from Freitag et al. (2006b), copyright by AAS

In Fig. 21, we have a density profile that shows us the evolution of a single-mass galactic nucleus with a MBH while letting relaxation play a role (i.e., the simulations were run for at least a $T_\mathrm{rlx}$). The initial density profile is depicted in red and shows already a cusp because the authors were using a King model (Freitag et al. 2006a, b), so that it diverges at the centre. When we let it evolve, the profile obtains a much steeper cusp, the blue curve, reaching later a power-law cusp of $\rho \propto R^{-1.75}$. This cusp is kept as the system continues to evolve and the cluster expands. The time units are expressed in Fokker–Plank units.^8^

This is not intuitive. This phenomenon occurs because at the centre we have a sink, the MBH is removing stars, either through tidal disruptions or EMRIs. The stars removed from the system must have a very negative energy, they are very close to the centre, and stars also physically collide with each other and they are partially or totally destroyed in the process, which also represents a loss of stellar mass in the system. For the rest of the system, this represents actually *a source of heat*. The total energy in the system has increased. We can also envisage the picture as follows: the stars that will be removed have to give energy to the rest of the stellar system in order to approach the central sink. When they do so, they heat up the system.

**Fig. 22:**
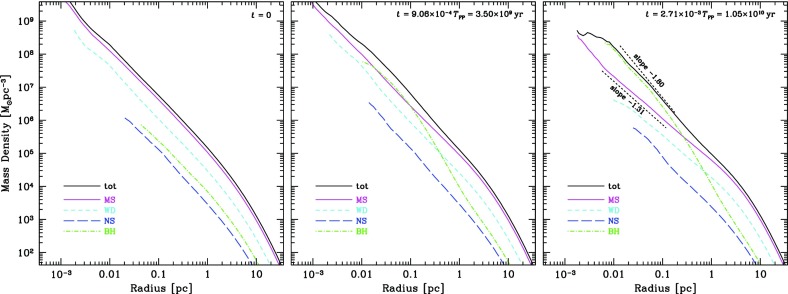
Evolution of a multi-mass system corresponding to Sgr A*. The model contains stellar-mass black holes, with masses between 10 and 30 times larger than MS stars on average. On the left panel, we have the initial conditions, at $t=0$ yr. When we leave the system evolve, the components separate and roughly after a Hubble time we obtain the situation corresponding to the right panel. Image reproduced with permission from Freitag et al. (2006b), copyright by AAS

In Fig. 22, we have a somehow more realistic situation: the authors depict the mass-density distribution for a system that has different stellar components and not only single-mass stars. After some $10^{10}$ yrs the total density has not changed much but in the centre, within $\sim 0.1$ pc, the stellar-mass black holes overwhelmingly defeat the rest of the stellar components. Therefore, within a radius of $\sim 0.1$ pc around a MBH such as the one in our GC, the mass density will be dominated by the stellar-mass black holes. This does not apply to the number density of stellar black holes. They are less numerous as compared to MS stars, but more massive. The important point here is that we expect to have about $2\cdot 10^3$ stellar black holes within 0.1 pc, or $2\cdot 10^4$ within 1 pc of Sgr A* Freitag et al. (2006a, b).

Before we further analyse realistic models with a mass spectrum and address the potential implications for EMRI production, we will start assuming that all stars have the same mass. As we mentioned in the foreword, the main goal of this document is to give a self-consistent starting point to understand the complexity of the different astrophysical phenomena associated with EMRIs. Thus, the first kind of systems I will address will contain only one kind of star. As Donald Lynden-Bell puts it (Lynden-Bell and Wood 1968, p. 515, Sect. 4.5),

*Our other excuse for leaving out high order correlations is that* ***only a fool tries the harder problem when he does not understand the simplest special case.***

In this section, I will illustrate the different phenomena with numerical simulations published for the first time in this review.

### Single-mass clusters

Peebles (1972) was the first to realise^9^ that the statistical thermal equilibrium in a stellar cluster, i.e., the fact that the distribution of energy in the cluster is $f(E) \propto e^{-E/\sigma ^2}$, with $\sigma$ the velocity dispersion, must be violated when we are close to the MBH, because we have three characteristic radii within which stars are lost for the system. These are the tidal radius, $R_\mathrm{t}$, the “Schwarzschild radius” $R_\mathrm{Schw}$ (i.e., the capture radius via gravitational loss), and the collisional radius $R_\mathrm{coll}$. Peebles found that there should be a steady state with a net inward flux of stars and energy in the stellar system. Nevertheless, well within the influence radius $R_{h}$ of the MBH but far from $R_\mathrm{t}$, the stars should have nearly-isotropic velocities. Peebles derived a solution in the form of a power-law for a system in which all stars have the same mass. The quasi-steady solution takes the form (for an isotropic distribution function) $f(E)\sim E^p$, $\rho (r)\sim r^{-\gamma }$, with $\gamma = 3/2 + p$. Nevertheless, Peebles derived the wrong exponent. A few years later, Bahcall and Wolf (1976) did an exhaustive kinematic treatment for single-mass systems and found that the exponent should be $\gamma =7/4$ and $p=\gamma -3/2=1/4$. This solution has been corroborated in a number of semi-/analytical approaches, and approximative numerical schemes, see e.g., Shapiro and Marchant (1978), Marchant and Shapiro (1979), Marchant and Shapiro (1980), Shapiro and Teukolsky (1985), Freitag and Benz (2001), Amaro-Seoane et al. (2004), as well as direct-summation *N*-body simulations, of which the work of Preto et al. (2004) was the first one.

This is one of the most important phenomena in the production of EMRIs, since the galactic nuclei of interest for us, the ones which are thought to be harbouring EMRIs in their cores and are in the range of frequencies of interest, are relaxed. These nuclei are relatively small and are likely to have at least gone through at least one full relaxation time. In general, nuclei in the range of interest for LISA are relaxed (see the rule of thumb introduced in Preto 2010).

### Mass segregation in two mass-component clusters

As we have just seen, the processes that one-component clusters bring about are nowadays relatively well understood and has been plentifully studied by different authors to check for the quality of their approaches. Nonetheless, the properties of multi-mass systems are only very poorly represented by idealised models in which all stars have a single mass. New features of these systems’ behaviour arise when we consider a stellar system in which masses are divided into two groups. Hence, since the idealised situation in which all stars in a stellar cluster have the same mass has been arduously examined in literature, we have the right to extend the analysis a further step. Here I address more realistic configurations in which the stellar system is split into various components. The second integer immediately after one is two, so we will first extend, cautious and wary as we are, our models to two-component star clusters.

Initial mass functions (IMFs), introduced with more detail in Sect. 5.4, ranging between $[0.1,\, \sim 120] M_\odot$ can be approximated to first order by two well-separated mass scales : one with a mass of the order of ${\mathscr {O}}(1 M_\odot )$ (which could represent main-sequence stars, MS, white dwarfs, WD, or neutron stars NS) and ${\mathscr {O}}(10 M_\odot )$ (stellar-mass black holes). Depending on how the system taken into consideration is configured we will exclude *dynamical equilibrium* (meaning that the system is not stable on dynamical time-scales) or equipartition of different components kinetic energies is not allowed (*thermal equilibrium*).

The work of Spitzer Jr (1969) was in this respect pioneering. For some clusters, it seemed impossible to find a configuration in which they enjoy dynamical and thermal equilibrium together. The heavy components sink into the centre because they cede kinetic energy to the light ones when reaching equipartition. The process will carry on until equipartition is fully gained. In most of the cases, equipartition happens to be impossible, because the subsystem of massive stars will undergo core collapse before equipartiton is reached. Anon, a *gravothermal collapse* will happen in this component and, as a result, a small dense core of heavy stars is formed Spitzer Jr (1969), Lightman and Fall (1978). This gravothermal contraction is a product of negative heat capacity, a typical property of gravitationally bound systems Elson et al. (1987).

Different authors have addressed the problem of thermal and dynamical equilibrium in such systems, using techniques such as direct *N*-body Portegies Zwart and McMillan (2000), Khalisi et al. (2007) and Monte Carlo simulations Watters et al. (2000) to direct integration of the Fokker–Planck equation Inagaki and Wiyanto (1984), Kim et al. (1998) or moments of it Amaro-Seoane et al. (2004), including Monte Carlo approaches to the numerical integration of this equation Spitzer Jr and Hart (1971). For a general and complete overview of the historical evolution of two-stars stellar components, see Watters et al. (2000), Amaro-Seoane et al. (2004) and references therein.

If we do not have any energy source in the cluster and stars do not collide (physically), the contraction carries on self-similarly indefinitely; in such a case, one says that the system undergoes *core collapse*. This phenomenon has been observed in a large number of works using different methods Hénon (1973, 1975), Spitzer Jr and Shull (1975), Cohn (1980), Marchant and Shapiro (1980), Stodołkiewicz (1982), Takahashi (1993), Giersz and Heggie (1994), Takahashi (1995), Spurzem and Aarseth (1996), Makino (1996), Quinlan (1996), Drukier et al. (1999), Joshi et al. (2000). Core collapse is not just a characteristic of multi-mass systems, but has been also observed in single mass analysis.

Spitzer Jr (1969) gives the analytical criterion to determine whether a two-component system has achieved energy equipartition. According to this analysis, energy equipartition between the light and heavy component exists if the following inequality holds

52 $$\begin{aligned} {S}:= \left( \frac{{\mathscr {M}}_\mathrm{h}}{{\mathscr {M}}_\mathrm{l}}\right) \left( \frac{m_\mathrm{h}}{m_\mathrm{l}}\right) ^{3/2} < 0.16. \end{aligned}$$

Where ${\mathscr {M}}_\mathrm{l}$ and ${\mathscr {M}}_\mathrm{h}$ are the total numbers of light and heavy components, respectively (i.e., the total stellar mass in light stars and heavy stars in the system). More numerical calculations Watters et al. (2000) have settled this criterion to

53 $$\begin{aligned} {\varLambda }:= \left( \frac{{\mathscr {M}}_\mathrm{h}}{{\mathscr {M}}_\mathrm{l}}\right) \left( \frac{m_\mathrm{h}}{m_\mathrm{l}}\right) ^{2.4} < 0.16 \end{aligned}$$

When we modify the ratio ${\mathscr {M}}_{\max }/{\mathscr {M}}$, the time required to reach core-collapse is different. In a cluster with, for instance, a broad Salpeter initial mass function (IMF) between $[0.2\,M_{\odot },\,120\,M_{\odot }]$, core-collapse takes place after a time $\lesssim 0.1\,t_\mathrm{rh}(0)$, while for a single-mass Plummer model it occurs after a time $\gtrsim 10\,t_\mathrm{rh}(0)$ (this specific example was taken from the Monte Carlo-based calculations of Gürkan et al. 2004).

**Fig. 23:**
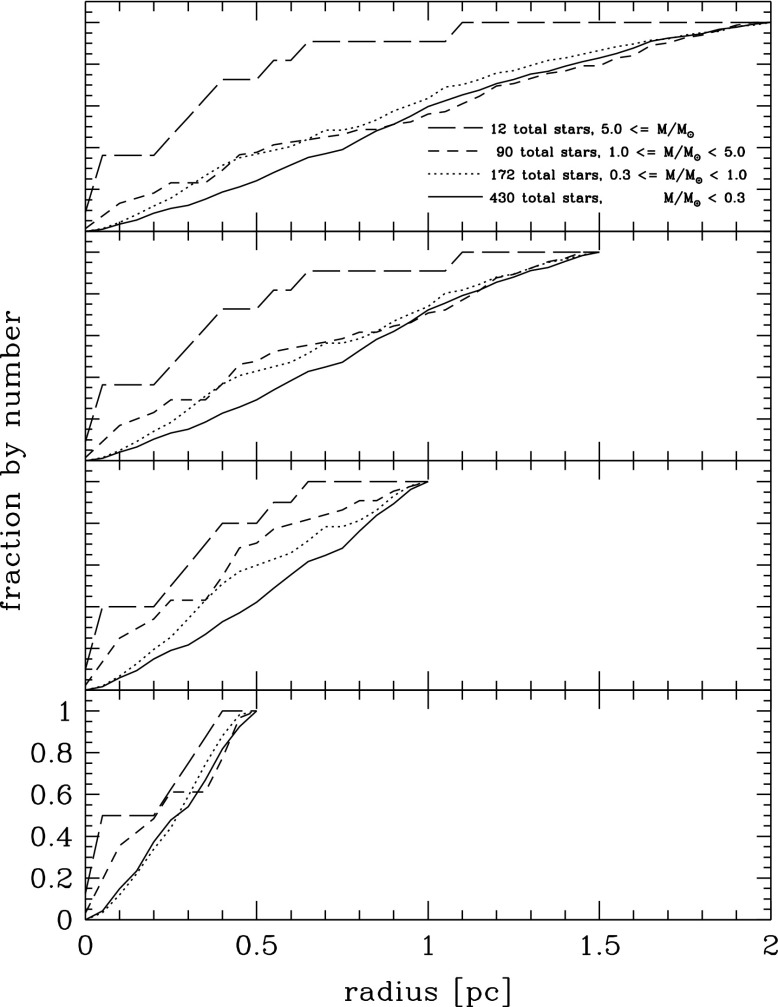
In this plot, we see mass-segregation of stars more massive than $5\,M_{\odot }$ (long-dashed lines) toward the cluster centre and some evidence for general mass segregation persisting down to 1–$2\,M_{\odot }$ in the Orion Nebula cluster. The cumulative radial distributions of source counts over different mass intervals are shown. To clarify the sensitivity of the cumulative plots to the outer radius they have shown here four panels with four different limiting radii. Image reproduced with permission from Hillenbrand and Hartmann (1998), copyright by AAS

There is an ample evidence for mass segregation in observed clusters. McCaughrean and Stauffer (1994) and Hillenbrand and Hartmann (1998) provided deep infrared observations of the Trapezium cluster in Orion that clearly show the mass segregation in the system, with the highest mass stars segregated into the centre of the cluster. To test whether there is evidence for more general mass segregation, they showed in a plot reproduced in Fig. 23 cumulative distributions with radius of stars contained within different mass intervals. They include in the figure four different panels in order to make clear the sensitivity to the limiting radius. They find that, inside 1.0 pc, general mass segregation appears to be established in the cluster, with stars of masses less than 0.3, 0.3–1.0, 1.0–5.0 $M_{\odot }$, and greater than 5 $M_{\odot }$ progressively more centrally concentrated with increasing mass.

**Fig. 24:**
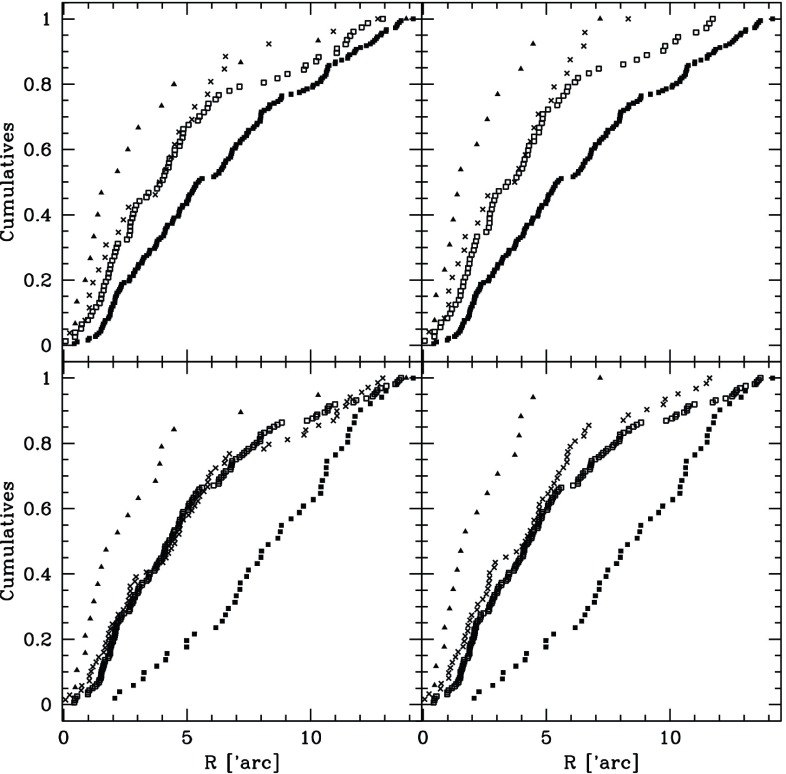
Mass segregation in NGC 623 for two mass interval sets. The two left panels include all sample stars, while the right ones do not include the 9 bright stars of the cluster corona. For the two top figures $M<5\,M_{\odot }$ (filled squares), $M \in \,[5,\,10[\,M_{\odot }$ (open squares), $M \in \,[10,\,20[\,M_{\odot }$ (crosses) and $M \ge 20\,M_{\odot }$ (triangles). For the two bottom figures, $M<2.5\,M_{\odot }$ (filled squares), $M \in \,[2.5,\,6.3[\,M_{\odot }$ (open squares), $M \in \,[6.3,\,15.8[\,M_{\odot }$ (crosses) and $M \ge 15.8\, M_{\odot }$ (triangles). Image reproduced with permission from Raboud and Mermilliod (1998), copyright by ESO

At this point, the question looms up of whether for very young clusters mass segregation is due to relaxation, like in our models, or rather reflects the fact that massive stars are formed preferentially around the centre of the cluster, as some models predict.

Raboud and Mermilliod (1998) addressed the radial structure of Praesepe and of the very young open cluster NGC 6231. There they find evidence for mass segregation among the cluster members and between binaries and single stars. They put it down to the greater average mass of the multiple systems. Figure 24 reproduces a plot of Raboud and Mermilliod (1998), where again we have clear evidence for mass segregation in NGC 6231. In the two first panels, the mass intervals are set in a different way to those in the bottom.

The two left-hand panels of Fig. 24 include the 9 bright stars of the cluster Corona, while on the right do not. The manifestation of mass segregation for massive stars (triangles) is clearly displayed, while stars with masses between $[5,\,20]\,M_{\odot }$ are spatially well mixed (open squares and crosses); i.e., mass segregation is not yet established over a rather large mass interval. This population is more concentrated than the lower-mass population (here shown with filled squares). They derive from Fig. 24 that only a dozen, bright, massive, mainly binary stars are well concentrated toward the cluster centre.

It, therefore, seems interesting to set up multi-mass models with two-components as a starting point, since they are well-studied and we have robust observational evidence of this phenomenon. On the other hand, observations do not tell us whether mass segregation is due to relaxation. I now show the results from a set of $10^4$ simulations for two-component models using the “Gaseous Model” programme to illustrate this (see Sect. 8). I define two parameters now that describe the physics of the system,

54 $$\begin{aligned} q&:= {{\mathscr {M}}_\mathrm{h}}/{\mathscr {M}},\nonumber \\ \mu&:= {m_\mathrm{h}}/{m_\mathrm{l}} \end{aligned}$$

In this definition, ${\mathscr {M}}$ is the total mass of the system, ${\mathscr {M}}_\mathrm{h}$ the total mass in heavy stars and ${m_\mathrm{h,\,l}}$ the mass of one heavy (light) star. In the expression, *q* is the total stellar mass in heavy stars normalised to the total mass of the system, and $\mu$ the mass ratio between heavy and light stars.

Now, I introduce the quantity $\zeta \equiv 1-q$, and we let $\zeta$ vary from $10^{-4}$ to $9.99 \cdot 10^{-1}$. For each $\zeta$ value, we let $\mu$ vary between 1.03 and $10^3$. The values for *q* are regularly distributed in $\log {(\zeta )}$. For $\zeta \approx 1$ we have added a series of values in $\log {(\zeta -1)}$. The mean particle mass is $1,M_{\odot }$ and the total mass $10^6 \,M_{\odot }$, but this is not important for our study, because the physics of the system is driven by relaxation and therefore the only relevant concept is the relaxation time. We can always extend the physics to any other system containing more particles, with the proviso that only relaxation is at play. The mean mass is therefore just a normalisation. What really determines the dynamics of the system are the mass ratios, *q* and $\mu$, which is the reason why I use them to explore the system.

**Fig. 25:**
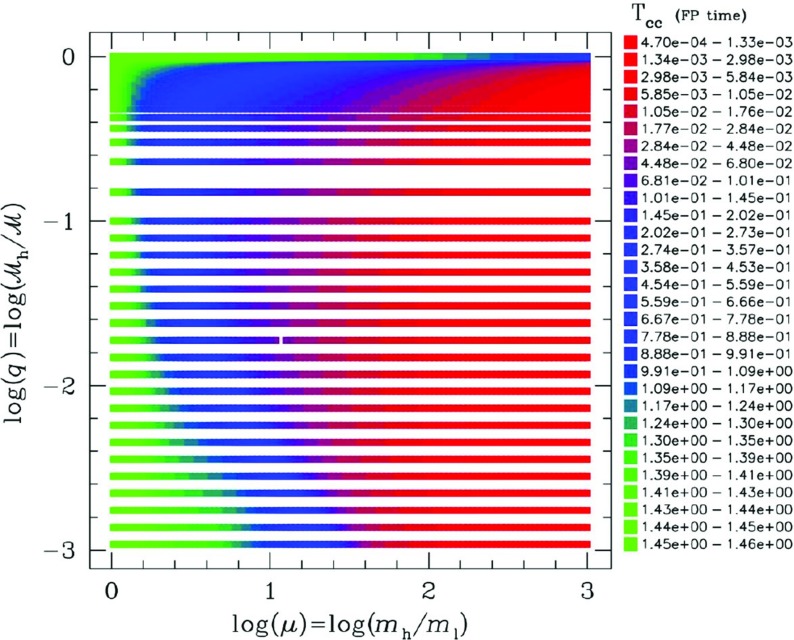
Parameter space for the set of $10^4$ simulations. Here, $t_\mathrm{end}$ stands for the core collapse time and is expressed in FP units (see text); time at which the simulation ended. *q* and $\mu$ are plotted logarithmically

In Fig. 25, I show the whole $(q,\,\mu )$-parameter space in a plot where the time at which the core-collapse begins is included. The green zone corresponds to the quasi single-mass case. In the red zone we have the largest difference between masses and blue is an intermediate case.

**Fig. 26:**
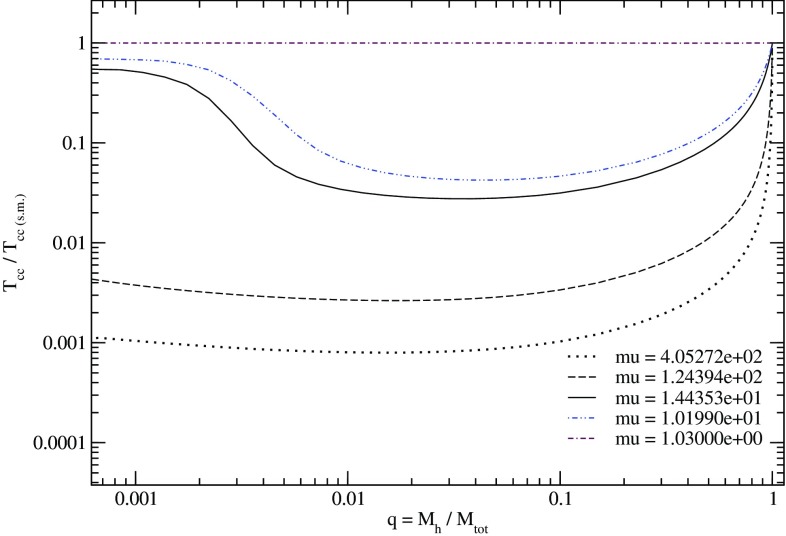
Core-collapse time for different values of *q* and $\mu$

Figure 26 shows collapse times for cluster models with two mass components normalised to the single-mass core-collapse time for different values of $\mu$. The initial clusters are Plummer spheres without segregation. The collapse times are displayed as a function of the mass fraction of the heavy component in the cluster. When compared to single-mass component systems, we see that the core-collapse time is accelerated notably for a wide range of the heavy component ${\mathscr {M}}_\mathrm{h}$ ($M_2$). Even a small number of heavy stars accelerate the core-collapse time.

It is really interesting to compare the capacity of our approach by comparing the results of this set of simulations to the *N*-body calculations of star clusters with two-mass components performed by Khalisi et al. (2007) with direct-summation techniques. For this aim, I plot the evolution of the average mass in Lagrangian shells of the cluster from the averaged mass in Lagrangian *spheres* containing the following mass percentages [0–1], [2–5], [10–20], [40–50], [75–95]%, among others, to be able to compare with the results of Khalisi et al. (2007). These are the comprised volume between two Lagrangian radii, which contain a fixed mass fraction of the bound stars in the system.

**Fig. 27:**
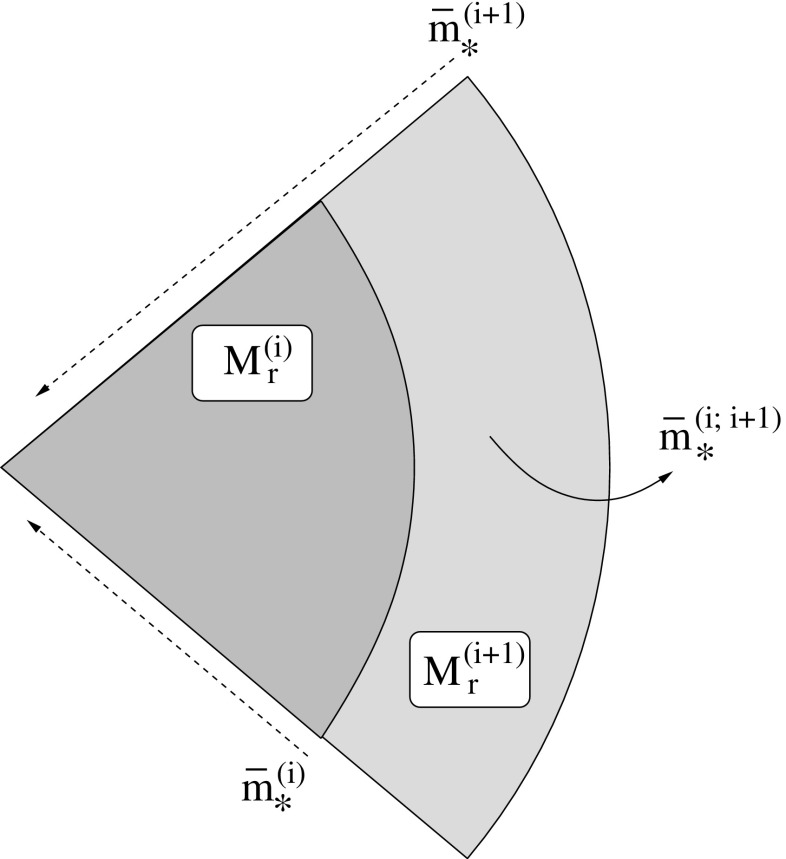
Average mass in Lagrangian shells from averaged mass in Lagrangian spheres

We have calculated the average mass as follows: If $M_\mathrm{r}^{(\mathrm{i})}$ is the total mass for the component $\mathrm{i}$ comprised at the radius r and $\bar{m}_{\star }^{(\mathrm{i})}$ is the average mass for this component within that radius, we can find out what is the value of $\bar{m}_{\star }^{(\mathrm{i};\,\mathrm{i}+1)}$ (the average mass between $\bar{m}_{\star }^{(\mathrm{i})}$ and $\bar{m}_{\star }^{(\mathrm{i}+1)}$) knowing $M_\mathrm{r}^{(\mathrm{i})}$, $M_\mathrm{r}^{(\mathrm{i}+1)}$, $\bar{m}_{\star }^{(\mathrm{i})}$ and $\bar{m}_{\star }^{(\mathrm{i}+1)}$. This is schematically shown in Fig. 27. Indeed,

55 $$\begin{aligned} M_\mathrm{r}^{(\mathrm{i}+1)} =N_\mathrm{r}^{(\mathrm{i})}\cdot \bar{m}_{\star }^{(\mathrm{i})} + N_\mathrm{r}^{(\mathrm{i};\,\mathrm{i}+1)} \cdot \bar{m}_{\star }^{(\mathrm{i};\,\mathrm{i}+1)} = N_\mathrm{r}^{(\mathrm{i}+1)} \cdot \bar{m}_{\star }^{(\mathrm{i}+1)}. \end{aligned}$$

Since

56 $$\begin{aligned} N_\mathrm{r}^{(\mathrm{i}+1)}=N_\mathrm{r}^{(\mathrm{i})}+N_\mathrm{r}^{(\mathrm{i};\,\mathrm{i}+1)}, \end{aligned}$$

where

57 $$\begin{aligned} N_\mathrm{r}^{(\mathrm{i})}=\frac{M_\mathrm{r}^{(\mathrm{i})}}{\bar{m}_{\star }^{(\mathrm{i})}} \end{aligned}$$

we have that, from Eq. (55),

58 $$\begin{aligned} \bar{m}_{\star }^{(\mathrm{i};\,\mathrm{i}+1)}= \frac{M_\mathrm{r}^{(\mathrm{i}+1)}-M_\mathrm{r}^{(\mathrm{i})}}{\frac{M_\mathrm{r}^{(\mathrm{i}+1)}}{\bar{m}_{\star }^{(\mathrm{i}+1)}} - \frac{M_\mathrm{r}^{(\mathrm{i})}}{\bar{m}_{\star }^{(\mathrm{i})}}} \end{aligned}$$

**Fig. 28:**
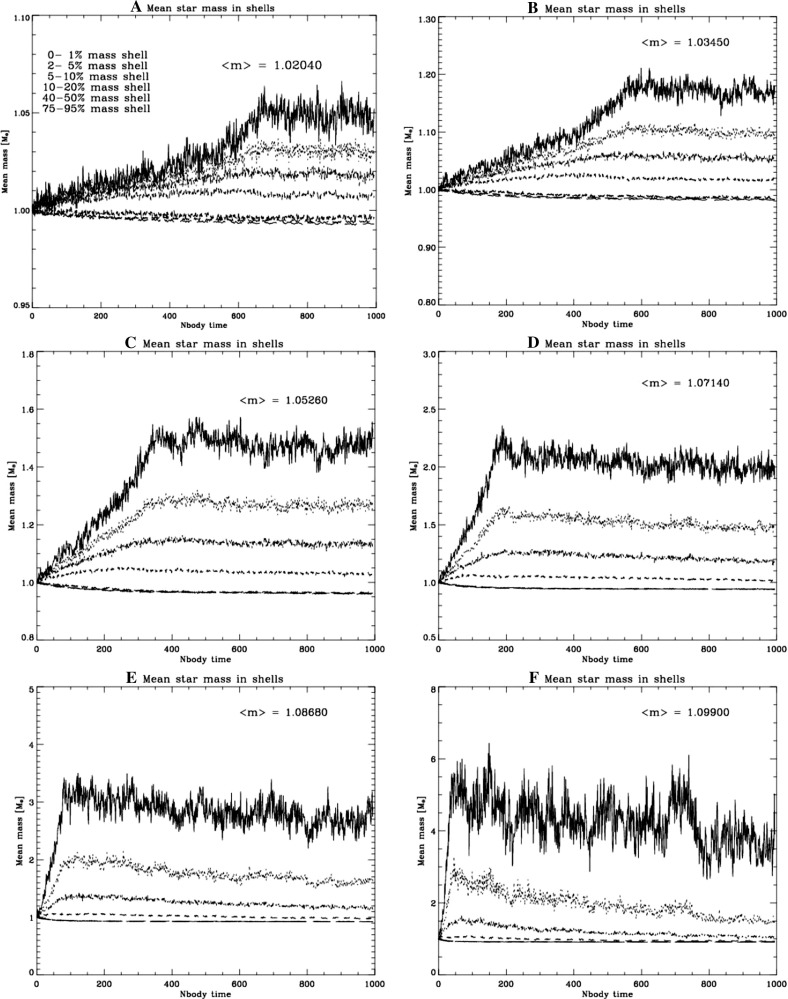
Average Lagrangian radii shells for the *N*-body models of Khalisi et al. (2007) (see the text for further explanation)

**Fig. 29:**
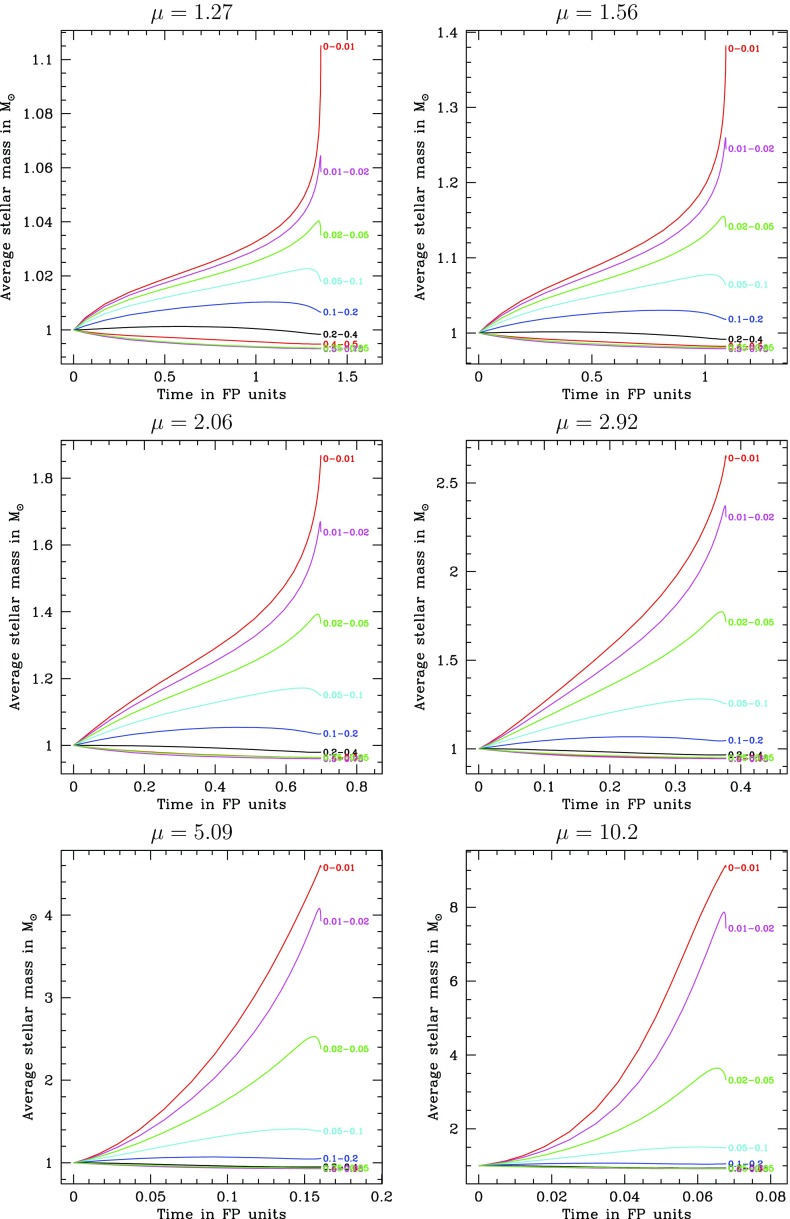
Average Lagrangian radii shells for our models, equivalent to those of Fig. 28

Figures 28 and 29 show the curves corresponding to the values shown in Table 1.

**Table 1:** Different $\mu$ values used in the *N*-body calculations and in our gaseous model results of Fig. 28

$\mu$ in Khalisi et al. (2007)	$\mu$ in this work
1.25	1.27
1.5	1.56
2	2.06
3	2.92
5	5.09
10	10.2

We have followed in the curves the evolution of the system until a deep collapse of the system. These figures show the evolution until the most massive component dominates the centre.

In order to compare our plots with those of Khalisi et al. (2007), one should look at their diagrams in the region *during* core contraction. At this point, we can observe in Fig. 28 a self-similarity after core-collapse (Giersz and Heggie 1996). Binaries are responsible for interrupting core-collapse and driving core re-expansion in the *N*-body simulations. The flattening in the *N*-body plots at the moment of core-collapse is due to the binary energy generation. This means that we can only compare the steep rise, but not the saturation.

For instance, in the second plot of the *N*-body set (second column on the top of Fig. 28), we have to look at the point at which the average mass of the *N*-body system is about 1.20 in the 0–1% shell. This establishes the limit until which we can really compare the behaviour as given by both methods. Our simulations yield a very similar evolution until that point. The gaseous model behaves (it clearly shows the tendency) like the *N*-body result.

By converting the Fokker–Planck units, we find that the conversion factor is the same; namely, for $\gamma =0.11$, $\ln (\gamma \cdot {\mathscr {N}}_{\star })/ {\mathscr {N}}_{\star }= 0.0022$. On the other hand, the value of $\gamma$ is not so well defined and depends on the mass spectrum (Hénon 1975). This means that potentially it is not the same for the different models. For a broader mass spectrum, $\gamma$ is about 0.01 and, unfortunately, in the case of having a small particle number, it will definitively make an important difference despite the “smoothing” effect of the logarithm, viz $\ln (\gamma \cdot {\mathscr {N}}_{\star })/ {\mathscr {N}}_{\star } = 0.0013$. Thus, in order to be able to compare the different models, one should consider $\gamma$ as a free parameter ranging between 0.01 and 0.2 and look for the best fit for the majority of cases. On the other hand, we must bear in mind that the *N*-body simulations of Khalisi et al. (2007) do not go into deep core collapse and so, the moment at which the core radius reaches a minimum is not the same as for our model. To sum up, although we cannot say exactly to what point we can compare the two methods (the Gas Model and direct-summation simulations), because the core collapse time will be different, the physics of the system is the same in the two cases. This should provide the reader with a good understanding of the phenomena in play, as well as a proof that they are independent of the details of the algorithm used.

### Clusters with a broader mass spectrum with no MBH

In order to understand the phenomena that I will describe later, which is crucial for EMRI formation, it is of relative relevance to understand first the physics behind cluster dynamics *without* a central MBH. This section is also interested in interpreting observations of young stellar clusters extending to a larger number of mass components. In clusters with realistic IMFs, equipartition cannot be reached, because the most massive stars build a subsystem in the cluster’s centre as the process of segregation goes on thanks to the kinetic energy transfer to the light mass components until the cluster undergoes core collapse (Spitzer Jr 1969; Inagaki and Wiyanto 1984; Inagaki and Saslaw 1985). Although the case in which the MBH is lurking at the centre of the host cluster is more attractive for EMRI production and from a dynamical point of view, one should study, in a first step, more simple models.

In this section we want, thus, to go a step further and evaluate stellar clusters with a broad mass function (MF hereafter). For this, I will again be using the Gas Model, because it is a good compromise between accuracy and integration time for this review.

We study those clusters for which the relaxation time is relatively short, because the most massive stars will sink to the centre of the system due to mass segregation before they have time to leave the main sequence (MS). In this scenario we can consider, as an approximation, that stellar evolution plays no role; stars did not have time to start evolving. The configuration is similar to that of Gürkan et al. (2004), but they employ a rather different approach based on a Monte Carlo code (MC), using the ideas of Hénon (1973) that allow one to study various aspects of the stellar dynamics of a dense stellar cluster with or without a central MBH. Our scheme, although being more approximate than MC codes (and direct-summation *N*-body ones) and unable, in its present version, to account for collision has the advantage, as we will see in the Sect. 8, of being much faster to run, and of providing data that has no numerical noise. It captures the essential features of the physical systems considered in our analysis and is an interesting, powerful tool for illustrating the different scenarios in this review.

One of the first questions we should address is the maximum number of components one should take into consideration when performing our calculations. Since the computational time becomes larger and larger when adding more and more components to the system -even for an approximative scheme such as the Gas Model-, we should first find out what is a realistic number of components in our case. For this end I have performed different computations with different number of stellar components.

For the simulations shown here, the initial cluster models are Plummer models with a Salpeter IMF (Salpeter 1955),

59 $$\begin{aligned} \frac{dN_{\star }}{dM_{\star }} \propto M_{\star }^{-\alpha } \end{aligned}$$

between 0.2 and $120\,M_{\odot }$. In this equation $\alpha =2.35$. There is no initial mass segregation. The discretisation of the mass components follows this recipe:

60 $$\begin{aligned} \log (M_\mathrm{comp}|_i) =\log (M_{\min })+ \log \left( \frac{M_{\max }}{M_{\min }}\right) \cdot \left( \frac{i}{N_\mathrm{comp +1}} \right) ^{\delta } \end{aligned}$$

In this equation, $\delta$ is the discretisation exponent. If $\delta >1$ we have more bins at low mass; for $\delta <1$, we have more bins at high mass. That is, $\delta$ allows one to put more discretised mass components at low masses ($\delta >1$) or at high masses ($\delta <1$), $\delta =1$ gives the logarithmical equal spacing. $M_{\max ,\min }$ are, respectively, the maximum and minimum individual stellar masses for the components. For all simulations that I present, the number of mass bins has been typically set to 15. I have chosen a Plummer model by default and the stellar clusters have $10^6$ stars. The model radius by default is $R_\mathrm{Pl}=1$ pc. The default initial mass function is Salpeter.

**Fig. 30:**
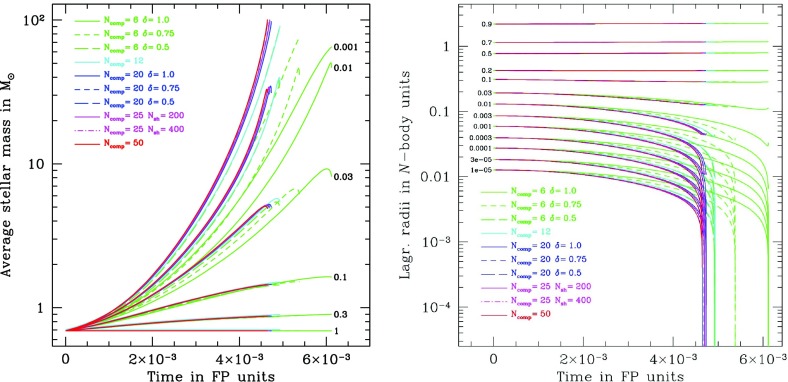
Lagrangian radii and average stellar mass for 10 models with different mass spectrum (see text for details)

In Fig. 30 we see the Lagrangian radii for ten different models and look for the main dynamical characteristics of the system: the core collapse time and the Lagrangian radii containing 90, 70, 50, 20, 10, 3, 1, 0.3, 0.1, $3\cdot 10^{-2}$, $10^{-2}$, $3\cdot 10^{-3}$ and $10^{-3}\%$ of the stellar mass. In this plot, $N_\mathrm{comp}$ stands for the mass spectrum different components number. For $N_\mathrm{comp}=6$ I have performed three simulations varying the $\delta$ parameter between 1.0 (equal logarithmic spacing of components), 0.75 (more massive components) and 0.5 (even more). For $N_\mathrm{comp}=12$ I have performed only one simulation (with $\delta =1$, by default); for the $N_\mathrm{comp}=20$ case I have repeated the same procedure as with $N_\mathrm{comp}=6$, the penultimate one that I have chosen is $N_\mathrm{comp}=20$ and, in this case, we studied two grid resolutions, $N_\mathrm{sh}=200$ (the default value) and 400 grid points, in order to check whether this could influence the results. To finish with, a last simulation with $N_\mathrm{comp}=50$ was performed and included in the analysis. Whilst we can see an important difference between models of 6 and 12 components, we see that the global behaviour from 12 components onwards is very similar. Therefore, unless indicated, I choose 15 components in our study in this section, since a higher number would not contribute anything essential.

**Fig. 31:**
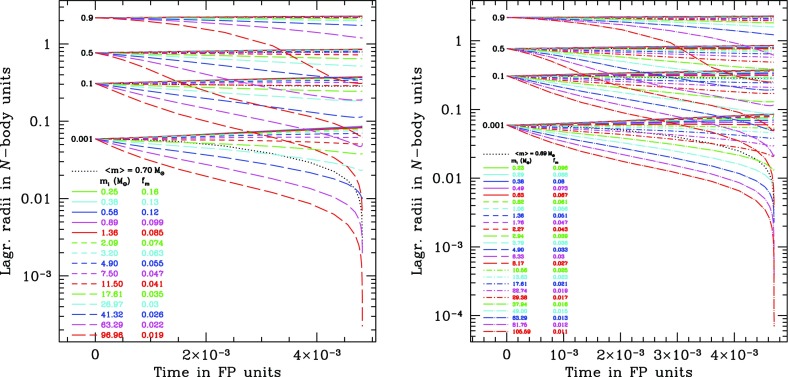
Lagrangian radii for each stellar mass $m_i$ for the cases of 25 and 15 mass components

To see this in more detail, I show the Lagrangian radii for each stellar mass $m_i$ and the corresponding mass fraction $f_m$ for the 25 and 15 components simulations in Fig. 31. Again, we cannot see any substantial difference between the 25 and 15 cases.

**Fig. 32:**
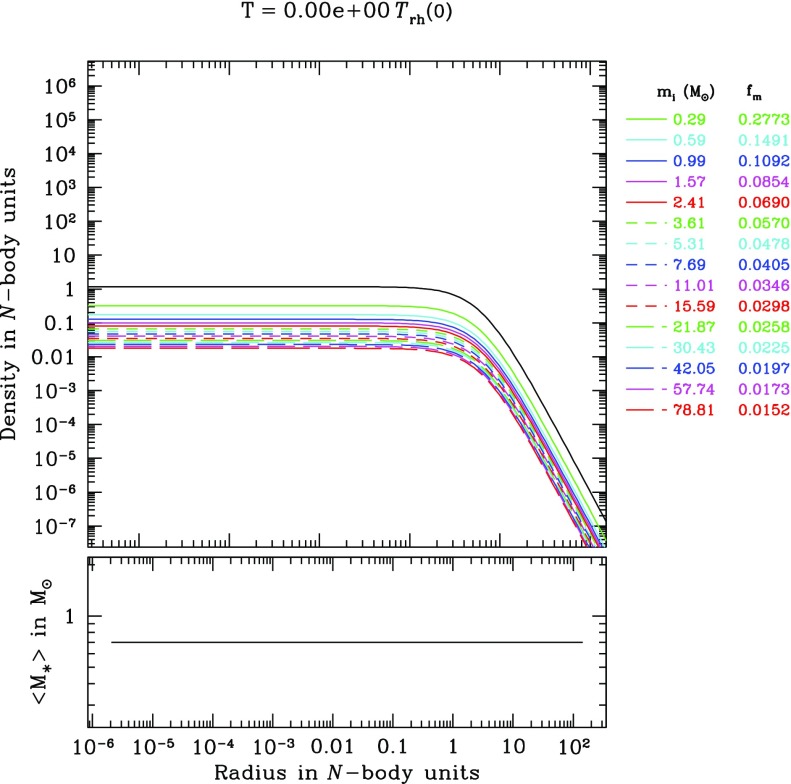
Initial density profile for a stellar cluster with 15 components (upper panel) in *N*-body units and average total stellar mass in $M_{\odot }$ (lower panel)

**Fig. 33:**
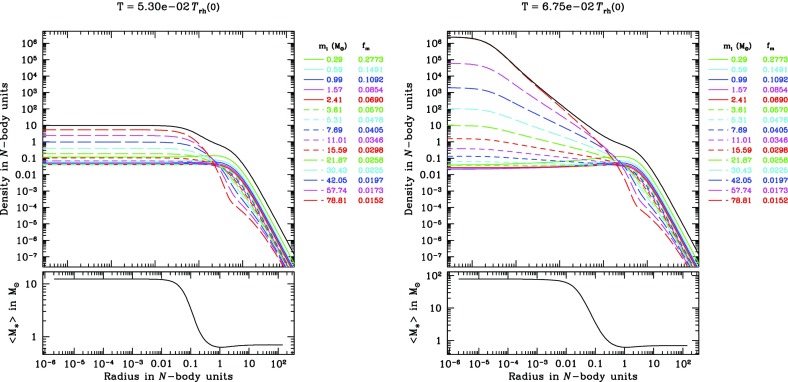
Same situation as in Fig. 32 but at later times. See text for explanations

Taking the last arguments into account, I have done an analysis of mass segregation in multi-mass models with more than two stellar components without MBH. In Figs. 32 and 33, I show the evolution of a stellar cluster of 15 components (in colours); *m* is the mass (in $M_{\odot }$) of the stars in each component and $f_m$ the corresponding fraction of the total mass. In the upper box we have the density profile, where the solid black line represents the total density; below, we have the average total mass for the system. I show different snapshots of the system. At $T=0$ we have the initial model, which duly shows no mass segregation. As time passes, at $T=5.30\cdot 10^{-2}\,T_\mathrm{rh}(0)$, with $T_\mathrm{rh}(0)$ the value of $T_\mathrm{rh}$ at the beginning of the simulation, we observe how mass segregation has fragmented the initial configuration; the heavy components have sunk into the central regions of the stellar cluster and, thus, increased the mean average mass. The outer parts of the system start losing their heavy stars quickly and, consequently, their density profile decreases. This becomes more acute for later times at $T=6.75\cdot 10^{-2}\,T_\mathrm{rh}(0)$, as the plots on the right in Fig. 33 show. In these plots and, more clearly in the right panel of density profile, we can observe a depletion at intermediate radii.

### Core-collapse evolution

**Fig. 34:**
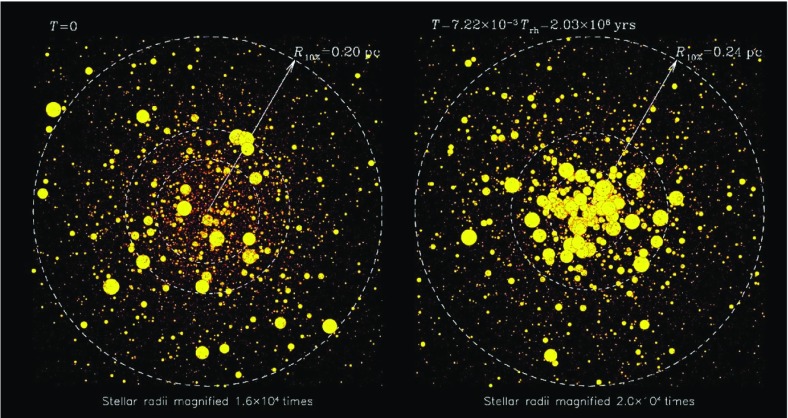
Illustration of core-collapse in multi-mass systems treated with a Monte Carlo approach (courtesy of M. Freitag)

**Fig. 35:**
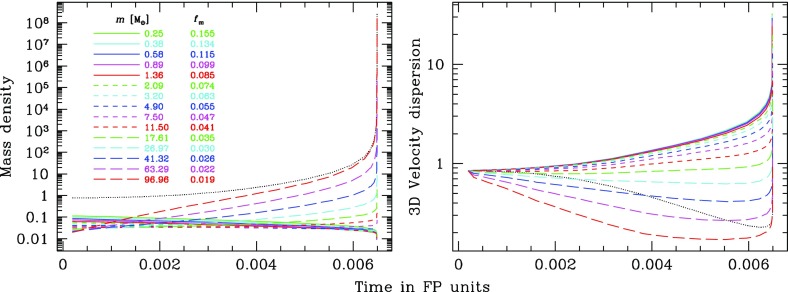
Evolution of the central density and 3*D*-velocity dispersion in a model with 15 components (see text for further explanations)

Gürkan et al. (2004) show that for a broad MF—either Salpeter or Kroupa—, mass segregation produces a core-collapse of the system that happens very fast. For clusters of moderate initial concentration, they find that this happens in about 10% of the $T_\mathrm{rh}(0)$, the initial half-mass relaxation time (i.e., the half-relaxation time that the cluster had when time started, at $t=0$). A good and clear illustration of this is Figs. 34 and  35. In the former, on the left panel we have the initial configuration of the system. On the right one, we have the cluster at the moment of core-collapse. In the figure, all stars within a slice containing the centre have been depicted. On the other hand, this does not represent a real physical system, because all radii have been magnified (see the bottom of each panel). The dashed circles represent spheres containing 1, 3 and 10 % of the total cluster mass (from the centre). We can clearly see how the massive, large stars are segregated towards the centre. In Fig. 35, I show the core-collapse evolution of a multi-mass stellar cluster simulated with the gaseous model. As usual, *m* is the mass (in $M_{\odot }$) of the stars in each component and $f_m$ the corresponding fraction of the total mass. On the left panel I display the time evolution of the central density for a model in which I have employed 15 individual mass components. The total density is given by the dotted line. On the right panel we have the evolution of the central velocity dispersions. The dotted black line shows the mass-averaged value

61 $$\begin{aligned} \bar{\sigma }^2=\sqrt{\frac{\sum _{i=1}^{15}m_i\,\sigma _i^2}{\bar{m}}}, \end{aligned}$$

and I use *N*-body units for the *y*-axes.

One notes that, during core collapse, the central regions of the cluster become completely dominated by the most massive stars. But, contrary to the case of single-mass clusters, the central velocity dispersion *decreases* (see Fig. 35).

### Clusters with a broader mass spectrum with a MBH

Afer having addressed the systems studied in previous sections we now look into the dynamical problem of a multi-mass component cluster harbouring a central seed MBH that grows due to stellar accretion.

In this section, I extend our analysis to systems for which I use an evolved mass function of an age of about 10 Gyr. We consider a mass spectrum with stellar remnants. We employ a Kroupa IMF (Kroupa et al. 1993; Kroupa 2001) with ZAMS mass^10^ from 0.1 to $120\,M_{\odot }$ with the turn-off mass of $1\,M_{\odot }$. I have chosen the following values for the exponent according to the mass interval,

62 $$\begin{aligned} \alpha = \left\{ \begin{array}{lll} 1.3, &{} 0.008 \le m_{\star }/M_{\odot }< 0.5 \\ 2.2, &{} 0.5 \le m_{\star }/M_{\odot }< 1 \\ 2.7, &{} 1 \le m_{\star }/M_{\odot }\le 120. \end{array} \right. \end{aligned}$$

And with the following distribution of components,

(i) Main sequence stars of 0.1–$1\,M_{\odot }$ ($\sim$ 7 components)
(ii) White dwarfs of $\sim \, 0.6\,M_{\odot }$ (1 component)
(iii) Neutron stars of $\sim \,1.4\,M_{\odot }$ (1 component)
(iv) Stellar black holes of $\sim \,10M_{\odot }$ (1 component)

The defined IMF evolves and provides an evolved population with compact remnants. This means that main sequence stars can be transformed into white dwarfs, neutron stars or stellar-mass black holes according to their masses. If $m_\mathrm{MS}$ is the mass of a MS star, I have defined the following mass ranges for the evolution into compact remnants:

- White dwarfs in the range of $1 \le m_\mathrm{MS}/M_{\odot }< 8$
- Neutron stars for masses $8 \le m_\mathrm{MS}/M_{\odot }< 30$
- Stellar black holes for bigger masses, $\ge 30 M_{\odot }$

As I have already mentioned, I place at the centre a seed BH whose initial mass is $50\,M_{\odot }$. The initial model for the cluster is a Plummer sphere with a Plummer radius $R_\mathrm{Pl}=1$ pc. The total number of stars in the system is ${\mathscr {N}}_\mathrm{cl}=10^6$.

**Fig. 36:**
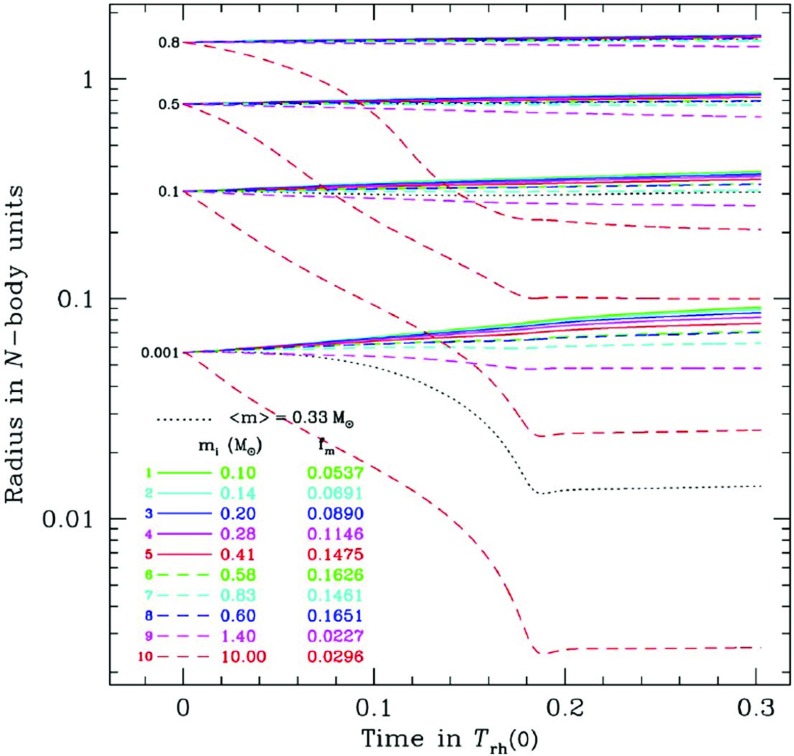
Lagrange radii evolution for a 10 components calculation with a seed BH and stellar remnants

**Fig. 37:**
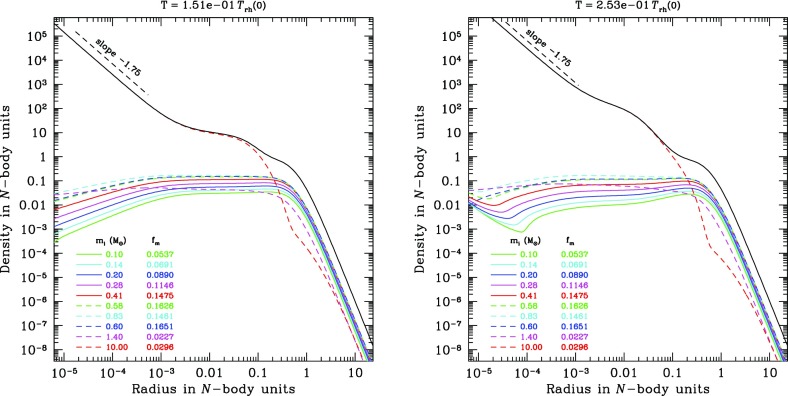
Density profiles in a multi-mass system with seed BH before and after core-collapse (see text)

The presence of a small fraction of stellar remnants may greatly affect the evolution of the cluster and growth of the MBH because they segregate to the centre, and in doing so, they expel MS stars from it but, being compact, they cannot be tidally disrupted. This kind of evolution is shown in Figs. 36 and 37.

Figure 36 shows us the time evolution of different Lagrange radii with 0.1, 10, 50, 80% of the mass of each component. Here, the core collapse happens at about $T=0.18\,T_\mathrm{rh}(0)$. The later re-opening out is due MBH accretion.

In Fig. 37, I plot the density profiles of the system before and after the post-collapse phase. We can also see that the slope of $\rho \propto R^{-7/4}$ on account of the cusp of stellar-mass black holes that has formed around the central MBH. We can see how the different components redistribute in the process, as I mentioned at the beginning of this section. We can see how the MBH dominates the dynamics at the centre.

We can study how the system evolves from the point of view of the distribution of kinetic energies between the different components of the clusters during the process of mass segregation.

**Fig. 38:**
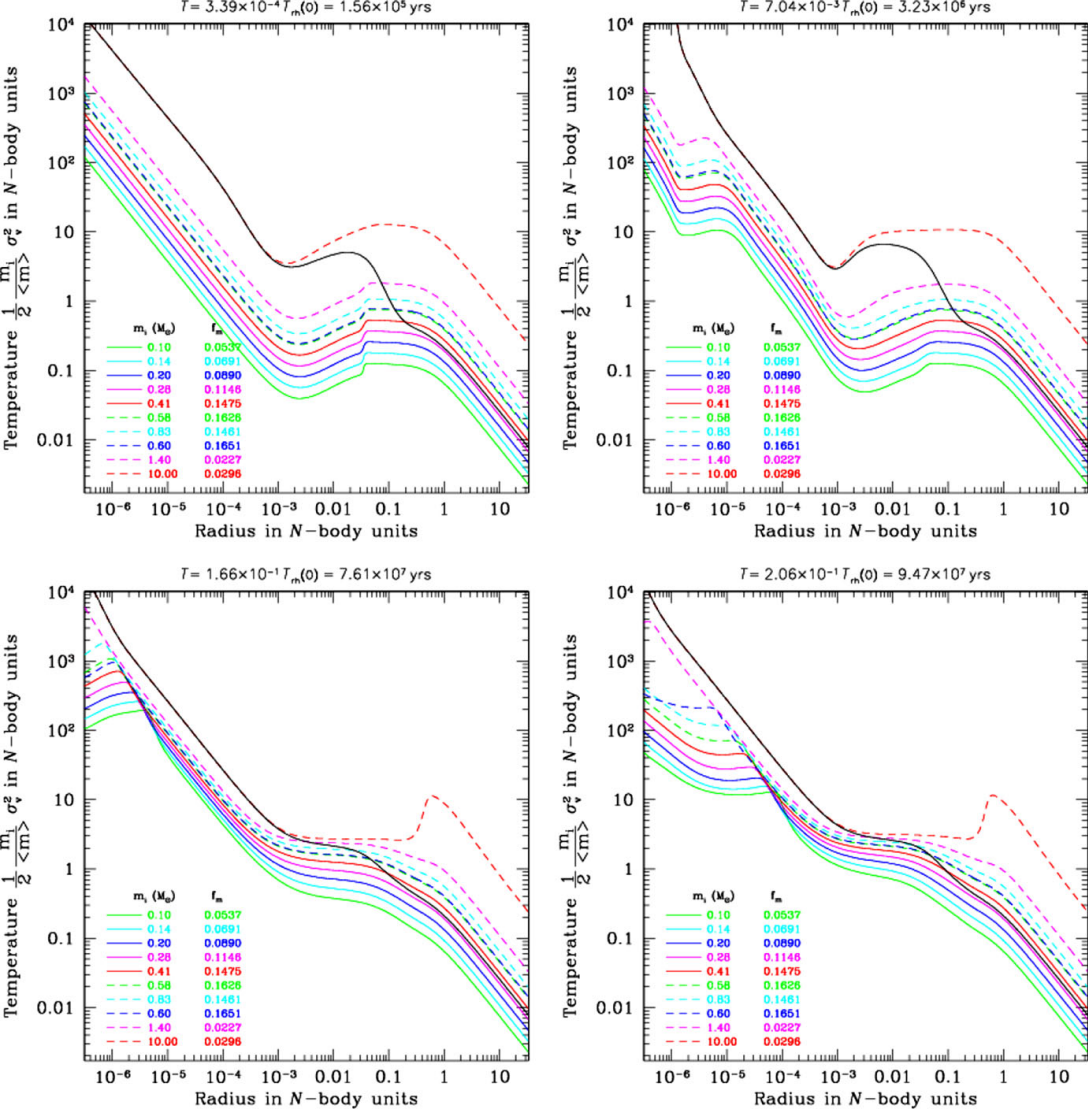
Different moments in the evolution of the cluster temperature for a 10 stellar components system with a seed MBH

Figure 38 shows the evolution of the “temperature” of the system, defined as the mean kinetic energy per star divided by the global mean mass (in order to have a “temperature” expressed in square velocity units). In this plot, I show the core-collapse situation corresponding to Figs. 36 and 37. I consider a 10 component cluster with the characteristics explained before. The mean temperature is defined as

63 $$\begin{aligned} \langle T \rangle = \frac{\sum n_\mathrm{i}\,T_\mathrm{i}}{\sum n_{i}}, \end{aligned}$$

where $n_\mathrm{i}$ is the numerical local density for component *i*. This corresponds to the mean kinetic energy per star. We can see in Fig. 38 that it is about the same as the heaviest component in the inner regions, even though one could think that segregation should not have set in the beginning. This is due to the fact that the moment does not correspond to exactly the initial moment, $T=0$. We can already see how the mean central temperature moves back as time passes (solid black line) and the most massive component (dashed red line) increases. For later times, the kinetic energies of the different components rise at the inner part of the cluster and the most massive one approaches the sum of all of them. This is even more evident in the last plot, where the temperatures of all components sink except for that corresponding to the most massive one.

## Two-body extreme mass ratio inspirals

After the first sections we have a good understanding of the fundamentals of two-body relaxation in dense stellar systems, including mass segregation and dynamical friction, which could be roughly described as “relaxation when we have a large mass ratio”. In this section, I address the subject of capture of compact objects by a massive black hole considering that the driving mechanism in the production is two-body relaxation.

### A hidden stellar population in galactic nuclei

The question about the distribution and capture of stellar-mass black holes at the Galactic Centre has been addressed a number of times by different authors, from both a semi- or analytical and numerical standpoint, see e.g., Sigurdsson and Rees (1997), Miralda-Escudé and Gould (2000), Freitag (2001, 2003b), Freitag et al. (2006a, b), Hopman and Alexander (2006b), Amaro-Seoane et al. (2007), Preto and Amaro-Seoane (2010), Amaro-Seoane and Preto (2011). Addressing this problem has implications for a variety of astrophysical questions, including of course inspirals of compact objects onto the central MBH, but also on the distribution of X-ray binaries at the Galactic Centre, tidal disruptions of main sequence stars, and the behaviours of the so-called “source” stars, which were introduced in Sect. 2.2. Even if we only consider single stellar-mass black holes, the impact they can have on the S-stars is not negligible; a distribution of non-luminous matter around the Galactic Centre would have a clear fingerprint on their orbits. Current data are insufficient to detect such an extended non-luminous cusp which typically would induce a slight Newtonian retrograde precession (Mouawad et al. 2005), so that we will have to wait for future telescopes before we can hope to see such trajectory deflections. The study of Weinberg et al. (2005) estimated that proposed 30–100 m aperture telescopes will allow us to observe about three trajectory deflections per year between any of the monitored “source” stars and a stellar-mass black hole.

The centermost part of the stellar spheroid, the *galactic nucleus,* constitutes an extreme environment for stellar dynamics. With stellar densities higher than $10^6\,M_{\odot }\,\mathrm {pc}^{-3}$, relative velocities in excess of 100 $\mathrm{km\,s}^{-1}$ the nucleus (unlike most of the rest of the galaxy) is the site of a variety of “collisional processes”—both close encounters and actual collisions between stars, as we have seen in the previous sections. The central MBH and the surrounding stellar environment interact through various mechanisms: some are global, like the accretion of gases liberated by stellar evolution or the adiabatic adaptation of stellar orbits as the mass of the MBH increases; others, which involve the close interaction between a star and the MBH—EMRIs and stellar disruptions—are local in nature. As we have seen in Sect. 4.4, to interact closely with the central MBH, stars have to find themselves on “loss-cone” orbits, which are orbits elongated enough to have a very close-in periapsis (Frank and Rees 1976; Lightman and Shapiro 1977; Amaro-Seoane and Spurzem 2001).

The rate of tidal disruptions can be established (semi-)analytically if the phase space distribution of stars around the MBH is known, see Magorrian and Tremaine (1999), Syer and Ulmer (1999), Wang and Merritt (2004) for estimates in models of observed nearby nuclei. However, in order to account for the complex influence of mass segregation, collisions and the evolution of the nucleus over billions of years, detailed numerical simulations are required, as in the work of David et al. (1987a, b), Murphy et al. (1991), Freitag and Benz (2002), Baumgardt et al. (2004b), Freitag et al. (2006b), Khalisi et al. (2007), Preto and Amaro-Seoane (2010), Amaro-Seoane and Preto (2011).

In the case of a gradual inspiral following the “capture” of a compact object (i.e., an EMRI), the situation becomes even more complex, even in the idealised case of a spherical nucleus with stars all of the same mass. As the star spirals down towards the MBH, it has many opportunities to be deflected back by two-body encounters on to a “safer orbit”, i.e., an orbit which does not lead to gravitational capture (Alexander and Hopman 2003) hence even the definition of a loss-cone is not straightforward. Once again, the problem is a compound of the effects of mass segregation, general relativity and resonant relaxation, to mention three main complications. As as result, considerable uncertainties are attached to the (semi-)analytical predictions of capture rates and orbital parameters of EMRIs.

Only self-consistent stellar dynamical modeling of galactic nuclei will provide us with a better understanding of these questions. Some steps in that direction have been made by Freitag (2001, 2003a, b) using Monte Carlo simulations. Later, Freitag et al. (2006a, b) improved upon these results. Yet these studies neglected a direct estimation of EMRIs or “direct plunges”, due in part to the fact that, to follow stars on very eccentric orbits, one needs the combined effects of GW emission and relaxation on timescales much shorter than the capabilities of the numerical Monte Carlo code. Much work remains to be done to confirm these results and improve on them with a more accurate treatment of the physics, to extend them to a larger domain of the parameter space and to more general situations, including non-spherical nuclei.

Classical studies based on approximate stellar dynamics methods that neglect, in particular, the motion of the central MBH and strong 2-body interactions, indicate that, in dense enough clusters, a “seed” MBH (in the IMBH mass range) could swallow a significant fraction of the cluster mass, and thus become a MBH over the span of a few Gyrs (Murphy et al. 1991; Freitag and Benz 2002; Amaro-Seoane et al. 2004). More detailed, higher fidelity *N*-body simulations of relatively small clusters (Baumgardt et al. 2004a, b) have not confirmed this classical result, calling for a critical re-examination and improvement of approximation techniques, the only ones that can cope with the high particle numbers found in massive clusters such as galactic nuclei. It has also been suggested that some processes, such as the effects of chaotic orbits in a slightly non-spherical potential, may effectively keep the loss-cone orbits populated. In this case disruptions and captures can efficiently feed the central MBH and produce the $M-\sigma$ relation (Zhao et al. 2002; Merritt and Poon 2004).

Understanding the astrophysical processes within galacto-centric clusters that give rise to EMRI events has significant bearing on LISA’s applicability to this science. Accurate predictions of the event rate are important for preparing LISA data analysis and design —many events lead to source-confusion, which must be dealt with, while a few events necessitate identifying weak sources in the presence of instrumental noise (Amaro-Seoane et al. 2007). More importantly, LISA observations alone cannot decouple the mass distribution of the galactic black hole population from the mass-dependence of the EMRI rate within a single system. If we can improve our understanding of the latter, we improve LISA’s utility as a probe of the former. In this section I elaborate in detail on the “standard” physics leading to sources of gravitational radiation in the millihertz regime—i.e., in the bandwidth of a LISA-like detector—originating in two-body relaxation processes.

### Fundamentals of EMRIs

In the simplest idealisation, an EMRI consists of a binary of two compact objects, a massive black hole (MBH) and a—typically—stellar black hole (SBH) describing a large number of cycles around the MBH as it approaches the LSO, emitting important, coherent amounts of GWs at every periapsis passage.^11^ After every $2\pi$ around the orbit, the semi-major axis decays a fraction proportional to the energy loss. After typically some $10^{4{-}5}$ cycles, the small body, the CO, plunges through the horizon of the MBH and is lost. The emission of GW finishes. This is what makes this system so attractive. We can regard it as a camera flying around a MBH taking extremely detailed pictures of the space and time around it. With one EMRI we are provided with a set of $\sim 10^{4{-}5}$ pictures from a binary, and the information contained in them will allow us also to know with an unprecedented accuracy in the history of astronomy about the mass of the system, the inclination, the semi-major axis, the spin, to mention some, and it will also be an accurate test of the general theory of relativity.

At first glance the task seems simple and, of course, worth doing; we just have to analyse a binary which decays slowly in time proportionally to $a^4$, where *a* is the semi-major axis. The work seems to be easy for such a big gain. The only problem is that it is not as easy as it seems, because we need to understand how a star can become an EMRI in such a dynamically complex system as a galactic nucleus. Also, the EMRI might suffer perturbations either from gas or from the stellar system (Kocsis et al. 2011; Amaro-Seoane et al. 2012b; Barausse et al. 2014).

In Fig. 40, I show what systems would missions such as LISA be more sensitive to. Obviously, this is only an illustration and the data analysis of the signal will be much more complicated in reality, but it is just an indication already that if the central MBH has a mass larger than $10^7\,M_{\odot }$, then the signal, even at the LSO, will have a frequency too low for detecting the system. On the other hand, if it is less massive than $10^4\,M_{\odot }$, the signal will also be quite weak unless the source is very close. This is why one usually assumes that the mass range of MBHs of interest in the search of EMRIs for LISA is between $[10^4,\,10^7]\,M_{\odot }$. We note that this picture is shifted towards lighter masses in the eLISA configuration, as explained in Amaro-Seoane et al. (2012a, 2013a). Nonetheless, if the MBH is rotating fast, then even if it has a mass larger than $10^7\,M_{\odot }$, the LSO will be closer to the MBH and thus, even at a higher frequency the system should be detectable. This would push to the left the total mass to a few $\sim 10^7\,M_{\odot }$. Indeed, in Fig. 1 of Gair (2009) we can see how the sensitivity varies as we vary the spin of the MBH. The sensitivity limit for non-spinning black holes is about $5\times 10^6\,M_{\odot }$, but this goes up to a few times $10^7\,M_{\odot }$ for prograde inspirals into rapidly spinning black holes. More recently, in Fig. 5 of Babak et al. (2017), we have sky-average horizons for prograde inspirals into maximally spinning black holes. The authors show that we can see inspirals out to $z \sim 1$ even if the MBH has a mass of $10^7\,M_{\odot }$. From the point of view of astrophysics, this range of masses corresponds to low-mass SMBHs. They are not easily detectable and we do not know much about them.

**Fig. 39:**
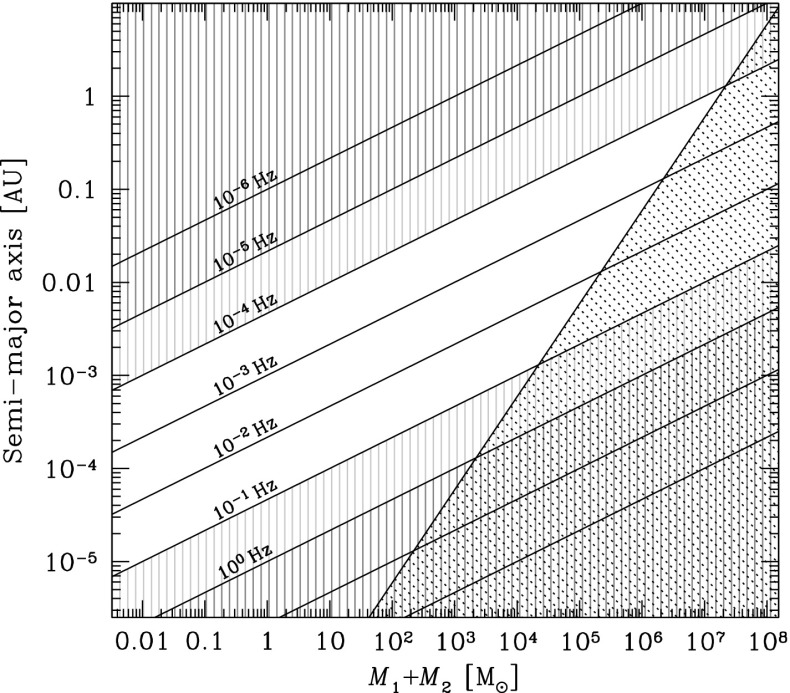
Frequency of a binary of total mass $M_1 + M_2$ against their semi-major axis and the corresponding frequencies. The solid, dark straight line delimits the LSO, so that anything on the right of that line is of no interest for our purposes

A different way of looking at the same picture is Fig. 39. I depict, as a function of the total, non-redshifted mass of the binary $M_1 + M_2$, the semi-major axis of the binary assuming zero eccentricity. We note here that, even if for some particular models, LISA can in principle detect EMRIs out to a redshift of $\sim 4$ (see Babak et al. 2017) most EMRIs will very likely originate from within $z\sim 1$, so that for the rest of this work I neglect it. In Fig. 39 I show the orbital frequency of the binary. Obviously, for the binary to be in the LISA band, it has to have a frequency of roughly—being generous—between 1 and $10^{-5}$ Hz. The emission of GWs is more efficient as they approach the LSO, so that LISA will detect the sources when they are close to the LSO line. For masses larger than $10^7\,M_{\odot }$ the frequencies even close to the LSO will be too low, so that their detection will be very difficult. On the other hand, for a total mass of less than $10^3\,M_{\odot }$ in principal we could detect them at an early stage but then the amplitude of the GWs would be rather low. On top of that, the existence of intermediate-mass black holes is uncertain.

**Fig. 40:**
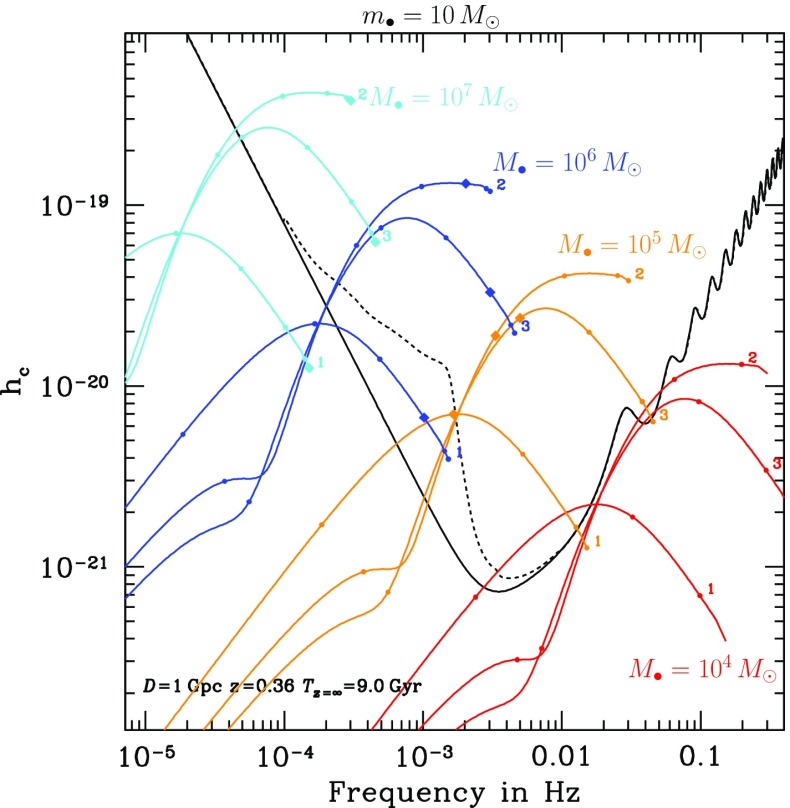
LISA’s sensitivity window and four EMRI signals. The groups of colour correspond to the 1st, 2nd and 3rd harmonic in the quadrupole approximation of Peters (1964) for a SBH of $10\,M_{\odot }$ inspiralling on to a MBH of mass $10^7\,M_{\odot }$ (cyan, left “cascade” of harmonics), $10^6\,M_{\odot }$ (blue, second group from the left), $10^5\,M_{\odot }$ (orange, third cascade) and and $10^4\,M_{\odot }$ (red cascade, first from the right). In each case, the distance to the source has been set up to 1 Gpc

In a spherical potential, at any given time, the stars and compact objects in the nucleus simply orbit the MBH with their semi-major axes and eccentricities changing slowly, owing to 2-body relaxation. For an EMRI to occur, in this standard picture, 2-body relaxation has to bring a compact remnant on to an orbit with such a small periapsis distance that dissipation of energy by emission of GWs becomes significant.

If the object is on a very eccentric orbit but one for which the timescale for passage through periapsis, $t_\mathrm{peri}\simeq (1-e)^{3/2}P$, is less than $\sim 10^4$ s, the source will generate bursts of gravitational radiation in the LISA band each time the object passes through periapsis. However, such GW signals consist of bursts which can probably not benefit from coherent signal processing even if they repeat with a periodicity shorter than LISA mission duration. Only if they reside at the Milky Way centre is there a non-vanishing probability for LISA to detect such sources (Rubbo et al. 2006; Hopman et al. 2007; Berry and Gair 2013). An extra-galactic source is only likely to be detectable if it radiates continuously in the LISA band. As a rough guide, therefore, a detectable EMRI source must have an orbital frequency higher than about $f_\mathrm{LISA}=10^{-4}\,$Hz, corresponding to the condition on the semi-major axis

64 $$\begin{aligned} a\lesssim 0.5\,\mathrm{AU}\, \left( \frac{f_\mathrm{LISA}}{10^{-4}\,\mathrm{Hz}}\right) ^{-2/3} \left( \frac{{\mathscr {M}}_{\bullet }}{{10^6\,M_{\odot }}}\right) ^{1/3}. \end{aligned}$$

As there is no sharp cut-off in the predicted LISA sensitivity curve at $10^{-4}\,$Hz, a strong source might be detectable at a lower frequency.

Not all objects with an inspiral time by GW emission shorter than a Hubble time will end up as EMRIs. This is because, although relaxation can increase the eccentricity of an object to very high values, it can also perturb the orbit back to a more circular one for which GW emission is completely negligible. Typically, neglecting GW emission, it takes a time of the order $t_\mathrm{rlx}\ln (1-e)$ for an orbit to reach a (large) eccentricity *e* through the effects of 2-body relaxation. However, the periapsis distance $R_\mathrm{p}=a(1-e)$ can be significantly altered by relaxation on a timescale $t_\mathrm{rel,p} \simeq (1-e)\,t_\mathrm{rlx}$, so the condition for a star to become an EMRI is that it moves onto an orbit for which the timescale for orbital decay by GW emission, $\tau _\mathrm {GW}$ [see Eq. (73)] is sufficiently shorter than $(1-e)\,t_\mathrm{rlx}$. If the semi-major axis of the orbit is too large, this condition cannot be obeyed unless the star actually finds itself on an unstable, plunging orbit, with $e\ge e_\mathrm{pl}(a) \equiv 1{-}4R_\mathrm{Schw}/a$ where $R_\mathrm{Schw}$ is the Schwarzschild radius of the MBH. The very short burst of gravitational radiation emitted during a plunge through the horizon can only be detected if originating from the Galactic centre (Hopman et al. 2007). Coherent integration of the GW signal for $>10^4$ cycles with a frequency in LISA band is required for detection of extragalactic EMRIs. Therefore a central concern in the determination of EMRI rates is to distinguish between plunges and progressive inspirals (Hils and Bender 1995; Hopman and Alexander 2005).

The situation for EMRI production in the standard picture is more complicated than that of tidal disruptions by the MBH (e.g., Rees 1988; Magorrian and Tremaine 1999; Syer and Ulmer 1999; Wang and Merritt 2004) or GW bursts from stars on very eccentric orbits (Rubbo et al. 2006; Hopman et al. 2007) because these processes require a single passage within a well-defined distance $R_\mathrm{enc}$ from the MBH to be “successful”. In such cases, at any distance from the centre and for any given modulus of the velocity, as mentioned in Sect. 4.4 and later, there exists a “loss cone” inside which the velocity vector of a star has to point for it to pass within $R_\mathrm{enc}$ of the MBH (Frank and Rees 1976; Bahcall and Wolf 1977; Lightman and Shapiro 1977; Amaro-Seoane and Spurzem 2001). In contrast, an EMRI is a progressive process which will only be successful (as a potential source for LISA) if the stellar object experiences a very large number of successive dissipative close encounters with the MBHs (Alexander and Hopman 2003). There is no well-defined loss cone for such a situation.

As described above, a source becomes an EMRI when the orbital period becomes shorter than about $10^4$ s. Even at those distances, the evolution of such a tight orbit could in principle be modified by other stars (Amaro-Seoane et al. 2012b), but based on our current knowledge of nuclei it is an extreme situation, because it requires a second star being very close to the EMRI. It is not so unlikely at earlier stages of the inspiral as 2-body relaxation, experienced mostly at apoapsis, can easily induce a change in the periapsis distance large enough to either render GW emission completely insignificant or, on the contrary, cause a sudden plunge into the MBH (Hils and Bender 1995; Hopman and Alexander 2005).^12^ The condition for a successful inspiral is not that the periapsis distance must be sufficiently small, like for tidal disruptions or GW bursts, but that the timescale for orbit evolution by emission of GWs (see Eq. 73) is sufficiently shorter than the timescale over which 2-body relaxation can affect the periapsis distance significantly,

65 $$\begin{aligned} \tau _\mathrm {GW}< C_\mathrm{EMRI}\, (1-e)\,t_\mathrm {rlx}. \end{aligned}$$

What “sufficiently shorter” means is the main problem and is encoded in $C_\mathrm{EMRI}$, a “safety” numerical constant that makes this condition sufficient ($C_\mathrm{EMRI}<1$). For a given semi-major axis, one can define a critical eccentricity $\tilde{e}(a)$ above which GW emission dominates over orbital evolution due to relaxation and a corresponding time scale

66 $$\begin{aligned} \tilde{\tau }(a)\equiv \tau _\mathrm {GW}(\tilde{e},a)\equiv C_\mathrm{EMRI}(1-\tilde{e})\,t_\mathrm {rlx}\end{aligned}$$

Plunging orbits (for non-rotating MBH, see Sect. 7.6 to understand how this picture changes for Kerr MBH) have

67 $$\begin{aligned} e\ge e_\mathrm{pl}\,(a) \equiv 1-\frac{4R_\mathrm{Schw}}{a}, \end{aligned}$$

so EMRIs (as opposed to direct plunges) can only happen if $e_\mathrm{pl}(a)>\tilde{e}(a)$. This defines a critical semi-major axis which is a typical value for an EMRI at the moment orbital evolution starts being dominated by GW emission,

$$\begin{aligned} a_\mathrm{EMRI} = 5.3\times 10^{-2}\,\mathrm{pc}\,C_\mathrm{EMRI}^{2/3} \times \left( \frac{t_\mathrm {rlx}}{10^9\,\mathrm{yr}}\right) ^{2/3} \left( \frac{m}{10\,M_{\odot }}\right) ^{2/3} \left( \frac{{\mathscr {M}}_{\bullet }}{10^6\,M_{\odot }}\right) ^{-1/3}. \end{aligned}$$

The corresponding eccentricity is given by

68 $$\begin{aligned} 1-e_\mathrm{EMRI} = 7.2\times 10^{-6}\,C_\mathrm{EMRI}^{-2/3} \times \left( \frac{t_\mathrm {rlx}}{10^9\,\mathrm{yr}}\right) ^{-2/3} \times \left( \frac{m}{10\,M_{\odot }}\right) ^{-2/3} \left( \frac{{\mathscr {M}}_{\bullet }}{10^6\,M_{\odot }}\right) ^{4/3}. \end{aligned}$$

The situation is represented in Fig. 43 in the semi-major axis—eccentricity plane. I plot schematically the trajectory for a typical EMRI evolving according to the standard scenario (labelled “1-body inspiral” to distinguish it from the binary tidal separation scenario discussed later). Initially the values of semi-major axis and eccentricity perform a random walk due to 2-body relaxation. As it takes of the order of $t_\mathrm{rlx}$ to change semi-major axis by a factor of 2 but only $(1-e)t_\mathrm{rlx}$ to change the value of $1-e$ (and hence the periapsis), the random walk seems more and more elongated in the horizontal direction, the smaller the value of $1-e$. It is much more likely for a star to cross over to the plunging or GW-dominated region by acquiring a very high eccentricity than by shrinking the semi-major axis significantly. Typically, an EMRI “progenitor” starts with a semi-major axis slightly lower than $a_\mathrm{EMRI}$. It takes on average a time of order $\ln (1-\tilde{e})^{-1} t_\mathrm{rlx} \simeq 10 t_\mathrm{rlx}$ for relaxation to produce an eccentricity such that GW emission becomes dominant. From that point, the object will follow a path closer and closer to a pure inspiral [as approximated by Peters equations (Peters 1964)]. At larger semi-major axis values, inspirals are practically impossible because GW emission is not significant in comparison to relaxation even on plunge orbits. Unless they first shrink their orbit through 2-body relaxation, these objects will be swallowed by the MBH on a direct plunge. Inspirals staring with $a\ll a_\mathrm{EMRI}$ are rare because, for a density cusp $n\propto r^{-\alpha }$ with $\alpha \simeq 1.4{-}1.8$ (Baumgardt et al. 2004a, b; Freitag et al. 2006b; Hopman and Alexander 2006a), the number of stars per unit $\log (a)$ is roughly $\mathrm{d}N_{\star }/\mathrm{d}(\log a)\propto a^{(3-\alpha )}$. Also, as one goes inwards, the value of $\alpha$ is lowered by the progressively larger plunge loss cone (Lightman and Shapiro 1977; Amaro-Seoane et al. 2004). In other words, the stellar density is reduced there (in comparison to a pure power law) because to come and populate this region a star has to spend several relaxation times drifting down in energy while avoiding entering the GW-dominated region and inspiraling quickly.

Implementing this basic scenario in various ways (see Sect. 8.7), several authors have estimated the rate at which stellar remnants are captured by the central MBH, with results between $\sim 10^{-6}\,{\hbox {and}}\,10^{-8}~\mathrm{yr}^{-1}$ for a $10^6\,M_\odot$ central black hole (Hils and Bender 1995; Sigurdsson and Rees 1997; Ivanov 2002; Hopman and Alexander 2005). When combined with the uncertainty in the number density of massive black holes with ${\mathscr {M}}_{\bullet }<\mathrm{few}\times 10^6\,M_\odot$, the net predicted number of detections that LISA can make spans over three orders of magnitude, from a few to a few thousand events per year.

We note, incidentally, that even in the LISA band (in the final year of inspiral), the eccentricity of the typical EMRI in the standard picture is high enough that a large number of harmonics are likely to contribute to the gravitational waves (Freitag 2003b; Barack and Cutler 2004; Hopman and Alexander 2005). In addition, the orbital plane of the EMRIs is unlikely to be significantly correlated with the spin plane of the MBH. These characteristics are distinct from those in non-standard scenarios (discussed below), leading to optimism that some aspects of the nuclear dynamics could be inferred from just a few events.

The word “capture” is sometimes used to refer to EMRIs, but this is misleading as, in the standard picture, stellar objects are not captured by emission of GWs. They are already bound to the MBH when they are brought into the GW-dominated regime by 2-body relaxation. A star originally unbound to the MBH, with energy $\frac{1}{2}v^2$, will be left bound to it by GW emission if it passes with a periapsis distance smaller than

69 $$\begin{aligned} {r_\mathrm{capt}} \approx 5R_\mathrm{Schw}\left( \frac{m}{10\,M_\odot }\right) ^{2/7} \left( \frac{{\mathscr {M}}_{\bullet }}{10^6\,M_\odot }\right) ^{-2/7} \left( \frac{v}{100~\mathrm{km/s}}\right) ^{-4/7}. \end{aligned}$$

In order to become an EMRI (rather than experience a direct plunge), the semi-major axis has to be smaller than a few $10^{-2}\,$pc (see Fig. 43 and Eq. 6.2), requiring a passage within a distance

70 $$\begin{aligned} {r_\mathrm{capt,\,EMRI}} \approx 3R_\mathrm{Schw}\left( \frac{m}{10\,M_\odot }\right) ^{2/7} \left( \frac{{\mathscr {M}}_{\bullet }}{10^6\,M_\odot }\right) ^{-4/7} \left( \frac{a_\mathrm{capt}}{0.05~\mathrm{pc}}\right) ^{2/7}. \end{aligned}$$

Therefore, for masses significantly smaller than $10^6\,M_{\odot }$ there is a possibility of capturing unbound (or loosely bound) stars directly on to EMRI orbits. To my knowledge, the contribution of this channel to EMRI rates has not been estimated in detail but is probably small because it is present only for the lowest-mass MBHs in the LISA range, although we should note that it would be on the “sweet spot” of the LISA configuration (Amaro-Seoane et al. 2012a, 2013a).

### Orbital evolution due to emission of gravitational waves

Consider a binary with component masses $m_1$ and $m_2$, which thus has total mass $M=m_1+m_2$ and reduced mass $\mu =m_1m_2/M$. Suppose that its semi-major axis is *a* and eccentricity is *e*. The Peters equations for gravitational-wave emission from a Keplerian orbit (Peters 1964) give

71 $$\begin{aligned} \left\langle \frac{da}{dt}\right\rangle =-\frac{64}{5}\frac{G^3\mu M^2}{ c^5a^3(1-e^2)^{7/2}} \left( 1+\frac{73}{24}e^2+\frac{37}{96}e^4\right) \end{aligned}$$

and

72 $$\begin{aligned} \left\langle \frac{de}{dt}\right\rangle =-\frac{304}{15}e\frac{G^3\mu M^2}{c^5a^4(1-e^2)^{5/2}} \left( 1+\frac{121}{304}e^2\right) \; . \end{aligned}$$

We note that the Peters formalism does not capture the orbital evolution in the strong-field regime, before plunge. In particular, for EMRIs around a spinning MBH, a slight * increase* in eccentricity might occur in the late evolution (Gair and Glampedakis 2006). This does not affect the present discussion. From Eq. 72, the characteristic time to change the eccentricity is

73 $$\begin{aligned} \tau _\mathrm{GW}= & {} \frac{e}{|de/dt|}\approx \frac{15}{304}\frac{c^5a^4(1-e^2)^{5/2}}{G^3\mu M^2} \nonumber \\&\approx 8\times 10^{17}~\mathrm{yr}\left( \frac{M_\odot }{\mu }\right) \left( \frac{M_\odot }{M}\right) ^2 \left( \frac{a}{1\mathrm{AU}}\right) ^4 \left( 1-e^2\right) ^{5/2}. \end{aligned}$$

Here, I neglect the near-unity factor $(1+121e^2/304)$.

We can rewrite this in terms of gravitational-wave frequency. Let us consider in particular the frequency emitted at periapsis. If the orbit is substantially eccentric, then the orbital frequency at that point will be approximately $\sqrt{2}$ times the circular frequency at that radius (because the speed is $\sqrt{2}$ times greater than a circular orbit). If we dictate a maximum gravitational-wave frequency $f_{\max }$ to be double the frequency at periapsis, then

74 $$\begin{aligned} f_{\max }\approx {1\over \pi }\left[ 2GM\over {(a(1-e))^3}\right] ^{1/2}\; . \end{aligned}$$

Therefore

75 $$\begin{aligned} a^4= 0.75\mathrm{AU}^4\left( \frac{M}{10^6\,M_\odot }\right) ^{4/3}\left( \frac{f_{\max }}{10^{-4}~\mathrm{Hz}}\right) ^{-8/3} (1-e)^{-4}, \end{aligned}$$

and

76 $$\begin{aligned} \tau _\mathrm{GW}&\approx 6\times 10^2~\mathrm{yr}\left( \frac{\mu }{10^3\,M_\odot }\right) ^{-1}\left( \frac{M}{10^6\,M_\odot }\right) ^{-2/3} \left( \frac{f_{\max }}{10^{-4}~\mathrm{Hz}}\right) ^{-8/3}(1+e)^{5/2}(1-e)^{-3/2}\nonumber \\&\approx 3\times 10^3~\mathrm{yr}\left( \frac{\mu }{10^3\,M_\odot }\right) ^{-1}\left( \frac{M}{10^6\,M_\odot }\right) ^{-2/3} \left( \frac{f_{\max }}{10^{-4}~\mathrm{Hz}}\right) ^{-8/3}(1-e)^{-3/2} \end{aligned}$$

where in the last line I assume a relatively high eccentricity, so that $1+e\approx 2$.

A classic EMRI, with $M=10^4{-}10^7\,M_\odot$ and $\mu =1{-}10\,M_\odot$, could have a significant eccentricity if (as expected in galactic nuclei) the orbits come in from large distances, $a>10^{-2}\,$ pc with $e\gtrsim 0.9999$. Hopman and Alexander (2005) made an estimate of the distribution of eccentricities for one body inspiral and their results showed that it is skewed to high-e values, with a peak of the distribution at $e \sim 0.7$, at an orbital period of $10^4$ s. On the other hand, following a binary separation event (and possibly the tidal capture of giant’s core), the compact star is deposited on an orbit with semi-major axis of order a few tens to a few hundreds of AU. In this case, the GW-dominated regime is reached with an eccentricity smaller than 0.99 and the orbit should be very close to circular when it has shrunk into the LISA band. Such typical orbital evolutions for EMRIs are shown in Fig. 43.

### Decoupling from dynamics into the relativistic regime

In the late stage of the inspiral, a binary may become a detectable source of GWs. The characteristic amplitude of the gravitational radiation from a source emitting at frequency *f* is

77 $$\begin{aligned} h_\mathrm{c} = \frac{(2\dot{E}/\dot{f})^{1/2}}{\pi D} \end{aligned}$$

where *D* is the distance to the source, $\dot{E}$ is the power emitted and $\dot{f}$ the time derivative of the frequency (Finn and Thorne 2000). With this definition, the signal-to-noise ratio (SNR) of an event is obtained, assuming ideal signal processing, by the integral^13^

78 $$\begin{aligned} (\mathrm{SNR})^2=\int _{f_1}^{f_2}\frac{h^2_\mathrm{c}(f)}{f\,S_h(f)}\,d(\ln f) \end{aligned}$$

where $f_1$ and $f_2$ are the initial and final frequencies of the source during the observation and $S_h(f)$ is the instrumental noise of the detector at frequency *f* (Phinney 2002; Barack and Cutler 2004).

**Fig. 41:**
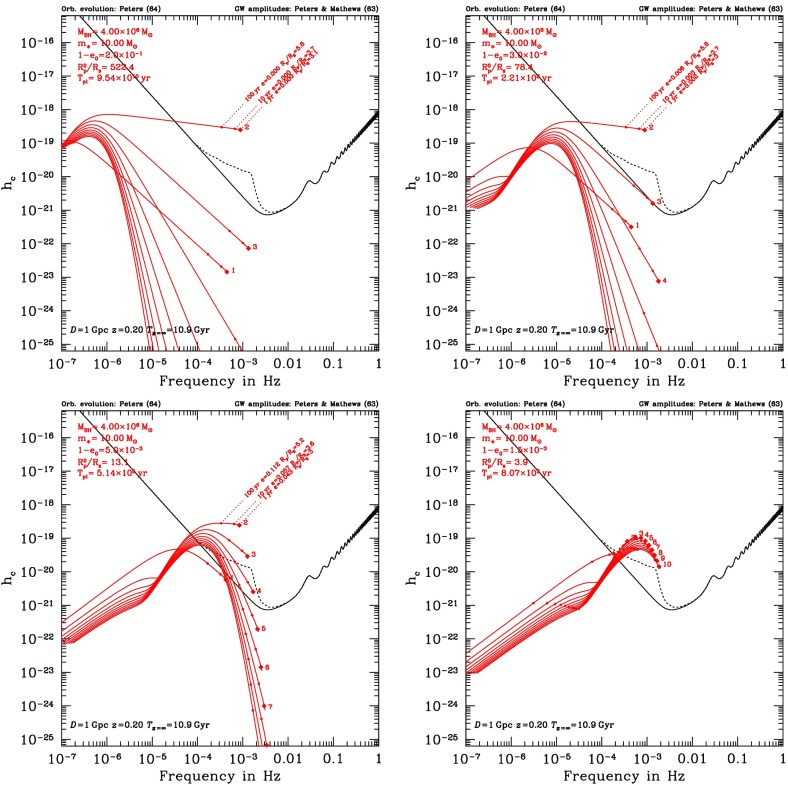
Characteristic amplitude, introduced in Eq. (77), of the first harmonics of the quadrupolar gravitational radiation emitted during the inspiral of a stellar-mass BH of $m_{\bullet }=10\,M_{\odot }$ ($m_*$ in the plot) into a MBH of mass ${\mathscr {M}}_{\bullet }=4\times 10^{6}$ ($\mathrm M_{BH}$ in the plot). I assume the source is at a distance $D=1\,$Gpc. I indicate the noise curve $\sqrt{f\,S_h(f)}$ for a LISA-like detector (Larson et al. 2000; Larson 2003), with the Galactic binary white dwarf confusion background in dashed line (Bender and Hils 1997). Note that the height of the point for the amplitude above the curve does *not* represent the SNR (see text). From the top to the bottom and from left to right, the panels represent a binary which starts at a semi-major axis of $10^{-3}$ pc and we change the eccentricity, $e=0.8,\,0.97,\,0.995,\,0.9985$. Each panel shows the ratio $R_\mathrm{p}^0/R_\mathrm{s}$, the initial periapsis distance over the Schwarzschild radius of the system. The first three panels display three moments in the evolution for which the time to coalescence in the is 100, 10 and 1 yr

In Fig. 41, I follow the signal emitted by a binary consisting of a Milky Way-like MBH and a stellar BH during their GW-driven inspiral without taking into account any possible dynamical interaction; i.e. we only allow the system to evolve via gravitational radiation emission. I plot the five lowest harmonics of the quadrupolar emission in a rough approximation (Peters and Mathews 1963), only useful for illustrative purposes. In this figure, I assume a distance of 1 Gpc.

For low-eccentric captures only the $n=2$ harmonic is detectable, during the last few years of inspiral. However, the small residual eccentricity induces a difference in the phase evolution of the $n=2$ signal compared to a perfect circular inspiral (Amaro-Seoane and Freitag 2006). If the source is followed from a time $\tau _\mathrm{LSO}$ before merger until merger, the accumulated phase shift is

79 $$\begin{aligned} \varDelta \psi _e \simeq \left( \frac{e_{10^{-4}\mathrm Hz}}{0.05}\right) ^2 \left( \frac{\tau _\mathrm{LSO}}{1\,\mathrm yr}\right) ^{17/12} \left( \frac{{\mathscr {M}}_z}{10^3\,M_{\odot }}\right) ^{25/36}, \end{aligned}$$

where $e_{10^{-4}\mathrm Hz}$ is the eccentricity when the $n=2$ signal has reached a frequency of $10^{-4}$ Hz and

80 $$\begin{aligned} {\mathscr {M}}_z \equiv (1+z)\frac{({\mathscr {M}}_{\bullet } m_{\bullet })^{3/5}}{({\mathscr {M}}_{\bullet }+m_{\bullet })^{1/5}} \end{aligned}$$

is the redshifted chirp mass of the binary (Cutler and Harms 2006). This means that in principle we can easily distinguish between high-eccentricity captures and low-eccentricity captures. The implications of this result will become clear in the next sections.

Figure 43 displays the last stable orbit in the effective Keplerian approximation ($R_\mathrm{p}\simeq 4\,R_\mathrm{Schw}$ for $e\ll 0.1$, see Cutler et al. (1994) with a solid, thick diagonal line. The thin dotted blue lines are contours of constant time left until the final coalescence, $T_\mathrm{GW}$ in the Peters (1964) approximation. The years are show on the right. The thin diagonal green lines are the inspiral, capture orbits due * only* to the emission of GWs. The upper dash-dotted red line shows $\tilde{e}(a)$, defined by $t_e=T_\mathrm{GW}$ [Eq. (65) with $C_\mathrm{EMRI}=1$] assuming a constant value $t_\mathrm{rlx}=1$ Gyr. The lower dash-dotted red lines depict the same threshold times a factor 10, 100, 1000, 10,000 and 100,000. On the right-hand side of these lines the evolution of the binary is driven mainly by relaxation, GW emission is totally negligible and vice-versa; i.e., on the left-hand side the evolution is led by the loss of energy in GWs. An interesting point is the intersection of the first of these red lines (the uppermost one) with the last stable orbit line. This is the transition between the so-called direct plunges and the EMRIs.

The thick, dashed black line shows the tidal disruption radius. Any extended star fording that radius will be torn apart by tidal forces of the MBH, which we assume to have a mass ${\mathscr {M}}_{\bullet } = 4 \times 10^6\,M_{\odot }$ ($\mathrm{M}_{\mathrm{MBH}}$ in the plot). Then, as an illustration, I depict the trajectory of a $10\,M_{\odot }$ stellar BH ($m_{bh}$ in the plot) inspiralling into the MBH. We can separate two kind of sources according to their astrophysical origin; namely low-eccentricity captures, stars captured by tidal binary separation, and high-eccentricity captures, stemming from “simple” two-body relaxation. The latter initially have semi-major axis values of order 100–1000 AU [$5\times (10^{-4}{-}10^{-3})\,$pc] and $e=0.9{-}0.99$ (Miller et al. 2005). The evolution of the eccentricity is a random walk leading to nearly-circular orbits after a timescale of about $T_\mathrm{rlx}\ln (1-\tilde{e})^{-1}$. The latter correspond to stars on capture orbits due to diffusion form large radii or capture by GW emission and have initially have a much larger value of semi-major axis and hence a higher eccentricity. If a star has a semi-major axis $\gtrsim 5\times 10^{-2}$ pc, it will not reach small orbital periods, i.e., it will not enter a millihertz detector such as LISA unless the semi-major axis is reduced considerably, which in the context of “normal” relaxation theory, takes about a time $t_\mathrm{rlx}$.

**Fig. 42:**
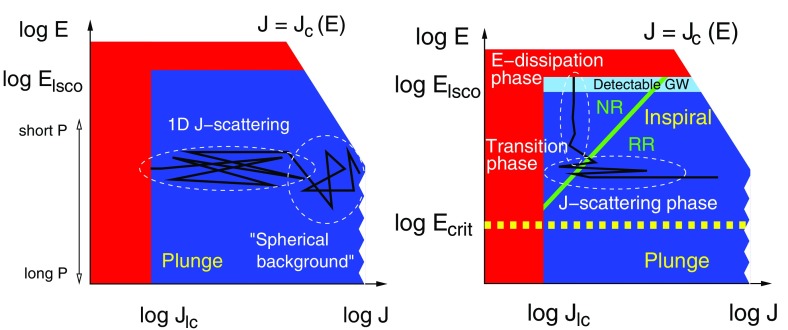
Plunge, inspiral and critical energy of an EMRI in phase-space. *Left panel:* Direct plunge of a source. *Right panel:* Adiabatic inspiral of a source subject to dissipation of energy at periapsis and limiting critical energy (image courtesy of Tal Alexander)

A different way of looking at the same picture is by displaying the energy and angular momentum of the system. Working in terms of energy and angular momentum has advantages that can be important to understand some very subtle phenomena that possibly play a major role in the process of capturing stars. We can see this in Fig. 42 (courtesy of Tal Alexander): To get close to the central MBH, it is faster to relax angular momentum than to relax energy. Let us assume that we do not have any dissipation mechanism. Figure 42 depicts the phase-space of the system in terms of energy and angular momentum and I use the convention that energy is defined with a negative sign, so that high positive values of energy mean that the star is very close to the MBH. The red region represents the zone where the star cannot exist, i.e., we are closer to the MBH than the LSO. The upper right diagonal line expresses the fact that for a value of energy you can only have up to some maximum value of $J_c$, the angular momentum of a circular orbit. Our test star, a compact remnant, will suffer gravitational tugs whenever it is far away from the energy and angular momentum edges. These tugs are random and originate from interactions with other stars that happen to have a very close position in phase-space and the scattering rate is very similar in both directions. This means that the time spent in one of the horizontal segments is approximately the same as the time spent in one of the vertical segments in the zig-zag trajectory displayed in the figure:

81 $$\begin{aligned} t_J \sim \left( \frac{J}{J_c(E)}\right) ^2\,t_E \sim t_E; \end{aligned}$$

i.e., the timescale to change angular momentum, $t_J$ is approximately the same as the timescale to change energy, $t_E$. This means that if every zig-zag represents a change over a fixed amount of time, say $10^9$ yrs, the star will travel approximately the same distance in one or the other way. If the star gets close to a very low angular momentum, which is statistically probable, then the picture changes: the rate at which the star will change angular momentum will be much shorter than the rate at which it changes energy. The star moves approximately in phase-space in one dimension, horizontally in the figure. If we wait long enough the star will eventually enter the loss-cone and “plunge” on to the central MBH. That is, the source of GWs is lost after a few periapsis passages, a few intense GW bursts and is not as interesting as a gradual, slowly inspiraling source. This picture corresponds to the general scenario that was described already a few decades ago, when people were investigating ways of feeding the MBH (Lightman and Shapiro 1977; Cohn and Kulsrud 1978).

However, if we have a dissipation process acting on to the star, which could be energy loss in the form of GWs as well as drag forces originating in an accretion disc or, obviously tidal forces created by the central MBH, the picture changes significantly. The process follows the same path and, at some point, the star reaches the region in which it is on a very radial orbit, i.e. where the zig-zag stops and we can approximate the curve by a horizontal line. Nonetheless, in this case, at every periapsis passage, the star will emit an intense burst of GWs and, thus, shrink its semi-major axis. If this happens “efficiently enough”, i.e., “fast enough” (we will elaborate on this later), the star is more and more bound to the central MBH and drifts away (goes up in the energy axis of the figure). The danger of being scattered away from the capture orbit by other stars decreases more and more and the compact object finds itself on a safe inspiraling EMRI orbit. The precise details of the dynamics that lead to this situation determines the distribution of eccentricities that we can expect. The semi-major axis shrinks to the point that the source enters the “Detectable GW” regime (light-blue band in the right panel of Fig. 42). As the source advances in that band, the period becomes shorter and shorter and, hence, the power (emitted energy per unit of time) grows larger and larger, so that the gravitational radiation can be measured when it enters the frequency band of the observatory.

The *statistical* orbital properties of the EMRI in the region where GW emission is prominent are fully determined by the transition phase between the region dominated by 2-body scattering processes (the right part of the curve) of the random walk in phase-space and the *deterministic* dissipation part of the capture trajectory, i.e., where the energy loss occurs.

As described in Hopman and Alexander (2005), in this statistical treatment there is a critical energy, i.e., a certain distance from the central MBH, of the order $\sim 10^{-2}$ pc, that can be envisaged as the threshold between the two regions. This means that stars with energy below the yellow dashed line of the right panel of Fig. 42 will have “longer horizontal segments”, they will scatter faster in angular momentum than in energy and then they will end up as direct plunges. They approach the central MBH in such a radial orbit that they are swallowed after one or, at most, a few intense bursts of GWs. This situation is reverted if the energy of the star is above the line; the star will spiral in adiabatically and it will not be perturbed out of the EMRI trajectory, with a significant amount of GW bursts at periapsis before coalescing with the MBH.

Hence, and again, statistically at first order, one has to consider only stars whose energy falls within the critical region and we can ignore all other stars, even if their energy and angular momentum indicate that they are good candidates for EMRIs. Thus, *the event rate will be determined by the “microphysics” affecting the innermost volume around the MBH, of radius* $\sim 10^{-2}$ *pc*. As the reader will surely have guessed by now, the task is non-trivial.

**Fig. 43:**
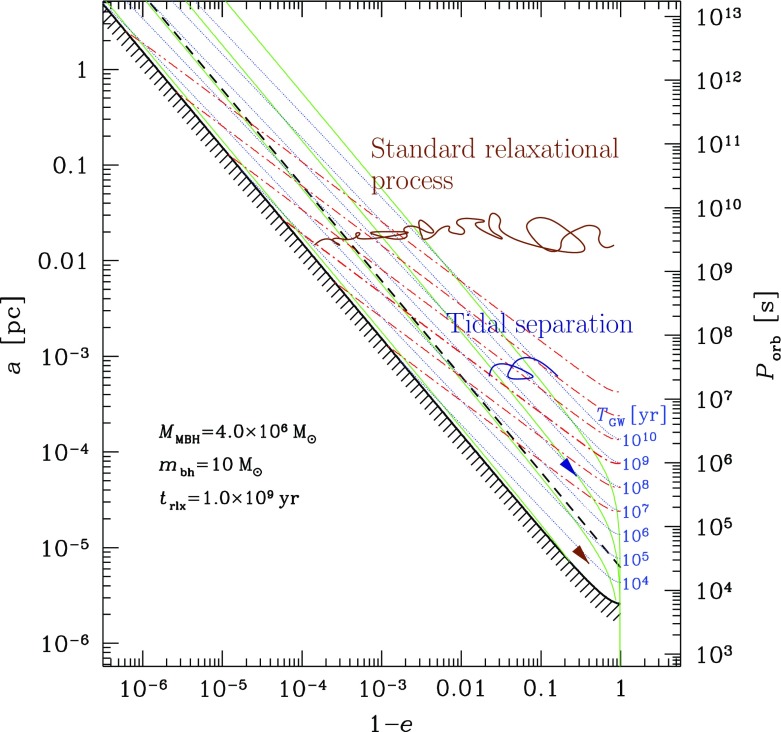
Capture trajectories in the *a* – $(1-e)$ plane and tidal disruption limit. See text for a detailed explanation of the figure

## Beyond the standard model of two-body relaxation

### The standard picture

The intelligent reader will very surely have realised that the picture is much more complex than plain two-body relaxation. Quoting something that Sterling Colgate said once in Aspen,

*Do you know what the standard (American) model is? : One gallon per flush.*

Although Sterling was not directly referring to our standard model, of course. This means that, illustrating and enlightening as it might be, the standard model we have been describing so far must be regarded as a (probably very well) educated guess.

As the interest in a millihertz mission started to grow and develop, astrophysicists started to dedicate more and more time to a problem that, naively, was not very difficult. How do you get a small black hole into a massive black hole in a galactic nucleus? Now, some decades after the very first estimates, we have a much better and clear vision of the main phenomena at play in the process. Well before any space-borne mission is launched, our understanding of theory related to stellar dynamics has become much broader and new, unexpected effects have emerged.

### Coherent or resonant relaxation^14^

As I have discussed previously, in a gravitational potential with a high degree of symmetry, a test star will receive gravitational tugs from the rest of the field stars which are not totally arbitrary and hence do not add up in a random walk way, but *coherently*. As we have seen in Sect. 3, the potential will prevent stellar orbits from evolving in an erratic way. In a two-body Keplerian system, a SBH will orbit around the MBH in a fixed ellipse. The stellar BH will not feel random gravitational tugs. It evolves coherently as the result of the action of the gravitational potential. When an EMRI approaches the periapsis of its orbit, we can envisage the situation as a pure two-body problem; initially Newtonian but later GR effects must be taken into account as the periapsis grows smaller and smaller. Nonetheless, as the stellar BH goes back to the apoapsis, it will feel the surrounding stellar system, distributed in the shape of a cusp which grows in mass the further away we are from the periapsis. The time spent in the region in which we can regard this as a two-body problem is much shorter than the time in which the stellar BH will feel the rest of the stellar system. This is particularly true for the kind of objects of our interest, since the very high eccentricity implies a large semi-major axis. The time spent on periapsis is negligible as compared with the time spent on apoapsis, so that the stellar BH can feel the graininess of the potential. The gravitational tugs from other stars will alter its orbit. The mean free path in angular momentum-space of that test stellar BH is very large and thus, it has a *fast* random walk. Both the magnitude and direction of angular momentum of the stellar black hole are altered. When the magnitude changes but not the direction, we talk of “scalar” resonant relaxation, and correspondingly when the direction is changed but not the size, “vector” resonant relaxation.

A very radial orbit can become a very eccentric one, so that a compact object initially set on a potential EMRI orbit can be “pushed out” of it. In a more general case, a spherical potential that is non-Keplerian, the orbits, as we have described before, are rosettes and averaged over time they are circular anuli. In that case we can change the direction of angular momentum but not the modulus. An eccentric orbit will stay eccentric, but any coherence that was there will be washed out.

**Fig. 44:**
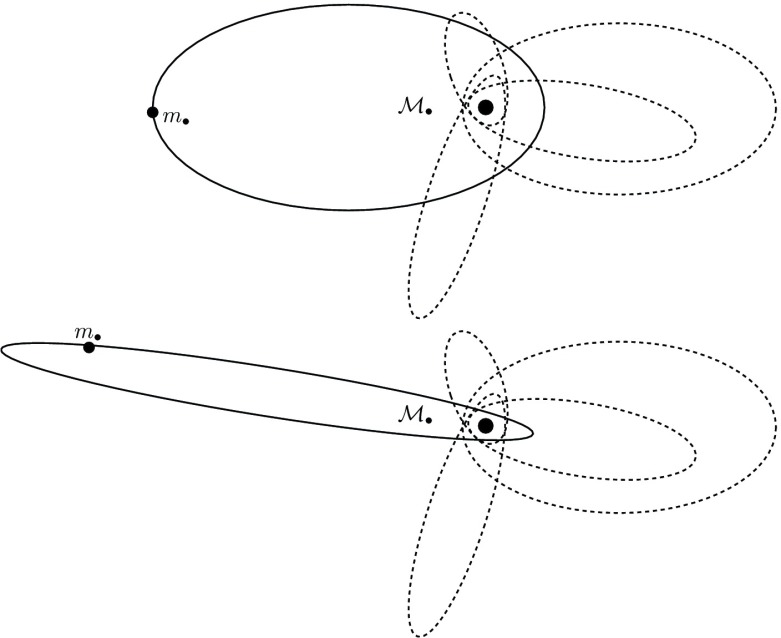
If we have a point-like potential, scalar coherent relaxation can modify the size of angular momentum and the inclination of the orbital plane of a test star. Dashed lines depict the perturbing orbits on the test star, $m_{\bullet }$, whose orbit is displayed as a solid line in two moments of the evolution

In particular, as illustrated in Fig. 44, in the potential of a point mass, orbits are frozen fixed ellipses that exert a continuous torque on the test star. A test star does not feel random kicks from all directions. When we add up the individual contributions coming from all the rest of stars on to the test star, there is a residual, non-negligible torque that will influence its evolution. The mean free path of the star in angular momentum space is very large. I will refer to this phenomenon as *scalar* coherent relaxation, because it can change both the magnitude of angular momentum and the inclination of the orbital plane of the test star. This scenario is a possible way to alter an initially very circular orbit and modify it in such a way that the test star will get very close to the MBH after the torques have acted. That is, we open a new window for stars to fall into a capture orbit that will lead to an EMRI.

In a more general case, if we have a potential that is simply spherical but not necessarily Keplerian (a point mass), the field stars, the perturbing orbits to the test star, describe rosettes -as we have seen- and averaged over time they can be approximated by a set of anuli that share a centre. From a secular point of view, the masses are smeared over those anuli which create torques that do not change the magnitude of angular momentum but they do change the orientation because of reasons of symmetry (Rauch and Tremaine 1996; Rauch and Ingalls 1998; Hopman and Alexander 2006b). Hence a circular test star will keep a negligible eccentricity and it will *not* approach the central MBH. Any coherence that was present in the system will nonetheless be destroyed. I will refer to this as *vectorial* coherent relaxation. From the standpoint of EMRI production, though, this process is not as relevant and we will not elaborate on it further, though it can be very relevant for phenomena related to galactic nuclei, for instance, warping of accretion discs (Bregman and Alexander 2009).

However, one must note that these illustrations are oversimplifications and depict perfect symmetries that might be affected or even totally cancelled out by other effects such as, e.g., the relativistic periapsis shift or Newtonian precession. Thus, after a certain time this symmetry is broken and the evolution is again a random walk, one with very large stepsize. I refer the reader to the review of Alexander (2007) for a detailed and excellent description of these processes.

**Fig. 45:**
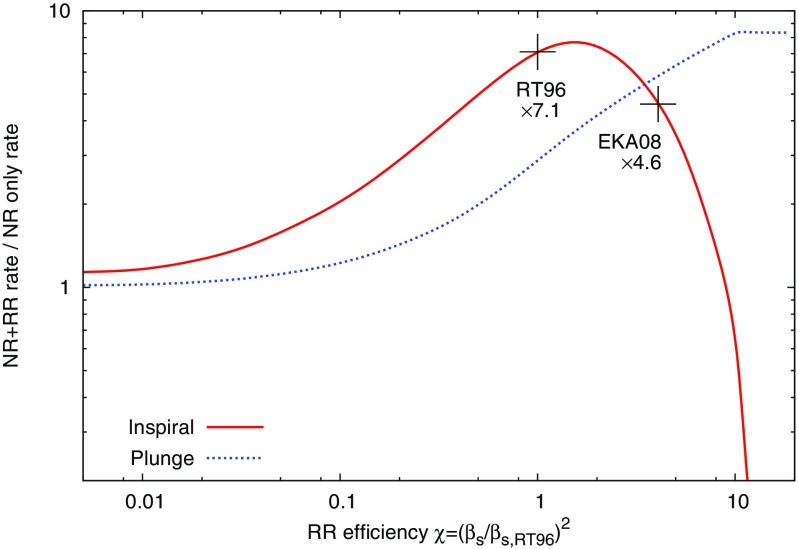
Total event rates produced by relaxation and coherent relaxation normalised by the rates obtained by considering only relaxation. Image reproduced with permission from Eilon et al. (2009), copyright by AAS

The impact of coherent relaxation on the production of EMRIs is important. While the underlying physics of the process is very robust, it is a rather difficult task to ponder the efficiency of the different parameters involved in the process. A possible way of evaluating it is given by Hopman and Alexander (2006b) and Eilon et al. (2009). Figure 45, which is Fig. 6 of Eilon et al. (2009), shows the rate of EMRIs and plunges in a system in which we take into account both orthodox or regular relaxation and coherent relaxation normalised to what one can expect when only taking into account normal relaxation as function of the $\varXi$ parameter, which gives us the efficiency of coherent relaxation. The units of $\varXi$ are such that the value suggested in Rauch and Tremaine (1996) is unity. We note that the work of these authors was limited to a very low number of particles, but we can consider it as a reference point to refer to. Thus, if coherent relaxation is more efficient than what they found, $\varXi >1$ and vice-versa, i.e., we approach the regime in which there is not coherent relaxation. It is very remarkable to see that by choosing the value suggested (Rauch and Tremaine 1996), we achieve the maximum of the EMRI rate curve. If the “real” value of $\varXi$ happened to be a factor 10 larger, then we would be drastically dropping the rates and increasing the direct plunges and, of course, also the tidal disruptions event rate, since these occur at larger radii.

**Fig. 46:**
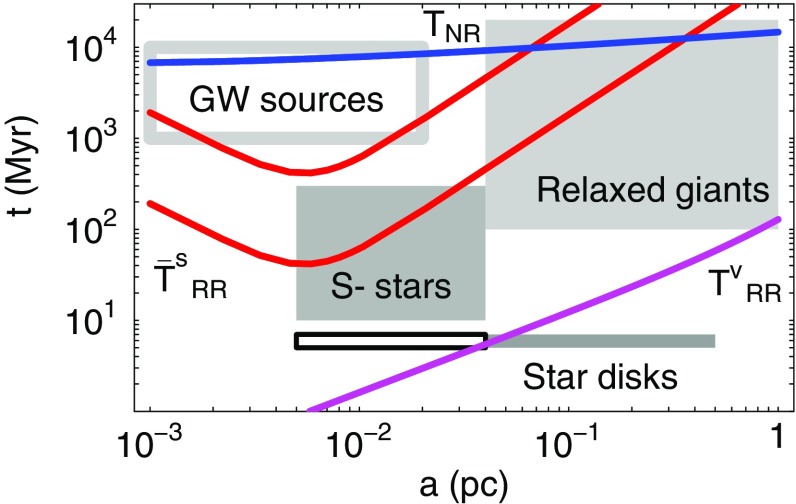
The different timescales dominating stellar dynamics in the Galactic Centre. Image reproduced with permission from Hopman and Alexander (2006b), copyright by AAS

At first glimpse, everything seems to boil down to calculating the precession of coherent relaxation. One obvious way is to do large-particle number simulations, since the first attempt of Rauch and Tremaine (1996) was really *very* limited and difficult to interpret (they were using fewer than 100 particles). However, the systems we are trying to simulate are much more complicated than something a simplified approach will be able to investigate. From a numerical point of view the complications are big and non-negligible. Nevertheless, there has been an important and impressive advance in this front recently but, before we address it, the results and interpretations, it is probably better to have a look at a very familiar system for us, Sgr A*. Hopman and Alexander (2006b) have done this interesting and useful exercise, which is summarised in their Fig. 6 (see Fig. 46). In this figure, the authors display the relevance of different dynamical components in an attempt to constrain the strength of coherent relaxation.

On the vertical axes, we have the age of different systems found in the GC as function of the semi-major axis of the stars with the object in Sgr A*. On the top of the figure we see a line giving us the timescale for normal relaxation, $T_\mathrm{NR}$ to use the same nomenclature as the authors and their plot, which is shorter than the Hubble time but not much shorter. The following two curves from the top give the timescale for scalar coherent relaxation for two cases, the first curve from the top corresponds to a system of $1\,M_{\odot }$ stars and $10\,M_{\odot }$ stars at large values the effect is quenched by the presence of an extended mass, i.e., Newtonian precession and at short distances it is periapsis shift that decreases its strength. The minima displayed in the figure fence in the potential range of values for the efficiency. The “real” value probably lies somewhere in the middle.

It is, nevertheless, important to note that the authors did not take into account the effect of a mass spectrum. In this respect, while it is easier to understand the fundamentals of the scenario, the system lacks an important ingredient in realism that could significantly change the narrative.

On the lower right corner of the figure, we have vector coherent relaxation, which is much more efficient with associated timescales shorter than a million years for a short enough semi-major axis.

In the same figure, we display the area from which we believe that EMRIs originate; i.e., within $\sim 0.01$ pc. These objects are typically compact remnants and, hence, will be accumulated in the top left corner of the figure because they are older than the typical time for relaxation. As we can see, and as shown in the calculations of the authors, they are embedded in the area which is totally dominated by coherent relaxation. This is a very striking result from the standpoint of standard relaxation theory: The dynamics of EMRIs will be dominated by this new “exotic” form of relaxation, coherent relaxation and not by normal (two-body) relaxation.

As I have already explained previously, there are different populations of stars in the GC that we can observe. One of these is the disc stars, some $\sim$ 50–100 very massive and young stars observed to be orbiting on discs and almost circularly. The upper limit on the edge is of a few $10^6\,M_{\odot }$ and, thus, the strip in the figure is very narrow. These discs are characterised by having a relatively well-defined and sharp inner cut-off. It is remarkable to note that the cut-off happens to be exactly at the place in the figure where the timescale associated with vectorial coherent relaxation ($T^\mathrm{V}_\mathrm{RR}$ in the plot) crosses the strip, without a fit, as Hopman and Alexander (2006b) claim. On the left side of the line, we have the S-stars, which are *not* on circular orbits, nor aligned with the disc, but randomly orientated. They are sometimes envisaged as the low-mass members of the disc of stars. In any case, it is intriguing that these stars lie exactly on the left of the curve, where we expect any disc structure to be destroyed by vectorial coherent relaxation. This would imply that the values derived by Rauch and Tremaine (1996) are very close to the real ones. While it is probably too early to make any strong statement from this fact, it is encouraging enough to keep us studying and trying to understand normal as well as coherent relaxation in galactic nuclei. Another interpretation of Fig. 46 is that we can expect some of the S-stars to have random eccentricities due to the fact that those which are close enough are affected by scalar coherent relaxation. Also, we can in principle explain why late-type giants do not have any particular orientation in their orbits, since they are in that part of the plot.

The numerical simulations of Eilon et al. (2009) show that coherent relaxation can enhance the EMRI rate by a factor of a few over the rates predicted assuming only slow stochastic two-body relaxation, as the authors prove.

### Strong mass segregation

We have seen in Sect. 5 that stars with different mass get distributed around a MBH in a galactic nucleus with different density profile. We devoted a significant part of that section to studying the case of single-mass, which was described in an analytical way by Bahcall and Wolf (1976), and previously in Peebles (1972). The authors extended the work to stellar systems with two mass components and argued heuristically for a scaling relation that depends on the star’s mass ratio only, namely $p_L = m_L/m_H \times p_H$ (Bahcall and Wolf 1977). They did not give a general result on inner slope of the heavy stars (the stellar-mass black holes in our case) and they did not discuss the dependence of the result on the component’s number fractions. Fortunately, Alexander and Hopman (2009) addressed this issue and found that there exist two branches of solutions, parametrised by

82 $$\begin{aligned} \varDelta \approx \frac{N_H m_H^2}{N_Lm_L^2} \frac{4}{3+m_H/m_L}, \end{aligned}$$

where capital letters denote total stellar mass and lower-case letters individual masses of stars. The quantity $\varDelta$ gives us a measure of the relevance of the SBH self-coupling relative to the other species coupling, the lighter stars in the system, and the main advantage of it is that it depends basically on the mass and number ratios. In this respect, Alexander and Hopman (2009) extend the study of Bahcall and Wolf (1977) to an additional, crucial parameter. For values of $\varDelta > 1$ we recover the scaling solutions of Bahcall and Wolf (1977). This is the regime that Alexander and Hopman (2009) refer to as the “weak branch” of the solution. On the other hand, for $\varDelta < 1$, we have a new kind of solution that generalises the solution of Bahcall and Wolf (1977). This is the “strong mass segregation” regime, because the density slopes that one obtains in this case are steeper.

Inspired by their work, Preto and Amaro-Seoane (2010) and Amaro-Seoane and Preto (2011) used direct-summation simulations as a calibration to Fokker–Planck experiments that allowed them to explore this new solution. This is a priori not obvious, since we are in a regime in which scattering is dominated by uncorrelated, 2-body, encounters and dense stellar cusps are robust against ejections. The authors proved that the agreement between both methods is quite good.

### The cusp at the Galactic Centre

**Fig. 47:**
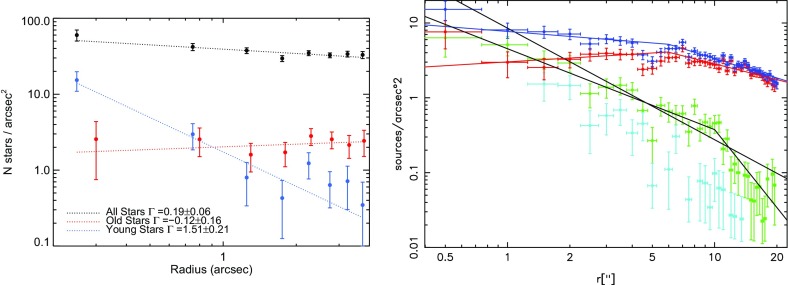
Number of sources per arcsec$^2$ as a function of radius from the Galactic Centre in seconds. Images reproduced with permission from (left) Do et al. (2009), copyright by AAS; and (right) Buchholz et al. (2009), copyright by ESO

The implications of these results are interesting and important for EMRI science, but also particularly timely. This is so because of recent progress in electromagnetic observations of our Galactic Centre. Some years ago, two independent groups have observed that there seems to be a deficit of old stars based on number counts of spectroscopically identified, old stars in a sub-parsec region around Sgr A* (down to magnitude $K=15.5$, see Buchholz et al. 2009 and Do et al. 2009). In Fig. 47, we show their main results. The best fits seem to favour slopes $\gamma < 1$ and the possibility of a core with the stellar density decreasing, $\gamma < 0$ is not excluded (Buchholz et al. 2009; Do et al. 2009; Bartko et al. 2010). One must take into account that detectable stars are essentially giants and they represent only a very small percentage of the underlying population, and the slope of the density profile is still weakly constrained and such a fit is only marginally better than one with $\gamma \sim 1/2$.

**Fig. 48:**
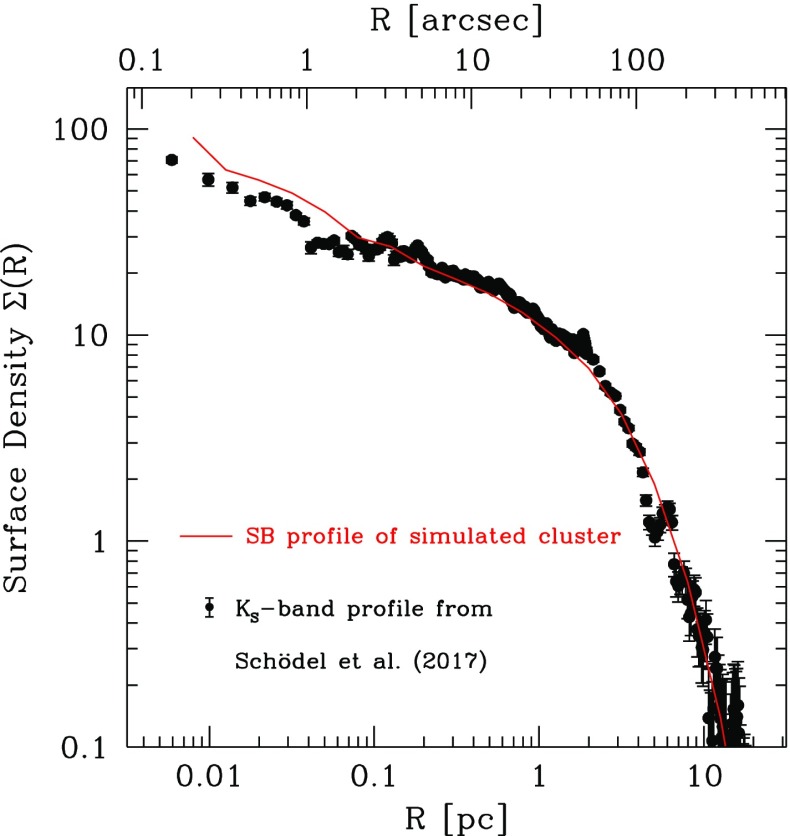
Comparison of the observational data of the surface luminosity profile of the diffuse light of the galactic centre by Schödel et al. (2018) with a direct-summation *N*-body simulation (red line). Image reproduced by permission from Baumgardt et al. (2018), copyright by ESO

Indeed, the work by Gallego-Cano et al. (2018), Schödel et al. (2018) suggests that the observational data of the Galactic Centre had to be re-analysed. They show that the red- and brighter giants display a core-like surface density profile within a projected radius of $R<0.3$ pc of the central MBH, in agreement with previous studies, but show a cusp-like surface density distribution at larger radii. The authors conclude that the observed stellar density at the Galactic Centre is consistent with the existence of a stellar cusp around the Milky Way’s MBH, and that it is well developed inside its influence radius. It is remarkable that this observational study agrees very well with the numerical work of Baumgardt et al. (2018). The authors of the paper ran a series of direct-summation *N*-body simulations of the Galactic Centre and found that the distribution of stars is what one might expect from usual two-body relaxation, without the need of invoking exotic phenomena. The comparison between the numerical simulation and the observational data is shown in Fig. 48.

The apparent lack of stars at projected distances of $R < 0.3$ pc can be explained in the theoretical framework of Amaro-Seoane and Chen (2014), Chen and Amaro-Seoane (2014): The fragmenting past of the stellar disc we observe now in our Galactic Centre would have been responsible for the apparent absence of bright giants. They would have lost their envelopes by interacting with the high-density clumps that formed in the fragmenting disc.

**Fig. 49:**
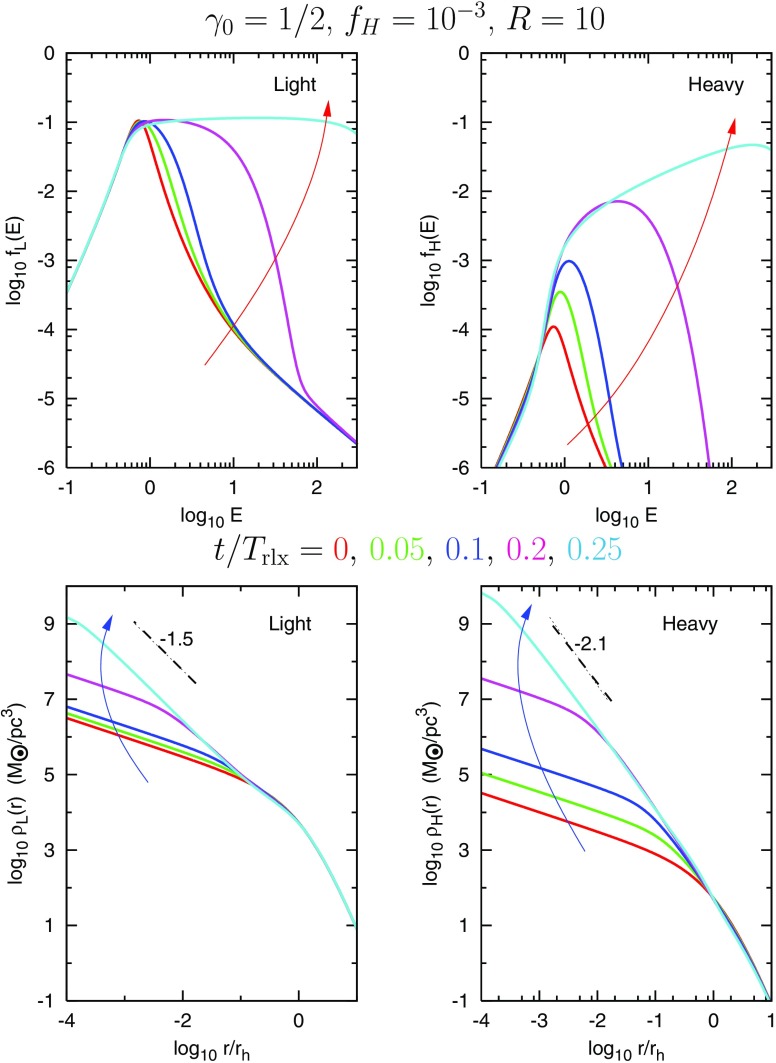
*Upper panels:* Evolution of the phase-space *f*(*E*) density, defined as in Binney and Tremaine (1987) *Lower panel:* Same for the spatial density $\rho (r)$ , from the simulations of Preto and Amaro-Seoane (2010) using their FP code which is isotropic, orbit-averaged multi-mass in energy space. In different colours we have different moments in the evolution of the system in units of the relaxation time. Image adapted from Fig. 3 of Preto and Amaro-Seoane (2010)

Nevertheless, Preto and Amaro-Seoane (2010) and Amaro-Seoane and Preto (2011) explored the following situation: How long would cusp growth take if at some point a central core is carved in the stellar density in a galactic nucleus similar to the Milky Way? They choose a model with $\gamma =1/2$ as an initial condition, so that the isotropization time is $\ll t_\mathrm{rlx}(r_h)$. The results are shown in Fig. 49.

We can see in the figure that by $t \sim 0.25 \ t_\mathrm{rlx}(r_h)$, cusps with $\gamma _L\sim 1.5$ and $\gamma _H \sim 2$ ($p_L\sim 0.05$ and $p_H\sim 0.5$, where the subscript “L” refers to the light species and “H” to the heavy stars) are *fully developed* ($\sim 0.02$ pc if scaled to a Milky Way-like nucleus). For masses similar to Sgr A*, ${\mathscr {M}}_{\bullet } \lesssim 5 \times 10^6 M_\odot$, this is shorter than a Hubble time. Hence, if indeed a carving event depleted the inner agglomeration of stars around the MBH, as soon as only 6 Gyr later a very steep cusp of stellar-mass black holes would have had time to re-grow.

I must note that this result is different to what Merritt (2010) finds, but this is probably due to the fact that the author only takes into account the effect of dynamical friction from the light stars over the heavy stars, and he neglects the scattering of the heavy stars. In this respect, he is limited in his approach to the early evolution of the system, when the heavy stars only represent a minor perturbation on the light stars. As a matter of fact, very similar results to ours were derived later by Gualandris and Merritt (2012).

The impact on EMRI production is the following: If carved nuclei were common in the range of masses relevant to an observatory like LISA, then we would be cutting down production of old remnants significantly. However, even if our Milky Way *had* a hole in its stellar cusp, LISA EMRI rates peak around ${\mathscr {M}}_{\bullet } \sim 4 \times 10^5 - 10^6 M_\odot$ and re-growth times are $\lesssim 1$ Gyr for ${\mathscr {M}}_{\bullet } \lesssim 1.2 \times 10^6 M_\odot$, so that we still expect that a substantial fraction of EMRI events will originate from segregated stellar cusps

On the other hand, strong mass segregation not only “comes to the rescue” in the case of carved nuclei. It helps in the production of EMRIs. Amaro-Seoane and Preto (2011) estimate that thanks to strong mass segregation one might expect EMRI even rates to be $\sim 1-2$ orders of magnitude larger than one would expect from using the Bahcall and Wolf solution, as they show.

Their solution for the weak branch is physically unrealistic, since it predicts a too high event rate because it uses unreasonably high number fractions of stellar-mass black holes $f_\bullet$ ($\ge 0.05$). In a more realistic case, when $\varDelta \sim 0.03$, ($f_\bullet \sim 10^{-3}$) the Bahcall and Wolf solution would lead to a strong suppression of the EMRI rate to—at best—a few tens of events per Gyr.

The new solution of strong mass segregation implies a higher $\rho _{\bullet }$ well inside the influence radius of the MBH, so that we have a boost in the diffusion of stellar-mass black holes close to the MBH. When going from number fractions that are based on unrealistic IMF, such as in Bahcall and Wolf (1977) (say $\varDelta =3$) to realistic values ($\varDelta =0.03$), the event rate is suppressed by factors of $\sim 100$–150, if we ignore strong mass segregation. Thanks to this new solution, based on more realistic physics, even for low values of $\varDelta =0.03$, we boost the rates from few tens to a few hundred per Gyr, $\sim 250$/Gyr if we consider a mass ratio of 10 between the stellar-mass black holes and the MS stars and if we take a fractional number for stellar-mass black holes of $f_{\bullet }=0.001$.

### Tidal separation of binaries

Another process contributing to the creation of EMRIs has its origin in the work of Hills (1988), where he describes the possibility of finding escaping stars that originate by this process:

*A close but Newtonian encounter between a tightly bound binary and a million solar mass black hole causes one binary component to become bound to the black hole and the other to be ejected at up to 4000 km/s. The discovery of even one such hypervelocity star coming from the Galactic center would be nearly definitive evidence for a massive black hole. The new companion of the black hole has a high orbital velocity which increases further as its orbit shrinks by tidal dissipation. The gravitational energy released by the orbital shrinkage of such a tidal star can be comparable to its total nuclear energy release.*

His work, about the tidal separation of binaries by a MBH, did not have a big impact for some 15 years until the discovery of the so-called “hyper-velocity stars”, stars with a velocity of $> 10^3\,\mathrm{km\,s}^{-1}$, which he had predicted. Indeed, several such stars have been discovered in the last years. I refer the reader to Brown et al. (2009) for a discussion of the properties of these stars, as well as for references.

While one of the objects is ejected into the stellar system, the other binary member can remain bound to the MBH on a rather tight orbit. If this star happens to be a compact object, then we would have an EMRI which would be rather “immune” to the problems of EMRIs caused by two-body relaxation. Since the tidal separation happens very close to the MBH, the CO will have a shorter apoapsis (usually only tens of times the periapsis distance) and thus, potential tugs that lead it out of the capture orbit are reduced. This process was described by Miller et al. (2005). The properties of these EMRIs are very interesting and I describe the process in this section, both from an astrophysical point of view and the observational signature.

**Fig. 50:**
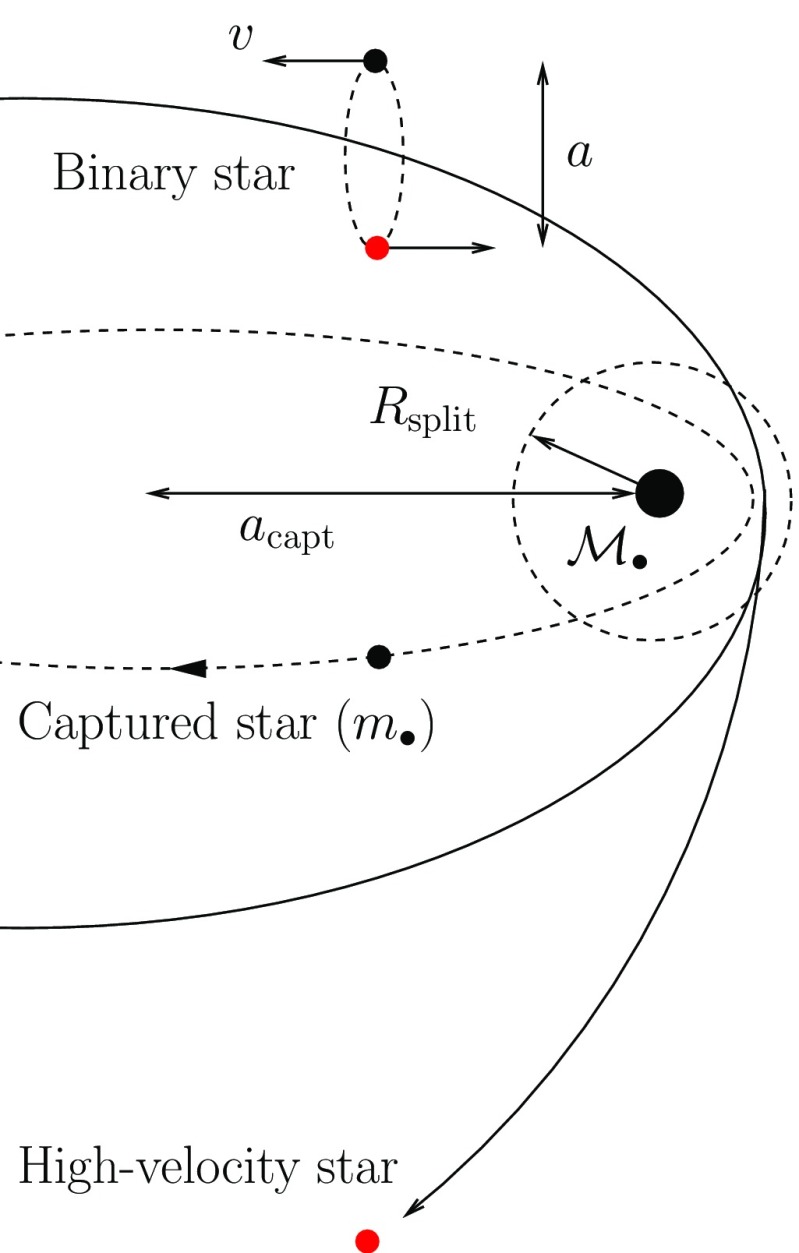
Schematic illustration of the process of the tidal separation of a binary by a MBH of mass ${\mathscr {M}}_{\bullet }$. $R_\mathrm{split}$ is defined as the radius within which the tidal forces of the MBH overcome the binding energy of the binary

In Fig. 50, we have a schematic view of the process. A binary which happens to fly by close enough to the central MBH will be tidally separated because the tides acting on the pair overcome the gravity in the binary. One of the stars is captured, meaning that it will be bound to the MBH, after losing a little energy compared to what it had before, and the other companion of the binary will obtain a bit more energy after the separation, so that it will be ejected with a high velocity, as in a slingshot. It is rather straightforward to make a toy model for the process and get the scalings, which sheds light on the process.

The size of the region where this process will occur, $R_\mathrm{split}$, is proportional to the size of the object, the separation of the binary (i.e. about the semi-major axis), the mass of the binary $m_\mathrm{bin}$ and the mass of the central MBH, ${\mathscr {M}}_{\bullet }$, as it was in the case of the tidal disruption of an extended star. Actually, we can follow the analogy very closely, except in that case we have to come closer to the MBH to have the tidal forces overcoming the binding energy of the binary, since $a > R_{\star }$, with $R_{\star }$ the radius of the star.

83 $$\begin{aligned} R_\mathrm{split} \sim a \left( \frac{{\mathscr {M}}_{\bullet }}{m_\mathrm{bin}}\right) ^{1/3}. \end{aligned}$$

The orbital velocity in the binary can be easily computed as follows,

84 $$\begin{aligned} v \sim \sqrt{\frac{G\,m_\mathrm{bin}}{a}}. \end{aligned}$$

I now normalise the last equation to nominal values assuming that it is a hard binary in a galactic nucleus,

85 $$\begin{aligned} v \sim 133\,\mathrm{km\,s}^{-1} \left( \frac{m_\mathrm{bin}}{2M_{\odot }} \right) ^{1/2} \left( \frac{a}{0.1\mathrm{AU}} \right) ^{-1/2}. \end{aligned}$$

**Fig. 51:**
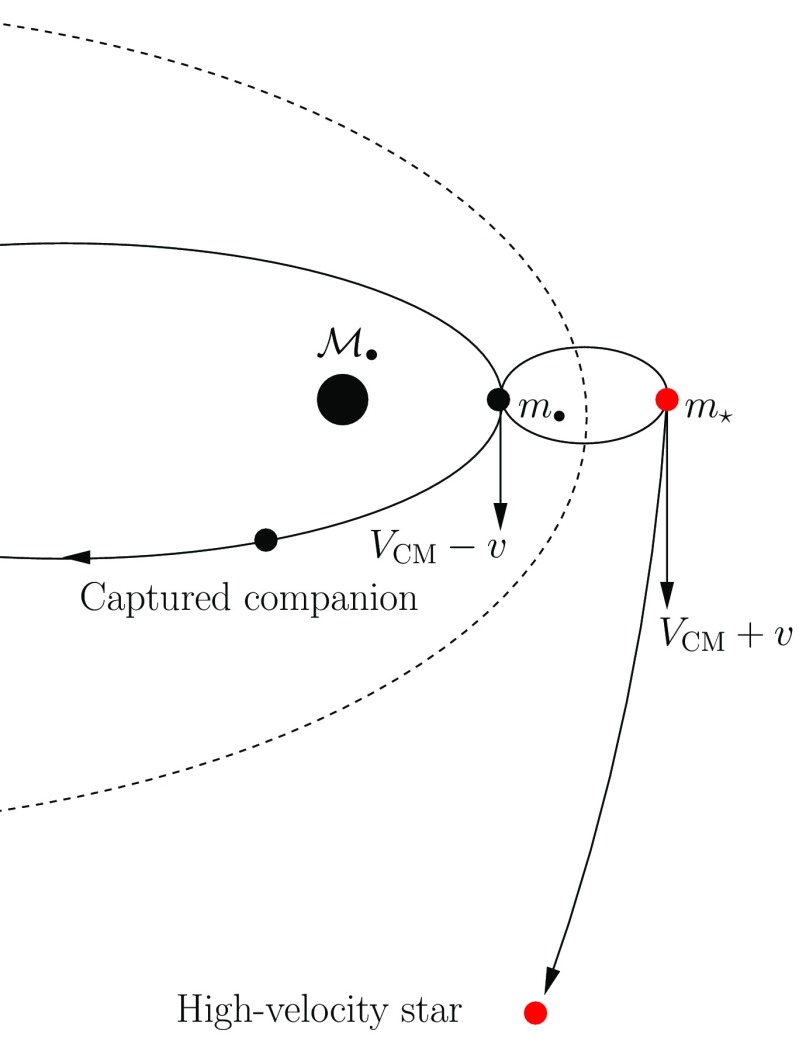
Zoom of Fig. 50 for the splitting of the pair. I assume a parabolic encounter (dashed curve) for the COM of the binary and a mass $m_{\bullet }$ and $m_{\star }$ for the stars

Figure 51 shows a zoom-in of Fig. 50 at the moment in which the binary is at the periapsis of the MBH. We now estimate the ejection velocity. The centre-of-mass (CM) of the binary has a velocity $V_\mathrm{CM}$ which we can easily calculate by assuming that the encounter is parabolic

86 $$\begin{aligned} V_\mathrm{CM} \gtrsim \sqrt{\frac{G\,{\mathscr {M}}_{\bullet }}{R_\mathrm{split}}}\sim \,v \left( \frac{{\mathscr {M}}_{\bullet }}{m_\mathrm{bin}}\right) ^{1/3} \gg v. \end{aligned}$$

This allows us to estimate the ejection velocity of the slingshot star, because the difference of energy will be

87 $$\begin{aligned} \pm \delta E \simeq V_\mathrm{CM}\cdot v \ge v^2 \left( \frac{{\mathscr {M}}_{\bullet }}{m_\mathrm{bin}} \right) ^{1/3} \simeq \frac{v_\mathrm{eject}^2}{2}. \end{aligned}$$

Then, we have that

88 $$\begin{aligned} v_\mathrm{eject} \gtrsim \left( \frac{{\mathscr {M}}_{\bullet }}{m_\mathrm{bin}}\right) ^{1/6}. \end{aligned}$$

Since we are dealing with a binary, the star of mass $m_{\bullet }$, which we assume to be a stellar-mass black hole, will be slowed down by *v*, as in Eq. (84) and the star of mass $m_{\star }$, which can be an extended star or a compact object, will be accelerated by the same amount. I now assume that in that moment the stars do not interact gravitationally with each other and they only “see” the potential created by the MBH. Therefore, we have a simple situation with a simplified geometry that allows us to compute the initial orbits of the two companions in the pair at the moment of splitting.

Hence, the stellar-mass BH is bound to the MBH and the escaping star leaves the system with a high velocity, which is of the order of the velocity in the binary, typically of about $\sim 10\,\hbox {km}\,s$, multiplied by the same mass ratio as in Eq. (83) but to a different power, as we can see in Eq. (88).

One very interesting aspect of this particular process to produce the capture of compact objects by MBHs is the eccentricity that the orbit has. We can estimate it roughly by computing the semi-major axis of the bound pair after the separation of the initial binary, $a_\mathrm{capt}$,

89 $$\begin{aligned} a_\mathrm{capt} \approx a \left( \frac{{\mathscr {M}}_{\bullet }}{m_\mathrm{bin}} \right) ^{2/3}. \approx 10^4a \end{aligned}$$

As we can see in Fig. 50, we can approximate the separation radius $R_\mathrm{split}$ to the periapsis distance,

90 $$\begin{aligned} R_\mathrm{peri} = (1-e_\mathrm{capt})\,a_\mathrm{capt}\approx R_\mathrm{split}. \end{aligned}$$

Hence, this kind of sources will typically have a capture eccentricity of

91 $$\begin{aligned} e_\mathrm{capt} = 1-\left( \frac{{\mathscr {M}}_{\bullet }}{m_\mathrm{bin}} \right) ^{-1/3} \sim 0.98. \end{aligned}$$

Contrary to “usual” EMRIs, tidally-split MS stars have a low eccentricity when they form, and possibly when they reach the bandwidth of the detector (for convenience, we will call these tidally-split EMRIs “TSEMRI”). This is because no energy needs to be dissipated in order to have a capture. As a result, capture can occur at much larger radii than is possible in the two-body case, as described in Miller et al. (2005). For a $10\,M_{\odot }$ object this should be of the order $1-e_\mathrm{TSEMRI}\approx 0.99$. On the other hand, we have seen that typical EMRI eccentricities when reaching the LISA bandwidth are $1-e\approx [10^{-3},\,10^{-7}]$

In order to understand the difference in terms of detectability, we need to introduce some definitions of the geometric model of signal analysis. We treat the waveforms as vectors in a Hilbert space (Helstrom 1968), which allows us to define the natural scalar product

92 $$\begin{aligned} \left<h\left| s\right. \right> := 2\int _{0}^{\infty }{df}\,\frac{ \tilde{h}(f)\tilde{s}^{*}(f) + \tilde{h}^{*}(f)\tilde{s}(f)}{S_{n}(f)}, \end{aligned}$$

where

93 $$\begin{aligned} \tilde{h}(f) = \int _{-\infty }^{\infty }\, dt\, h(t)e^{2\pi \imath ft} \end{aligned}$$

is the Fourier transform of the time domain waveform *h*(*t*). I have introduced the $S_{n}(f)$, which is the one-sided noise spectral density of LISA, see e.g., Thorne (1987), Finn (1992). One can think of LISA as two detectors, so that the signal in each of them is given by $s_{i}(t) = h_{i}(t)+n_{i}(t)$, with $i=1,\,2$ label each detector. I adopt the assumption that the noise $n_{i}(t)$ is stationary, Gaussian, uncorrelated in each detector and characterised by the noise spectral density $S_{n}(f)$. Hence, we can define the signal-to-noise ratio in each detector as

94 $$\begin{aligned} \rho _{i} = \frac{\left<h\left| s_{i}\right. \right>}{\sqrt{\left<h\left| h\right. \right>}}. \end{aligned}$$

Therefore, if we consider the waveform of a TSEMRI and compare it with a normal EMRI, we can calculate the mismatch of their expected signal-to-noise ratio for LISA as

95 $$\begin{aligned} {\mathscr {M}} := 1 - \frac{\left<h_\mathrm{TSEMRI}\left| h_\mathrm{EMRI}\right. \right>}{\sqrt{\left<h_\mathrm{TSEMRI}\left| h_\mathrm{TSEMRI}\right. \right> \left<h_\mathrm{EMRI}\left| h_\mathrm{EMRI}\right. \right>}}. \end{aligned}$$

For a TSEMRI and a normal EMRI starting with exactly the same orbital parameters at the GC and coloured for LISA, I have calculated with the LISACode^15^ (Petiteau et al. 2008) that there is a mismatch of 99.9971%. Using the standard definition of signal-to-noise ratio $\rho _i=\left<h|s_i\right>/\sqrt{\left<h|h\right>}$, we have that the TSEMRI is calculated to have an average signal-to-noise ratio of $\rho _\mathrm{TSMI} \sim 27637$ and the normal EMRI of $\rho _\mathrm{EMRI} \sim 18848$, both set to be at a distance of 8.3 kpc.

**Fig. 52:**
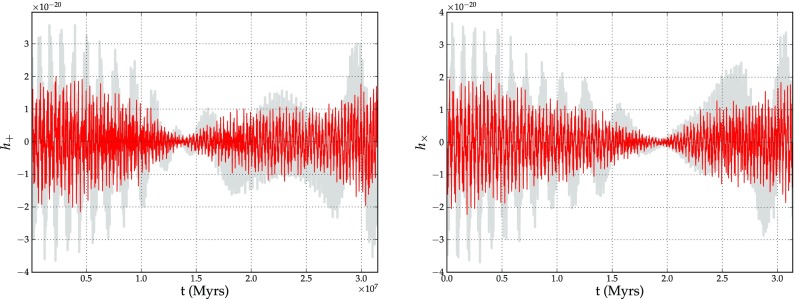
Waveforms of a normal EMRI and a TSEMRI (see text) superimposed for a full year of data before the final plunge, which has been defined to happen at a periapsis of $r_\mathrm{plunge} \equiv 2\times r_\mathrm{ISCO}$, with $r_\mathrm{ISCO}$ the radius of the innermost stable circular orbit, which is $\sim 8\,M$ in this case. The mass of the central MBH is $3\times 10^6\,M_{\odot }$, the mass of the star $0.53\,M_{\odot }$. The spin of the MBH is set to $a = 0.5\,M$ and we neglect the spin of the star

**Fig. 53:**
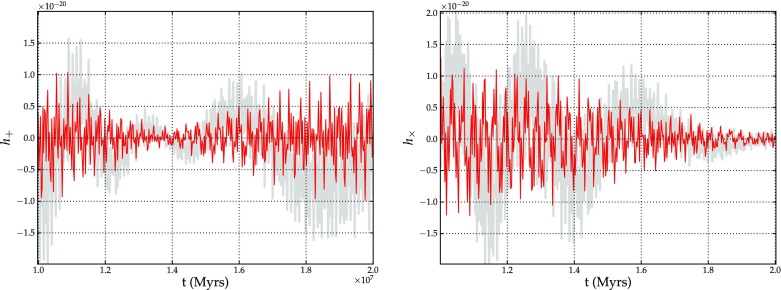
Zoom of Fig. 52. For the two figures only one point out of 1600 is used to make the plot lighter. Still, we can see that even in the region of maximum overlap there is a significant difference. In the computation of the mismatch function of Eq. (95), however, the full waveform has been used

Figure 52 shows the waveforms for an observer at $\theta = 55$ degrees, with origin at the MBH, with a mass of $3\cdot 10^6\,M_{\odot }$ and z-axis along direction of big black hole spin. The waveforms are from Steve Drasco and have been calculated using the kludge approximation of Gair and Glampedakis (2006) (Fig. 53).

### A barrier for captures ignored by rotating MBHs

A number of authors have addressed the question of EMRI event rates in a Milky Way-like galaxy. The numbers differ but a common denominator to all estimates is that the number of “direct plunges” is much larger than slowly decaying, “adiabatic” EMRIs. This is so simply because the region of the galaxy from which potential plunges originate contains many more stars than the volume within which we expect EMRIs, as we have seen in Sect. 6.

For a long time “plunges” have been considered to be irrelevant for the purposes for which EMRIs are best. After one intense burst of radiation, the source would be lost along with, obviously, the SBH. Some studies have looked into that, such as Hopman et al. (2007), which is probably one of the most meticulous one since it incorporates a high realism of the physics in that regime. However, the conclusions of the authors are that these sources are not interesting because they could only be detected if they originated in our own Galactic Centre. Later, Berry and Gair (2013) addressed the possible constraints on parameters of our Milky Way’s MBH if one of this bursting sources was to be observed with LISA.

In contrast, a few years later, Amaro-Seoane et al. (2013b) showed that since MBH are likely to be spinning, it is actually very hard for a SBH on a plunge orbit to “hit” the MBH. They show that the majority of plunging orbits for spinning MBHs are actually not plunging but EMRI orbits. They prove that since spin allows for stable orbits very near the LSO in the case in which the EMRI is prograde, the contribution of each cycle to the SNR is much bigger than each cycle of an EMRI around a non-spinning MBH. On the other hand, retrograde orbits “push the LSO outwards” and hence, it is easier for a SBH to plunge, and the EMRI is lost. However, this situation is not symmetric, resulting in an effective enhancement of the rates. These results have been also confirmed by Will and Maitra (2017) using a different method based in a post-Newtonian algorithm. In this approach these EMRI spend a lower number of cycles in the band of the detector. However, as Will and Maitra (2017) state, “(...) the PN approximation is being pushed up to or beyond its limit of validity, so we do not wish to claim too much accuracy for our values of $T_\mathrm{{plunge}}$ in Table III.”

Amaro-Seoane et al. (2013b) also show that vectorial coherent relaxation is not efficient enough to turn a prograde orbit into a retrograde one, which would be fatal for this scenario, once the evolution is dominated by GW emission. This result is crucial in the formation of EMRI sources. To understand why, first we need to introduce the problem of the so-called “Schwarzschild barrier”.

**Fig. 54:**
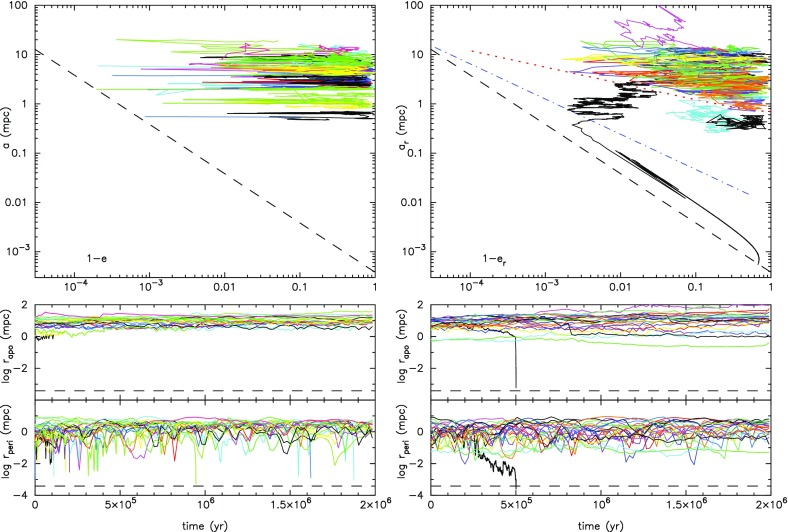
The Schwarzschild barrier. In the left panel, we have the Newtonian case and in the right one the simulations with relativistic corrections. We clearly see the barrier and also a particle crossing it. Images reproduced with permission from arXiv e-print of Merritt et al. (2011)

Merritt et al. (2011) performed direct-summation *N*-body simulations and found that EMRI event rates are severely suppressed when introducing relativistic precession in the integrations. The precession limits the action of torques from the stellar potential in the orbital angular momenta. Nevertheless, they do find some particles that do cross this barrier (the Schwarzschild barrier, to use their nomenclature). In Fig. 54 we see this scenario. This is from Merritt et al. (2011) and shows a Newtonian simulation in the left panel. The authors display the semi-major axis and eccentricity of the two-body system consisting of one star and the MBH. In the right panel, they depict the situation with all the relativistic correction terms “switched on”. $a_r$ and $e_r$ are the 1PN generalisations of the semi-major axis and eccentricity. In the upper panel, the red dotted line corresponds to the barrier, given by their expression:

96 $$\begin{aligned} {\tilde{a}} = C_\mathrm {SB} \left( 1-e^2\right) ^{-1/3}, \end{aligned}$$

where $C_\mathrm {SB}$ is a constant of order unity. The blue, dash-dotted line corresponds to the GW capture.

**Fig. 55:**
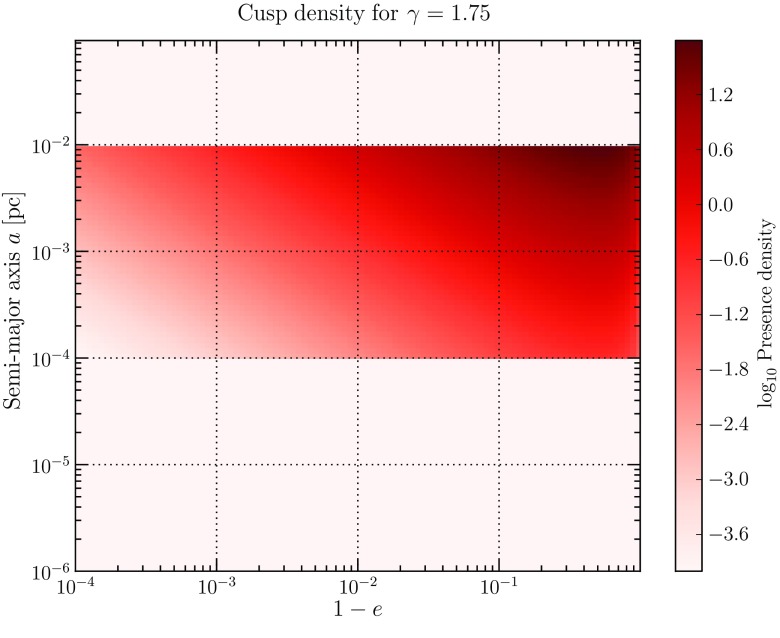
Theoretical distribution for the density of presence of a cusp of power-law 1.75 around a MBH, using a truncated distribution

This finding has been confirmed and quantified by Brem et al. (2014) using a statistical sample of 2500 direct-summation *N*-body simulations using both a post-Newtonian but also, and for the first time, a geodesic approximation for the relativistic orbits. However, in their work, the authors do not find a sharp transition “barrier”, but an area in phase space within which particles (stars) spend more time than outside of it.

**Fig. 56:**
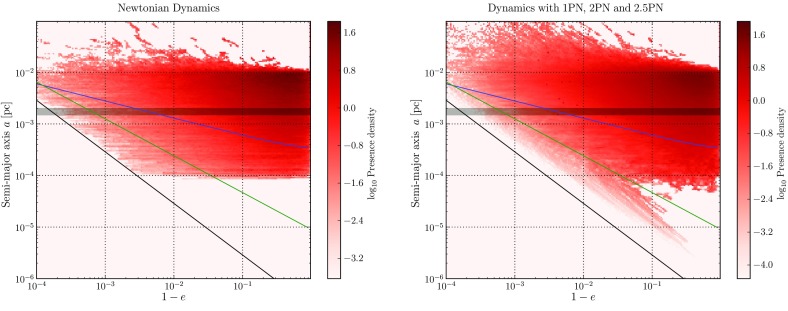
Integrated presence density for the Newtonian (left panel) and the relativistic case (right panel). The lines indicate the position of the Schwarzschild barrier with $C_\mathrm{SB} = 0.35$ (*blue*) and the limit for capture onto inspiral orbits for non-resonant relaxation (*green*)

A better way of displaying this barrier is not by following a few individual orbits, which are not representative of the phenomenon, but to depict a full presence density map. Indeed, in Figs. 55 and 56, we have the normalised presence density as a histogram in the $(a, 1-e)$ plane for the Newtonian case, Fig. 56 (left panel) and the relativistic case (right panel), and I give the theoretical distribution in Fig. 55. In these figures, we see that on the right of the blue line there is a region within which stars significantly spend more time than in other areas. If we consider our specific setup, there are 3 different regions in the $(a,1-e)$ plane where different mechanisms are efficient. In the right region, where pericenters are large, coherent relaxation plays the dominant role. The left border of this region is roughly given by the appearance of the Schwarzschild precession which inhibits stellar-mass black holes from experiencing coherent torques (Brem et al. 2014).

Bar-Or and Alexander (2014) addressed this problem in terms of the adiabatic invariance of the precession against the slowly varying random background torques and find that this precession-induced barrier in angular momentum is maximal for smooth noise. The barrier is not such, nor a reflecting one. It is an effective division of phase-space where resonant relaxation is effective, and where it is not.

This interesting and pioneering scenario would obviously imply a priori a severe suppression of EMRI event rates, *if we relied on resonant relaxation*. While this is true for EMRIs originating at these distances, the whole picture looks much more different at larger semi-major axis and eccentricities.

We have seen in Sect. 6.2 that the small compact object will be on a so-called “plunging orbit” if $e\ge e_\mathrm{plunge} \equiv 1-4\,R^{}_\mathrm{Schw}/a$. It has been claimed a number of times by different authors that this would result in a too short burst of gravitational radiation which could only be detected if it was originated in our own Galactic Centre (Rubbo et al. 2006; Hopman et al. 2007; Yunes et al. 2008; Berry and Gair 2013) because one needs a coherent integration of some few thousand repeated passages through the periapsis in the LISA bandwidth.

Therefore, such “plunging” objects would then be lost for the GW signal, since they would be plunging “directly” through the horizon of the MBH and only a final burst of GWs would be emitted, and such burst would be very difficult to recover, since the very short signal would be buried in a sea of instrumental and confusion noise and the information contained in the signal would be practically nil.

**Fig. 57:**
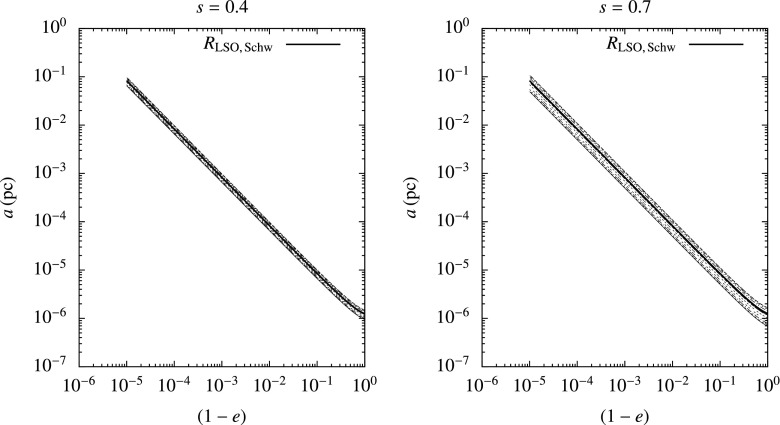
LSO for a MBH of mass $4\times 10^4\,M^{}_{\odot }$ and a SBH of mass $m_{\bullet }=10\,M^{}_{\odot }$ for a Kerr MBH of spin $s=0.4$ (left) and $s=0.7$ (right). The Schwarzschild separatrix is given as a solid black line. Curves above it correspond to retrograde orbits and inclinations of $\iota =5.72,\,22.91,\,40.10,\,57.29,\,74.48$ and $89.95^{\circ }$ starting from the last value ($89.95^{\circ }$). In the left panel we can barely see any difference from the different inclinations due to the low value of the spin

**Fig. 58:**
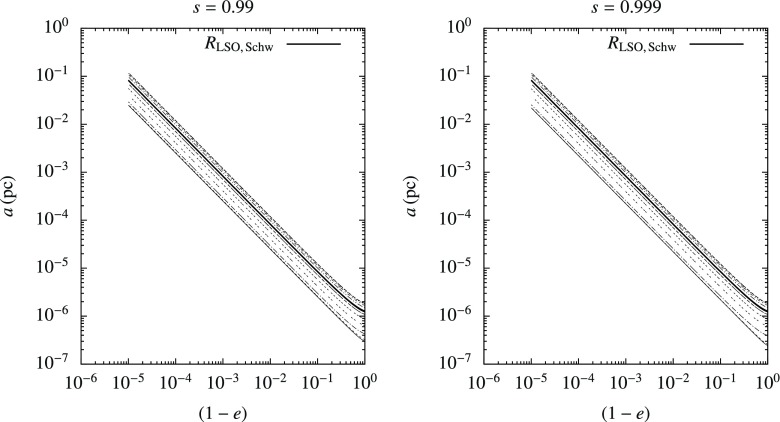
The same as in Fig. 57 but for a spin of $s=0.99$ (left) and $s=0.999$ (right panel). The larger the spin, the “further away” the Kerr LSO gets from the Schwarzschild LSO

However, Amaro-Seoane et al. (2013b) showed that this is not true. Figures 57 and 58 show plots of the location of the LSO in the plane *a* (pc)–$(1-e)$, including the Schwarzschild separatrix between stable and unstable orbits, $p -6 - 2e = 0$, for both prograde and retrograde orbits and for different values of the inclination $\iota$. Each plot corresponds to a different value of the spin, showing how increasing the spin makes a difference in shifting the location of the separatrix between stable and unstable orbits, pushing prograde orbits near $GM^{}_{\bullet }/c^{2}$ while retrograde orbits are pushed out towards $9GM^{}_{\bullet }/c^{2}$. The procedure used to build these plots is a standard one. Briefly, given a value of the dimensionless spin parameter $s\equiv a^{}_{\bullet }c^{2}/(GM^{}_{\bullet })$ and a value of the eccentricity and inclination angle $\iota$.

Amaro-Seoane et al. (2013b) estimated that the number of cycles that certain EMRI orbital configurations, which were thought to be plunging orbits (or orbits with no sufficient cycles), in the case of non-spinning MBHs, can spend in a frequency regime of $f\in [10^{-4},1]$ Hz during their last year(s) of inspiral before plunging into the MBH. This is important to assess how many of these EMRIs will have sufficient Signal-to-Noise Ratio (SNR) to be detectable. It was found that (prograde) EMRIs that are in a “plunge” orbit actually spend a significant number of cycles, more than sufficient to be detectable with good SNR. The number of cycles has been associated with $N^{}_{\varphi }$ (the number of times that the azimuthal angle $\varphi$ advances $2\pi$) which is usual for binary systems. However, as I have discussed above, the structure of the waveforms from EMRIs is quite rich since they contain harmonics of three different frequencies. Therefore, the waveforms have cycles associated with the three frequencies $(f^{}_{r},f^{}_{\theta },f^{}_{\varphi })$ which makes them quite complex and in principle this is good for detectability (assuming we have the correct waveform templates). Moreover, these cycles happen just before plunge and take place in the strong field region very near the MBH horizon. Then, these cycles should contribute more to the SNR than cycles taking place farther away from the MBH horizon.

The authors also estimate the impact on the event rates. Since “direct plunges” are actually disguised EMRIs, although with a higher eccentricity. They prove that

97 $$\begin{aligned} \frac{{{a}_\mathrm{EMRI}^\mathrm{Kerr}}}{{{a}_\mathrm{EMRI}^\mathrm{Schw}}}= & {} {\mathscr {W}}^{\frac{-5}{6-2\gamma }}(\iota ,\,s) \end{aligned}$$

98 $$\begin{aligned} \frac{{\dot{N}_\mathrm{EMRI}^\mathrm{Kerr}}}{{\dot{N}_\mathrm{EMRI}^\mathrm{Schw}}}= & {} {\mathscr {W}}^{\frac{20\gamma -45}{12-4\gamma }} (\iota ,\,s) . \end{aligned}$$

Here, ${\mathscr {W}}$ is a function that depends on $\iota$, the inclination of the EMRI and *s*, its spin.^16^ I also have assumed that the stellar-mass black holes distribute around the central MBH following a power-law cusp of exponent $\gamma$, i.e., that the density profile follows $\rho \propto r^{-\gamma }$ within the region where the gravity of the MBH dominates the gravity of the stars, with $\gamma$ ranging between 1.75 and 2 for the heavy stellar components (Peebles 1972; Bahcall and Wolf 1976, 1977; Amaro-Seoane et al. 2004; Preto et al. 2004; Alexander and Hopman 2009; Preto and Amaro-Seoane 2010; Amaro-Seoane and Preto 2011) (see Gurevich 1964 for an interesting first idea of this concept).^17^ For instance, for a spin of $s=0.999$ and an inclination of $\iota = 0.4\,$rad, they estimate that ${\mathscr {W}}\sim 0.26$ and, thus, $\dot{N}_\mathrm{EMRI}^\mathrm{Kerr} \sim 30$.

To sum up, the existence of the barrier prevents “traditional EMRIs” from approaching the central MBH, but if the central MBH is spinning the rate will be dominated by highly-eccentric extreme mass ratio inspirals anyway, which insolently ignore the presence of the barrier, because they are driven by chaotic two-body relaxation.

### Extended stars EMRIs

In this section, I review the idea described in Freitag (2003b) that MS stars can be potential sources of GWs in our Galactic Centre. I include this in this section because in the whole review our standard CO is considered to be a SBH and so, it falls into the category of “not in the standard model”.

Indeed, a MS star can reach close enough distances to the central MBH depending on its average density and stellar structure. For a mass of around $0.07\,M_{\odot }$, the density of the MS star is maximum and corresponds to the transition to a sub-stellar object (Chabrier and Baraffe 2000). For masses smaller than $0.3 - 0.4\,M_{\odot }$, the core is totally convective and can be described with a polytrope of index $n = 3/2$.

**Fig. 59:**
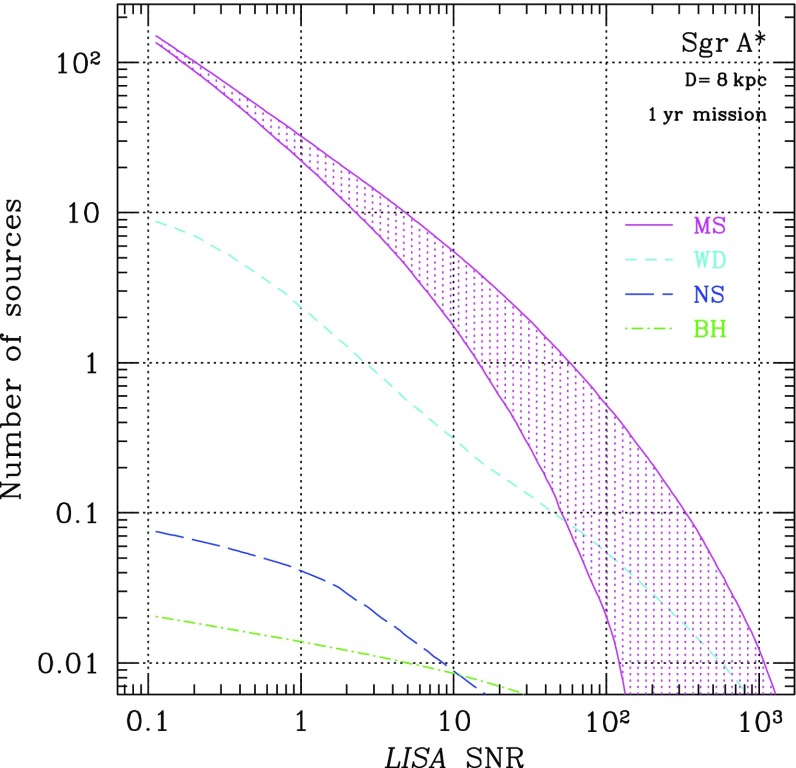
Number of sources at the GC depending on their compactness. This figure displays the number of MSSs, WDs, NSs and stellar-mass black holes as a function of the SNR in LISA for a one year mission and assuming a distance of 8 kpc. Image reproduced with permission from Freitag (2003b), copyright by AAS

Freitag (2001) and Freitag (2003b) estimated the number of single MS stars which can become an abundant source of GWs in our GC by inspiraling into the central MBH. In his work, the author calculated with Monte Carlo simulations that there must be one to a few low-mass MS stars sufficiently bound to the GC to be discernible by LISA. In Fig. 59, we show some of the most relevant results of the investigation. Nevertheless, we note that the assumptions made by the numerical tool are probably biasing the results to an overestimation. These assumptions rely in the nature of the Monte Carlo code.

### The butterfly effect

An interesting effect, described in Amaro-Seoane et al. (2012b), is the lack of determinism in an EMRI system if a perturbing star is close enough to the binary formed by the MBH and the SBH. One immediate question that arises is how realistic it is to assume that we can have a second star so close to the EMRI so as to perturb it.

I estimate how likely is to have a star close enough to perturb the EMRI in a measurable way. For this, I take our Galactic Centre as a representative system of the scenario that we want to analyse. If we admit that for Sgr A* half of the mass within the orbit of S-2, which has a periapsis of $6\times 10^{-4}$pc (Ghez et al. 2008; Genzel et al. 2010), is $M_\mathrm{encl}/2 = \eta \times M_{\bullet }$, with $\eta \le 0.040$ and $M_{\bullet }$ the mass of the MBH (Gillessen et al. 2009), i.e., $M_\mathrm{encl} = 172,000\,M_{\odot }$, and we assume that the stars build a cusp following a power-law of the type $R^{-\gamma }$, then we can estimate that the mass at radius *R* is

99 $$\begin{aligned} M(R) = \int _{0}^{R} 4 \pi r^2 \rho (r) \, dr \propto \int _0^R r^{-\gamma +2} dr \propto R^{3-\gamma }, \end{aligned}$$

for $\gamma <3$. Hence, the number of stars within a sphere of radius *R* is given by

100 $$\begin{aligned} N(R) \simeq 8.6 \times 10^4 \left( \frac{R}{6\times 10^{-4} \mathrm{~pc}}\right) ^{3-\gamma }. \end{aligned}$$

And so, the radius within we can expect to find in average a star is

101 $$\begin{aligned} R_1 \simeq 6\times 10^{-4} \mathrm{~pc} \times \left( \frac{1}{8.6 \times 10^4}\right) ^{\frac{1}{3-\gamma }}. \end{aligned}$$

**Fig. 60:**
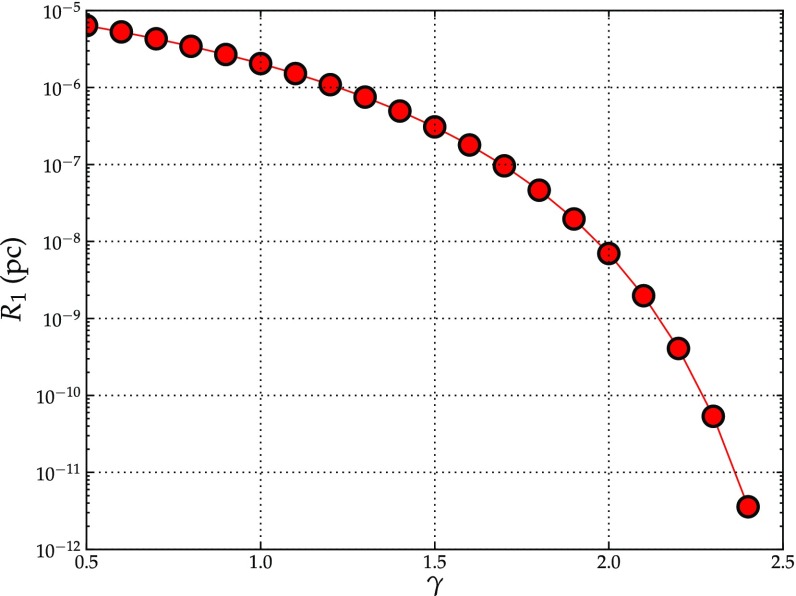
Evolution of the one-star radius as a function of the slope as in Eq. (101). We can see that for very mild slopes and even a core the distances are within a millihertz gravitational-wave detector similar to LISA; i.e., of orbital periods of $10^5$ s. In this regime we expect sources of GWs. For instance, an EMRI of $10\,M_{\odot }$ with a MBH of $4\times 10^{6}M_{\odot }$ has a semi-major axis of about $a_{\bullet } \approx 8\times 10^{-4}$ pc and is well within the bandwidth

We note, however, that the value derived for $\eta$ is not observational. As a matter of fact, with current limitations in the observations, it is impossible to know whether all mass enclosed by the orbit of S-2 corresponds to the MBH or it contains also an “extended” component. Hence, in order to obtain $\eta$, one has to model the system by admitting that it consists of a punctual source (the MBH) along with a stellar component whose properties are parametrised by following a model, not an observation. Figure 60 shows the dependence on $\gamma$ of $R_1$. We can see that $R_1 \simeq 3 \times 10^{-7}$ pc for $\gamma = 1.5$ or $7\times 10^{-8}$ pc for $\gamma = 1.75$, see Amaro-Seoane et al. (2004), Freitag et al. (2006b), Preto et al. (2004). These distances are of the same order of magnitude than an EMRI, which is within the bandwidth of a LISA-like observatory. Even if this argument is based on the concept of a cusp and, hence, it is difficult to define at such short radii, in my work with Marc Freitag and Vassiliki Kalogera (Freitag et al. 2006b) we derive in our Milky Way-like G25 model some $15\,M_{\odot }$ within $3\times 10^{-4}$ pc. It is possible that at such distances the mass density is totally dominated by stellar-mass black holes, but the work of Freitag et al. (2006b) does not allow one to resolve them for distances shorter than 0.01 pc. In this case, strong mass segregation would play a crucial role (Alexander and Hopman 2009; Preto and Amaro-Seoane 2010; Amaro-Seoane and Preto 2011), since for the kind of slopes that one can expect in the case the density is dominated by stellar-mass black holes, the “one-star” radius is much shorter.

**Fig. 61:**
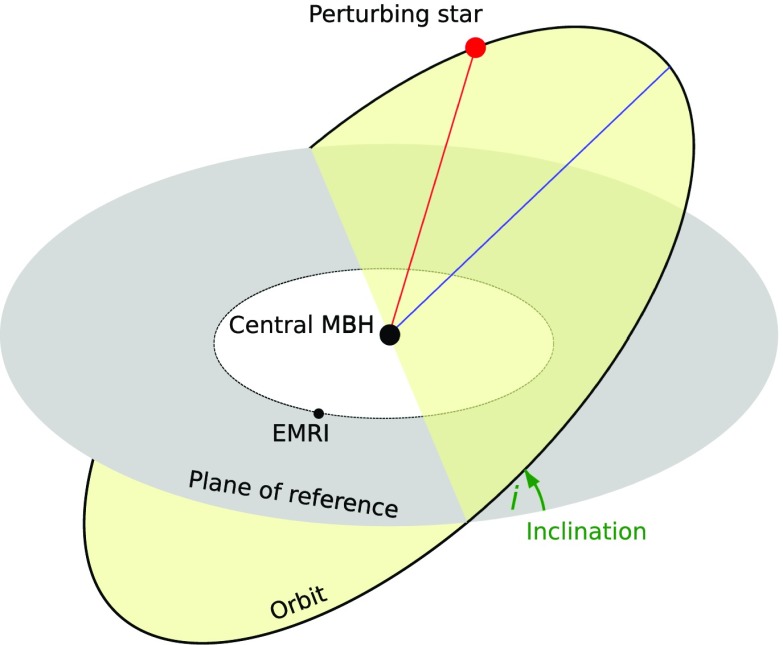
Description of the scenario for the butterfly effect by Amaro-Seoane et al. (2012b) (adapted from a figure by Lucas Snyder)

In Fig. 61, we have the initial setup for the fiducial case by Amaro-Seoane et al. (2012b). The mass of the MBH is assumed to be ${M}_{\bullet } = 10^{6}\,M_{\odot }$, the initial semi-major axis of the EMRI of $a_{\bullet ,\,\mathrm i} \simeq 1.45\times 10^{-6}$ pc (i.e., it is well within the band of LISA) , the mass of the EMRI is $m_{\bullet } = 10\,M_{\odot }$ (but they also successfully tested 5 and $1.44\,M_{\odot }$), the mass of the perturbing star is of $m_{\star } = 10\,M_{\odot }$, the initial semi-major axis of the star $a_{\star ,\,\mathrm i} \simeq 4.1 \times 10^{-6}$ pc, and the initial eccentricity $e_{\star ,\,\mathrm i} = 0.5$ and the inclination is $i_{\bullet ,\,\star } = 30^{\circ }$ at $T=0$.

We find that the interloper introduces an observable modification in the orbit of the EMRI when using a code that uses loss of energy via gravitational radiation at periapsis. The interesting result, though, is that when taking into account also the two first-order non-dissipative post-Newtonian contributions, the orbital evolution is not deterministic. We do not know what the stellar distribution around a MBH is at such short radii, but if this scenario was possible, then the detection of EMRIs would be much more challenging than it was thought, because the waveforms developed for detection would be of little use. There has also been work about the role of a massive perturber on an EMRI. I refer the reader to Chen et al. (2011), Yunes et al. (2011), Seto (2012).

### Role of the gas

Another proposal is related to the presence of massive accretion discs around MBHs. At distances of $\sim 0.1{-}1$ pc from the MBH and with typical accretion rates, these discs can be unstable to star formation (Collin and Zahn 1999; Levin and Beloborodov 2003; Goodman 2003; Goodman and Tan 2004; Milosavljević and Loeb 2004; Levin 2003, 2006; Nayakshin 2006). If, as in some calculations, there is a bias towards the production of massive stars in the disc, they could evolve to become black holes, which are then dragged in along with the disc matter. Alternately, massive stars on orbits that cross the disc could be captured and then evolve into black holes (Syer et al. 1991; Rauch 1995; Šubr and Karas 1999; Karas and Šubr 2001). Rates are highly uncertain as well as the mass of the stellar remnants formed (which could even be IMBHs). However these events would likely have a different signature waveform than those of the other two classes because they should occur on co-rotating, circular orbits lying in the equatorial plane of the spinning MBH if it has gained a significant fraction of its mass by accreting from the disc (Bardeen 1970; King et al. 2005; Volonteri et al. 2005). Moreover, there is the exciting possibility that in such a scenario the compact object would open a gap in the disc, which could lead to an optical counterpart to the EMRI event (Levin 2006).

Barausse et al. (2007) address the imprint on the waveform of compact, massive tori close to the central MBH. The kludge waveforms generated in their study were indistinguishable from pure Kerr waveforms in the regime on which they focused. Barausse and his collaborators later extended the study to a non self-gravitating torus with constant specific angular momentum and found that typically one should not expect big differences, although for a certain region of the parameter space the hydrodynamic drag acting on the EMRI does have an impact comparable to the radiation-reaction, so that it could, in principle, be measurable (Barausse and Rezzolla 2008). Later, this work was expanded in Barausse et al. (2014). Nevertheless, it is not clear what the appropriate gas distribution around the MBH is in the regime of their study. Perturbations to the SBH are likely to be negligible if accretion onto the hole happens in a low density, radiatively inefficient flow. Such flows are much more common than dense accretion discs, which in principle could yield observable phase shifts during the inspiral (Kocsis et al. 2011), at least within the redshift range in which we expect to observe EMRIs.

## Integration of dense stellar systems and EMRIs

### Introduction

In this section, we give a summary of the current numerical approaches available for studying stellar dynamics in systems for which relaxation is an important factor.^18^

As of writing this article, only approximate methods using a number of simplifying assumptions have been used to estimate the rates and characteristics of EMRIs. I review these approaches, their accomplishments and limitations. Thanks to the rapid computational power increase and the development of new algorithms, it is most likely that direct *N*-body techniques will soon be able to robustly confirm or disprove these approximate results and extend them. One of the main issues is that exceptionally long and accurate integrations are required to account correctly for secular effects such as coherent relaxation or Kozai oscillations. These requirements, and the extreme mass ratio pose new challenges to developers of *N*-body codes.

**Fig. 62:**
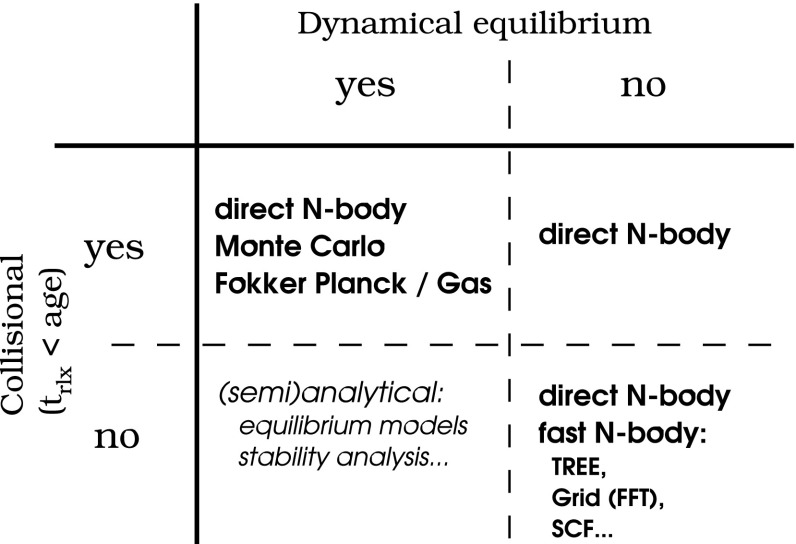
Possible methods to study the various realms of stellar dynamics

We can approximately classify the different kinds of techniques employed for studying stellar dynamics according to the dynamical regime(s) they can cope with. In Fig. 62 we have a classification of these techniques. (Semi-)analytical methods are generally sufficient only to study systems which are in dynamical equilibrium and which are not affected by collisional (relaxational) processes. In all other cases, including those of importance for EMRI studies, the complications that arise if we want to extend the analysis to more complex (realistic) situations, force us to resort to numerical techniques.

The *N*-body codes are the most straightforward approach from a conceptual point of view. In those, one seeks to integrate the orbital motion of *N* particles interacting gravitationally. It is necessary to distinguish between the *direct* *N*-body approaches which are extremely accurate but slow and the fast *N*-body approaches, which less accurate and therefore generally deemed inadequate for studying relaxing systems because relaxation is the cumulative effect of *small* perturbations of the overall, smooth, gravitational potential. Fast *N*-body codes are usually based on either TREE algorithms (Barnes and Hut 1986) or on an FFT (Fast Fourier Transform) convolution to calculate the gravitational potential and force for each particle (Fellhauer et al. 2000) or on an SCF (self-consistent-field) (Clutton-Brock 1973; Hernquist and Ostriker 1992) approach. I will not describe these numerical techniques in this section because they have never been used to study E/IMRIs and the approximations on which they are based make them unsuitable for an accurate study of such systems, since relaxation plays a role of paramount importance. Fast *N*-body algorithms can only be employed in situations in which relaxation is not relevant or over relatively short dynamical times, such as in studying bulk dynamics of whole galaxies.

On the other hand, if we want to study a system including both collisional effects and dynamical equilibrium, we can employ direct *N*-body codes or use faster approaches, like the Monte Carlo, Fokker Planck and Gas methods, which we will describe below. The only technique that can cope with all physical inputs is the direct *N*-body approach, in which we make no strong assumptions other than that gravity is Newtonian gravity (although nowadays post-Newtonian corrections have also been incorporated, see Sect. 8.8).

If we neglect capture processes driven by tidal effects, the region from which we expect most EMRIs to come is limited to $\sim$ 1–0.1 pc around the central MBH (see Sects. 7.6). In that zone the potential is totally spherical. Non-spherical structures such as triaxial bulges or stellar discs are common on scales of 100–1000 pc, and the nucleus itself may be non-spherical. For example, it could be rotating, as a result of a merger with another nucleus (Milosavljević and Merritt 2001) or due to dissipative interactions between the stars and a dense accretion disc (Rauch 1995).

It is unclear whether this effect could enhance the replenishment of the loss cone, see Murphy et al. (1991), Freitag and Benz (2002), Amaro-Seoane et al. (2004), Baumgardt et al. (2004a, b), Merritt and Vasiliev (2011), Vasiliev and Merritt (2013); and Vasiliev et al. (2014) in particular for the even more complex of binaries of massive black holes, in the context of the “final parsec problem”. The problem is further compounded, for example, by the presence of multiple stellar populations whose spatial distributions are segregated (“mass segregation”), with more massive stars sinking deeper into the potential well and approaching closer to the central black hole. Besides, two interacting stars may become gravitationally bound (become a binary) so that during the subsequent interactions with other stars or massive black holes they behave differently from single stars, or they may collide into each other, then the subsequent evolution will be determined by gas-dynamics. As these “micro-physical” effects are usually not incorporated into the global modeling of the entire nuclear star clusters, considerable uncertainties are attached to the theoretical predictions of the abundance and orbital parameters of the stars in the relativistic regime.

Whilst assuming sphericity will probably not have any impact on the estimate of capture rates, it is of huge relevance for “tidal processes”, since this is the region in which binary tidal separation and the tidal capture of giant cores will happen. For these processes the critical radius is beyond the influence radius of the central MBH and so triaxiality can probably play an important role. Due to computer power and the limitations of simulation codes, galactic nuclei have so far been modelled only as isolated spherical clusters with purely Newtonian gravity (e.g., Murphy et al. 1991; Freitag and Benz 2002). Vasiliev (2015) used the Princeton approach to derive a new Monte Carlo code, which presents a scheme to deal with asphericity (with other issues remaining open), with the limitation that it assumes isotropy of background stars population, so that it cannot model a highly flattened system with significant rotation support.

More realistic situations could only be explored with *N*-body methods or possibly with hybrid codes (Monte Carlo combined with *N*-body, for instance). While important approaches exist that implement small-number *N*-body integrations in the core of Monte Carlo, see Hypki and Giersz (2013), Fregeau and Rasio (2007), these approaches typically focus on binary scattering interactions, with less than five bodies. An important exception is the work of Rodriguez et al. (2015), which can integrate larger numbers, but it is limited to CPUs. Being based on KIRA (Portegies Zwart et al. 2001), it can in principle run on GRAPEs, a special-purpose chip to compute gravitational forces that was used in the past by many groups, see e.g., Fukushige et al. (2005), but (i) it is fair to say that these cards are obsolete, and virtually all efforts focus now on GPUs (there exists a library that can allow a GRAPE to mimic a GPU (Gaburov et al. 2009), but it is far from trivial to do it and in any case sub-optimal), (ii) it does not account for relativistic corrections, crucial to EMRI astrophysics, (iii) the code requires spherical symmetry and (iv) the code does not account for a central MBH.

**Fig. 63:**
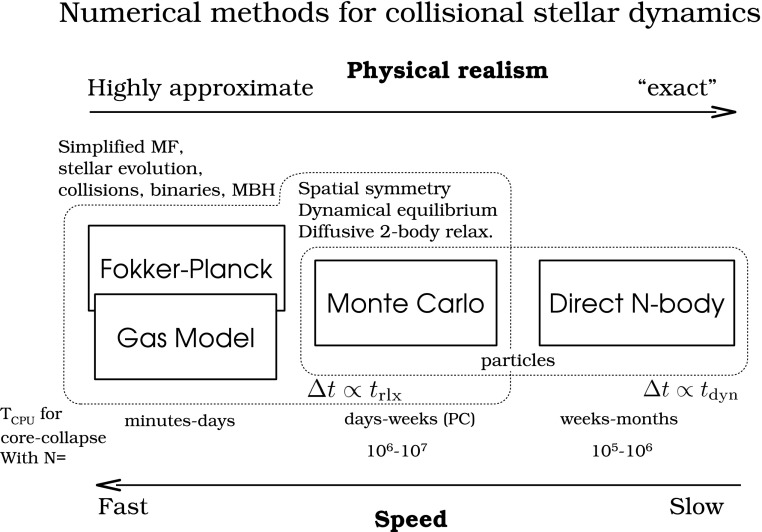
The various methods used to study collisional stellar dynamics. In the case of direct *N*-body computations, the simulations require the use of either special-purpose hardware such as the GRAPEs, Beowulf clusters for the parallel version of Rainer Spurzem or Graphical Computing Units (GPUs). A version of the parallel code *N*-body6++ ported to GPU architecture has been developed, see Wang et al. (2015, 2016), which allows us to simulate more realistic particles numbers

Figure 63 shows a schematic illustration of the current available codes for stellar dynamics including relaxation. The *physical realism* of the codes increases from the left to the right while the computational speed decreases. The two-dimensional numerical direct solutions of the Fokker–Planck equation (Takahashi 1997, 1996, 1995) probably require the least computational time, but these are followed closely by the gaseous model. The idea behind it is to treat two-body relaxation as a transport process such as in a conducting plasma (Hachisu et al. 1978; Lynden-Bell and Eggleton 1980). Multi-mass models have been implemented(Louis and Spurzem 1991; Spurzem 1992; Giersz and Spurzem 1994; Spurzem and Takahashi 1995) and improved for the detailed form of the conductivities by comparing to direct *N*-body models (described below). The addition of a central accreting MBH and a treatment for loss-cone effects was done by Amaro-Seoane et al. (2004) (a comprehensive description of the code is in the appendix of the same work) for the single-mass case, and also for a stellar mass spectrum (Amaro-Seoane 2004). The advantage of these two codes is the computational time required to perform a simulation (typically of the order of one minute on a regular PC for a Hubble time) and since they are not particle-based, the resolution can be envisaged as infinite, so that they are not limited by the particle number of the system and there is practically no numerical noise. Nevertheless, although they should be envisaged as powerful tools to make an initial, fast exploration of the parameter space, the results give us *tendencies* of the system, rather than an accurate answer (Amaro-Seoane 2004). Studying the astrophysical I/EMRI problem requires a meticulous characterisation of the orbital parameters, so that approximate techniques should be regarded as exploratory only (de Freitas Pacheco et al. 2006).

### The Fokker–Planck approach

Instead of tracking the individual motion of a large number of particles, as in *N*-body methods, one can attempt to describe a system consisting of a very large number of stars through the *1-particle phase-space distribution function* (DF for short) $f(\mathbf {x},\mathbf {v},t)$. The best interpretation of *f*, with the proviso that it has been properly normalised, is as a probability density if it is normalised to 1—$f(\mathbf {x},\mathbf {v},t)d^3x\,d^3v$ is the probability of finding, at time *t*, any given particle within the volume of phase space $d^3x\,d^3v$ around the 6-D phase-space point $(\mathbf {x},\mathbf {v})$; the average number of particles in this volume would be ${N_{\star }}f(\mathbf {x},\mathbf {v},t)d^3x\,d^3v$, with ${N_{\star }}$ the total number of particles. If the particles move in a common smooth potential $\varPhi$, the evolution of *f* is described by the collisionless Boltzmann equation (Binney and Tremaine 1987):

102 $$\begin{aligned} D_{t}f \equiv \frac{\partial {f}}{\partial {t}}+\mathbf {v}\cdot \mathbf {\nabla }f-\mathbf {\nabla }\varPhi \cdot \frac{\partial {f}}{\partial {\mathbf {v}}}=0. \end{aligned}$$

$\varPhi$ is obtained from *f* and a possible external potential $\varPhi _\mathrm{ext}$ (such as the one produced by a central MBH) from the Poisson equation.

In a real self-gravitating *N*-particle system the potential cannot be smooth on small scales but has some graininess, i.e., short-term, small-scale fluctuations, $\varPhi _\mathrm{real}=\varPhi +\varDelta \varPhi _\mathrm{grainy}$. Relaxation describes the effects of these fluctuations on *f*. They arise because a given particle sees the rest of the system as a collection of point masses rather than as a smooth mass distribution. Relaxational effects, also known (somewhat confusingly) as collisional effects, can therefore be seen as particles influencing each other individually as opposed as to collectively. To allow for these effects, a *collision term* has to be introduced on the right-hand side of the Boltzmann equation, in Eq. (102). This equation cannot be equated to zero if we want to take into account relaxational effects. The Fokker–Planck (FP) equation is derived by assuming that relaxation is due to a large number of 2-body gravitational encounters, each of which leads to a small deflection and occurs “locally”, i.e., they affect the velocity of a star without affecting its position. This is the basis for Chandrasekhar’s theory of relaxation (Chandrasekhar 1960; Binney and Tremaine 1987; Spitzer Jr 1987). To take care of encounters between stars, hence, we have to equate Eq. (102) not to zero, but to a collision term,

103 $$\begin{aligned} D_{t}f = -\sum _{i=1}^{3} \frac{\partial {}}{\partial {v_i}}\left[ f(\mathbf {x},\mathbf {v})\langle {\varDelta v_i}\rangle \right] + \frac{1}{2}\sum _{i,j=1}^{3} \frac{\partial ^2}{\partial v_i\partial v_j}\left[ f(\mathbf {x},\mathbf {v})\langle {\varDelta v_i \varDelta v_j}\rangle \right] , \end{aligned}$$

where the “diffusion coefficient” $\langle {\varDelta v_i}\rangle$ is the average change in $v_i$ per unit of time due to encounters (see Rosenbluth et al. 1957; Binney and Tremaine 1987 for a derivation).

From Jeans’ theorem (Jeans 1915; Merritt 1999), for a spherical system in dynamical equilibrium, the DF *f* can depend on the phase-space coordinates $(\mathbf {x},\mathbf {v})$ only through the (specific) orbital binding energy *E* and angular momentum (in modulus) *J*,

104 $$\begin{aligned} f(\mathbf {x},\mathbf {v}) = F(E(\mathbf {x},\mathbf {v}),J(\mathbf {x},\mathbf {v})). \end{aligned}$$

In the vast majority of applications, the Fokker–Planck formalism is applied in the two-dimensional (*E*, *J*)-space or, assuming isotropy, the one-dimensional energy-space rather than the six-dimensional phase space, through the operation of “orbit averaging” (see Cohn 1979, 1980, 1985; Spitzer Jr 1987 amongst others).

A standard form of the FP equation for an isotropic, spherical system is

105 $$\begin{aligned} D_t N(E) \equiv \frac{\partial {N}}{\partial {t}} + \frac{\partial {N}}{\partial {E}}\left. \frac{dE}{dt}\right| _{\phi } = -\frac{\partial {{\mathscr {F}}_E}}{\partial {E}} \end{aligned}$$

where

106 $$\begin{aligned} {\mathscr {F}}_E=m{\mathscr {D}}_{E}F-{\mathscr {D}}_{EE}\frac{\partial {F}}{\partial {E}} \end{aligned}$$

is the flux of particles in the energy space; $\left. {dE}/{dt}\right| _{\phi }$ is the change of energy due to the evolution of the potential $\phi$; *N*(*E*) is the density of stars in $E-$space,

107 $$\begin{aligned} N(E) = 16\pi ^2 p(E) F(E) \end{aligned}$$

with

108 $$\begin{aligned} p(E)=\int _0^{r_{\max }}r^2v\, dr. \end{aligned}$$

The “flux coefficients” are

109 $$\begin{aligned} {\mathscr {D}}_{E}&= 16\pi ^3\lambda m_\mathrm{f} \int _{\phi (0)}^E dE'p(E')F_\mathrm{f}(E'), \end{aligned}$$

110 $$\begin{aligned} {\mathscr {D}}_{EE}&= 16\pi ^3\lambda m_\mathrm{f}^2 \Big [ q(E)\int _E^0 dE'F_\mathrm{f}(E') + \int _{\phi (0)}^E dE'q(E')F_\mathrm{f}(E') \Big ], \end{aligned}$$

where $\lambda \equiv 4\pi G^2 \ln \varLambda$ and $q(E)=\frac{1}{3}\int _0^{r_{\max }} r^2v^3\, dr$ is the volume of phase space accessible to particles with energies lower than energy, and $p(E)={\partial q}/{\partial E}$ (Goodman 1983).

We use an index “f” for “field” to distinguish the mass and DF of the population we follow (“test-stars”) from the “field” objects. This distinction does not apply to a single-component system but it is easy to generalise to a multi-component situation by summing over components to get the total flux coefficient

111 $$\begin{aligned} {\mathscr {D}}_{E} = \sum _{l=1}^{N_\mathrm{comp}} {{\mathscr {D}}_{E}}_{,l},\ \ {\mathscr {D}}_{EE} = \sum _{l=1}^{N_\mathrm{comp}} {{\mathscr {D}}_{EE}}_{,l}, \end{aligned}$$

where the flux coefficient for component *l* can written by replacing the subscript “f” by “*l*” in Eq. (110).

I now explain schematically how the FP equation is implemented numerically to follow the evolution of star clusters. A more detailed description can be found in, e.g., Chernoff and Weinberg (1990). In the most common scheme, pioneered by Cohn (1980), two types of steps are employed alternately, a method known as “operator splitting”:

1. **Diffusion step**. The change in the distribution function *F* for a discrete time-step $\varDelta t$ is computed by using the FP equation *assuming the potential* $\phi$ *is fixed*, i.e., setting $D_t N = {\partial N}/{\partial t} = \left. {\partial N}/{\partial t}\right| _\mathrm{coll}$. The FP equation is discretized on an energy grid. The flux coefficients are computed using the DF(s) of the previous step; this makes the equations linear in the values of *F* on the grid points. The finite-differencing scheme is the implicit Chang and Cooper (1970) algorithm, based on a finite difference scheme for initial value problems, which is first order in time and energy.
2. **Poisson step**. Now, the change of potential resulting from the modification in the DF *F* is computed and *F* is modified to account for the term $\left. dE/dt\right| _\phi$, i.e., assuming $D_t N = {\partial N}/{\partial t} + {\partial N}/{\partial E}\left. {dE}/{dt}\right| _{\phi } = 0$. This can be done implicitly because, as long as the change in $\phi$ over $\varDelta t$ is very small, the actions of each orbit are adiabatic invariants. Hence, during the Poisson step, the distribution function, expressed in terms of the actions, does not change. In practice, an iterative scheme is used to compute the modified potential, determined implicitly by the modified DF, through the Poisson equation. The iteration starts with the values of $\phi$, $\rho$, etc. computed before the previous diffusion step.

A variant of the FP equation analogous to Eq. (105) can be written, which allows for anisotropy by taking into account the dependence of *F* on angular momentum and including a angular momentum-flux and corresponding flux coefficients (Cohn and Kulsrud 1978; Cohn 1979, 1985; Takahashi 1995, 1996, 1997; Drukier et al. 1999). The expressions for the flux coefficients are significantly longer than in the isotropic case and I do not present them here. However, we note that in galactic nuclei, in contrast to globular clusters, anisotropy plays a key role because of the existence of a loss cone.

The use of the FP approach to determine the distribution of stars around a MBH requires a few modifications. First the (Keplerian) contribution of the MBH to the potential has to be added. Several authors have made use of the FP or similar formalisms to study the dynamics well within the influence radius under the assumption of a fixed potential (Bahcall and Wolf 1976, 1977; Lightman and Shapiro 1977; Cohn and Kulsrud 1978; Hopman and Alexander 2006b, a; Merritt et al. 2006), which is a significant simplification. The static potential included a contribution for the stellar nucleus in the last study (Merritt et al. 2006) but was limited to a Keplerian MBH potential in the other cases. The presence of the MBH also constitutes a central sink as stars are destroyed or swallowed if they come very close to it. This has to be implemented into FP codes as a boundary condition. Lightman and Shapiro (1977) and Cohn and Kulsrud (1978) have developed detailed (and rather complex) treatments of the loss cone for the anisotropic FP formalism. It can be used in a simplified way in an isotropic FP analysis (Bahcall and Wolf 1977) to obtain a good approximation to the distribution of stars around a MBH and of the rates of consumption of stars by the MBH. However, additional analysis is required to determine what fraction of the swallowed stars are EMRIs and what their orbital properties are (Hopman and Alexander 2005, 2006a).

### Moment models

Another way to approximately solve the (collisional) Boltzmann equation is to take velocity moments of it. The moment or order $n=0$ of the DF is the density, the moments of order $n=1$ are bulk velocities and $n=2$ corresponds to (anisotropic) pressures (or velocity dispersions). This is analogous to the derivation of the Jeans equation from the collisionless Boltzmann equation (Binney and Tremaine 1987) but the collision term introduces moments of order $n+1$ in the equations for moments of order *n*.

In statistical moment models, we employ velocity moments to characterise the local velocity distribution function. The *n*-th moment of a velocity distribution *f*(*v*) is defined as $\langle v^n \rangle = \int (v)^n\,f(v) \,\, \mathrm {d}v$. The accuracy of these models is then limited by the order of the highest moment included to describe the velocity distribution, as discussed in detail in Schneider et al. (2011).

**Fig. 64:**
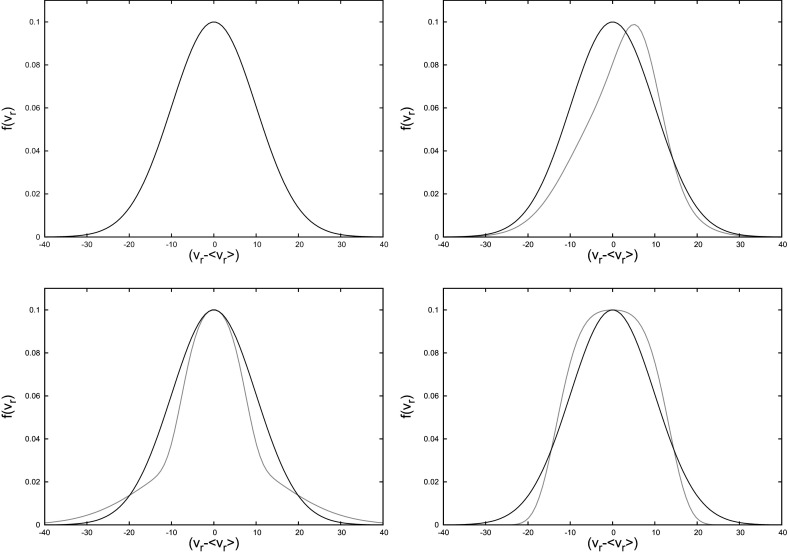
One-dimensional velocity distribution functions for different cases. *Upper panels:* from the left to the right, I first show the Gaussian velocity distribution describing thermodynamical equilibrium with a variance of $\sigma =10~\text {km}~{\text {s}^{-1}}$. The Gaussian appears in the subsequent panels for comparison (black). On the right, velocity distribution (grey) with a skewness in positive $v_r$-direction indicating energy flow in $v_r$-direction. *Lower panels:* two velocity distributions (grey) with an excess and the deficit of high velocity stars, respectively, as compared to a situation of thermodynamical equilibrium

Since each stellar dynamical process driving the evolution of a cluster has a different impact on the local velocity distribution, this motivates us to construct a distribution function that is able to reflect the effects of each of these processes properly so as not to lose information that influences the clusters evolution. The velocity distribution can be written as a series expansion using a *truncated Gauss–Hermite series*, as in Gerhard (1993), van der Marel and Franx (1993) to illustrate the meaning of the first four moments:

112 $$\begin{aligned} f(v_r) \propto \exp \left( -\frac{v_r-\bar{v}_r}{2\sigma }\right) \left[ 1+\sum ^4_{k=3}h_k H_k(v_r-\bar{v}_r)\right] , \end{aligned}$$

where $H_k$ are the Hermite polynomials (see, e.g., the Appendix A of van der Marel and Franx 1993), $v_r$ the velocity in radial direction (or the line-of-sight velocity which is the velocity measured in direction of an observer), and $\bar{v}_r$, $\sigma$, $h_3$ and $h_4$ are free parameters. The first moments can be related to physical properties of the system that we are studying:

*0th moment:* The zeroth moment of a velocity distribution is 1 due to normalisation.
*1st moment:* The first moment of a velocity distribution is the mean velocity $\bar{v}_r$ and denotes the bulk mass transport velocity.
*2nd moment:* The second moment of a velocity distribution is the variance $\sigma$ and is equal to the velocity dispersion. It determines the width of $f(v_r)$ and thus the scattering of stellar velocities around the mean velocity $\bar{v}_r$. If $f(v_r)$ is fully determined by $\bar{v}_r$ and $\sigma$ and $h_3=h_4=0$ it is a Gaussian (upper left panel in Fig. 64) corresponding to thermal equilibrium. Then the symmetry of the one-dimensional velocity distribution $f(v_r)$ to $\bar{v}_r$ reflects isotropy.
*3rd moment:* The third moment, denotes the transport of random kinetic energy and depends on $h_3$. If the third moment of the velocity distribution does not vanish, implying that $h_3\ne 0$, then the shape of the velocity distribution is a skewed Gaussian (Fig. 64, upper right panel). The asymmetry indicates the direction of the energy flux, and the uneven distribution of velocities in different directions denotes anisotropy.
*4th moment:* The fourth moment is a measure of the excess or deficiency of particles/stars with high velocities as compared to thermodynamical equilibrium, and depends on the value of $h_4$. An excess of particles with high velocities results in thicker wings of the velocity distribution and a more pointed maximum (Fig. 64, lower left panel). A deficiency of high velocities causes a broader shape around the mean and thinner wings of the velocity distribution (Fig. 64, lower right panel).

Third and fourth-order moments therefore denote deviations from thermodynamical equilibrium. Modeling processes that lead to the transport of random kinetic energy in a cluster or that strongly affect the high velocity wings of the distribution suggest the use of a model that includes fourth-order moments. These processes are, for example, the “evaporation” of high velocity stars from the cluster, which reduces the number of high velocity stars. On the other hand, binaries and a mass spectrum transfer kinetic energy between different stellar components and thereby produce high velocity stars. These high velocity stars then transfer their excess energy to their environment in subsequent distant two-body encounters which can lead to a transport of kinetic energy between different regions in the GC.

The so-called “gaseous model”, is a particular case of moment models.^19^ In this approach, one assumes spherical symmetry (but not necessarily dynamical equilibrium) and stops the infinite set of moment equations at $n=2$. The system is closed with the assumption that energy exchanges between stars through 2-body relaxation can be approximated by an ad hoc (local) heat conduction prescription (Hachisu et al. 1978; Lynden-Bell and Eggleton 1980). This reduces the study of the stellar system to that of a self-gravitating conducting gas sphere. Multi-mass models have been implemented (Louis and Spurzem 1991; Spurzem 1992; Giersz and Spurzem 1994; Spurzem and Takahashi 1995) and the detailed forms for the conductivities have been improved by comparing to direct *N*-body models (described below). The addition of a central accreting MBH and a treatment for loss-cone effects was done in Amaro-Seoane et al. (2004) for the single-mass case (a comprehensive description of the code is in the appendix of the same work), and in Amaro-Seoane (2004) for a stellar mass spectrum.

The system is treated as a continuum, which is only adequate for a large number of stars and in well populated regions of the phase space. Here, I consider spherical symmetry and single-mass stars. We handle relaxation in the Fokker–Planck approximation, i.e., like a diffusive process determined by local conditions. We also make use of the hydrodynamical approximation; that is to say, only local moments of the velocity dispersion are considered, not the full orbital structure. In particular, the effect of the two-body relaxation can be modelled by a local heat flux equation with an appropriately tailored conductivity.

For our description I use polar coordinates, (*r* $\theta$, $\phi$). The vector $\mathbf{v} = (v_i), i=r,\theta ,\phi$ denotes the velocity in a local Cartesian coordinate system at the spatial point $r,\theta ,\phi$. For simplicity, I will employ the notation $u=v_\mathrm{r}$, $v=v_\theta$, $w=v_\phi$. The distribution function *f*, is a function of *r*, *t*, *u*, $v^2+w^2$ only due to spherical symmetry, and is normalised according to

113 $$\begin{aligned} \rho (r,t) = \int f(r,u,v^2+w^2,t) du\,dv\,dw. \end{aligned}$$

Here $\rho (r,t)$ is the mass density; if $m_{\star }$ denotes the stellar mass, we get the particle density $n=\rho /m_{\star }$. The Euler-Lagrange equations of motion corresponding to the Lagrange function

114 $$\begin{aligned} {\mathscr {L}} = {1\over 2}\bigl ({\dot{r}}^2 + r^2{\dot{\theta }}^2 + r^2 \sin ^2\!\!\theta \, {\dot{\phi }}^2\bigr ) - \varPhi (r,t) \end{aligned}$$

are the following

115 $$\begin{aligned}&{\dot{u}} =&- \frac{\partial \varPhi }{\partial r} + {v^2\!+\!w^2\over r}, \nonumber \\&{\dot{v}} =&- {uv\over r} + {w^2\over r\tan \theta }, \nonumber \\&{\dot{w}} =&- {uw\over r} - {vw\over r\tan \theta }. \end{aligned}$$

And so we get a complete local Fokker–Planck equation,

116 $$\begin{aligned} \frac{\partial f}{\partial t} +v_\mathrm{r}\frac{\partial f}{\partial r}+\dot{v_\mathrm{r}} \frac{\partial f}{\partial v_\mathrm{r}}+\dot{v_{\theta }}\frac{\partial f}{\partial v_{\theta }}+\dot{v_{\varphi }}\frac{\partial f}{\partial v_{\varphi }}= \left( \frac{\delta f}{\delta t} \right) _{FP}. \end{aligned}$$

In our model we do not solve the equation directly; we use a so-called *moments process*. The moments of the velocity distribution function *f* are defined as follows

117 $$\begin{aligned} {\langle }i,\,j,\,k{\rangle } := \int ^{+\infty }_{-\infty } (v_\mathrm{r})^{i} (v_{\theta })^{j} (v_{\phi })^{k} \,f(r, v_\mathrm{r}, v_{\theta },v_{\phi },t)\,dv_\mathrm{r}dv_{\theta }dv_{\phi }; \end{aligned}$$

Using the previous definition, I introduce now the following moments of the velocity distribution function,

118 $$\begin{aligned} {\langle }0,0,0{\rangle }&:= \rho = \int f\, du\,dv\,dw, \end{aligned}$$

119 $$\begin{aligned} {\langle }1,0,0{\rangle }&:= {u} = \int uf\, du\,dv\,dw,\end{aligned}$$

120 $$\begin{aligned} {\langle }2,0,0{\rangle }&:= p_\mathrm{r} + \rho {u}^2 =\int u^2 f\, du\,dv\,dw,\end{aligned}$$

121 $$\begin{aligned} {\langle }0,2,0{\rangle }&:= p_\theta = \int v^2 f\, du\,dv\,dw,\end{aligned}$$

122 $$\begin{aligned} {\langle }0,0,2{\rangle }&:= p_\phi = \int w^2 f\, du\,dv\,dw,\end{aligned}$$

123 $$\begin{aligned} {\langle }3,0,0{\rangle }&:= F_\mathrm{r} + 3{u}p_\mathrm{r} + {u}^3 =\int u^3 f\, du\,dv\,dw,\end{aligned}$$

124 $$\begin{aligned} {\langle }1,2,0{\rangle }&:= F_\theta + {u}p_\theta = \int uv^2 f\, du\,dv\,dw,\end{aligned}$$

125 $$\begin{aligned} {\langle }1,0,2{\rangle }&:= F_\phi + {u}p_\phi = \int uw^2 f\, du\,dv\,dw, \end{aligned}$$

where $\rho$ is the density of stars, *u* is the bulk velocity, $v_\mathrm{r}$ and $v_\mathrm{t}$ are the radial and tangential flux velocities,

126 $$\begin{aligned} v_\mathrm{r}&= {F_\mathrm{r} \over 3 p_\mathrm{r}} + u, \nonumber \\ v_\mathrm{t}&= {F_\mathrm{t} \over 2 p_\mathrm{t}} + u, \end{aligned}$$

$p_\mathrm{r}$ and $p_\mathrm{t}$ are the radial and tangential pressures, $F_\mathrm{r}$ is the radial and $F_\mathrm{t}$ the tangential kinetic energy flux (Louis and Spurzem 1991). Note that the definitions of $p_i$ and $F_i$ are such that they are proportional to the random motion of the stars. Due to spherical symmetry, we have $p_{\theta } = p_{\phi }=: p_\mathrm{t}$ and $F_{\theta } = F_{\phi } =: F_\mathrm{t}/2$. By $p_\mathrm{r} = \rho \sigma _\mathrm{r}^2$ and $p{_\mathrm{t}} = \rho \sigma _\mathrm{t}^2$ the random velocity dispersions are given, which are closely related to observable properties in stellar clusters.

$F = (F_\mathrm{r} + F_\mathrm{t})/2$ is a radial flux of random kinetic energy. In the notion of gas dynamics it is just an energy flux. Whilst for the $\theta -$ and $\phi -$ components in the set of Eqs. (125) are equal in spherical symmetry, for the *r* and *t*- quantities this is not true. In stellar clusters the relaxation time is larger than the dynamical time and so any possible difference between $p_\mathrm{r}$ and $p_\mathrm{t}$ may *survive* many dynamical times. We shall call such differences anisotropy. In case of *weak* isotropy ($p_\mathrm{r}$=$p_\mathrm{t}$), $2F_\mathrm{r}$ = $3F_\mathrm{t}$, and thus $v_\mathrm{r}$ = $v_\mathrm{t}$, i.e., the (radial) transport velocities of radial and tangential random kinetic energy are equal.

The Fokker–Planck equation (116) is multiplied by various powers of the velocity components *u*, *v*, *w*. We get up to second order we get a set of moment equations: A mass equation, a continuity equation, an Euler equation (force) and radial and tangential energy equations. The system of equations is closed by a phenomenological heat flux equation for the flux of radial and tangential RMS (*root mean square*) kinetic energy, both in radial direction. The concept is physically similar to that of Lynden-Bell and Eggleton (1980). The set of equations is

127 $$\begin{aligned} \frac{\partial {\rho }}{\partial t}&+ \frac{1}{r^2}\frac{\partial }{\partial r} (r^2u\,\rho )= 0,\nonumber \\ \frac{\partial u}{\partial t}&+u\,\frac{\partial u}{\partial r} + {GM_\mathrm{r}\over r^2} + {1\over \rho }\frac{\partial p_\mathrm{r}}{\partial r} + 2\,\frac{p_\mathrm{r} - p_\mathrm{t}}{\rho \, r} = 0,\nonumber \\ \frac{\partial {p_\mathrm{r}}}{\partial {t}}&+ \frac{1}{r^2} \frac{\partial }{\partial r} (r^2 u \,p_\mathrm{r})+2 \,p_\mathrm{r} \frac{\partial u}{\partial r} + \frac{1}{r^2} \frac{\partial }{\partial r} (r^2 F_\mathrm{r}) \frac{2F_\mathrm{t}}{r} = -\frac{4}{5} \frac{(2p_\mathrm{r}-p_\mathrm{t})}{\lambda _A t_\mathrm{rlx}}, \nonumber \\ \frac{\partial {p_\mathrm{t}}}{\partial {t}}&+ \frac{1}{r^2} \frac{\partial }{\partial r} (r^2 u \,p_\mathrm{t})+2 \,\frac{p_\mathrm{t}\,u}{r}+\frac{1}{2 r^2} \frac{\partial }{\partial r} (r^2F_\mathrm{t}) + \frac{F_\mathrm{t}}{r} = \frac{2}{5} \frac{(2p_\mathrm{r}-p_\mathrm{t})}{\lambda _A t_\mathrm{rlx}} , \end{aligned}$$

where $\lambda _A$ is a numerical constant related to the time-scale of collisional anisotropy decay. The value chosen for it has been discussed in comparison with direct simulations performed with the *N*–body code (Giersz and Spurzem 1994). The authors find that $\lambda _A=0.1$ is the physically realistic value inside the half-mass radius for all cases of *N*, provided that close encounters and binary activity do not carry out an important role in the system, this is, however, inherent to systems with a large number of particles, as this is.

With the definition of the mass $M_\mathrm{r}$ contained in a sphere of radius *r*

128 $$\begin{aligned} \frac{\partial M_\mathrm{r}}{\partial r} = 4 \pi r^2 \rho , \end{aligned}$$

the set of equations is then equivalent to gas-dynamical equations coupled with the equation of Poisson. To close it, we need an independent relation, for moment equations of order *n* contain moments of order $n\,+\,1$. For this, I use the heat conduction closure, a phenomenological approach obtained in an analogous way to gas dynamics. It was used for the first time by Lynden-Bell and Eggleton (1980) but restricted to isotropy. In this approximation one assumes that heat transport is proportional to the temperature gradient,

129 $$\begin{aligned} F = -\kappa \frac{\partial T}{\partial r} = -\varLambda \frac{\partial \sigma ^2}{\partial r} \end{aligned}$$

That is the reason why such models are usually also called *conducting gas sphere models*.

It has been argued that for the classical approach $\varLambda \propto \bar{\lambda }^2/\tau$, one has to choose the Jeans’ length $\lambda _J^2 = \sigma ^2/(4\pi G\rho )$ and the standard Chandrasekhar local relaxation time $t_\mathrm{rlx}\propto \sigma ^3/\rho$ (Lynden-Bell and Eggleton 1980), where $\bar{\lambda }$ is the mean free path and $\tau$ the collisional time. In this context we obtain a conductivity $\varLambda \propto \rho / \sigma$. We shall consider this as a working hypothesis. For the anisotropic model we use a mean velocity dispersion $\sigma ^2 = (\sigma _\mathrm{r}^2 + 2\sigma _\mathrm{t}^2)/3$ for the temperature gradient and assume $v_\mathrm{r} = v_\mathrm{t}$ (Bettwieser and Spurzem 1986).

Therefore, the equations we need to close our model are

130 $$\begin{aligned} v_\mathrm{r}&- u + \frac{\lambda }{4\pi \,G\rho \,t_\mathrm{rlx}} \frac{\partial \sigma ^2}{\partial r} = 0, \nonumber \\ v_\mathrm{r}&= v_\mathrm{t}. \end{aligned}$$

I now introduce the interaction terms to be added to right hand of the component equations.

#### Equation of continuity

I now modify the star continuity equation to include the interaction terms (Langbein et al. 1990). The equation

131 $$\begin{aligned} \frac{\partial \rho _{\star }}{\partial \mathrm{t}}+ \frac{1}{\mathrm{r}^2} \frac{\partial }{\partial \mathrm{r}}(\mathrm{r^2} \rho _{\star } \mathrm{u_{\star }})=0, \end{aligned}$$

becomes

132 $$\begin{aligned} \frac{\partial \rho _{\star }}{\partial \mathrm{t}} + \frac{1}{\mathrm{r}^2} \frac{\partial }{\partial \mathrm{r}}(\mathrm{r^2} \rho _{\star } \mathrm{u_{\star }})= \left( \frac{\delta \rho _{\star }}{\delta \mathrm{t}} \right) _\mathrm{coll}+\left( \frac{\delta \rho _{\star }}{\delta \mathrm{t}} \right) _\mathrm{lc}, \end{aligned}$$

where the term on the right-hand side reflects the time variation of the star’s density due to stellar interactions (i.e., due to the calculation of the mean rate of gas production by stars’ collisions) and loss-cone (stars plunging onto the central object).

If $f(v_\mathrm{rel})$ is the stellar distribution of relative velocities, then the mean rate of gas production by stellar collisions is

133 $$\begin{aligned} \left( \frac{\delta \rho _{\star }}{\delta \mathrm{t}} \right) _\mathrm{coll}&= -\int _{|v_\mathrm{rel|}>\sigma _\mathrm{coll}} \frac{\rho _{\star }f_\mathrm{c}(v_\mathrm{rel})}{t_\mathrm{coll}} f(v_\mathrm{rel})\,d^3v_\mathrm{rel}, \end{aligned}$$

134 $$\begin{aligned} \left( \frac{\delta \rho _{\star }}{\delta \mathrm{t}} \right) _\mathrm{coll}&= -\int _{|v_\mathrm{rel|}>\sigma _\mathrm{coll}} \frac{\rho _{\star }f_\mathrm{c}(v_\mathrm{rel})}{t_\mathrm{coll}} f(v_\mathrm{rel})d^3v_\mathrm{rel}, \end{aligned}$$

where $f(v_\mathrm{rel})$ is a Schwarzschild - Boltzmann distribution,

135 $$\begin{aligned} f(v_\mathrm{rel}) =\frac{1}{2 \pi ^{3/2} \sigma _\mathrm{r} \sigma _\mathrm{t}^2} \, \mathrm{exp}\,\left[ -\frac{(v_\mathrm{rel,r}-u_{\star })^2}{4 \sigma _\mathrm{r}^2}-\frac{v_\mathrm{rel,t}^2}{2 \sigma _\mathrm{t}^2} \right] . \end{aligned}$$

With regards to $f_\mathrm{c}$, it is the relative fraction of mass liberated per stellar collision into the gaseous medium. Under certain assumptions given in the initial work of Spitzer Jr and Saslaw (1966), we can calculate it as an average over all impact parameters resulting in $r_{\min }<2\,r_{\star }$ and as a function of the relative velocity at infinity of the two colliding stars, $v_\mathrm{rel}$. Langbein et al. (1990) approximate their result by

136 $$\begin{aligned} f_\mathrm{c}(v_\mathrm{rel}) = \left\{ \begin{array}{ll} \left( 1+q_\mathrm{coll} \sqrt{\frac{\sigma _\mathrm{coll}}{v_\mathrm{rel}}} \right) ^{-1}&{} v_\mathrm{rel} > \sigma _\mathrm{coll},\\ 0 &{} v_\mathrm{rel} < \sigma _\mathrm{coll}, \end{array} \right. \end{aligned}$$

with $q_\mathrm{coll}=100$. Hence, we have that

137 $$\begin{aligned} f_\mathrm{c}(v_\mathrm{rel}) = \left\{ \begin{array}{ll} 0.01 &{} \sigma _\mathrm{coll}=v_\mathrm{rel},\\ 0 &{} \sigma _\mathrm{coll}>v_\mathrm{rel}. \end{array} \right. \end{aligned}$$

The first interaction term is

138 $$\begin{aligned} \left( \frac{\delta \rho _{\star }}{\delta \mathrm{t}} \right) _\mathrm{coll} =- \rho _{\star } \frac{\mathrm{f}_\mathrm{c}}{\mathrm{t}_\mathrm{coll}} \left[ 1-{\mathrm{erf}} \left( \frac{\sigma _\mathrm{coll}}{\sqrt{6}\sigma _\mathrm{r}} \right) \right] \left[ 1-\mathrm{erf} \left( \frac{\sigma _\mathrm{coll}}{\sqrt{6}\sigma _\mathrm{t}} \right) \right] ^2, \end{aligned}$$

which, for simplification, we call it

139 $$\begin{aligned} \left( \frac{\delta \rho _{\star }}{\delta \mathrm{t}} \right) _\mathrm{coll}\equiv -\rho _{\star } \mathrm{X_\mathrm{coll}}. \end{aligned}$$

Since the evolution of the system that we are studying can be regarded as stationary, I introduce for each equation the “logarithmic variables” in order to study the long-term evolution. On the other hand, if the system happens to have quick changes, we should use the “non-logarithmic” version of the equations. For this reason, I will write at the end of each subsection the equation in terms of the logarithmic variables.

In the case of the equation of continuity, I develop it and divide it by $\rho _{\star }$ because we are looking for the logarithm of the star density, $\partial \ln \rho _{\star } /\partial t=(1/\rho _{\star })\partial \rho _{\star }/ \partial t$. The result is:

140 $$\begin{aligned} \frac{\partial \ln \rho _{\star }}{\partial t} + \frac{\partial u_{\star }}{\partial r}+ u_{\star } \frac{\partial \ln \rho _{\star }}{\partial r}+ \frac{2u_{\star }}{r}= \frac{1}{\rho _{\star }}\left( \frac{\delta \rho _{\star }}{\delta \mathrm{t}} \right) _\mathrm{coll}+ \frac{1}{\rho _{\star }}\left( \frac{\delta \rho _{\star }}{\delta \mathrm{t}} \right) _\mathrm{lc} \end{aligned}$$

#### Momentum balance equation

The second equation has the following star interaction terms:

141 $$\begin{aligned} \frac{\partial u_{\star }}{\partial t} +u_{\star } \frac{\partial u_{\star }}{\partial r} + {GM_\mathrm{r}\over r^2} + {1\over \rho _{\star }}\frac{\partial p_\mathrm{r}}{\partial r} + 2\,{p_\mathrm{r} - p_\mathrm{t}\over \rho _{\star } r} = \left( \frac{\delta u_{\star }}{\delta t}\right) _\mathrm{drag}. \end{aligned}$$

The interaction term is due to the decelerating force that stars moving inside the gas are subject.

142 $$\begin{aligned} \left( \frac{\delta u_{\star }}{\delta t}\right) _\mathrm{drag} = - X_\mathrm{drag}\frac{1}{\rho _{\star }} (u_{\star }-u_\mathrm{g}), \end{aligned}$$

where I have introduced the following definition:

143 $$\begin{aligned} X_\mathrm{drag} \equiv -C_{D} \frac{\pi r_{\star }^2}{m_{\star }}\rho _{\star } \rho _\mathrm{g} \sigma _\mathrm{tot}, \end{aligned}$$

with $\sigma _\mathrm{tot}^2=\sigma _\mathrm{r}^2+\sigma _\mathrm{t}^2+(u_{\star }-u_\mathrm{g})^2$. In the “gaseous model”, I use a logarithmic expression of the equation, so that we multiply Eq. (141) by $\rho _{\star } r/p_\mathrm{r}$:

144 $$\begin{aligned} \frac{\rho _{\star } r}{p_\mathrm{r}} \left( \frac{\partial u_{\star }}{\partial t}+ u_{\star } \right) + \frac{GM_\mathrm{r}}{rp_\mathrm{r}}\rho _{\star }+\frac{\partial \ln p_\mathrm{r}}{\partial \ln r}+ 2\,\left( 1-\frac{p_\mathrm{t}}{p_\mathrm{r}}\right) =-X_\mathrm{drag} \frac{r}{p_\mathrm{r}}\left( u_{\star }-u_\mathrm{g}\right) . \end{aligned}$$

#### Radial energy equation

Regarding the penultimate equation, the interaction terms are:

145 $$\begin{aligned}&\frac{\partial {p_\mathrm{r}}}{\partial {t}} + \frac{1}{r^2} \frac{\partial }{\partial r} (r^2 u_{\star } p_\mathrm{r})+2 p_\mathrm{r} \frac{\partial u_{\star }}{\partial r}+ \frac{4}{5} \frac{(2p_\mathrm{r}-p_\mathrm{t})}{t_\mathrm{rlx}} + \frac{1}{r^2} \frac{\partial }{\partial r} (r^2 F_\mathrm{r})- \frac{2F_\mathrm{t}}{r}\nonumber \\&\quad = \left( \frac{\delta p_\mathrm{r}}{\delta t}\right) _\mathrm{drag}+\left( \frac{\delta p_\mathrm{r}}{\delta t}\right) _\mathrm{coll}, \end{aligned}$$

where

146 $$\begin{aligned} \left( \frac{\delta p_\mathrm{r}}{\delta t}\right) _\mathrm{drag}&=-2X_\mathrm{drag} \sigma _\mathrm{r}^2, \nonumber \\ \left( \frac{\delta p_\mathrm{r}}{\delta t}\right) _\mathrm{coll}&=-X_\mathrm{coll} \rho _{\star } \tilde{\sigma _\mathrm{r}}^2 \epsilon . \end{aligned}$$

In order to determine $\epsilon$, I introduce the ratio *k* of kinetic energy of the remaining mass after the encounter over its initial value (before the encounter); *k* is a measure of the inelasticity of the collision: for $k=1$ we have the minimal inelasticity, just the kinetic energy of the liberated mass fraction is dissipated, while if $k<1$ a surplus amount of stellar kinetic energy is dissipated during the collision (tidal interactions and excitation of stellar oscillations). If we calculate the energy loss in the stellar system per unit volume as a function of *k*, we obtain

147 $$\begin{aligned} \epsilon =f_\mathrm{c}^{-1}[1-k(1-f_\mathrm{c})]. \end{aligned}$$

We divide by $p_{r }$ so that we get the logarithmic variable version of the equation. We also make the following substitution:

148 $$\begin{aligned} F_\mathrm{r}&= 3p_\mathrm{r}v_\mathrm{r}, \nonumber \\ F_\mathrm{t}&= 2p_\mathrm{t}v_\mathrm{t}. \end{aligned}$$

The resulting equation is

149 $$\begin{aligned}&\frac{\partial \ln p_\mathrm{r}}{\partial t} + (u_{\star }+3v_\mathrm{r}) \frac{\partial \ln p_\mathrm{r}}{\partial r} +3 \left( \frac{\partial u_{\star }}{\partial r}+\frac{\partial v_\mathrm{r}}{\partial r} \right) + \frac{2}{r}\left( u_{\star }+3v_\mathrm{r}-2v_\mathrm{t} \frac{p_\mathrm{t}}{p_\mathrm{r}} \right) \nonumber \\&\quad + \frac{4}{5}\frac{ 2-{p_\mathrm{t}}/{p_\mathrm{r}}}{t_\mathrm{rlx}} =\frac{1}{p_\mathrm{r}} \left( \frac{\delta p_\mathrm{r}}{\delta t}\right) _\mathrm{drag}+\frac{1}{p_\mathrm{r}} \left( \frac{\delta p_\mathrm{r}}{\delta t}\right) _\mathrm{coll}. \end{aligned}$$

#### Tangential energy equation

To conclude the set of equations of the star component with the interaction terms, we have the following equation:

150 $$\begin{aligned} \frac{\partial {p_\mathrm{t}}}{\partial {t}}&+ \frac{1}{r^2} \frac{\partial }{\partial r} \left( r^2 u_{\star } p_\mathrm{t}\right) + 2\, \frac{p_\mathrm{t}u_{\star }}{r}- \frac{4}{5} \frac{(2p_\mathrm{r}-p_\mathrm{t})}{t_\mathrm{rlx}}+ \frac{1}{r^2} \frac{\partial }{\partial r}(r^2F_\mathrm{t})+\frac{2F_\mathrm{t}}{r} = \nonumber \\&\left( \frac{\delta p_\mathrm{t}}{\delta t}\right) _\mathrm{drag}+\left( \frac{\delta p_\mathrm{t}}{\delta t}\right) _\mathrm{coll}, \end{aligned}$$

where

151 $$\begin{aligned} \left( \frac{\delta p_\mathrm{t}}{\delta t}\right) _\mathrm{drag}&=-2X_\mathrm{drag} \sigma _\mathrm{t}^2\nonumber \\ \left( \frac{\delta p_\mathrm{t}}{\delta t}\right) _\mathrm{coll}&=-X_\mathrm{coll} \rho _{\star } \tilde{\sigma _\mathrm{t}}^2 \epsilon . \end{aligned}$$

We follow the same path like in the last case and so:

152 $$\begin{aligned}&\frac{\partial \ln p_\mathrm{t}}{\partial t} + (u_{\star }+2v_\mathrm{t}) \frac{\partial \ln p_\mathrm{t}}{\partial r} +\frac{\partial }{\partial r}(u_{\star }+2v_\mathrm{t})+ \frac{4}{r}\left( u_{\star }+2v_\mathrm{t}\right) - \frac{4}{5} \frac{2{p_\mathrm{r}}/{p_\mathrm{t}}-1}{t_\mathrm{rlx}}\nonumber \\&\quad =\frac{1}{p_\mathrm{t}} \left( \frac{\delta p_\mathrm{t}}{\delta t}\right) _\mathrm{drag}+ \frac{1}{p_\mathrm{t}} \left( \frac{\delta p_\mathrm{t}}{\delta t}\right) _\mathrm{coll}. \end{aligned}$$

### Solving conducting, self-gravitating gas spheres

In this subsection, I explain briefly how the gaseous model is solved. The algorithm used is a partially implicit Newton–Raphson–Henyey iterative scheme, see Henyey et al. (1959), Kippenhahn and Weigert (1994), their Sect. 11.2.

Putting aside the bounding conditions, the set of equations to be solved are Eq. (127) to (130). In practice, however, the equations are rewritten using the logarithm of all positive quantities as dependant functions. As explained in Giersz and Spurzem (1994), this greatly improves energy conservation. Formally, one may write this system as follows

153 $$\begin{aligned} \frac{\partial x^{(i)}}{\partial t} + f^{(i)}\left( \left\{ x^{(j)},\frac{\partial x^{(j)}}{\partial r}\right\} _{j=1}^{N_\mathrm{eq}} \right) =0,&\ \ \text{ for }\ i=1\cdots 4 \nonumber \\ f^{(i)}\left( \left\{ x^{(j)},\frac{\partial x^{(j)}}{\partial r}\right\} _{j=1}^{N_\mathrm{eq}} \right) =0,&\ \ \text{ for }\ i=5\cdots N_\mathrm{eq}, \end{aligned}$$

where the $x^{(i)}$ are the local quantities defining the state of the cluster, i.e.,

154 $$\begin{aligned} \underline{x} \equiv \left\{ x^{(1)}, x^{(2)}, \ldots o x^{(N_\mathrm{eq})}\right\} \equiv \{ \log \rho ,\, u,\, \log p_\mathrm{r},\, \log p_\mathrm{\, t},\, \log M_\mathrm{r},\, v_\mathrm{r}-u,\, v_\mathrm{t}-u \}, \end{aligned}$$

with $N_\mathrm{eq}=7$ in the present application.

To be solved numerically, this set of coupled partial differential equations have to be discretized according to time and radius. Let us first consider time stepping. Let $\varDelta t$ be the time step. Assume we know the solution $\underline{x}(t-\varDelta t)$ at time $t-\varDelta t$ and want to compute $\underline{x}(t)$. For the sake of numerical stability, a partially implicit scheme is used. I adopt the shorthand notations $x^{(i)} \equiv x^{(i)}(t)$ and $y^{(i)} \equiv x^{(i)}(t-\varDelta t)$. Time derivation is replaced by finite differences,

155 $$\begin{aligned} \frac{\partial x^{(i)}}{\partial t} \rightarrow \varDelta t^{-1}(x^{(i)}-y^{(i)}). \end{aligned}$$

In the terms $f^{(i)}$, I replace the $x^{(j)}$ by $\tilde{x}^{(j)}$ which are intermediate values between $y^{(j)}$ and $x^{(j)}$, $\tilde{x}^{(j)} = \zeta x^{(j)} + (1-\zeta )y^{(j)}$, with $\zeta = 0.55$ for stability purposes (Giersz and Spurzem 1994).

Spatial discretisation is done by defining all quantities (at a given time) on a radial mesh, $\{r_{1}, r_{2}, \ldots o r_{N_\mathrm{r}}\}$ with $r_{1}=0$ and $r_{N_\mathrm{r}}=r_{\max }$. A staggered mesh is implemented. While values of *r*, *u*, $v_\mathrm{t}$, $v_\mathrm{r}$ and $M_\mathrm{r}$ are defined at the boundaries of the mesh cells, $\rho$, $p_\mathrm{t}$ and $p_\mathrm{r}$ are defined at the centre of each cell. When the value of a “boundary” quantity is needed at the centre of a cell, or vice-versa, one does a simple averaging, i.e., $\hat{b}_{k} = (b_{k-1}+b_{k})/2$, $\hat{c}_{k} = (c_{k}+c_{k+1})/2$. Let us adopt the notation $x^{(j)}_{k}$ for the value of $x^{(j)}$ at position $r_{k}$ (or $\hat{r}_{k}$) and $\varDelta r_{k}\equiv r_{k}-r_{k-1}$. Then, radial derivatives in the terms $f^{(i)}$ are approximated by finite differences,

156 $$\begin{aligned} \frac{\partial x^{(j)}}{\partial r} \rightarrow \frac{\tilde{{x}}^{(j)}_{k}-\tilde{{x}}^{(j)}_{k-1}}{\varDelta r_{k}}, \end{aligned}$$

if the derivative has to be evaluated at a point where $x_{k}$ is defined (centre or border of a cell), or

157 $$\begin{aligned} \frac{\partial x^{(j)}}{\partial r} \rightarrow \frac{\hat{\tilde{{x}}}^{(j)}_{k}-\hat{\tilde{{x}}}^{(j)}_{k-1}}{\varDelta r_{k}} = \frac{\tilde{x}^{(j)}_{k+1}-\tilde{x}^{(j)}_{k-1}}{2\varDelta r_{k}}, \end{aligned}$$

otherwise. As an exception, I use upstream differencing in $\partial {u}/\partial {r}$ for the second equation in Eq. (127), i.e., the difference quotient is displaced by half a mesh point upstream to improve stability.

By making the substitutions for ${\partial x^{(j)}}/{\partial t}$ and ${\partial x^{(j)}}/{\partial r}$ in the set of differential equations (153), one obtains, at each mesh point $r_{k}$, a set of $N_\mathrm{eq}$ non-linear algebraic equations linking the new values to be determined, $\underline{x}_{k-1}$ and $\underline{x}_{k}$, to the “old” ones, $\underline{y}_{k-1}$ and $\underline{y}_{k}$, which are known,

158 $$\begin{aligned} \begin{aligned} {\mathscr {F}}^{(i)}_{k}\left( \underline{x}_{k-1},\underline{x}_{k} | \underline{y}_{k-1},\underline{y}_{k}\right) =0\\ \ i=1\cdots N_\mathrm{eq}, \ k=1\cdots N_\mathrm{r}. \end{aligned} \end{aligned}$$

Note that the structure of the equations is the same at all mesh points, except for $k=1$ and $k=N_\mathrm{r}$. In particular, terms with index $k-1$ do not appear in ${\mathscr {F}}^{(i)}_{1}$. Also, one has to keep in mind that only the $\underline{x}_{k-1}$ and $\underline{x}_{k}$ are unknown; the $\underline{y}_{k-1}$ and $\underline{y}_{k}$ play the role of fixed parameters in these equations (as do the $\varDelta r_{k}$). If one defines a $(N_\mathrm{eq}\times N_\mathrm{r})$-dimension vector ${{\mathscr {X}}^*}$ whose component $N_\mathrm{eq}(k-1)+i$ is $x^{(i)}_{k}$, one can write the system of $N_\mathrm{eq}\times N_\mathrm{r}$ equations as ${{\mathscr {F}}^*}({{\mathscr {X}}^*})=0$, i.e.

159 $$\begin{aligned} {{\mathscr {F}}^*}({{\mathscr {X}}^*}) \equiv \left( \begin{array}{c} {\mathscr {F}}^{(1)}_{1} \\ {\mathscr {F}}^{(2)}_{1} \\ \vdots \\ {\mathscr {F}}^{(N_\mathrm{eq})}_{1} \\ {\mathscr {F}}^{(1)}_{2} \\ \vdots \\ {\mathscr {F}}^{(N_\mathrm{eq})}_{2} \\ \vdots \\ {\mathscr {F}}^{(1)}_{N_\mathrm{r}} \\ \vdots \\ {\mathscr {F}}^{(N_\mathrm{eq})}_{N_\mathrm{r}} \\ \end{array}\right) = \left( \begin{array}{c} 0 \\ \vdots \\ 0 \\ \end{array}\right) , \end{aligned}$$

where I have defined

160 $$\begin{aligned} {{\mathscr {X}}^*} \equiv \left( \begin{array}{c} x^{(1)}_{1} \\ x^{(2)}_{1} \\ \vdots \\ x^{(N_\mathrm{eq})}_{1} \\ x^{(1)}_{2} \\ \vdots \\ x^{(N_\mathrm{eq})}_{2} \\ \vdots \\ x^{(1)}_{N_\mathrm{r}} \\ \vdots \\ x^{(N_\mathrm{eq})}_{N_\mathrm{r}} \\ \end{array}\right) . \end{aligned}$$

The system is solved iteratively using a Newton–Raphson scheme. If ${{\mathscr {X}}^*}_{[ m]}$ is the approximation to the solution of Eq. (159) after iteration *m*, with ${{\mathscr {F}}^*}_{[ m]} \equiv {{\mathscr {F}}^*}({{\mathscr {X}}^*}_{[ m]})\ne 0$, the solution is refined through the relation

161 $$\begin{aligned} {{\mathscr {X}}^*}_{[ m+1]} = {{\mathscr {X}}^*}_{[ m]} - \left( \frac{\partial {{\mathscr {F}}^*}}{\partial {{\mathscr {X}}^*}}\right) ^{-1} {{\mathscr {F}}^*}_{[ m]}, \end{aligned}$$

where $({\partial {{\mathscr {F}}^*}}/{\partial {{\mathscr {X}}^*}})^{-1}$ is the inverse of the matrix of derivatives. The latter, of dimension $(N_\mathrm{eq}\, N_\mathrm{r})\times (N_\mathrm{eq}\, N_\mathrm{r})$, has the following structure

162 $$\begin{aligned} \frac{\partial {{\mathscr {F}}^*}}{\partial {{\mathscr {X}}^*}} = \begin{pmatrix} {\blacksquare }&{} {\square _{+}}\\ {\square _{-}}&{} {\blacksquare }&{} {\square _{+}}\\ &{} {\square _{-}}&{} {\blacksquare }&{} {\square _{+}}\\ &{} &{} \ddots &{} \ddots \\ &{} &{} {\square _{-}}_{k} &{} {\blacksquare }_{k} &{} {\square _{+}}_{k} \\ &{} &{} &{} \ddots &{} \ddots \\ &{} &{} &{} {\square _{-}}&{} {\blacksquare }&{} {\square _{+}}\\ &{} &{} &{} &{} {\square _{-}}&{} {\blacksquare }\\ \end{pmatrix}. \end{aligned}$$

In this diagram, each square is a $N_\mathrm{eq}\times N_\mathrm{eq}$ sub-matrix. For $2\le k \le N_\mathrm{r}-1$, the lines ${N_\mathrm{eq}}k-6$ to ${N_\mathrm{eq}}k$ of ${\partial {{\mathscr {F}}^*}}/{\partial {{\mathscr {X}}^*}}$ are composed of a group of 3 such ${N_\mathrm{eq}}\times {N_\mathrm{eq}}$ matrices, ${\square _{-}}_{k}, {\blacksquare }_{k}, {\square _{+}}_{k}$ that span columns ${N_\mathrm{eq}}k-13$ to ${N_\mathrm{eq}}k+{N_\mathrm{eq}}$, while the rest is composed of zeros,163We can see this more explicitly in the two big matrix expressions of ${\partial {{\mathscr {F}}^*}}/{\partial {{\mathscr {X}}^*}}$,164

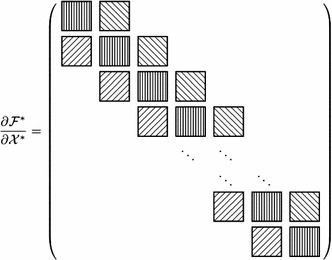




The Heyney method is a way to take advantage of the special structure of the matrix ${\partial {{\mathscr {F}}^*}}/{\partial {{\mathscr {X}}^*}}$ to solve system (161) efficiently, with the number of operations scaling like ${\mathscr {O}}(N_\mathrm{r})$ rather than ${\mathscr {O}}(N_\mathrm{r}^3)$ as would be the case if one uses a general-purpose matrix inversion scheme.^20^ Setting ${{\mathscr {B}}^*}\equiv -{{\mathscr {F}}^*}_{[ m]}$ and ${{\mathscr {W}}^*}\equiv {{\mathscr {X}}^*}_{[ m+1]}-{{\mathscr {X}}^*}_{[ m]}$, Eq. (161) is equivalent to

165 $$\begin{aligned} \left( \frac{\partial {{\mathscr {F}}^*}}{\partial {{\mathscr {X}}^*}}\right) {{\mathscr {W}}^*} = {{\mathscr {B}}^*}, \end{aligned}$$

where ${{\mathscr {W}}^*}$ is the unknown vector. I further decompose vectors ${{\mathscr {W}}^*}$ and ${{\mathscr {B}}^*}$ into $N_\mathrm{eq}$–dimensional sub-vectors, each one representing the values at a given mesh point,

166 $$\begin{aligned} {{\mathscr {W}}^*} = \begin{pmatrix} {\mathscr {W}}_{1} \\ {\mathscr {W}}_{2} \\ \vdots \\ {\mathscr {W}}_{k} \\ \vdots \\ {\mathscr {W}}_{N_\mathrm{r}} \\ \end{pmatrix}. \end{aligned}$$

Then, the system (166) can be written as a set of coupled $N_\mathrm{eq}$–dimensional vector equations,167The algorithm operates in two steps. First, going from $k=1$ to $N_\mathrm{r}$, one defines recursively a sequence of $N_\mathrm{eq}$–vectors ${\mathscr {V}}_{k}$ and $({N_\mathrm{eq}}\times {N_\mathrm{eq}})$–matrices ${\mathfrak M}_{k}$ through

168 $$\begin{aligned} \begin{aligned} {\mathscr {V}}_{1}&= \left( {\blacksquare }_{1}\right) ^{-1} {\mathscr {B}}_{1},\\ {\mathfrak M}_{1}&= \left( {\blacksquare }_{1}\right) ^{-1} {\square _{+}}_{1},\\ {\mathscr {V}}_{k}&= \left( {\blacksquare }_{k}-{\square _{-}}_{k}{\mathfrak M}_{k-1}\right) ^{-1}\left( {\mathscr {B}}_{k}-{\square _{-}}_{k}{\mathscr {V}}_{k-1}\right) ,\\ {\mathfrak M}_{k}&= \left( {\blacksquare }_{k}-{\square _{-}}_{k}{\mathfrak M}_{k-1}\right) ^{-1} {\square _{+}}_{k},~ 2\le k \le N_\mathrm{r}. \end{aligned} \end{aligned}$$

${\mathfrak M}_{N_\mathrm{r}}$ is not defined. In the second step, the values of the unknown ${\mathscr {V}}_{k}$ are computed, climbing back from $k=N_\mathrm{r}$ to 1, with

169 $$\begin{aligned} \begin{aligned} {\mathscr {W}}_{N_\mathrm{r}}&= {\mathscr {V}}_{N_\mathrm{r}},\\ {\mathscr {W}}_{k}&= {\mathscr {V}}_{k} - {\mathfrak M}_{k}{\mathscr {W}}_{k+1},~ 1\le k \le N_\mathrm{r}-1. \end{aligned} \end{aligned}$$

Note that, with this algorithm, only $({N_\mathrm{eq}}\times {N_\mathrm{eq}})$ matrices have to be inverted. I use Gauss elimination for this purpose because this venerable technique proves to be robust enough to properly deal with the kind of badly conditioned matrices that often appear in this application.

The initial model for the Newton–Raphson algorithm is given by the structure of the cluster at the previous time, ${{\mathscr {X}}^*}_{[ 0]}(t)={{\mathscr {X}}^*}(t-\varDelta t)$ One iterates until the following convergence criteria are met. Let us set $\delta x^{(i)}_{k} \equiv \left. x^{(i)}_{k}\right| _{[ m+1]}-\left. x^{(i)}_{k}\right| _{[ m]}$. Then, the condition for logarithmic quantities is

170 $$\begin{aligned} \max _{i=1\ldots N_\mathrm{eq}} \frac{1}{N_\mathrm{r}} \sum _{k=1\ldots N_\mathrm{r}} \left( \delta x^{(i)}_{k}\right) ^2 < \varepsilon _1, \end{aligned}$$

with $\varepsilon _1=10^{-6}$. For velocities (*u*, $v_\mathrm{r}-u$, $v_\mathrm{t}-u$), one checks

171 $$\begin{aligned} \max _{i=1\ldots N_\mathrm{eq}} \frac{1}{N_\mathrm{r}} \sum _{k=1\ldots N_\mathrm{r}} \left( \frac{ \delta x^{(i)}_{k} }{ x^{(i)}_{k}+\varepsilon _1 w_{k} }\right) ^2 < \varepsilon _2, \end{aligned}$$

with $\varepsilon _2=10^{-3}$ and $w_{k}=r_{k}(4\pi G\rho _{k})^{1/2}$. Generally, two iterations are sufficient to reach convergence.

### The local approximation

There are two alternative methods for further simplification of FP or moment models. One is the orbit average, which uses the fact that that any distribution function, being a steady state solution of the collisionless Boltzmann equation, can be expressed as a function of the constants of motion of an individual particle (Jeans’ theorem). For the sake of simplicity, it is assumed that all orbits in the system are regular, as it is the case for example in a spherically symmetric potential; thus the distribution function *f* now only depends maximally on three independent integrals of motion (strong Jeans’ theorem). Let us transform the Fokker–Planck equation to a new set of variables, which comprise the constants of motion instead of the velocities $v_i$. Since in a spherically symmetric system the distribution only depends on energy and the modulus of the angular momentum vector, the number of independent coordinates in this example can be reduced from six to two, and all terms in the transformed equation containing derivatives of other variables than energy and angular momentum vanish (in particular those containing derivatives of the spatial coordinates $x_i$). Integrating the remaining parts of the Fokker–Planck equation over the spatial coordinates is called orbit averaging, because in our present example (a spherical system) it would be an integration over accessible coordinate space for a given energy and angular momentum (which is a spherical shell between $r_{\min }(E,\,J)$ and $r_{\max }(E,\,J)$, the minimum and maximum radius for stars with energy *E* and angular momentum *J*). Such volume integration is, since *f* does not depend anymore on $x_i$ carried over to the diffusion coefficients *D*, which become orbit-averaged diffusion coefficients.

Orbit-averaged Fokker–Planck models effectively deal with the diffusion of orbits according to the changes of their constants of motion, taking into account the potential and the orbital structure of the system in a self-consistent way. However, they are not free of any problems or approximations. They require checks and tests, for example by comparisons with other methods, like the one described in the following. We treat relaxation like the addition of a big non-correlated number of two-body encounters. Close encounters are rare and, thus, I suppose that each encounter produces a very small deflection angle. Thence, relaxation can be regarded as a diffusion process.^21^

A typical two-body encounter in a large stellar system takes place in a volume whose linear dimensions are small compared to other typical radii of the system (total system dimension, or scaling radii of changes in density or velocity dispersion). Consequently, it is assumed that an encounter only changes the velocity, not the position of a particle. Thenceforth, encounters do not produce any changes ${\varDelta \mathbf x}$, so all related terms in the Fokker–Planck equation vanish. However, the local approximation goes even further and assumes that the entire cumulative effect of all encounters on a test particle can approximately be calculated as if the particle were surrounded by a very big homogeneous system with the local distribution function (density, velocity dispersions) everywhere. We are left with a Fokker–Planck equation containing only derivatives with respect to the velocity variables, but still depending on the spatial coordinates (a local Fokker–Planck equation).

In practical astrophysical applications, the diffusion coefficients occurring in the Fokker–Planck equation are not directly calculated, containing the probability $\varPsi$ for a velocity change ${\varDelta \mathbf v}$ from an initial velocity $\mathbf{v}$. Since $D(\varDelta v_i)$, and $D(\varDelta v_i\varDelta v_j)$ have dimensions of velocity (change) per time unit, and squared velocity (change) per time unit, respectively, one calculates such velocity changes in a more direct way, considering a test star moving in a homogeneous sea of field stars. Let the test star have a velocity $\mathbf{v}$ and consider an encounter with a field star of velocity $\mathbf{v}_f$. The result of the encounter (i.e., velocity changes $\varDelta v_i$ of the test star) is completely determined by the impact parameter *p* and the relative velocity at infinity $v_\mathrm{rel} = \vert \mathbf{v} - \mathbf{v}_f\vert$; thus by an integration of the type

172 $$\begin{aligned} \langle \varDelta {\dot{v}}_i\rangle _p = 2\pi \int (\varDelta v_i) \,v_\mathrm{rel}\, n_f\, p\, dp , \end{aligned}$$

the rate of change of the test star velocity due to encounters with $v_\mathrm{rel}$, in the field of stars with particle density $n_f$, averaged over all relevant impact parameters is computed. The integration is normally carried out from $p_0$ (impact parameter for $90^{\circ }$ deflection) until *R*, which is some maximum linear dimension of the system under consideration. Such integration generates in subsequent equations the Coulomb logarithm $\ln \varLambda$; as we have seen previously, it can be well approximated by $\ln (0.11 N)$, where *N* is the total particle number. The diffusion coefficient finally is

173 $$\begin{aligned} D(\varDelta v_i) = \int \langle \varDelta {\dot{v}}_i\rangle _p f(\mathbf{v}_f) d^3\,\mathbf{v}_f, \end{aligned}$$

where $f(\mathbf{v}_f)$ is the velocity distribution of the field stars. In an equal mass system, $f(\mathbf{v}_f)$ should be equal to the distribution function of the test stars occurring in the Fokker–Planck equation for self-consistency. In the case of a multi-mass system, however, $f(\mathbf{v}_f)$ could be different from the test-star distribution, if the diffusion coefficient arising from encounters between two different species of stars is to be calculated. The diffusion coefficients are (for an exact procedure see Binney and Tremaine 2008):

174 $$\begin{aligned} D(\varDelta v_i) =&4\pi G^2 m_f \ln \varLambda \frac{\partial {}}{\partial {v_i}}h(\mathbf{v}), \nonumber \\ D(\varDelta v_iv_j) =&4\pi G^2 m_f \ln \varLambda \frac{{\partial ^2}}{ \partial v_i\partial v_j} g(\mathbf{v}), \end{aligned}$$

where $h(\mathbf{v})$ and $g(\mathbf{v})$ are given by the Rosenbluth potentials (Rosenbluth et al. 1957),

175 $$\begin{aligned} h(\mathbf{v})&= (m+m_f) \int {f(\mathbf{v}_f)\over \vert \mathbf{v}-\mathbf{v}_f\vert } d^3\!\mathbf{v}_f, \nonumber \\ g(\mathbf{v})&= m_f \int f(\mathbf{v}_f) \vert \mathbf{v}-\mathbf{v}_f\vert d^3\!\mathbf{v}_f \ . \end{aligned}$$

With these results we can finally write down the local Fokker–Planck equation in its standard form for the Cartesian coordinate system of the $v_i$:

176 $$\begin{aligned} \left( \frac{\delta f}{\delta t} \right) _\mathrm{enc} = -4\pi G^2 m_f \ln \varLambda \Biggl [ \sum _{i=1}^3 \frac{\partial {}}{\partial {v_i}}\Bigl ( f(\mathbf{v}) \frac{\partial {h}}{\partial {v_i}} \Bigr ) + {1\over 2} \sum _{i,j=1}^3 \frac{{\partial ^2}}{\partial v_i\partial v_j}\Bigl ( f(\mathbf{v}) {\partial ^2 g \over \partial v_i\partial v_j} \Bigr )\Biggr ] \end{aligned}$$

Note that in Rosenbluth et al. (1957), the above equation is given in a covariant notation, which allows for a straightforward transformation into other curvilinear coordinate systems.

Before going ahead the question is raised, why such approximation can be reasonable, regarding the long-range gravitational force, and the impossibility to shield gravitational forces as in the case of Coulomb forces in a plasma by opposite charges. The key is that logarithmic intervals in impact parameter *p* contribute equally to the mean square velocity change of a test particle, provided $p\gg p_0$ (see, e.g., Spitzer Jr 1987, Sect. 2.1). On one side, the lower limit of impact parameters ($p_0$, the $90^o$ deflection angle impact parameter) is small compared to the mean interparticle distance *d* but, on the other side, *D* is a typical radius connected with a change in density or velocity dispersions (e.g., the scale height in a disc of a galaxy), and *R* is the maximum total dimension of the system.

Let us assume $D=100\,d$, and $R=100\,D$. In that case the volume of the spherical shell with radius between *D* and *R* is $10^6$ times larger than the volume of the shell defined by the radii *d* and *D*. Nevertheless the contribution of both shells to diffusion coefficients or the relaxation time is approximately equal. This is a heuristic illustration of why the local approximation is not so bad; the reason is that there are a lot more encounters with particles in the outer, larger shell, but the effect is exactly compensated by the larger deflection angle for encounters happening with particles from the inner shell. If we are in the core or in the plane of a galactic disc the density would fall off further out, so the actual error will be smaller than outlined in the above example. By the same reasoning one can see, however, that the local approximation for a particle in a low-density region, which suffers from relaxation by a nearby density concentration, is prone to failure.

These simple examples should illustrate that under certain conditions the local approximation is a priori not bad. On the other hand, it is obvious from our previous arguments that, if we are interested in relaxation effects on particles in a low-density environment, whose orbit occasionally passes distant, high-density regions, the local approximation could be completely wrong. One might think here, for example, of stars on radially elongated orbits in the halo of globular clusters or of stars, globular clusters, or other objects as massive black holes, on spherical orbits in the galactic halo, passing the galactic disc. In these situations an orbit-averaged treatment seems much more appropriate.

### Monte Carlo codes

The Monte Carlo (MC) numerical scheme is intermediate in realism and numerical efficiency between Fokker–Planck or moment/gas approaches, which are very fast but based on a significantly idealised description of the stellar system, and direct *N*-body codes, which treat (Newtonian) gravity in an essentially assumption-free way but are extremely demanding in terms of computing time. The MC scheme was first introduced by Hénon to follow the relaxational evolution of globular clusters (Hénon 1971a, b, 1973, 1975). To my knowledge, there exist three independent codes in active development and use that are based on Hénon’s ideas. The first is the one written by M. Giersz (see Giersz 2006), which implements many of the developments first introduced by Stodołkiewicz (Stodołkiewicz 1982, 1986). The second code is the one written by K. Joshi (Cluster Monte Carlo, MCM), see Joshi et al. (2000), Joshi et al. (2001) and greatly improved and extended by A. Gürkan and J. Fregeau (see, e.g., Fregeau et al. 2003; Gürkan et al. 2004; Fregeau et al. 2005; Gürkan et al. 2006 and Pattabiraman et al. 2013 describing the latest parallel version). Finally, M. Freitag developed an MC code specifically aimed at the study of galactic nuclei containing a central MBH (Freitag and Benz 2001, 2002; Freitag et al. 2006b).^22^ The description of the method given here is based on this particular implementation.

The MC technique assumes that the cluster is spherically symmetric^23^ and represents it as a set of particles, each of which may be considered as a homogeneous spherical shell of stars sharing the same orbital and stellar properties. The number of particles may be lower than the number of stars in the simulated cluster but the number of stars per particle has to be the same for each particle. Another important assumption is that the system is always in dynamical equilibrium so that orbital time scales need not be resolved and the natural time-step is a fraction of the relaxation (or collision) time. Instead of being determined by integration of its orbit, the position of a particle (i.e., the radius *R* of the shell) is picked up at random, with a probability density for *R* that reflects the time spent at that radius: $\mathrm {d}P/\mathrm {d}R\propto 1/V_\mathrm {r}(R)$ where $V_\mathrm {r}$ is the radial velocity. The Freitag scheme adopts time steps that are a small fraction *f* of the local relaxation (or collision) time: $\delta t(R) \simeq f_{\delta t} \left( t_\mathrm {rlx}^{-1} + t_\mathrm {coll}^{-1}\right) ^{-1}$. Consequently the central parts of the cluster, where evolution is faster, are updated much more frequently than the outer parts. At each step, a pair of neighbouring particles is selected randomly with probability $P_\mathrm {selec} \propto 1/\delta t(R)$. This ensures that a particle stays for an *average* time $\delta t(R)$ at *R* before being updated.

Relaxation is treated as a diffusive process, using the classical Chandrasekhar theory on which FP codes are also based. The long-term effects on orbits of the departure of the gravitational field from a smooth stationary potential are assumed to arise from a large number of uncorrelated, small angle, hyperbolic 2-body encounters. If a star of mass $M_1$ travels with relative velocity $v_\mathrm {rel}$ through a homogeneous field of stars of mass $M_2$ with number density *n* for a time $\delta t$, then in the centre-of-mass reference frame, its trajectory will be deflected by an angle $\theta _{\delta t}$ with average values

177 $$\begin{aligned} \langle \theta _{\delta t} \rangle&= 0, \ \ \text{ and }\nonumber \\ \langle \theta ^2_{\delta t} \rangle&= 8\pi \ln \varLambda \, G^2 n \left( M_1+M_2\right) ^2 \delta t, \end{aligned}$$

where *G* is the gravitational constant and $\ln \varLambda \simeq 10-15$ is the Coulomb logarithm. In the MC code, at each step, the velocities of the particles of the selected pair are modified by a hyperbolic encounter with deflection angle $\theta _\mathrm {eff}=\sqrt{\langle \theta ^2_{\delta t} \rangle }$. The particles are then put at random positions on the slightly modified orbits. As a given particle will be selected many times, at various positions on its orbit, the MC scheme will integrate the effect of relaxation over the particle’s orbit and over all possible field particles. Proper averaging is ensured if the time steps are sufficiently short for the orbit to be modified significantly only after a large number of effective encounters. The energy is trivially conserved to machine accuracy in such a scheme because the same deflection angle $\theta _\mathrm {eff}$ is applied to both particles in an interacting pair. Only the direction of the relative velocity vector is changed by $\theta _\mathrm {eff}$.

Using a binary tree structure which allows quick determination and updating of the potential created by the particles, the self gravity of the stellar cluster is included accurately. This potential is not completely smooth because the particles are infinitesimally thin spherical shells whose radii change discontinuously. Test computations have been used to verify that the additional, unwanted, relaxation is negligible provided the number of particles is larger than a few tens of thousands.

Although Hénon’s method is based on the assumption than all departures from the smooth potential can be treated as 2-body small angle scatterings, it is flexible enough to incorporate more realism. The dynamical effect of binaries (i.e., the dominant 3- and 4-body processes), which may be important in the evolution of globular clusters, have been included in various MC codes through the use of approximate analytical cross-sections (Stodołkiewicz 1986; Giersz and Spurzem 2000; Rasio et al. 2001). Fregeau et al. (2005), Gürkan et al. (2006), and Hypki and Giersz (2013) introduced a much more realistic treatment of binaries by on-the-fly, explicit integrations of the 3- or 4-body interactions, a brute force approach that is necessary to deal with the full diversity of unequal-mass binary interactions. This approach was pioneered by Giersz and Spurzem (2003) in a hybrid code where binaries are followed as MC particles while single stars are treated as a gaseous component. In particular, the code MCM of Joshi et al. (2000), Joshi et al. (2001) has been further developed to integrate larger numbers of particles than earlier attempts with the integrator of Fregeau et al. (2004), named RAPID, see Rodriguez et al. (2015), Fregeau and Rasio (2007), but it is limited to CPUs, and the code does not account for a central MBH in its current status.

The few 2-body encounters that lead to large angle ($> \pi /10$, say) deflections are usually neglected. In globular clusters, these “kicks” have a negligible imprint on the overall dynamics (Hénon 1975; Goodman 1983) but it has been suggested that they lead to a high ejection rate from the density cusp around a central (I)MBH (Lin and Tremaine 1980). Kicks can be introduced in the MC code, where they are treated in a way similar to collisions, with a cross section $\pi b_\mathrm {l.a.}^2$, where $b_\mathrm {l.a.}=f_{\mathrm {l.a.}}G(M_1+M_2)v_\mathrm {rel}^{-2}$. $f_{\mathrm {l.a.}}$ is a numerical factor to distinguish between kicks and “normal” small angle scatterings (impact parameter $> b_\mathrm {l.a.}$). However, simulations seem to indicate that such kicks have little influence on the evolution of a stellar cusp around a MBH (Freitag et al. 2006b).

The MC code is much faster than a direct *N*-body integration: a simulation of a Milky-Way-type galactic nucleus represented by $10^7$ particles requires between a few days and a few weeks of computation on a single CPU. Furthermore, with the proper scaling with the number of stars, the number of stars represented is independent of the number of particles. A high particle number is obviously desirable for robust statistics, particularly when it comes to rare events such as star-MBH interactions. In contrast, because they treat gravitational (Newtonian) interactions on a elementary level, without relying on any theory about their collective and/or long-term effects, the results of direct *N*-body codes can generally be applied only to systems with a number of stars equal to the number of particles used.

### Applications of Monte Carlo and Fokker–Planck simulations to the EMRI problem

MC and FP codes are only appropriate for studying how collisional effects (principally relaxation) affect spherical systems in dynamical equilibrium. These assumptions are probably valid within the radius of influence of MBHs with masses in the LISA range. Indeed, assuming naively that the Sgr A* cluster at the centre of our Galaxy is typical (as far as the total stellar mass and density is concerned) and that one can scale to other galactic nuclei using the $M - \sigma$ relation in the form $\sigma = \sigma _\mathrm{MW} ({\mathscr {M}}_{\bullet }/3.6\times 10^6\,M_{\odot })^{1/\beta }$ with $\beta \approx 4-5$ (Ferrarese and Merritt 2000; Tremaine et al. 2002), one can estimate the relaxation time at the radius of influence to be $t_\mathrm {rlx}(R_\mathrm{infl}) \approx 25\times 10^9\,\mathrm{yr}\,({\mathscr {M}}_{\bullet }/3.6\times 10^6\,M_{\odot })^{(2-3/\beta )}$.

Although observations suggest a large spread amongst the values of the relaxation time at the influence radius of MBHs with similar masses (see, e.g., Fig. 4 of Merritt et al. 2006), most galactic nuclei hosting MBHs less massive than a few $10^6\,M_{\odot }$ are probably relaxed and amenable to MC or FP treatment. Even if the age of the system is significantly smaller than its relaxation time, such approaches are valid as long as the nucleus is in dynamical equilibrium, with a smooth, spherical distribution of matter. In such conditions, relaxational processes are still controlling the EMRI rate, no matter how long the relaxation time is, but one cannot assume a steady-state rate of diffusion of stars onto orbits with small periapsis, as is often done in FP codes (see the discussion in Milosavljević and Merritt (2003), in the different context of the evolution of binary MBHs).

The Hénon-type MC scheme of Freitag and Benz (2002) has been used to determine the structure of galactic nuclei (Freitag and Benz 2002; Freitag et al. 2006b). Predictions for the distribution of stars around a MBH have also been obtained by solving some form of the Fokker–Planck equation (Bahcall and Wolf 1977; Murphy et al. 1991; Hopman and Alexander 2006b, a; Merritt et al. 2006) or using the gaseous model (Amaro-Seoane 2004; Amaro-Seoane et al. 2004). These methods have proved useful to determine how relaxation, collisions, large-angle scatterings, MBH growth, etc., shape the distribution of stars around the MBH, which is an obvious prerequisite for the determination of the rate and characteristics of EMRIs. Of particular importance is the inward segregation of stellar BHs as they lose energy to lighter objects. This effect, combined with the fact that stellar BHs produce GWs with higher amplitude than lower-mass stars, explains why they are expected to dominate the EMRI detection rate (Sigurdsson and Rees 1997; Hopman and Alexander 2006a). An advantage of the MC approach is that it can easily and realistically include a continuous stellar mass spectrum and extra physical ingredients. However, the first point might not be critical here as MC results suggest that, for models where all the stars were born $\sim$ 10 Gyr ago, the pattern of mass segregation can be well approximated by a population of two components only, one representing the stellar BHs and the other representing all other (lighter) objects (Freitag et al. 2006b). Furthermore, the uncertainties are certainly dominated by our lack of knowledge about where and when stellar formation takes place in galactic nuclei, what the masses of the stars which form might be, and what type of compact remnants they become.

The most recent FP results concerning mass segregation were obtained under the assumptions of a fixed potential and an isotropic velocity dispersion, with the effects of (standard or resonant) relaxation being averaged over angular momentum at a given energy. The MC code includes the self-gravity of the cluster so the simulated region can extend past the radius of influence, allowing a more natural outer boundary condition. We note that one has to impose a steeper density drop-off at large radii than what is observed to limit the number of particles to a reasonable value while keeping a good resolution in the region of influence. The MC code naturally allows anisotropy and implicitly follows relaxation in both energy and angular momentum. Anisotropic FP codes for spherical self-gravitating systems exist (Takahashi 1996, 1997; Drukier et al. 1999) but, to our knowledge, none are currently in use that also include a central MBH. Unique amongst all stellar dynamical codes based on the Chandrasekhar theory of relaxation is Fopax, a FP code which assumes axial rather than spherical symmetry, thus permitting the study of clusters and nuclei with significant global rotation (see Fiestas et al. 2006 and references therein) and which has been adapted to include a central MBH (Fiestas 2006).

Determining the EMRI rates and characteristics is a harder challenge for statistical stellar dynamics codes because these events are intrinsically rare and critically sensitive to rather fine details of the stellar dynamics around a MBH. As I explained previously, the main difficulty, in comparison with, for example, tidal disruptions, is that EMRIs are not “one-passage” events but must be gradual. The first estimate of EMRI rates was performed by Hils and Bender (1995). Assuming a static cusp profile, they followed the evolution of the orbits of test-particles subject to GW emission, Eqs. (71) and (72), and 2-body relaxation introduced by random perturbations of the energy and angular momentum according to pre-computed “diffusion coefficients”. Hopman and Alexander (2005) have used a refined version of this “single-particle Monte Carlo method”, as well as the Fokker–Planck equation, to make a more detailed analysis. It was found that no more than $\sim 10\%$ of the compact objects swallowed by the MBH are EMRIs, while the rest are direct plunges.

Determination of EMRI rates and characteristics were also attempted with Freitag’s MC code (Freitag 2001, 2003a, b). Despite its present limitations, this approach might serve to inspire future, more accurate, computations and is therefore worth describing in some detail. The MC code does not include GW emission explicitly (or any other relativistic effects). At the end of each step in which two particles have experienced an encounter (to simulate 2-body relaxation), each particle is tested for entry into the “radiation-dominated” regime, defined by Eq. (65) (with $C_\mathrm{EMRI}=1$). A complication arises because the time step $\delta t$ used in the MC code is a fraction $f_{\delta t}=10^{-3}-10^{-2}$ of the local relaxation time $t_\mathrm {rlx}(R)$, which is generally much larger than the critical timescale defined by the equality $\tau _\mathrm {GW}(e,a) = C_\mathrm{EMRI}\, (1-e)t_\mathrm {rlx}$. In other words, the effective diffusion angle $\theta _\mathrm{eff}$ is generally much larger than the opening angle of the “radiation cone”, $\tilde{\theta }\equiv (1-\tilde{e})^{1/2}$. So that the entry of the particle into the radiation cone (corresponding to a possible EMRI) is not missed, it is assumed that, over $\delta t$, the energy of a given particle does not change. Hence, each time it comes back to a given distance from the centre, its velocity vector has the same modulus but relaxation makes its direction execute a random walk with an individual step per orbital period of $\theta _\mathrm{orb} = \theta _\mathrm{eff} (P_\mathrm{orb}/\delta t)^{1/2}$. Entry into the unstable or radiation cone is tested at each of these sub-steps. If the particle is found on a plunge or radiation-dominated orbit, it is immediately removed from the simulation and its mass is added to the MBH.

Unfortunately, in addition to this approximate way of treating relaxation on small time scales, there are a few reasons why the results of these simulations may be only indicative. One is the way $t_\mathrm {rlx}$ is estimated, using the coefficient in front of $\delta t$ in Eq. (177), i.e., an estimate based on the neighbouring particle. Even if it is correct on average, this estimate is affected by a very high level of statistical noise and its value can be far too long in some cases (e.g., when the relative velocity between the particles in the pair is much larger than the local velocity dispersion). This could lead one to conclude erroneously that a star has reached the radiation-dominated regime and will become an EMRI. To improve on this one could base the $t_\mathrm {rlx}$ estimate on more than one point on the orbit and on more than one “field-particle” (the number of stars within a distance of $10^{-2}$ pc of Sgr A* is probably larger than 1000, so $t_\mathrm {rlx}$ is a well-defined quantity even at such small scales). Another limitation is that GW emission is not included in the orbital evolution, which forces one to assume an abrupt transition when $\tau _\mathrm{GW} = (1-e)t_\mathrm {rlx}$. Hopman and Alexander (2005) have also shown that a value of $C_\mathrm{EMRI}$ as small as $10^{-3}$ might be required to be sure the EMRI will be successful. Furthermore, the MC simulations carried out so far suffer from relatively poor resolution, with each particle having the statistical weight of a few tens of stars. To improve this one would need to limit the simulation to a smaller volume (such as the influence region) or develop a parallel implementation of the MC code to use $\sim 10^8$ particles.

### Direct-summation *N*-body codes

We finally consider the direct *N*-body approach (Aarseth 1999, 2003; Portegies Zwart et al. 2001). This is the most expensive method because it involves integrating all gravitational forces for all particles at every time step, without making any a priori assumptions about the system. The *N*-body codes use the improved Hermite integration scheme as described in Aarseth (1999, 2003), which requires computation of not only the accelerations but also of their time derivatives. Since these approaches integrate Newton’s equations directly, all Newtonian gravitational effects are included naturally. More relevant for this subject is that the family of the direct *N*-body codes of Aarseth also includes versions in which both *KS regularisation* and *chain regularisation* are employed, so that when particles are tightly bound or their separation becomes too small during a hyperbolic encounter, the system is regularised (as described first in Kustaanheimo and Stiefel 1965; Aarseth 2003) to prevent dangerous small individual time steps. This means that we can accurately follow and resolve individual orbits in the system. Other schemes which make use of a softening in the gravitational forces (i.e., $1/(r^2+\epsilon ^2)$ instead of $1/r^2$, where $\epsilon$ is the softening parameter) cannot be employed because $\epsilon$ can induce unacceptable errors in the calculations. The *N*-body codes scale as $N_{\star }^2$, or $\varDelta t \propto t_\mathrm{dyn}$, which means that even with special-purpose hardware, a simulation can take of the order of weeks if not months. This hardware is the GRAPE (short for GRAvity PipE), a family of hardware which acts as a Newtonian force accelerator. For instance, a GRAPE-6A PCI card has a peak performance of 130 Gflop, roughly equivalent to 100 single PCs (Fukushige et al. 2005). It is possible to parallelise basic versions of the direct *N*-body codes (without including regularisation schemes) on clusters of PCs, each equipped with one GRAPE-6A PCI card. This leads to efficiencies greater than 50% and speeds in excess of 2 TFlops and thus the possibility of simulating up to $N_{\star } = 2\cdot 10^6$ stars (Harfst et al. 2006). Nevertheless, when we consider the situation relevant to an EMRI, in which mass ratios are large and we need to follow thousands of orbits, the Hermite integrator is not suitable and problems show up even in the Newtonian regime. Aarseth (2006, 2003) summarise different methods developed to cope with large systems with one or more massive bodies. The problem becomes even more difficult when including relativistic corrections to the forces when the stellar-mass black hole approaches the central MBH, because extremely small time-scales are involved in the integration. Progress is being made in this direction with a developed time-transformed leapfrog method (Mikkola and Aarseth 2002) (for a description of the leapfrog integrator see Mikkola and Merritt 2006) and the even more promising wheel-spoke regularisation, which was developed to handle situations in which a very massive object is surrounded by strongly bound particles, precisely the situation for EMRIs (Zare 1974; Aarseth 2003). Additionally, one must include post-Newtonian corrections in the direct *N*-body code because secular effects such as Kozai or resonant relaxation may be smoothed out significantly by relativistic precession and thus have an impact on the number of captures, see, e.g., Merritt et al. (2011).

#### Relativistic corrections: the post-Newtonian approach

Direct *N*-body have been modified to take into account the role of relativity. The first inclusion of relativistic corrections at 1PN, 2PN (periapsis shifts) and 2.5PN (energy loss in the form of gravitational-wave emission) in an *N*-body code was presented in Kupi et al. (2006). Later, in Brem et al. (2013), we presented the first implementation of the effect of spin in mergers in a direct-summation code, NBODY6. We employ non-spinning post-Newtonian (PN) corrections to the Newtonian accelerations up to 3.5 PN order as well as the spin-orbit coupling up to next-to-lowest order and the lowest order spin-spin coupling.

In Kupi et al. (2006), we included perturbations in the *KS regularisation* scheme, so that the forces (actually the accelerations) were modified by

178 $$\begin{aligned} {F} = \underbrace{{F}_0}_\mathrm{Newtonian} +\underbrace{\underbrace{c^{-2}{F}_2}_{1\mathrm{PN}} + \underbrace{c^{-4}{F}_4}_{2\mathrm{PN}}}_\mathrm{periapsis~shift} + \underbrace{\underbrace{c^{-5}{F}_5}_{2.5\mathrm{PN}}}_\mathrm{GW} + \mathcal {O}(c^{-6}) \end{aligned}$$

These corrections are valid for two isolated bodies and shall thus only be applied to the Newtonian acceleration in the case of strong, relativistic pair-interactions where the perturbation by third bodies is sufficiently small. Because of this, one should restrict the implementation of PN terms to regularised KS pairs. Note that formally the perturbation force in the KS formalism does not need to be small compared to the two-body force, see Mikkola (1997). If the internal KS time step is properly adjusted, the method will work even for relativistic terms becoming comparable to the Newtonian force component. For this reason, I also choose the centre-of-mass frame, which is equivalent to the centre-of-mass Hamiltonian in the ADM (Arnowitt, Deser and Misner) formalism, see Blanchet and Iyer (2003), and not the formulation in the general frame.

These KS pairs are only formed when the interaction between two bodies becomes strong enough so that the pair, as mentioned, has to be regularised. During the KS regularisation the relative motion of the companions is still far from relativistic. Hence, only a small, relativistic subset of all regularised KS pairs will need post-Newtonian corrections.

In the centre-of-mass frame,

179 $$\begin{aligned} \frac{d \varvec{v}}{dt}=-\frac{m}{r^2}\Big [(1+{\mathscr {A}})\,\varvec{n} + {\mathscr {B}}\,\varvec{v} \Big ]+ \varvec{C}_\mathrm{1.5,SO} + \varvec{C}_\mathrm{2,SS} + \varvec{C}_\mathrm{2.5,SO} , \end{aligned}$$

where the relative separation of the binary components is $x^i=y_1^i-y_2^i$, $r=|\mathbf{x}|$ and $n^i={x^i}/{r}$; ${\mathscr {A}}$ and ${\mathscr {B}}$ are given by the expressions (3.10a) and (3.10b) of Blanchet and Iyer (2003). The spin terms $\varvec{C}_\mathrm{N}$, where $\mathrm{N}$ denotes the PN order, are taken from Faye et al. (2006) and Tagoshi et al. (2001). SO stands for spin-orbit and SS for spin-spin coupling.

We can organise the different terms in the following form, using forces per unit mass, $f^i_{g}$, i.e., accelerations:

180 $$\begin{aligned} f^{i}_{g} =&- \frac{GM}{r^{2}}n^{i} + \frac{GM}{r^{2}}\Bigl \{ \left( {\mathscr {A}'}^{}_\mathrm{1PN} + {\mathscr {A}'}^{}_\mathrm{2PN}\right) n^{i} + \frac{\varvec{n v}}{c} \left( {\mathscr {B}'}^{}_\mathrm{1PN} + {\mathscr {B}'}^{}_\mathrm{2PN} \right) \frac{v^{i}}{c} \nonumber \\&+ \frac{\varvec{n v}}{c}{\mathscr {A}'}^{}_\mathrm{2.5PN}\;n^{i} +{\mathscr {B}'}^{}_\mathrm{2.5PN}\frac{v^{i}}{c}\Bigr \} , \end{aligned}$$

where here *M* is the two-body total mass. I list here the PN coefficients for $m_\star \ne 0$ [see, e.g., Blanchet 2006, in particular Eq. (131)]:

181 $$\begin{aligned} {\mathscr {A}'}^{}_\mathrm{1PN} =&\frac{3}{2}\nu \left( \frac{\varvec{nv}}{c}\right) ^{2} -(1+3\nu )\frac{v^{2}}{c^{2}} + \left( 4+2\nu \right) \frac{R^{}_{g}}{r} , \end{aligned}$$

182 $$\begin{aligned} {\mathscr {A}'}^{}_\mathrm{2PN} =&-\frac{15}{8}\nu \left( 1+3\nu \right) \left( \frac{\varvec{nv}}{c}\right) ^{4} +\nu \left( 3-4\nu \right) \left[ \frac{3}{2}\left( \frac{\varvec{n v}}{c}\right) ^{2} -\frac{v^{2}}{c^{2}}\right] \frac{v^{2}}{c^{2}} \nonumber \\ +&\frac{R^{}_{g}}{r} \Bigl \{ 2\left( 1+\frac{25}{2}\nu +\nu ^{2}\right) \left( \frac{\varvec{n v}}{c}\right) ^{2} +\nu \left( \frac{13}{2}-2\nu \right) \frac{v^{2}}{c^{2}}\Bigr \} - \left( 9+\frac{87}{4}\nu \right) \frac{R^{2}_{g}}{r^2} , \end{aligned}$$

183 $$\begin{aligned} {\mathscr {A}'}^{}_\mathrm{2.5PN} =&\frac{24}{5}\frac{R^{}_{g}}{r}\frac{v^2}{c^{2}} + \frac{136}{15}\nu \left( \frac{R^{}_{g}}{r}\right) ^{2} , \end{aligned}$$

184 $$\begin{aligned} {\mathscr {B}'}^{}_\mathrm{1PN} =&4-2\nu , \end{aligned}$$

185 $$\begin{aligned} {\mathscr {B}'}^{}_\mathrm{2PN} =&-\frac{3}{2}\nu \left( 3 +2\nu \right) \left( \frac{\varvec{n v}}{c}\right) ^{2} + \nu \left( \frac{15}{2}+2\nu \right) \frac{v^2}{c^{2}} -\left( 2+\frac{41 \nu }{2} +4 \nu ^2\right) \frac{R^{}_{g}}{r} , \end{aligned}$$

186 $$\begin{aligned} {\mathscr {B}'}^{}_\mathrm{2.5PN} =&-\frac{24}{5}\nu \left( \frac{R^{}_{g}}{r}\right) ^{2} -\frac{8}{5}\nu \frac{R^{}_{g}}{r}\frac{v^{2}}{c^{2}} , \end{aligned}$$

where $\nu$ is the symmetric mass ratio, $\nu = m_\star M_\bullet /M^2$, with $m_\star$ the mass of the stellar-mass black hole, and $R^{}_{g} = GM/c^{2}$. One can verify that the coefficients in Eqs. (228) to (231) agree with Eqs. (181) to (186) for $\nu = 0$.

Whilst the gauge choice was not a problem for the system studied in Kupi et al. (2006), since we were interested in the global dynamical evolution, for the EMRI problem the centre-of-mass frame (located at the origin of the coordinates) must be employed. The integration cannot be extended to velocities higher than $\sim$ 0.3 c, because at these velocities the post-Newtonian formalism can no longer be applied accurately. This means that we cannot reach the final coalescence of the stellar BH with the MBH, but this is not a big issue, because this part of the evolution does not contribute significantly to the SNR of the GW signal. We note that it will not be possible to include in *N*-body codes all the $\mathrm{PN}$ corrections that are required for accurate modelling of the phase evolution of the EMRI during the last few years before plunge. However, the *N*-body codes are not required in that regime, since the system is then decoupled from the rest of the stellar cluster.

The expressions for the accelerations are:

187 $$\begin{aligned} \varvec{a}_2 =&\frac{Gm_2}{r^2} \Biggl \{ \varvec{n}\left[ -v_1^2-2v_2^2+4\varvec{v_1 v_2}+\frac{3}{2}(\varvec{n v_2})^2+ 5\left( \frac{Gm_1}{r}\right) +4\left( \frac{Gm_2}{r}\right) \right] \nonumber \\&+(\varvec{v}_1-\varvec{v}_2) \left[ 4\varvec{n v_1}-3\varvec{n v_2}\right] \Biggr \} \end{aligned}$$

188 $$\begin{aligned} \varvec{a}_4 =&\frac{Gm_2}{r^2} \Biggl \{ \varvec{n} \Biggl [-2v_2^4+4v_2^2(\varvec{v_1 v_2})-2(\varvec{v_1 v_2})^2 + \frac{3}{2}v_1^2(\varvec{n v_2})^2+ \frac{9}{2}v_2^2(\varvec{n v_2})^2\nonumber \\&-6(\varvec{v_1 v_2})(\varvec{n v_2})^2 - \frac{15}{8}(\varvec{n v_2})^4+ \left( \frac{Gm_1}{r} \right) \nonumber \\&\times \Bigl (-\frac{15}{4}v_1^2+ \frac{5}{4}v_2^2- \frac{5}{2}\varvec{v_1 v_2} +\frac{39}{2}(\varvec{n v_1})^2-39(\varvec{n v_1})(\varvec{n v_2})+\frac{17}{2}(\varvec{n v_2})^2 \Bigr ) \nonumber \\&+\left( \frac{Gm_2}{r}\right) (4v_2^2-8\varvec{v_1 v_2}+2(\varvec{n v_1})^2 -4(\varvec{n v_1})(\varvec{n v_2})-6(\varvec{n v_2})^2) \Biggr ] \nonumber \\&+ (\varvec{v}_1-\varvec{v}_2) \Biggl [ v^2_1(\varvec{n v_2})+ 4v_2^2(\varvec{n v_1})-5v_2^2(\varvec{n v_2}) -4(\varvec{v_1 v_2})(\varvec{n v_1})+ 4(\varvec{v_1 v_2})(\varvec{n v_2}) \nonumber \\&-6(\varvec{n v_1})(\varvec{n v_2})^2 +\frac{9}{2}(\varvec{n v_2})^3+\left( \frac{Gm_1}{r} \right) \left( -\frac{63}{4}\varvec{n v_1}+\frac{55}{4}\varvec{n v_2} \right) \nonumber \\&+\left( \frac{Gm_2}{r} \right) \left( -2\varvec{n v_1}-2\varvec{n v_2} \right) \Biggr ] \Biggr \}+ \frac{G^3m_2}{r^4}\varvec{n} \left[ -\frac{57}{4}m_1^2-9m_2^2-\frac{69}{2}m_1m_2\right] , \end{aligned}$$

189 $$\begin{aligned} \varvec{a}_5 =&\frac{4}{5}\frac{G^2m_1m_2}{r^3}\Biggl \{\left( \varvec{v}_1- \varvec{v}_2\right) \left[ -\left( \varvec{v}_1- \varvec{v}_2\right) ^2+2\left( \frac{Gm_1}{r}\right) -8\left( \frac{Gm_2}{r}\right) \right] \nonumber \\&+\varvec{n}(\varvec{n v_1}-\varvec{n v_2})\left[ 3(\varvec{v}_1-\varvec{v}_2)^2- 6\left( \frac{Gm_1}{r} \right) + \frac{52}{3}\left( \frac{Gm_2}{r}\right) \right] \Biggr \}. \end{aligned}$$

The basis of direct Nbody4 and Nbody6++ codes relies on an improved Hermit integrator scheme by Makino and Aarseth (1992) and Aarseth (1999), for which we need not only the accelerations but also their time derivative, given by

190 $$\begin{aligned} {\dot{\varvec{a}}}_0=&-Gm_2\left( \frac{\varvec{v}_1-\varvec{v}_2}{r^3}+3\frac{\varvec{n}}{r^3}(\varvec{n v_1}-\varvec{n v_2})\right) \end{aligned}$$

191 $$\begin{aligned} \dot{\varvec{a}}_2 =&Gm_2 {\{} -\Biggl [(\varvec{v}_1-\varvec{v}_2)\frac{v_1^2}{r^3}+ 2\varvec{n}\frac{v_1a_1}{r^2}+ 3\varvec{n}\frac{v_1^2(\varvec{n v_2}-\varvec{n v_1})}{r^3}\Biggr ]\nonumber \\&-2\Biggl [(\varvec{v}_1-\varvec{v}_2)\frac{v_2^2}{r^3}+2\varvec{n}\frac{\varvec{v_2 a_2}}{r^2}+ 3\varvec{n}\frac{v_2^2(\varvec{n v_2}-\varvec{n v_1})}{r^3}\Biggr ] \nonumber \\&+ 4\Big [(\varvec{v}_1-\varvec{v}_2)\frac{\varvec{v_1 v_2}}{r^3}+\varvec{n}\frac{\varvec{a_1 v_2}+\varvec{a_2 v_1}}{r^2}+ 3\varvec{n}\frac{\varvec{v_1 v_2}(\varvec{n v_2}-\varvec{n v_1})}{r^3}\Big ]\nonumber \\&+\frac{3}{2}\Biggl [(\varvec{v}_1-\varvec{v}_2)\frac{(\varvec{n v_2})^2}{r^3}+ 2\varvec{n}(\varvec{n v_2})\frac{r(\varvec{n a_2})+\varvec{v_1 v_2}-v_2^2}{r^3}\nonumber \\&+ 5\varvec{n}\frac{(\varvec{n v_2})^2(\varvec{n v_2}-\varvec{n v_1})}{r^3}\Biggr ] \nonumber \\&+ G\Biggl [\frac{\varvec{v}_1-\varvec{v}_2}{r^4}+4\varvec{n}\frac{\varvec{n v_2}-\varvec{n v_1}}{r^4}\Biggr ] (5m_1+4m_2)+4\frac{\varvec{n v_1}}{r^2}(\varvec{a}_1-\varvec{a}_2)\nonumber \\&+ 3\frac{\varvec{n v_2}}{r^2}(\varvec{a}_2-\varvec{a}_1) \nonumber \\&+ 4\frac{v_1^2-\varvec{v_1 v_2}+r(\varvec{n a_1})+3(\varvec{n v_2}-\varvec{n v_1})\varvec{n v_1}}{r^3}(\varvec{v}_1-\varvec{v}_2) \nonumber \\&+ 3\frac{\varvec{v_1 v_2}-v_2^2+r(\varvec{n a_2})+3(\varvec{n v_2}-\varvec{n v_1})\varvec{n v_2}}{r^3}(\varvec{v}_2-\varvec{v}_1) \end{aligned}$$

$$\begin{aligned} \dot{\varvec{a}}_4 =&Gm_2 \Biggl \{ -2\left[ (\varvec{v}_1-\varvec{v}_2)\frac{v_2^4}{r^3}+ \varvec{n}\frac{4v_2^2(a_2v_2)}{r^2}+3\varvec{n}\frac{v_2^4(\varvec{n v_2}-\varvec{n v_1})}{r^3}\right] \\&+ 4\left[ (\varvec{v}_1-\varvec{v}_2)\frac{v_2^2\varvec{v_1 v_2}}{r^3}+ 2\varvec{n}\frac{(\varvec{v_2 a_2})(\varvec{v_1 v_2})}{r^2}+\varvec{n}\frac{v_2^2(\varvec{a_1 v_2}+v_1a_2)}{r^2}\right. \\&\left. + 3\varvec{n}\frac{v_2^2(\varvec{v_1 v_2})(\varvec{n v_2}-\varvec{n v_1})}{r^3}\right] \\&- 2\left[ (\varvec{v}_1-\varvec{v}_2)\frac{(v_2v_1)^2}{r^3}+ 2\varvec{n}\frac{(\varvec{v_1 v_2})(\varvec{a_1 v_2}+\varvec{a_2 v_1})}{r^2}+ 3\varvec{n}\frac{(\varvec{v_1 v_2})^2(\varvec{n v_2}-\varvec{n v_1})}{r^3}\right] \\&+ \frac{3}{2}\Biggl [(\varvec{v}_1-\varvec{v}_2)\frac{v_1^2(\varvec{n v_2})^2}{r^3}+ 2\varvec{n}\frac{v_1a_1(\varvec{n v_2})^2}{r^2}+2\varvec{n}\frac{v_1^2(\varvec{n v_2})}{r^2} \left( \varvec{n a_2}+\frac{\varvec{v_1 v_2}-v_2^2}{r}\right) \\&+5\varvec{n} \frac{v_1^2(\varvec{n v_2})^2}{r^3}(\varvec{n v_2}-\varvec{n v_1}) \Biggr ] \end{aligned}$$

$$\begin{aligned}&+ \frac{9}{2} \left[ (\varvec{v}_1-\varvec{v}_2)\frac{v_2^2(\varvec{n v_2})^2}{r^3}+ 2\varvec{n}\frac{\varvec{v_2 a_2}(\varvec{n v_2})^2}{r^2}+2\varvec{n}\frac{v_2^2(\varvec{n v_2})}{r^2} \left( \varvec{n a_2}+\frac{\varvec{v_1 v_2}-v_2^2}{r}\right) \right. \\&\left. + 5\varvec{n}\frac{v_2^2(\varvec{n v_2})^2}{r^3}(\varvec{n v_2}-\varvec{n v_1})\right] \\&- 6 \Biggl [ (\varvec{v}_1-\varvec{v}_2)\frac{\varvec{v_1 v_2}(\varvec{n v_2})^2}{r^3}+ \varvec{n} \frac{(\varvec{a_1 v_2}+v_1a_2)(\varvec{n v_2})^2}{r^2} \\&+ 2\varvec{n}\frac{\varvec{v_1 v_2}(\varvec{n v_2})}{r^2} \left( \varvec{n a_2}+\frac{\varvec{v_1 v_2}-v_2^2}{r}\right) + 5\varvec{n}\frac{\varvec{v_1 v_2}(\varvec{n v_2})^2(\varvec{n v_2}-\varvec{n v_1})}{r^3} \Biggr ] \\&- \frac{15}{8}\left[ (\varvec{v}_1-\varvec{v}_2)\frac{(\varvec{n v_2})^4}{r^3}+ 4\varvec{n}\frac{(\varvec{n v_2})^3}{r^2}\left( \varvec{n a_2}+\frac{\varvec{v_1 v_2}-v_2^2}{r}\right) \right. \\&\left. + 7\varvec{n}\frac{(\varvec{n v_2})^4}{r^3}(\varvec{n v_2}-\varvec{n v_1})\right] \\&+ Gm_1 \Bigg \langle -\frac{15}{4}\left[ (\varvec{v}_1-\varvec{v}_2)\frac{v_1^2}{r^4}+ 2\varvec{n}\frac{v_1a_1}{r^3}+ 4\varvec{n}\frac{v_1^2(\varvec{n v_2}-\varvec{n v_1})}{r^4}\right] \\&+ \frac{5}{4}\left[ (\varvec{v}_1-\varvec{v}_2)\frac{v_2^2}{r^4}+ 2\varvec{n}\frac{\varvec{v_2 a_2}}{r^3}+ 4\varvec{n} \frac{v_2^2(\varvec{n v_2}-\varvec{n v_1})}{r^4}\right] \\&- \frac{5}{2}\left[ (\varvec{v}_1-\varvec{v}_2)\frac{\varvec{v_1 v_2}}{r^4}+ \varvec{n}\frac{\varvec{a_1 v_2}+v_1a_2}{r^3}+4\varvec{n} \frac{\varvec{v_1 v_2}(\varvec{n v_2}-\varvec{n v_1})}{r^4}\right] \\&+ \frac{39}{2}\left[ (\varvec{v}_1-\varvec{v}_2)\frac{(\varvec{n v_1})^2}{r^4}+ 2\varvec{n}\frac{\varvec{n v_1}}{r^3}\left( \varvec{n a_1}+\frac{v_1^2-\varvec{v_1 v_2}}{r}\right) \right. \\&+\left. 6\varvec{n}\frac{(\varvec{n v_1})^2(\varvec{n v_2}-\varvec{n v_1})}{r^4}\right] \\&- 39 \Biggl [(\varvec{v}_1-\varvec{v}_2)\frac{(\varvec{n v_1})(\varvec{n v_2})}{r^4}\\&+ \frac{\varvec{n}}{r^3}\left( (\varvec{n v_1})(\varvec{n a_2})+(\varvec{n v_2})(\varvec{n a_1})+ \frac{\varvec{n v_1}(\varvec{v_1 v_2}-v_2^2)}{r}+\frac{\varvec{n v_2}(v_1^2-\varvec{v_1 v_2})}{r}\right) \\&+ 6\varvec{n}\frac{(\varvec{n v_1})(\varvec{n v_2})}{r^4}(\varvec{n v_2}-\varvec{n v_1})\Biggr ]\\&+\frac{17}{2}\left[ (\varvec{v}_1- \varvec{v}_2)\frac{(\varvec{n v_2})^2}{r^4}+2\varvec{n}\frac{\varvec{n v_2}}{r^3} \left( \varvec{n a_2}+\frac{\varvec{v_1 v_2}-v_2^2}{r}\right) \right. \\&\left. +6\varvec{n} \frac{(\varvec{n v_2})^2(\varvec{n v_2}-\varvec{n v_1})}{r^4}\right] \Bigg \rangle \\&+ Gm_2 \Bigg \langle 4\left[ (\varvec{v}_1-\varvec{v}_2)\frac{v_2^2}{r^4}+2\varvec{n} \frac{\varvec{v_2 a_2}}{r^3}+4\varvec{n}\frac{v_2^2(\varvec{n v_2}-\varvec{n v_1})}{r^4}\right] \\&- 8\left[ (\varvec{v}_1-\varvec{v}_2)\frac{\varvec{v_1 v_2}}{r^4}+\varvec{n} \frac{\varvec{a_1 v_2}+v_1a_2}{r^3}+4\varvec{n}\frac{\varvec{v_1 v_2}(\varvec{n v_2}-\varvec{n v_1})}{r^4}\right] \end{aligned}$$

$$\begin{aligned}&+ 2\left[ (\varvec{v}_1-\varvec{v}_2)\frac{(\varvec{n v_1})^2}{r^4}+2\varvec{n} \frac{\varvec{n v_1}}{r^3}\left\{ \varvec{n a_1}+\frac{v_1^2-\varvec{v_1 v_2}}{r}\right\} \right. \\&\left. + 6\varvec{n}\frac{(\varvec{n v_1})^2(\varvec{n v_2}-\varvec{n v_1})}{r^4}\right] \\&- 4 \Biggl [ (\varvec{v}_1-\varvec{v}_2)\frac{(\varvec{n v_1})(\varvec{n v_2})}{r^4}\\&+ \frac{\varvec{n}}{r^3}\left\{ (\varvec{n v_1})(\varvec{n a_2})+(\varvec{n v_2})(\varvec{n a_1})+ \frac{\varvec{n v_1}(\varvec{v_1 v_2}-v_2^2)}{r}+\frac{\varvec{n v_2}(v_1^2-\varvec{v_1 v_2})}{r}\right\} \\&+ 6\varvec{n}\frac{(\varvec{n v_1})(\varvec{n v_2})}{r^4}(\varvec{n v_2}-\varvec{n v_1}) \Biggr ] \\&- 6 \left[ (\varvec{v}_1-\varvec{v}_2)\frac{(\varvec{n v_2})^2}{r^4}+2\varvec{n} \frac{\varvec{n v_2}}{r^3}\left\{ \varvec{n a_2}+\frac{\varvec{v_1 v_2}-v_2^2}{r}\right\} \right. \\&\left. + 6\varvec{n}\frac{(\varvec{n v_2})^2(\varvec{n v_2}-\varvec{n v_1})}{r^4}\right] { \Bigg \rangle } \\&+ (\varvec{a}_1-\varvec{a}_2)\frac{v_1^2(\varvec{n v_2})}{r^2}+(\varvec{v}_1-\varvec{v}_2)\\&\times \left\{ \frac{2(v_1a_1)(\varvec{n v_2})}{r^2}+\frac{v_1^2}{r^2} \left( \varvec{n a_2}+\frac{\varvec{v_1 v_2}-v_2^2}{r}+3\frac{\varvec{n v_2}}{r}(\varvec{n v_2}-\varvec{n v_1})\right) \right\} \\&+ 4(\varvec{a}_1-\varvec{a}_2)\frac{v_2^2(\varvec{n v_1})}{r^2}\\&+4(\varvec{v}_1-\varvec{v}_2) \left\{ \frac{2(\varvec{v_2 a_2})(\varvec{n v_1})}{r^2}+\frac{v_2^2}{r^2} \left( \varvec{n a_1}+\frac{v_1^2-\varvec{v_1 v_2}}{r}+3\frac{\varvec{n v_1}}{r}(\varvec{n v_2}-\varvec{n v_1})\right) \right\} \\&- 5(\varvec{a}_1-\varvec{a}_2)\frac{v_2^2(\varvec{n v_2})}{r^2}\\&-5(\varvec{v}_1-\varvec{v}_2) \left\{ \frac{2(\varvec{v_2 a_2})(\varvec{n v_2})}{r^2}+\frac{v_2^2}{r^2} \left( \varvec{n a_2}+\frac{\varvec{v_1 v_2}-v_2^2}{r}+3\frac{\varvec{n v_2}}{r}(\varvec{n v_2}-\varvec{n v_1})\right) \right\} \\&- 4(\varvec{a}_1-\varvec{a}_2)\frac{(\varvec{v_1 v_2})(\varvec{n v_1})}{r^2}-4(\varvec{v}_1-\varvec{v}_2) \Biggl \{ \frac{(\varvec{a_1 v_2}+v_1a_2)(\varvec{n v_1})}{r^2} \\&+ \frac{\varvec{v_1 v_2}}{r^2} \left( \varvec{n a_1}+\frac{v_1^2-\varvec{v_1 v_2}}{r}+3\frac{(\varvec{v_1 v_2})(\varvec{n v_1})}{r}(\varvec{n v_2}-\varvec{n v_1}) \right) \Biggr \} \\&+ 4(\varvec{a}_1-\varvec{a}_2)\frac{(\varvec{v_1 v_2})(\varvec{n v_2})}{r^2}+4(\varvec{v}_1-\varvec{v}_2) \Biggl \{\frac{(\varvec{a_1 v_2}+v_1a_2)(\varvec{n v_2})}{r^2} \\&+ \frac{\varvec{v_1 v_2}}{r^2} \left( \varvec{n a_2}+\frac{\varvec{v_1 v_2}-v_2^2}{r}+ 3\frac{(\varvec{v_1 v_2})(\varvec{n v_2})}{r}(\varvec{n v_2}-\varvec{n v_1}) \right) \Biggr \} \\&-6(\varvec{a}_1 -\varvec{a}_2)\frac{(\varvec{n v_1})(\varvec{n v_2})^2}{r^2} \\&- 6(\varvec{v}_1-\varvec{v}_2)\Biggl \{\frac{(\varvec{n v_2})^2}{r^2} \left( \varvec{n a_1}+\frac{v_1^2-\varvec{v_1 v_2}}{r}\right) \end{aligned}$$

192 $$\begin{aligned}&+\frac{2(\varvec{n v_1})(\varvec{n v_2})}{r^2} \left( \varvec{n a_2}+\frac{\varvec{v_1 v_2}-v_2^2}{r}\right) + 5\frac{(\varvec{n v_1})(\varvec{n v_2})^2}{r^3}(\varvec{n v_2}-\varvec{n v_1})\Biggr \}\ \nonumber \\&+ \frac{9}{2}(\varvec{a}_1-\varvec{a}_2)\frac{(\varvec{n v_2})^3}{r^2}\nonumber \\&+ \frac{9}{2}(\varvec{v}_1-\varvec{v}_2)\left\{ \frac{3(\varvec{n v_2})^2}{r^2} \left( \varvec{n a_2}+\frac{\varvec{v_1 v_2}-v_2^2}{r}\right) + 5\frac{(\varvec{n v_2})^3}{r^3}(\varvec{n v_2}-\varvec{n v_1})\right\} \nonumber \\&+ G \Biggl \langle \left[ (\varvec{a}_1-\varvec{a}_2)\frac{\varvec{n v_1}}{r^3}+ (\varvec{v}_1-\varvec{v}_2)\left\{ \frac{\varvec{n a_1}}{r^3}+ \frac{v_1^2-\varvec{v_1 v_2}}{r^4}+4\frac{\varvec{n v_1}}{r^4}(\varvec{n v_2}-\varvec{n v_1})\right\} \right] \nonumber \\&\times \left[ -\frac{63}{4}m_1-2m_2\right] \nonumber \\&+ \left[ (\varvec{a}_1-\varvec{a}_2)\frac{\varvec{n v_2}}{r^3}+(\varvec{v}_1-\varvec{v}_2) \left\{ \frac{\varvec{n a_2}}{r^3}+\frac{\varvec{v_1 v_2}-v_2^2}{r^4}+4\frac{\varvec{n v_2}}{r^4}(\varvec{n v_2}-\varvec{n v_1}) \right\} \right] \nonumber \\&\times \left[ \frac{55}{4}m_1-2m_2\right] \Biggr \rangle \Biggr \} \nonumber \\&+ G^3 m_2\left( -\frac{57}{4}m_1^2-9m_2^2-\frac{69}{2}m_1m_2\right) \left( \frac{\varvec{v}_1-\varvec{v}_2}{r^5}+5\varvec{n}\frac{\varvec{n v_2}-\varvec{n v_1}}{r^5}\right) \end{aligned}$$

193 $$\begin{aligned} \dot{\varvec{a}}_5 =&\frac{4}{5}G^2m_1m_2\Biggl \{-\frac{(\varvec{a}_1-\varvec{a}_2) (\varvec{v}_1-\varvec{v}_2)^2}{r^3}-2\frac{\varvec{v}_1- \varvec{v}_2}{r^3}(v_1a_1+\varvec{v_2 a_2}-v_2a_1-v_1a_2) \nonumber \\&+6\frac{\varvec{v}_1-\varvec{v}_2}{r^4}(\varvec{v}_1-\varvec{v}_2)^2(\varvec{n v_1}-\varvec{n v_2})+ G(2m_1-8m_2)\nonumber \\&\times \left[ \frac{\varvec{a}_1-\varvec{a}_2}{r^4}+ 4\frac{\varvec{v}_1-\varvec{v}_2}{r^5}(\varvec{n v_2}-\varvec{n v_1})\right] \nonumber \\&+ 3 {\Biggl [}\varvec{n}\frac{(\varvec{v}_1-\varvec{v}_2)^2}{r^3} \left( \varvec{n a_1}-\varvec{n a_2}+\frac{v_1^2+v_2^2-2\varvec{v_1 v_2}}{r}\right) \nonumber \\&+2\varvec{n} \frac{\varvec{n v_1}-\varvec{n v_2}}{r^3}(v_1a_1+\varvec{v_2 a_2}-v_2a_1-v_1a_2) \nonumber \\&- 5\varvec{n}\frac{(\varvec{v}_1-\varvec{v}_2)^2(\varvec{n v_1}-\varvec{n v_2})^2}{r^4} {\Biggr ]} + G\left( \frac{52}{3}m_2-6m_1\right) {\Biggl [}(\varvec{v}_1-\varvec{v}_2) \frac{\varvec{n v_1}-\varvec{n v_2}}{r^5} \nonumber \\&+ \frac{\varvec{n}}{r^4}\left( \varvec{n a_1}-\varvec{n a_2}+ \frac{v_1^2-2\varvec{v_1 v_2}+v_2^2}{r}\right) - 6\varvec{n}\frac{(\varvec{n v_2}-\varvec{n v_1})^2}{r^5} {\Biggr ]} \Biggr \} \end{aligned}$$

In the last expressions, I have used different kinds of brackets, including angle brackets for legibility reasons. Gábor Kupi and I derived in 2005 independently the equations for our joint work of Kupi et al. (2006) and compared the results with the output of a computer algebra system by defining a function that was the substraction of our derivations and the numerical output. The result turned out to be smaller than $10^{-18}$. On the other hand, the terms have been now tested by a number of independent works that adapted them in their codes. Also, in Brem et al. (2013) we did a series of tests that compared the terms with the semi-Keplerian approximation of Peters (1964).

In addition to the effects on the acceleration, the spin of compact objects of Eq. (179) undergoes precession in relativistic two-body interactions. This is also taken into account by integrating the spin precession equations

194 $$\begin{aligned} \frac{d \varvec{S}}{d t}&= \frac{1}{c^2} \varvec{U}_\mathrm{1,SO} + \frac{1}{c^3} \varvec{U}_\mathrm{1.5,SS} + \frac{1}{c^4} \varvec{U}_\mathrm{2,SO}, \end{aligned}$$

195 $$\begin{aligned} \frac{d \varvec{\varSigma }}{d t}&= \frac{1}{c^2} \varvec{V}_\mathrm{1,SO} + \frac{1}{c^3} \varvec{V}_\mathrm{1.5,SS} + \frac{1}{c^4} \varvec{V}_\mathrm{2,SO}, \end{aligned}$$

196 $$\begin{aligned} \varvec{S}&= \varvec{S}_1 + \varvec{S}_2, \end{aligned}$$

197 $$\begin{aligned} \varvec{\varSigma }&= m\left( \frac{\varvec{S}_2}{m_2}-\frac{\varvec{S}_1}{m_1}\right) . \end{aligned}$$

$\varvec{S}$ and $\varvec{\varSigma }$ describe the spin state of the pair. The individual terms for $\varvec{U}_N$ and $\varvec{V}_N$, where *N* denotes the PN order, can be found in Faye et al. (2006) and Buonanno et al. (2003).

**Fig. 65:**
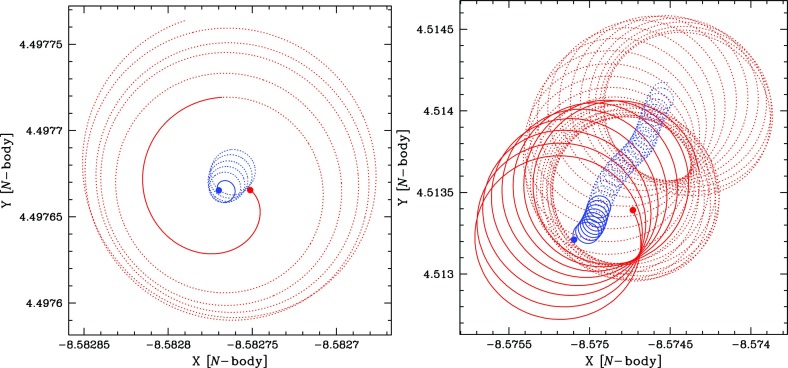
Projection in the x–y plane of two moments in the orbital evolution of a binary. The length units are given in *N*-body units For this example, the binary has a mass ratio of 10, and is integrated with *N*-body6 including the post-Newtonian treatment described in Kupi et al. (2006). The expressions corresponding to the relativistic corrections for the accelerations and their time derivatives are given in Eqs. (187)–(193). For this particular simulation, I did an *N*-body unit of length corresponds to 0.21 pc (see Sect. 8.8.3)

Figure 65 shows the evolution of a binary that formed in an *N*-body simulation with the code developed in Kupi et al. (2006). The binary corresponded to two stellar-mass black holes with a mass ratio of 10, and I am only using the PN terms for perihelion shift and gravitational-radiation loss.

#### Relativistic corrections: a geodesic solver

Brem et al. (2014) presented, for the first time, a geodesic approximation for the relativistic orbits in an *N*-body code. I show in this section, the geodesic equations of motion in a form that is suitable to be included in an *N*-body code that uses a Newtonian-type formulation of the equations of motion (initially presented in the appendix of Brem et al. 2014). Also, so as to be able to compare results with post-Newtonian approach, I show the geodesic equations using harmonic coordinates for Schwarzschild, which are compatible with the harmonic gauge condition of post-Newtonian theory.

Since we are integrating stars, we need to consider the geodesics for massive particles (i.e., timelike geodesics). Given our system of spacetime coordinates $\{x^\mu \} = \{t,x^i\}$ ($\mu ,\nu , \ldots = 0-3;\; i , j , \ldots = 1-3$), a geodesic will be given by $\{x^\mu (\tau )\}$, where $\tau$ denotes the particle’s proper time. The components of the velocity vector are defined as

198 $$\begin{aligned} u^\mu = \frac{dx^\mu (\tau )}{d\tau } . \end{aligned}$$

This four-velocity vector satisfies:

199 $$\begin{aligned} g^{}_{\mu \nu }u^{\mu }u^{\nu } = - c^{2} , \end{aligned}$$

where $g^{}_{\mu \nu }$ is the Schwarzschild metric in our coordinate system and *c* denotes the speed of light. Since we are interested in geodesics, the velocity vector must satisfy the following equation of motion, see e.g., the book by Misner et al. (1973).

200 $$\begin{aligned} u^\nu \nabla ^{}_\nu u^\mu = 0 , \end{aligned}$$

where $\nabla ^{}_\mu$ denotes the canonical covariant derivative associated with the spacetime metric $g_{\mu \nu }$. Expanding this equation we have

201 $$\begin{aligned} \frac{du^\rho }{d\tau } + \varGamma ^{\rho }_{\mu \nu }u^\mu u^\nu = 0 , \end{aligned}$$

being $\varGamma ^\rho _{\mu \nu }$ the Christoffel symbols associated with the spacetime metric $g^{}_{\mu \nu }$. They are given in terms of the metric by:

202 $$\begin{aligned} \varGamma ^{\mu }_{\alpha \beta } = \frac{1}{2} g^{\mu \nu }\left( \frac{\partial g^{}_{\alpha \nu }}{\partial x^{\beta }} + \frac{\partial g^{}_{\beta \nu }}{\partial x^{\alpha }} - \frac{\partial g^{}_{\alpha \beta }}{\partial x^{\rho }} \right) . \end{aligned}$$

Using the splitting of time and space we can write the velocity vector as follows:

203 $$\begin{aligned} \mathbf {\varvec{u}} = u^t \frac{\partial }{\partial t} + u^i\frac{\partial }{\partial x^i} , \end{aligned}$$

where $\{u^t,u^i\}$ are the velocity components in the $\{t,x^i\}$ coordinate system:

204 $$\begin{aligned} u^t = \frac{\partial t(\tau )}{\partial \tau } ,~~~~~ u^i = \frac{\partial x^i(\tau )}{\partial \tau } . \end{aligned}$$

Therefore, on the trajectory of the particle we can write

205 $$\begin{aligned} u^i = \frac{dx^i(t)}{dt}\frac{\partial t}{\partial \tau } = v^i u^t \equiv \varGamma v^{i} , \end{aligned}$$

where $v^i$ are the spatial components of the velocity

206 $$\begin{aligned} v^i = \frac{dx^i(t)}{dt} , \end{aligned}$$

and $\varGamma$ is the general relativistic version of the special relativistic gamma factor, which is given in terms of the components of the spatial velocity and the metric tensor as:

207 $$\begin{aligned} \varGamma ^{2} = -\frac{c^{2}}{g^{}_{tt} + 2g^{}_{ti}v^i + g^{}_{ij}v^iv^j} . \end{aligned}$$

which, in the weak-field limit ($g^{}_{tt}\approx -c^{2}\,$, $g^{}_{ti}\approx 0\,$, $g^{}_{ij}\approx \delta ^{}_{ij}\,$), has the usual expression:

208 $$\begin{aligned} \varGamma ^{2} \approx \frac{1}{1-\frac{v^{2}}{c^{2}}} , \qquad (v^{2}\equiv \delta ^{}_{ij}v^{i}v^{j}) . \end{aligned}$$

At this point, we can now adopt a Newtonian point of view by looking at the geodesic equations for the six quantities: $\{x^i(t),v^i(t)\}$, that is, for the spatial coordinates and spatial velocity components. They can be written as:

209 $$\begin{aligned} \frac{dx^i}{dt}&= v^i , \end{aligned}$$

210 $$\begin{aligned} \frac{dv^i}{dt}&= \,f^i_{g} , \end{aligned}$$

where, as we have mentioned before, the forces, $f^i_{g}$, are actually forces per unit mass, i.e. accelerations, since they should not depend on the mass of the body (according to the equivalence principle). Moreover, these specific forces depend on the spacetime metric (and its first derivatives) and on $v^i$. We can write them as

$$\begin{aligned} f^i_{g} = v^i\,\varGamma ^t_{tt}-\varGamma ^i_{tt} + 2\left( v^i\,\varGamma ^t_{tj}-\varGamma ^i_{tj}\right) v^j + \left( v^i\,\varGamma ^t_{jk}-\varGamma ^i_{jk}\right) v^jv^k . \end{aligned}$$

Given initial conditions $\{x^i_o,v^i_o\}$, Eqs. (209 and 210) have a unique solution $\{x^i(t),v^i(t)\}\,$. Note that the $c^{2}$ factor dividing the forces, when going to the right-hand side of the equation (multiplying the Christoffel symbols) will cancel the $c^{2}$ factor in the denominator of $r^{}_{g}\,$ [see expressions in Eqs. (218)–(223)].

Since up to now, the development has been quite general, let us now consider the case of a non-spinning (Schwarzschild) MBH black hole of mass $M_{\bullet }$. The metric components, in harmonic coordinates, can be written in the following form:

211 $$\begin{aligned} g^{}_{tt}&= - \frac{1-\frac{r^{}_{g}}{r}}{1+\frac{r^{}_{g}}{r}}\, c^{2} , \end{aligned}$$

212 $$\begin{aligned} g^{}_{ti}&= 0 , \end{aligned}$$

213 $$\begin{aligned} g^{}_{ij}&= \frac{1+\frac{r^{}_{g}}{r}}{1-\frac{r^{}_{g}}{r}}\, n^{}_{i}n^{}_{j} +\left( 1+\frac{r^{}_{g}}{r}\right) ^{2}\left( \delta ^{}_{ij} - n^{}_{i}n^{}_{j}\right) , \end{aligned}$$

where

214 $$\begin{aligned} r = \sqrt{\delta ^{}_{ij}\,x^{i}x^{j}} , \qquad n^{i} = \frac{x^{i}}{r} ,\qquad r^{}_{g} = \frac{GM_{\bullet }}{c^{2}} . \end{aligned}$$

From here, the components of the inverse metric are:

215 $$\begin{aligned} g^{tt}&= - \frac{1+\frac{r^{}_{g}}{r}}{1-\frac{r^{}_{g}}{r}}\,\frac{1}{c^{2}} , \end{aligned}$$

216 $$\begin{aligned} g^{ti}&= 0 , \end{aligned}$$

217 $$\begin{aligned} g^{ij}&= \frac{1-\frac{r^{}_{g}}{r}}{1+\frac{r^{}_{g}}{r}}\, n^{i}n^{j} +\frac{1}{\left( 1+\frac{r^{}_{g}}{r}\right) ^{2}}\left( \delta ^{ij} - n^{i}n^{j}\right) , \end{aligned}$$

where $$x^{}_{i}=\delta ^{}_{ij}\,x^{j}$$ and $$n^{}_{i} =\delta ^{}_{ij}\,n^{j}$$.

To determine the forces we need to compute the Christoffel symbols. From their definition (202), we find the following result

218 $$\begin{aligned} \varGamma ^{t}_{tt} =&0 , \end{aligned}$$

219 $$\begin{aligned} \varGamma ^{t}_{ti} =&\frac{r^{}_{g}}{r^{2}}\frac{n^{}_{i}}{1-\left( \frac{r^{}_{g}}{r}\right) ^{2}} , \end{aligned}$$

220 $$\begin{aligned} \varGamma ^{t}_{ij} =&0 , \end{aligned}$$

221 $$\begin{aligned} \varGamma ^{i}_{tt} =&\frac{r^{}_{g}}{r^{2}}\frac{1-\frac{r^{}_{g}}{r}}{\left( 1+\frac{r^{}_{g}}{r}\right) ^{3}}\,n^{i}\,c^{2} , \end{aligned}$$

222 $$\begin{aligned} \varGamma ^{i}_{tj} =&0 , \end{aligned}$$

223 $$\begin{aligned} \varGamma ^{i}_{jk} =&\frac{r^{}_{g}}{r^{2}}\frac{1}{1+\frac{r^{}_{g}}{r}} \left[ \left( 1+\frac{r^{}_{g}}{r}\right) \,n^{i} \left( \delta ^{}_{jk}-n^{}_{j}n^{}_{k}\right) - \frac{n^{i}n^{}_{j}n^{}_{k}}{1-\frac{r^{}_{g}}{r}} -2 n^{}_{(j}\left( \delta ^{i}_{k)}- n^{i}n^{}_{k)} \right) \right] . \end{aligned}$$

And this determines completely the geodesic equations of motion in Eqs. (209) and (210).

Finally, we can make a post-Newtonian expansion of the equations of motion. That is, an expansion for $r^{}_{g}/r \ll 1\,$, and $v/c \ll 1\,$. In our case, the expression for the *force* simplifies to [see Eq. (8.8.2) and Eqs. (218)–(223)]:

224 $$\begin{aligned} f^i_{g} = -\varGamma ^i_{tt} + 2\,v^i\,\varGamma ^t_{tj}v^j -\varGamma ^i_{jk}v^jv^k . \end{aligned}$$

Expanding this we get:

225 $$\begin{aligned} f^{i}_{g} =&-\frac{r^{}_{g}c^{2}}{r^{2}}\left[ 1 - 4\frac{r^{}_{g}}{r} + 9\left( \frac{r^{}_{g}}{r}\right) ^{2} - 16\left( \frac{r^{}_{g}}{r}\right) ^{3} \right] \,n^{i} + 2\,\frac{r^{}_{g}c^{2}}{r^{2}} \left[ 1+\left( \frac{r^{}_{g}}{r}\right) ^{2}\right] \left( \frac{n^{}_{j}v^{j}}{c}\right) \frac{v^{i}}{c} \nonumber \\&- \frac{r^{}_{g}c^{2}}{r^{2}} \Bigl \{ \,n^{i}\left( \delta ^{}_{jk}-n^{}_{j}n^{}_{k}\right) - \left[ 1 + \left( \frac{r^{}_{g}}{r}\right) ^{2} \right] n^{i}n^{}_{j}n^{}_{k} - 2 \left[ 1 - \frac{r^{}_{g}}{r} + \left( \frac{r^{}_{g}}{r}\right) ^{2} - \left( \frac{r^{}_{g}}{r}\right) ^{3} \right] \nonumber \\&n^{}_{(j}\left( \delta ^{i}_{k)}- n^{i}n^{}_{k)} \right) \Bigr \} \times \frac{v^{j}}{c}\frac{v^{k}}{c} , \end{aligned}$$

where the first two rows correspond to the first two terms in Eq. (224). We have expanded in Taylor series the functions of $r^{}_{g}/r$ up to order $(r^{}_{g}/r)^{4}\,$. We can now collect the terms and we find the following expression, which is valid to order 2PN [see Eq. (180) below]:

226 $$\begin{aligned} f^{i}_{g} = - \frac{GM_{\bullet }}{r^{2}}n^{i} + \frac{GM_{\bullet }}{r^{2}}\Bigl \{ \left( {\mathscr {A}}^{}_\mathrm{1PN} + {\mathscr {A}}^{}_\mathrm{2PN}\right) n^{i} + \frac{\varvec{n\cdot v}}{c} \left( {\mathscr {B}}^{}_\mathrm{1PN} + {\mathscr {B}}^{}_\mathrm{2PN}\right) \frac{v^{i}}{c}\Bigr \} ,\qquad \end{aligned}$$

where

227 $$\begin{aligned} \frac{\varvec{n\cdot v}}{c} =&\frac{\varvec{x}}{cr}\frac{d\varvec{x}}{dt} = \frac{1}{2cr}\frac{d\varvec{x}^{2}}{dt} = \frac{1}{2cr}\frac{dr}{dt} = \frac{\dot{r}}{c} , \nonumber \\ v^{2} =&\varvec{v\cdot v} = \delta ^{}_{ij}v^{i}v^{j} , \end{aligned}$$

and

228 $$\begin{aligned} {\mathscr {A}}^{}_\mathrm{1PN} =&4\frac{r^{}_{g}}{r} - \frac{v^{2}}{c^{2}} , \end{aligned}$$

229 $$\begin{aligned} {\mathscr {A}}^{}_\mathrm{2PN} =&-9\left( \frac{r^{}_{g}}{r}\right) ^{2} + 2 \left( \frac{\varvec{n\cdot v}}{c}\right) ^{2}\frac{r^{}_{g}}{r} , \end{aligned}$$

230 $$\begin{aligned} {\mathscr {B}}^{}_\mathrm{1PN} =&4 , \end{aligned}$$

231 $$\begin{aligned} {\mathscr {B}}^{}_\mathrm{2PN} =&-2\frac{r^{}_{g}}{r} . \end{aligned}$$

#### *N*-body units and conversion

In *N*-body simulations, we use the so-called *N*-body units, as defined in, e.g., the book by Heggie and Hut (2003), although they were introduced in Heggie and Mathieu (1986). In these units, the total mass of the system *M* and *G* are set to unity, $M = G = 1$. Hence, to convert length *r*, mass *m*, time *t* and velocity *v* from *N*-body (“Nbody”) to physical units (“phys”), we need to multiply them by a conversion factor “conv”:

$$\begin{aligned} r_\mathrm{phys}&=r_\mathrm{conv}\cdot r_{_\mathrm{Nbody}}\\ m_\mathrm{phys}&=m_\mathrm{conv}\cdot m_{_\mathrm{Nbody}}\\ t_\mathrm{phys}&=t_\mathrm{conv}\cdot t_{_\mathrm{Nbody}}\\ v_\mathrm{phys}&=v_\mathrm{conv}\cdot v_{_\mathrm{Nbody}}. \end{aligned}$$

We usually fix $r_\mathrm{conv}$ by deciding the size of the system, and $m_\mathrm{conv}$ is fixed to the average mass of a star in the system, so that

$$\begin{aligned} t_\mathrm{conv}=&\sqrt{\frac{r_\mathrm{conv}^{3}}{G\,m_\mathrm{conv}}}\\ v_\mathrm{conv}=&\sqrt{\frac{G\,m_\mathrm{conv}}{r_\mathrm{conv}}}. \end{aligned}$$

## A Comment on the use of the Ancient Greek word barathron and bathron to refer to a black hole

In the literature related to the main subject of this review, I have now and again found the expression peribathron to refer to the concept of periapsis. Some authors use it in the case in which one has a black hole instead of a simple star (for which we have periastron). In this last section, I will try to convince the reader (from the Latin *cum vincere*, i.e., win with her or him the truth) that this is not correct. Nevertheless, if one has an uncontrollable urge to be pedantic, I would instead recommend the term apo- or peribarathron. In the next lines I explain why.

From the *Dictionnaire grec français*, A. Bailly. ed. Hachette, édition n$^\mathrm{o}$44, p. 347 we can find that
 **I** trou profond, d’où: **1** abîme, gouffre, ARSTT. Probl. 26, 28; part. à Athènes, barathre, gouffre où l’on précipitait les condamnés (cf. à Sparte , DT. 7, 133; PLAT. Gorg. 516 e; AR. Nub. 1450 // 2 fig. ruine, perte, DÈM. 101, 1; d’où cause de ruine ou de perte, “un vrai gouffre”, en parl. d’une femme, THÉOPH. (ATH. 587 f) // **II** ornement de femme, AR. fr. 309, 8 // **III** c.  HDT. l. c. v. ce mot (R. *gwera-/ gwre/o,^24^ cf. , lat. uoro, etc.).

On p. 340, we have the definition of 
 surface servant de fondement, d’où: **1** base HDT. 1, 183; part. piédestal d’une statue, HDT. 5, 85; ESCH. Pers. 811; XÉN. Eq. 1, 1 // **2** degré, marche, HDT. 7, 23; SOPH. O. C. 1591; part. degré d’échelle, échelon, EUR. Ph. 1179; HDN. 4, 2, 9; fig. , EUR. Cycl. 352, les approches (litt. les degrés d’un danger)// **3** banc, siège SOPH. O. R. 142; O. C. 101; PLAT. Prot. 315 c; DÉM. 313, 12, etc.;  ( ) SOPH. Ant. 854, le trône (de la Justice)// **4** en gén. fondement solide, d’où sol (d’une maison, SOPH. Aj. 860; d’un pays, SOPH. Aj. 135; Phil. 1000); ou fondations, assises (de la terre, SOPH. O. C. 1662; d’une ville, EUR. Suppl. 1198); fig. fondement, support, PD. O. 13, 6;  EUR. Tr. 47, être ferme sur de solides fondements;  DH. 8, 1; LUC. de fond en comble ( ) The Greek–English Lexicon by Liddell & Scott, gives similar definitions. The short version can be consulted at the website of the Perseus Project.

The barathron (as it has been transliterated, e.g., by García Gual in the Odyssey, is described as an abyss, the pit of Tartarus, even though the name itself has never been employed exclusively—there is no “Barathron” par excellence. Here are the oldest references to it in the Homeric texts (reproduced below). By the way, in the Epic and in the Ionic dialect of Herodotus, one finds not , but the dialect variety , as Bailly indicates. Iliad 8, 14 (Zeus very angry) “Hear me,” said he, “gods and goddesses, that I may speak even as I am minded. Let none of you neither goddess nor god try to cross me, but obey me every one of you that I may bring this matter to an end. If I see anyone acting apart and helping either Trojans or Danaans, he shall be beaten inordinately ere he come back again to Olympus; or I will hurl him down into dark Tartarus far into the **deepest pit** under the earth, where the gates are iron and the floor bronze, as far beneath Hades as heaven is high above the earth, that you may learn how much the mightiest I am among you.”
Odyssey 12, 94 (Spanish, by García Gual) La otra ruta se abre entre dos promontorios. La cima de uno de ellos se clava en el cielo anchuroso (...) Tenebrosa caverna se abre a mitad de su altura orientada a las sombras de ocaso y del Érebo (...) Ni el más hábil arquero podría desde el fondo del barco con su flecha alcanzar la oquedad de la cueva en que Escila vive haciendo sentir desde allí sus terribles aullidos (...) La mitad de su cuerpo se esconde en la cóncava gruta; las cabezas, empero, por fuera del **báratro** horrible van mirando hacie el pie de la escarpa y exploran su presa (...) Regarding this dialect variation ( ), we should note that it has been attested in a shorter or contracted form through syncope: . This is what may lead to the mistake of using “bathron” instead of the correct form “barathron”. No dictionary mentions a potential form  with the appropriate meaning, which might have existed under the same rule as , i.e., as syncope form of the Attic variant .

Chantraine, in his book *La formation des noms en grec ancien*, states that barathron is a word with religious meaning. This should have been formed from an Indo-European root meaning “to devour” plus the suffix -thron ( ), which expresses an instrument, tool or place, much like the suffixes -tron and -terion. Its meaning is therefore something akin to “place of devouring”, “instrument or means of devouring”, “tool of devouring”. English “devour” comes through French from the Latin form voro, from the same Indo-European root.

Bathron, on the other hand, seems to be formed from the same root as the verb , which expresses the action of walking, lean the feet on the floor or in general to do any kind of movement on the floor. To this root one must add the same suffix of before and, so, we have a “place, tool or instrument to lean on”.

In conclusion, I maintain the words “bathron” and “barathron” are are nearly antonyms. “Barathron” expresses what one needs to express in the context of a black hole; “bathron” can be envisaged as the opposite. What’s more, and what’s worse, in modern Greek, a “bathron” is a cesspit.

The Barathron was in ancient Greece a cliff in Athens, below which lay an inaccessible or invisible place, where criminals were thrown to their deaths. This is attested in the works of Aristophanes, Plato and Herodotus (7133), and compiled by Bailly. It is not clear whether this Barathron was a common name or a proper noun, namely a specific cliff. One should look in the “Clouds” of Aristophanes.

According to Bailly, a cliff similar to the Barathron of Athens existed with the name “kaiadas”. So we can choose: peribarathron or perikaiadas.

Nevertheless, I would suggest something different. If you do not fancy periapsis or apoapsis, you could always make up a new word based on a modern language, such as English. In that case, I would advocate for the very nice term my friend Dave J. Vanecek coined in an e-mail exchange: “periholion”, from the English word “hole”.

## Glossary

$$1\,M_\odot $$: 1 solar mass = $$1.99\times 10^{30}\,\mathrm{kg}$$
${\mathscr {M}}_{\bullet }$: Mass of super- or massive black hole
$$1\,\mathrm{pc}$$: 1 parsec $$ \approx 3.09\times 10^{16}\,\mathrm{m}$$
1 Myr/Gyr: One million/billion years
AGN: Active galactic nucleus
BH: Black hole
CO: Compact object (a white dwarf or a neutron star), or a stellar-mass black hole. In general, a collapsed star with a mass $\in [1.4,\,10]\,M_{\odot }$ in this work
DCO: Dark compact object
DF: Dynamical friction
EMRI: Extreme mass ratio inspiral
GC: Galactic centre
GPU: Graphics processing unit
GW/GWs: Gravitational wave/s
HB: Giant stars in the horizontal branch
HST: Hubble space telescope
IMBH: Intermediate-mass black hole ($M \in \,[10^2,\,10^5]\,M_\odot$)
IMF: Initial mass function
IMRI: Intermediate mass ratio inspiral
LISA: Laser interferometer space antenna
LSO: Last stable orbit
MBH: Massive black hole ($M \approx 10^6 M_\odot$)
MC: Monte carlo
MW: Milky Way
NB6: Direct-summation *N*-body6
NS: Neutron star
PN: Post-Newtonian
RG: Red giant
RMS: Root mean square
SMBH: Super massive black hole ($M > 10^6 M_\odot$)
SNR: Signal-to-noise ratio
SPH: Smoothed particle hydronamics
TDE: Tidal disruption event
UCD: Ultra-compact dwarf galaxy
z: Redshift

## Footnotes

1 This term was first employed by John Archibald Wheeler (b. 1911).

2 If we do not take into account the ionising radiation on intergalactic medium.

3 The number of orders of magnitude between two numbers. This means that if we have two numbers within one dex, the ratio between the larger and the smaller number is less than one order of magnitude.

4 With the possible exception of M33 Gebhardt et al. (2001), Merritt et al. (2001) and M31, see e.g., Bender et al. (2005b).

5 http://sci.esa.int/lisapf.

6 In the related literature there exist other terms to refer to the distance of maximum or minimum approach to a black hole; namely *peribarathron* and *apobarathron*, respectively. There seems to be a confusion and wrong use of the later. I discuss this in Sect. A.

7 The terminology is somehow, and as forewarned, misleading; whilst in general we refer to “collisional” to any effect leading to exchange of energy and angular momentum among stars, here I mean real collisions between two stars. For a thorough discussion of the mechanism and an extremely detailed numerical study, I refer the reader to Freitag and Benz (2002).

8 We can relate standard *N*-body time units $T_\mathrm{NB}$ as defined in, e.g., Heggie and Hut (2003) to Fokker–Planck time units $T_\mathrm{FP}$ as follows: $T_\mathrm{FP} = T_\mathrm{NB} \cdot {N}_{\star } / \ln (\gamma \cdot {N}_{\star })$, with ${N}_{\star }$ the number of stars in the system.

9 We note that eight years earlier, the article by Gurevich (1964) had an interesting first idea of this concept: The authors obtained a similar solution for how electrons distribute around a positively charged Coulomb centre.

10 The zero age main sequence (ZAMS) corresponds to the position of stars in the Hertzsprung–Russell diagram where stars begin hydrogen fusion.

11 The systems emits gravitational radiation all the time, but the most important bursts of energy occur at periapsis.

12 This is not strictly true, the spin of the MBH might “push out” the LSO and so Schwarzschild plunges are Kerr EMRIs; see Amaro-Seoane et al. (2013b).

13 This is only meant as a very general illustrative description. I refer the reader to Amaro-Seoane et al. (2007), Babak et al. (2010) for a detailed introduction to the problem of detection and parameter estimation of EMRIs.

14 The reason for the title of this section is that probably the choice of “resonant” for this process is not a good one. Rauch and Tremaine (1996) coined this term thinking of the effect of a resonance between the radial and azimuthal periods in a Keplerian orbit.

15 http://www.apc.univ-paris7.fr/~petiteau/LISACode/Home.html.

16 For the derivation and some examples of values for ${\mathscr {W}}$, I refer the reader to Amaro-Seoane et al. (2013b).

17 The authors obtained a similar solution for how electrons distribute around a positively charged Coulomb centre.

18 A part of this section profits from Amaro-Seoane et al. (2007), though some parts have been significantly expanded and improved.

19 http://astro-gr.org/modelling-galactic-nuclei-self-gravitating-conducting-gas-spheres/.

20 Memory usage is also reduced, scaling like ${\mathscr {O}}(N_\mathrm{r})$ rather than ${\mathscr {O}}(N_\mathrm{r}^2)$.

21 Anyhow, it has been argued that rare deflections with a large angle may play a important role in the vicinity of a BH (Lin and Tremaine 1980).

22 http://astro-gr.org/monte-carlo-simulations-for-stellar-dynamics/.

23 But see Vasiliev (2015), who has developed a MC code base on the Princeton approach. The code features a scheme to deal with asphericity, with the limitation that it assumes isotropy of the population of background stars, so that it cannot model a highly flattened system with significant rotation support.
